# Enantiodivergent Synthesis of Allenes by Point‐to‐Axial Chirality Transfer

**DOI:** 10.1002/anie.201804446

**Published:** 2018-05-30

**Authors:** Roly J. Armstrong, Meganathan Nandakumar, Rafael M. P. Dias, Adam Noble, Eddie L. Myers, Varinder K. Aggarwal

**Affiliations:** ^1^ School of Chemistry University of Bristol Cantock's Close Bristol BS8 1TS UK

**Keywords:** allenes, boronic esters, elimination, enantiodivergent reactions, enantiospecific reactions

## Abstract

An enantiodivergent method for the synthesis of multiply substituted allenes is described. Highly enantioenriched, point‐chiral boronic esters were synthesized by homologation of α‐seleno alkenyl boronic esters with lithiated carbamates and eliminated to form axially chiral allene products. By employing either oxidative or alkylative conditions, both *syn* and *anti* elimination could be achieved with complete stereospecificity. The process enables the synthesis of either *M* or *P* allenes from a single isomer of a point‐chiral precursor and can be employed for the enantioselective assembly of di‐, tri‐, and tetrasubstituted allenes.

Allenes are versatile functional groups that can be employed in a wide range of chemical transformations.^1^ The unique pattern of reactivity displayed by allenes stems from the consecutive arrangement of two orthogonal π‐bonds—a feature that also results in axial chirality. Chiral non‐racemic allenes are extremely valuable intermediates in synthesis and have additional applications in medicinal chemistry, materials science, and catalysis.^2^ Consequently, considerable effort has been placed on the development of asymmetric methods for allene synthesis.^3^, ^4^ Although various elegant strategies have been reported, the most general method involves nucleophilic substitution of enantioenriched propargylic electrophiles (Scheme 1 A).^5^ Whilst broad‐ranging, this method can result in lower enantiospecificity for tetrasubstituted allenes, for which only a handful of enantioselective preparative methods exist.^6^ A further limitation of such strategies is that if the opposite enantiomer of the allene is desired, then the synthesis must be repeated by first preparing the enantiomeric propargylic electrophile.^7^

**Scheme 1:**
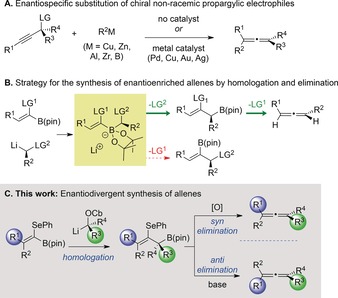
Previous work and our strategy for the enantiodivergent synthesis of allenes.

We envisaged a complementary strategy in which an alkenyl boronic ester bearing an α‐leaving group (LG^1^) is homologated with an enantioenriched lithium carbenoid (Scheme 1 B).^8^ A stereospecific elimination process would then convert the resulting point‐chiral intermediate into an axially chiral allene.^9^ In such a process, there are two critical points at which selectivity must be controlled. First, the boronate complex must undergo the desired 1,2‐metallate rearrangement in which LG^2^ is displaced, rather than a potentially competing rearrangement in which the vinylic leaving group (LG^1^) is expelled.^10^ Second, to obtain complete transfer of stereochemical information, the elimination process must proceed with very high stereospecificity.^11^ The choice of vinylic leaving group (LG^1^) is critical to controlling the selectivity in both of these key steps. We were attracted to selenium as we hoped that its relatively poor leaving group ability would enable it to act as a spectator group during the lithiation–borylation process and then, upon activation, to undergo elimination (Scheme 1 C). Moreover, we have recently shown that β‐seleno boronic esters can undergo selective *anti* elimination in the presence of base or *syn* elimination upon oxidation to the corresponding selenoxide.^12^ Using this strategy, either enantiomer of a given chiral allene could be obtained from a single intermediate in a highly divergent manner. Herein, we describe the successful implementation of this strategy and its application to the enantiodivergent synthesis of di‐, tri‐ and tetrasubstituted allenes.

We commenced our study with alkenyl boronic ester **1**, which was readily prepared as the pure *Z* isomer in three steps from benzaldehyde.^13^ Boronate complex formation was carried out with lithiated carbamate **2**, followed by promotion of the 1,2‐metallate rearrangement by addition of magnesium bromide in methanol and warming to 40 °C [Eq. 1].^14^ Pleasingly, no competing reactions involving displacement of the vinylic selenide were observed, and allylic boronic ester **3** was obtained in 91 % yield and 99:1 e.r.
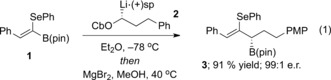



With highly enantioenriched material in hand, we turned our attention to the development of a protocol for *syn* elimination. Upon treatment of a THF solution of boronic ester **3** with *m*‐CPBA at −45 °C, we obtained the desired allene product (*P*)‐**4** in 41 % yield along with alcohol **5** in 41 % yield, which results from competing oxidation of the C−B bond (Table 1, entry 1). We were delighted to find that (*P*)‐**4** was formed with complete enantiospecificity, indicating that the point‐to‐axial chirality transfer had occurred with high fidelity. Reducing the amount of *m*‐CPBA from 2 equiv to 1.2 equiv led to a small improvement in selectivity for elimination over oxidation, and (*P*)‐**4** was obtained in 53 % yield (Table 1, entry 2). Lowering the temperature to −78 °C had very little impact, but carrying out the elimination at 0 °C led to improved selectivity in favour of (*P*)‐**4** (Table 1, entries 3 and 4). Remarkably, when the elimination was performed at room temperature, the desired allene (*P*)‐**4** was formed as the sole product in 88 % yield with complete enantiospecificity (Table 1, entry 5).

**Table 1:** Optimization of the reaction conditions for the *syn* elimination.^[a]^ 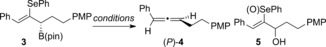

Entry	Conditions	*T* [°C]	Yield **4** [%]^[b]^	Yield **5** [%]^[b,c]^	e.s. **4** [%]^[d]^
1	*m*‐CPBA (2 equiv)	−45	41	41	>99
2	*m*‐CPBA (1.2 equiv)	−45	53	18	>99
3	*m*‐CPBA (2 equiv)	−78	39	42	>99
4	*m*‐CPBA (2 equiv)	0	54	36	>99
5	*m*‐CPBA (2 equiv)	RT	88^[e]^	–	>99

[a] **3** (1.0 equiv), *m‐*CPBA (1.2–2 equiv), THF. [b] Determined by ^1^H NMR analysis with 1,1,2,2‐tetrachloroethane as the internal standard. [c] Yields of **5** refer to combined yields of the selenide and selenoxide (see the Supporting Information for details). [d] Determined by HPLC analysis on a chiral stationary phase. [e] Yield of isolated material. PMP= 4‐methoxyphenyl, pin=pinacolato.

We next focused our attention on developing a procedure for enantiospecific *anti* elimination. Upon employing sodium methoxide in THF, we were disappointed to obtain (*M*)‐**4** in low yield and modest enantiospecificity (6 % yield, 44 % e.s.) along with a significant quantity of alkenyl selenide **6** (Table 2, entry 1). This result suggests that the selenide is too poor a leaving group to compete with facile base‐mediated allylic protodeborylation. We rationalized that if we could convert the selenoether into a better leaving group, the desired elimination process might become the dominant pathway. To test this hypothesis, **3** was transformed into the corresponding selenonium salt by alkylation with MeOTf followed by addition of sodium bicarbonate (Table 2, entry 2). Pleasingly, these conditions resulted in clean elimination to form (*M*)‐**4** in 59 % yield and significantly improved the enantiospecificity (89 % e.s.). Employing aqueous bicarbonate provided (*M*)‐**4** in an improved yield of 85 % with the same enantiospecificity (Table 2, entry 3). We evaluated a range of different aqueous bases (see the Supporting Information for full details) and found that in all cases, (*M*)‐**4** was produced with similar or reduced enantiospecificity (Table 2, entries 4–6). When we carried out the elimination with sodium bicarbonate in methanol, we obtained the desired allene product (*M*)‐**4** with almost complete enantiospecificity (98 % e.s.) in 79 % yield (Table 2, entry 7). Finally, performing the reaction with a reduced quantity of MeOTf (2 equiv) provided (*M*)‐**4** in 83 % yield and 98 % e.s. (Table 2, entry 8).

**Table 2:** Optimization of the reaction conditions for the *anti* elimination.^[a]^ 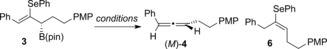

Entry	Conditions	Yield **4** [%]^[b]^	e.s. **4** [%]^[c]^
1^[d]^	NaOMe, THF, RT	6 (+64 % **6**)	44
2	MeOTf, CH_2_Cl_2_; then NaHCO_3_ (s)	59	89
3	MeOTf, CH_2_Cl_2_; then aq. NaHCO_3_	85	89
4	MeOTf, CH_2_Cl_2_; then aq. Na_2_CO_3_	79	86
5	MeOTf, CH_2_Cl_2_; then aq. NaOH	55	84
6	MeOTf, CH_2_Cl_2_; then aq. K_3_PO_4_	74	83
7	MeOTf, CH_2_Cl_2_; then NaHCO_3_, MeOH	79	98
8^[e]^	MeOTf, CH_2_Cl_2_; then NaHCO_3_, MeOH	83^[f]^	98

[a] **3** (1.0 equiv), MeOTf (5 equiv), CH_2_Cl_2_, RT; then base, RT. [b] Determined by ^1^H NMR analysis with 1,1,2,2‐tetrachloroethane as the internal standard. [c] Determined by HPLC analysis on a chiral stationary phase. [d] **3** (1.0 equiv), NaOMe (5 equiv), THF, RT. [e] Alkylation carried out with 2 equiv of MeOTf. [f] Yield of isolated material.

With optimized conditions for homologation and enantiodivergent elimination in hand, we set out to investigate the generality of the process, initially focusing our attention on variations of the alkene partner (Table 3). Introduction of an electron‐rich methoxy substituent was well tolerated, and both enantiomers of the corresponding allene **8** were obtained in 98:2 e.r. and in excellent yields. Electron‐deficient and sterically encumbered alkene partners also smoothly underwent the desired chemistry, providing allene products (*P*)‐**10**, (*M*)‐**10**, (*P*)‐**12**, and (*M*)‐**12** all in excellent yields and with very high enantioselectivity. An alkene partner with an aliphatic side chain (synthesized in two steps from 1‐pentyne) underwent efficient lithiation–borylation (71 % yield, 98:2 e.r.) and, after enantiodivergent elimination, provided allene products (*P*)‐**14** and (*M*)‐**14** in 77 % and 82 % yield, respectively, with complete enantiospecificity. We next investigated variations of the lithium carbenoid partner. We found that boronic ester **1** could be efficiently homologated with an enantioenriched carbenoid containing a silyl ether to form **15**, which underwent enantiospecific elimination affording (*P*)‐**16** (74 % yield, 98:2 e.r.) and (*M*)‐**17** (76 % yield, 98:2 e.r.).^15^, ^16^

**Table 3:** Enantiodivergent synthesis of disubstituted allenes.^[a]^ 
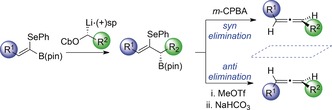

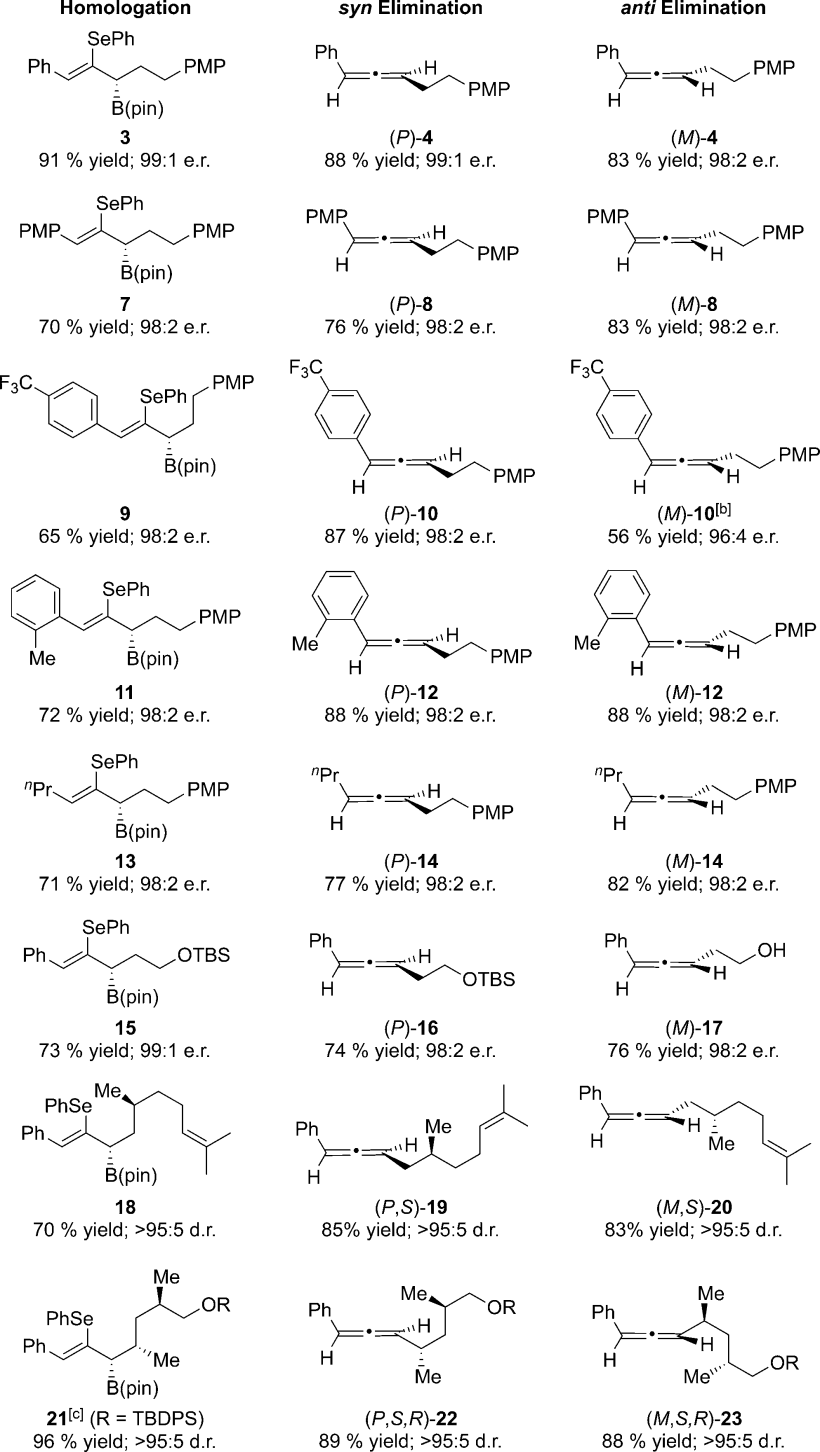

[a] Reaction conditions for the homologation: Carbamate (1.3 equiv), (+)‐sparteine (1.3 equiv), ^*s*^BuLi (1.2 equiv), Et_2_O, −78 °C, 5 h; then the alkenyl boronic ester (1 equiv), −78 °C, 1 h; then MgBr_2_ (2 equiv), −78 °C to reflux, 16 h. For the *syn* elimination: 1,2‐selenoboronic ester (1 equiv), *m*‐CPBA (2.0 equiv), THF, RT, 30 min. For the *anti* elimination: 1,2‐Selenoboronic ester (1 equiv), MeOTf (2 equiv), CH_2_Cl_2_, RT, 16 h; then NaHCO_3_ (20 equiv), CH_2_Cl_2_/MeOH, RT, 3 h. [b] The a*nti* elimination was performed by heating to 40 °C in the absence of NaHCO_3_. See the Supporting Information for the exact conditions. [c] Lithium carbenoid prepared from the corresponding stannane by Sn–Li exchange. Cb=*N*,*N*‐diisopropylcarbamoyl, TBDPS=*tert*‐butyldiphenylsilyl, TBS=*tert*‐butyldimethylsilyl.

Homologation of alkenyl partner **1** with a lithiated carbamate derived from (−)‐citronellol provided boronic ester **18** in 70 % yield as a single diastereoisomer. Elimination of this intermediate under either oxidative or alkylative conditions provided access to either diastereoisomer of the resulting allene (*P*,*S*)‐**19** or (*M*,*S*)‐**20**. Similarly, employing a lithiated carbamate containing two additional stereogenic centres enabled the highly diastereoselective synthesis of allenes (*P*,*S*,*R*)‐**22** and (*M*,*S*,*R*)‐**23**. The modular nature of this synthesis is particularly noteworthy; each alkenyl boronic ester could be paired with a series of different carbamates, rapidly building up a large library of enantioenriched allenes.

We next moved on to investigate the synthesis of trisubstituted allenes (Table 4). Accordingly, we carried out the one‐carbon homologation of **1** with an enantiopure lithiated secondary benzylic carbamate.8c The resulting tertiary allylic boronic ester **24** was obtained in 88 % yield as a single enantiomer. Subjecting this intermediate to our optimized reaction conditions for *syn* and *anti* elimination provided access to either enantiomer of the corresponding trisubstituted allene, (*M*)‐**25** (87 % yield, 99:1 e.r.) and (*P*)‐**25** (84 % yield, 99:1 e.r.). This approach was also successful with cyclic benzylic and aliphatic lithium carbenoids, providing allenes (*M*)‐**27**, (*P*)‐**27**, (*M*)‐**29**, and (*P*)‐**29** all with very high yields and excellent enantioselectivity. We next explored the introduction of an additional substituent on the alkene substrate. Allylic boronic ester **30** was synthesized in very high enantioselectivity by one‐carbon homologation of a tetrasubstituted alkenyl boronic ester. Enantiodivergent elimination afforded allenes (*P*)‐**31** and (*M*)‐**31** in good yields with very high enantiospecificity. Homologation of a tetrasubstituted styrenyl boronic ester afforded enantioenriched tertiary boronic ester **32** in 69 % yield and 97:3 e.r. Both oxidative and alkylative elimination of **32** proceeded smoothly; however, we were surprised to find that both reactions generated the same enantiomer of allene product **33** with high enantiospecificity.

**Table 4:** Enantiodivergent synthesis of tri‐ and tetrasubstituted allenes. 
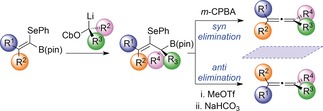

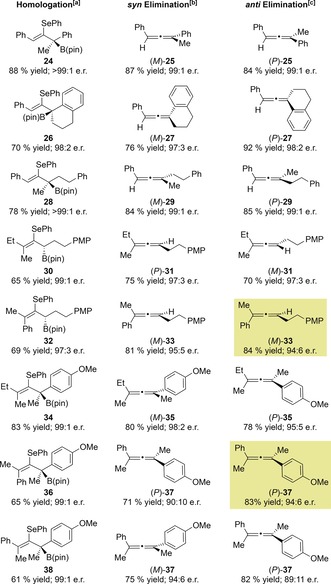

[a] See the Supporting Information for the preparation of the allylic boronic esters. [b] Reaction conditions for the *syn* elimination: 1,2‐Selenoboronic ester (1 equiv), *m*‐CPBA (2.0 equiv), THF, RT, 30 min. [c] Reaction conditions for the *anti* elimination: 1,2‐Selenoboronic ester (1 equiv), MeOTf (2–5 equiv), CH_2_Cl_2_, RT, 16 h; then NaHCO_3_ (20 equiv), CH_2_Cl_2_/MeOH, RT, 3 h.

The asymmetric assembly of tetrasubstituted allenes is known to be particularly challenging, and only a small number of enantioselective methods are currently available for their synthesis.^6^ We therefore set out to determine whether our method could target such materials. Accordingly, allylic boronic ester **34** was synthesized in high enantioselectivity by homologation of a tetrasubstituted alkenyl boronic ester with a lithiated secondary benzylic carbamate. We were delighted to find that **34** underwent elimination to form either enantiomer of the corresponding tetrasubstituted allene, (*M*)‐**35** and (*P*)‐**35**, in good yields and excellent enantiospecificity (98:2 e.r. and 95:5 e.r., respectively). Interestingly, the allylic boronic ester **36**, which bears a phenyl substituent *cis* to the boronic ester, underwent oxidative and alkylative elimination to form the same enantiomer of the tetrasubstituted allene **37**. To determine whether this elimination proceeded by a *syn* or an *anti* pathway, it was necessary to determine the absolute stereochemistry of allene **37**. As this compound was an oil, we were unable to establish its absolute configuration by X‐ray crystallographic analysis. We therefore simulated the electronic circular dichroism spectrum (ECD) of (*P*)‐**37** at the CAM‐B3LYP/6–311(d,p) level of theory.^17^ The simulated ECD spectrum for (*P*)‐**37** was a good match for the experimental spectrum, enabling us to determine that both oxidative and alkylative elimination proceeded via a *syn* mechanism (Scheme 2 a). This inversion of selectivity in the alkylative elimination of **36** and **32** is likely due to the fact that the conformation necessary for *anti* elimination results in significant A‐1,3 strain between the tetravalent boron centre and the bulky phenyl substituent (Scheme 2 b).^18^ In this case, an alternative *syn* elimination pathway with fewer unfavourable steric interactions is preferred. We rationalized that the isomeric allylic boronic ester **38** ought to develop significantly less A‐1,3 strain and might therefore undergo enantiodivergent elimination. Pleasingly, this proved to be the case, and using this approach, both (*M*)‐**37** and (*P*)‐**37** were efficiently synthesized with high enantiospecificity.

**Scheme 2:**
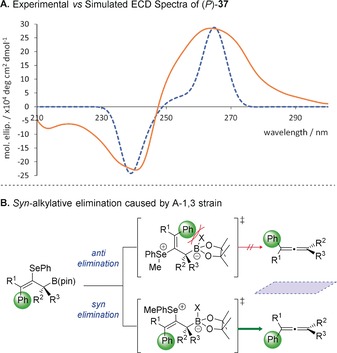
Determination of the absolute configuration for allene (*P*)‐**37** and rationalization of the observed selectivity. a) Experimental ECD spectrum (solid line): 0.23 mm in MeOH, RT. Simulated ECD spectrum (dashed line) calculated at the CAM‐B3LYP/6–311(d,p) level of theory. b) Rationalization of the *syn* elimination, which is driven by A‐1,3 strain.

In conclusion, we have developed a new method for the enantiodivergent synthesis of allenes by point‐to‐axial chirality transfer. Homologation of alkenyl boronic esters with enantioenriched lithium carbenoids followed by *syn* or *anti* elimination provided efficient access to either enantiomer of the resulting allene products. The method is extremely general, enabling the highly convergent synthesis of di‐, tri‐, and even tetrasubstituted allenes bearing a range of different aromatic and aliphatic groups. This method serves as a useful alternative to nucleophilic addition to propargylic electrophiles and will find widespread use for the synthesis of chiral, non‐racemic allenes.

## Conflict of interest

The authors declare no conflict of interest.

## Supporting information

As a service to our authors and readers, this journal provides supporting information supplied by the authors. Such materials are peer reviewed and may be re‐organized for online delivery, but are not copy‐edited or typeset. Technical support issues arising from supporting information (other than missing files) should be addressed to the authors.

Supplementary
