# Supplementary material for: Enantiodivergent Synthesis of Allenes by Point‐to‐Axial Chirality Transfer
Source: Angew Chem Int Ed Engl. 2018 May 30;57(27):8203–8. doi: 10.1002/anie.201804446 (PMC6033179; doi:10.1002/anie.201804446)
Supplement: Supplementary file 1 — Supplementary [file ANIE-57-8203-s001.pdf]

Angewandte Chemie
Eine Zeitschrift der Gesellschaft Deutscher Chemiker

Supporting Information

Enantiodivergent Synthesis of Allenes by Point-to-Axial Chirality Transfer

*Roly J. Armstrong, Meganathan Nandakumar, Rafael M. P. Dias, Adam Noble, Eddie L. Myers, and Varinder K. Aggarwal**

anie_201804446_sm_miscellaneous_information.pdf

Contents

1. General Information **S2**

2. Optimization **S4**

2.1. Optimization of Syn-Elimination **S4**

2.2. Optimization of Anti-Elimination **S6**

3. General Procedures **S8**

4. Experimental Procedures **S10**

4.1. Synthesis of Starting Materials **S10**

4.2. Lithiation–Borylation **S22**

4.3 Enantiodivergent Synthesis of Allenes **S44**

5. *DFT Simulation of Electronic Circular Dichroism (ECD) Spectra* **S72**

6. References **S73**

7. NMR spectra **S74**

1. General Information

Reactions were carried out in flame-dried glassware under an atmosphere of nitrogen unless stated otherwise. Room temperature refers to 20-25 °C. Temperatures of 0 °C were obtained using an ice/water bath. Temperatures of –78 °C were obtained using a dry ice/acetone bath. Temperatures of –45 °C were obtained using a dry ice/acetonitrile bath. Reflux conditions were obtained using an oil bath equipped with a contact thermometer.

Diethyl ether, dichloromethane, tetrahydrofuran and hexane were purified by filtration through activated alumina columns employing the method of Grubbs *et al*.[1] All other solvents were used as supplied without prior purification. *n*-Butyllithium was purchased from Acros Organics as a 1.6 M solution in hexanes and the molarity was established by titration against *N*-benzylbenzamide.[2] *Sec*-Butyllithium was purchased from Acros Organics as a 1.3 M solution in cyclohexane/hexane (92/8) and the molarity was established by titration against *N*-benzylbenzamide.[2] (+)-Sparteine was obtained as the free base (BOC sciences) and distilled over CaH_2. The sparteine free base readily absorbs atmospheric carbon dioxide and should be stored under nitrogen at −20 °C in a Schlenk tube. After homologation reactions, (+)-sparteine was recovered from the acidic aqueous phase by basifying with NaOH (pH=10) followed by extraction with ethyl acetate, concentration and distillation (>80% recovery). All other reagents were used directly as supplied by major chemical suppliers, or following purification procedures described by Perrin and Armarego.[3]

Thin layer chromatography was performed on Merck Kieselgel 60 F_{254} 0.25 mm precoated aluminium plates. Product spots were visualized under UV light (λ = 254 nm) and/or by staining with potassium permanganate solution. Flash chromatography was performed using VWR silica gel 60 (40-63 µm particle size) using head pressure by means of a nitrogen line.

NMR spectroscopy was carried out using Joel Lambda 300, Joel ECP 400, Varian 400-MR, VNMRS500a or Bruker Cryo 500 MHz spectrometers in the deuterated solvent stated, using the residiual non-deuterated solvent signal as an internal reference. Chemical shifts are quoted in ppm

with signal splittings recorded as singlet (s), doublet (d), triplet (t), quartet (q), quintet (qn), sextet (sext), septet (sept), octet (oct), nonet (non) and multiplet (m). The abbreviation br. denotes broad. Coupling constants, *J*, are measured to the nearest 0.1 Hz and are presented as observed.

Infrared spectra were recorded neat on a PerkinElmer Spectrum One FT-IR spectrometer equipped with an attenuated total reflectance attachment with internal calibration. Absorption maxima (λ_{max}) are quoted in wavenumbers (cm^{-1}).

Mass spectra were recorded by the University of Bristol, School of Chemistry departmental mass spectrometry service using electron impact ionisation (EI), chemical ionisation (CI) or electrospray ionisation (ESI) techniques for low- and high-resolution mass spectra. HRMS EI and CI were performed on a VG Analytical Autospec mass spectrometer at 70 eV. HRMS ESI was performed on either a Bruker Daltonics Apex IV, 7-Tesla FT-ICR or microTOF II. HRMS Matrix Assisted Laser Desorption Ionization (MALDI) was performed on a Brüker Daltonics UltrafleXtreme. Samples were submitted in CH_2Cl_2. LRMS EI (GC-MS) was performed on an Agilent 7820A using a HP-5MS UI column (30 m x 0.25 mm x 0.25 µm).

Optical rotations were recorded on a Bellingham and Stanley Ltd. ADP220 polarimeter in a cell with a path length of 1 dm (using the sodium D line, 589 nm). Concentrations are reported in g/100 mL. Temperatures are reported in °C.

Chiral HPLC was performed on an Agilent 1100 Series HPLC unit equipped with UV-vis diode-array detector monitored fitted with the appropriate Daicel Chiralpak column (dimensions: 0.46 cm ø x 25 cm) along with the corresponding guard column (0.4 cm ø x 1 cm). Wavelengths (λ) are reported in nm, retention times (t_R) are reported in minutes and solvent flow rates are reported in mL min^{-1}.

2. Optimization

2.1 Optimization of *syn*-elimination

A solution of **3** (1.0 eq.) in *the appropriate solvent* (0.2 mL/mmol **3**) was stirred at *the appropriate temperature* and *the appropriate oxidant* was added. The resulting solution was stirred at *the appropriate temperature* for 30 min. Dimethylsulfide (20 eq.) was added in a single portion and the resulting solution was stirred at room temperature for 5 min. The solution was diluted with diethyl ether and washed with saturated aqueous sodium carbonate and dried over anhydrous magnesium sulfate, filtered and concentrated under reduced pressure. This solution was analysed by ^{1}H NMR (400 MHz, $CDCl_3$) to determine NMR yields for **4**, **5a** and **5b**. The d.r. of **5b** was determined by ^{1}H NMR. An analytical sample of **4** was obtained by preparative TLC (98:2 pentane/diethyl ether) and was analysed by chiral HPLC to determine the enatiospecificity. Data is provided below for **5a** and **5b**, for characterization of **4** see experimental procedures section.

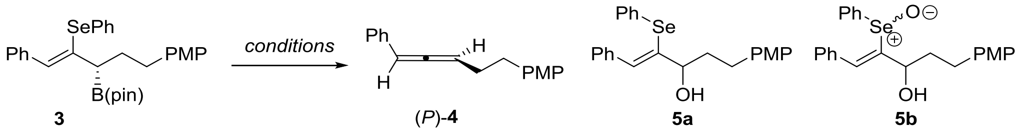

Solvent	Oxidant (eq.)	T / °C	NMR yield **4** / %	e.s. **4** / %	NMR yield **5a**	NMR yield **5b**	d.r. **5b**
THF	*m*CPBA (2.0)	–45	41	>99	<5	41	65:35
THF	*m*CPBA (1.2)	–45	53*	>99	<5	18	12:88
THF	*m*CPBA (2.0)	–78	39	>99	23	19	60:40
THF	*m*CPBA (2.0)	0	54	>99	<5	36	63:37
THF	*m*CPBA (2.0)	r.t.	88†	>99	<5	<5	–
1:1 acetone/H_2O	Oxone® (4.0)	r.t. (16 h)	<5	–	77	<5	–

* A small quantity of starting material **3** (c.a. 10-20 % NMR yield) was also present in the crude reaction mixture.

† Yield of isolated material after column chromatography.

Data for **5a**

^{1}H NMR (500 MHz, $CDCl_3$) δ_H = 7.53 (d, *J* = 7.4 Hz, 2H), 7.42 – 7.38 (m, 2H), 7.35 – 7.25 (m, 4H), 7.24 – 7.18 (m, 3H), 7.09 (d, *J* = 8.5 Hz, 2H), 6.83 (d, *J* = 8.5 Hz, 2H), 4.24 (dd, *J* = 7.9, 4.4 Hz, 1H), 3.81 (s, 3H), 2.73 (ddd, *J* = 13.9, 9.6, 5.5 Hz, 1H), 2.60 (ddd, *J* = 13.9, 9.5, 6.7 Hz, 1H), 2.12 (dddd, *J* = 13.9, 9.4, 6.7, 4.4 Hz, 1H), 1.98 (dddd, *J* = 13.4, 9.3, 7.9, 5.5 Hz, 1H), 1.58 (br s, 1H, O*H*);

^{13}C NMR (126 MHz, $CDCl_3$) δ_C = 157.8, 136.7, 136.5, 133.7, 133.7, 132.3, 129.7, 129.3, 129.3, 129.2, 128.0, 127.8, 127.1, 113.8, 75.8, 55.3, 38.6, 31.0;

FTIR (neat) ν/cm^{-1} = 3600–3200 (br), 2932, 1611, 1578, 1510, 1475, 1438, 1245, 1177, 1034, 737, 692;

HRMS (ESI^+): calculated $[M+Na]^+$ for $C_{24}H_{24}NaO_2Se$ = 447.0834, mass found = 447.0825.

Data for **5b** (Isolated from Table 1a, entry 1)

^{1}H NMR (500 MHz, $CDCl_3$) δ_H = 7.55 – 7.36 (m, 9H), 7.32 – 7.22 (m, 2H), 7.07 (d, *J* = 8.3 Hz, $2H_{min}$), 7.04 (d, *J* = 8.4 Hz, $2H_{maj}$), 6.83 (d, *J* = 8.5 Hz, $2H_{min}$), 6.80 (d, *J* = 8.4 Hz, $2H_{maj}$), 4.78 (dd, *J* = 8.8, 4.5 Hz, $1H_{min}$), 4.60 (dd, *J* = 8.9, 4.5 Hz, $1H_{maj}$), 3.81 (s, 3H), 2.83 – 2.68 (m, 1H), 2.65 – 2.55 (m, 1H), 2.23 – 2.08 (m, 1H), 1.92 – 1.82 (m, 1H);

^{13}C NMR (126 MHz, $CDCl_3$)* δ_C = 157.8 (min), 157.8 (maj), 135.8 (min), 135.5 (maj), 134.3 (min), 134.3 (maj), 133.6 (min), 133.5 (maj), 131.3 (maj), 131.1 (min), 129.7, 129.5, 129.4, 129.4, 129.2, 129.1, 129.0, 129.0, 128.9, 128.7, 127.1 (min), 126.6 (maj), 113.8 (maj + min), 73.8 (min), 71.1 (maj), 55.3 (maj + min), 37.7 (min), 37.4 (maj), 31.1 (min), 31.1 (maj);

FTIR (neat) ν/cm^{-1} = 3500–3000 (br), 2925, 2853, 1611, 1512, 1442, 1246, 1177, 1034, 811, 743, 690;

HRMS (ESI^+): calculated $[M+Na]^+$ for $C_{24}H_{24}NaO_3Se$ = 463.0783, mass found = 463.0780.

* Several of the ^{13}C NMR signals in the region 129.7–128.7 ppm overlapped.

2.2 Optimization of *anti*-elimination

Direct elimination: A solution of **3** (7 mg, 0.013 mmol) in THF (0.14 mL) was stirred at room temperature and sodium methoxide (3.5 mg, 0.065 mmol) was added in a single portion. The resulting suspension was stirred at room temperature for 15 minutes. After this time, the reaction mixture was diluted with diethyl ether and washed with water and dried over anhydrous magnesium sulfate, filtered and concentrated under reduced pressure. The residue was analysed by ^{1}H NMR (400 MHz, $CDCl_3$) to determine ^{1}H NMR yields for **4** (6 % NMR yield), and **6** (64 % NMR yield). An analytical sample of **4** was obtained by preparative TLC (98:2 pentane/diethyl ether) and was analysed by chiral HPLC to determine the enatiospecificity (44 % e.s.). Data is provided below for **6**, for characterization of **4** see experimental procedures section.

Data for **6**

^{1}H NMR (500 MHz, $CDCl_3$) δ_H = 7.41 – 7.37 (m, 2H), 7.30 – 7.19 (m, 6H), 7.11 (d, *J* = 8.3 Hz, 2H), 7.07 (d, *J* = 7.4 Hz, 2H), 6.86 (d, *J* = 8.4 Hz, 2H), 6.09 (t, *J* = 7.3 Hz, 1H), 3.83 (s, 3H), 3.56 (s, 2H), 2.72 (t, *J* = 7.4 Hz, 2H), 2.56 (q, *J* = 7.4 Hz, 2H);

^{13}C NMR (126 MHz, $CDCl_3$) δ_C = 157.9, 138.9, 137.4, 133.4, 133.2, 130.8, 130.3, 129.5, 129.0, 128.6, 128.3, 127.1, 126.2, 113.8, 55.3, 38.4, 34.6, 32.0;

FTIR (neat) ν/cm^{-1} = 3061, 2932, 2834, 1611, 1578, 1511, 1453, 1246, 1177, 1036, 823, 738, 693;

HRMS (ESI^{+}): calculated $[M+Na]^{+}$ for $C_{24}H_{24}NaOSe$ = 431.0885, mass found = 431.0865.

Methylation followed by elimination: A solution of **3** (1 eq.) in dichloromethane (10 mL/mmol **3**) was stirred at room temperature and methyl triflate (5 eq.) was added in a single portion. The vial was tightly capped and was stirred at room temperature for 1 h. After this time the reaction mixture was cooled to *the appropriate temperature* and *the appropriate solvent* was added (10 mL/mmol **3**) followed by *the appropriate base* and the resulting mixture was stirred rapidly at room temperature

for 1 h. The solution was diluted with diethyl ether and washed with water and then dried over anhydrous magnesium sulfate, filtered and concentrated under reduced pressure. The residue was analysed by ^{1}H NMR (400 MHz, $CDCl_3$) to determine an NMR yield for **4**. An analytical sample of **4** was obtained by preparative TLC (98:2 pentane/diethyl ether) and was analysed by chiral HPLC to determine the enatiospecificity. For characterization of **4** see experimental procedures section.

Solvent	Base (eq.)	T / °C	NMR yield **4** / %	e.s. **4** / %
none	$NaHCO_3$ (10)	r.t.	59	89
none	NaCl (10)	r.t.	9	–
none	NaOAc (10)	r.t.	82	96
none	$^{n}Bu_4NBr$ (5)	r.t.	<5*	–
H_2O	none	r.t.	9	–
H_2O	$NaHCO_3$ (sat.)	r.t.	85	89
H_2O	Na_2CO_3 (sat.)	r.t.	79	86
H_2O	NaCl (sat.)	r.t.	5	–
H_2O	NaOH (10)	r.t.	55	84
H_2O	K_3PO_4 (25)	r.t.	74	83
H_2O	K_2HPO_4 (30)	r.t.	94	94
H_2O	K_2HPO_4 (30)	0	57	95
DMSO	K_2HPO_4 (20)	r.t.	75	64
MeOH	none	r.t.	6	–
MeOH	K_2HPO_4 (20)	r.t.	80	97
MeOH	Et_3N (10)	r.t.	83	93
MeOH	$NaHCO_3$ (20)	r.t.	79	98
MeOH	$NaHCO_3$ (20)	0	83	98
MeOH†	$NaHCO_3$ (20)	r.t.	83‡	98

* Along with 76 % NMR yield of starting material **3**, resulting from bromide mediated demethylation.
† With MeOTf (2 eq.) at r.t. for 16 h then MeOH, $NaHCO_3$, r.t., 3 h (see general procedure D).
‡ Yield of isolated material after column chromatography.

3. General Procedures

3.1 General Procedure A: Li–Br Exchange and Borylation

A solution of α-selenobromide (1 eq.) in hexane (5 mL/mmol α-selenobromide) was stirred in a room temperature water bath and a solution of *n*-butyllithium (1.6 M in hexanes, 1.5 eq.) was added dropwise. The resulting milky white suspension was stirred at room temperature for 5 min. After this time, the reaction mixture was cooled to 0 °C and 2-isopropoxy-4,4,5,5-tetramethyl-1,3,2-dioxaborolane (1.5 eq.) was added dropwise. The resulting milky white suspension was warmed to room temperature and stirred for 30 min. After this time water and dichloromethane were added. The organic layer was separated and the aqueous layer was extracted twice with dichloromethane. The combined organic extracts were dried over anhydrous magnesium sulfate, filtered and concentrated under reduced pressure. Purification of the residue (see experimental methods section for specific details) afforded the corresponding α-selenoboronic ester product.

3.2 General Procedure B: Lithiation-Borylation of Primary Carbamates

A stirred solution of primary carbamate (1.3 eq.) and (+)-sparteine (1.3 eq.) in diethyl ether (5 mL/mmol α-selenoboronic ester) was cooled to –78 °C and a solution of *sec*-butyllithium (1.3 M in cyclohexane/hexane, 1.2 eq.) was added dropwise. The resulting solution was stirred at –78 °C for 5 hours and then a solution of α-selenoboronic ester (1 eq.) in diethyl ether (8.5 mL/mmol α-selenoboronic ester) was added dropwise. The resulting solution was stirred at –78 °C for 1 hour and was then warmed to room temperature. A freshly prepared solution of $MgBr_2.OEt_2$ in diethyl ether [prepared as follows: 1,2-dibromoethane (2 eq.) was added to a suspension of magnesium (2 eq.) in Et_2O (2.4 mL/mmol α-selenoboronic ester) at room temperature and the resulting mixture was stirred at room temperature for 1 h] was added. The resulting solution was heated to 40 °C for 16 hours and then cooled to room temperature and diluted with diethyl ether and 1M aqueous HCl. The organic layer was separated and washed with 1M aqueous HCl, saturated aqueous potassium carbonate and brine and then dried over anhydrous magnesium sulfate, filtered and concentrated

under reduced pressure. Purification of the residue (see experimental methods section for specific details) afforded the corresponding β-selenoboronic ester product. Racemic β-selenoboronic esters were obtained by substituting (+)-sparteine with TMEDA.

3.2 General Procedure C: Synthesis of Allenes by Syn-Elimination

A solution of β-selenoboronic ester (1 eq.) in THF (10 mL/mmol β-selenoboronic ester) was stirred in a room temperature water bath and a solution of *m*CPBA (0.2 M in THF, 2 eq.) was added dropwise. The resulting solution was stirred at room temperature for 30 min. Dimethylsulfide (20 eq.) was added in a single portion and the resulting solution was stirred at room temperature for 5 min. The solution was diluted with diethyl ether and washed twice with saturated aqueous sodium carbonate and brine and then dried over anhydrous magnesium sulfate, filtered and concentrated under reduced pressure. Purification of the residue (see experimental methods section for specific details) afforded the corresponding allene product.

3.3 General Procedure D: Synthesis of Allenes by Anti-Elimination

A solution of β-selenoboronic ester (1 eq.) in dichloromethane (10 mL/mmol β-selenoboronic ester) was stirred in a 7 mL vial at room temperature and methyl triflate (2-5 eq.) was added in a single portion. The vial was tightly capped and stirred at room temperature for 16 h. After this time, the cap was removed from the vial, methanol (10 mL/mmol β-selenoboronic ester) was added followed by solid sodium bicarbonate (20 eq.), and the vial was sealed with a subaseal pierced with a nitrogen balloon. The resulting suspension was stirred rapidly at room temperature for 3 h and was then diluted with diethyl ether and water. The organic phase was separated and washed with brine and then dried over anhydrous magnesium sulfate, filtered and concentrated under reduced pressure. Purification of the residue (see experimental methods section for specific details) afforded the corresponding allene product.

4. Experimental Procedures

4.1 Synthesis of Starting Materials

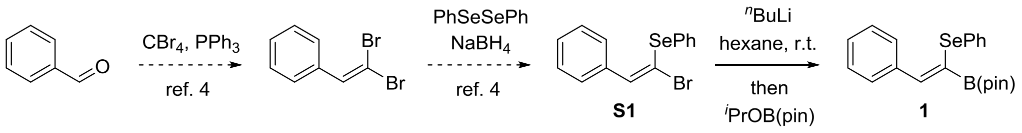

(*Z*)-4,4,5,5-Tetramethyl-2-(2-phenyl-1-(phenylselanyl)vinyl)-1,3,2-dioxaborolane, 1

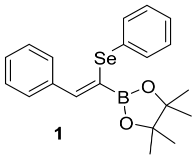

1

According to **General Procedure A** with α-selenobromide **S1**[4] (1.23 g, 3.63 mmol). Purification *via* column chromatography eluting with 95:5 pentane/diethyl ether afforded the title compound **1** as a white solid (969 mg, 69 %, 99.3:0.7 *Z*/*E* by GCMS). Recrystallization of the product from boiling hexane/ethyl acetate afforded the title compound **1** as a white solid (843 mg, 60 %, 99.8:0.2 *Z*/*E* by GCMS). The *Z*/*E* selectivity was confirmed by nOe (see NMR spectra).

Melting point 122–124 ^{o}C (hexane/ethyl acetate);

^{1}H NMR (400 MHz, $CDCl_3$) δ_H = 7.76 (s, 1H), 7.56 – 7.50 (m, 4H), 7.45 – 7.39 (m, 2H), 7.37 – 7.32 (m, 1H), 7.28 – 7.20 (m, 3H), 1.06 (s, 12H);

^{13}C NMR (101 MHz, $CDCl_3$) δ_C = 144.3, 137.5, 133.1, 132.5, 129.5, 128.8, 128.3, 128.0, 126.9, 84.1, 24.4. [*N.B. The carbon attached to boron was not observed due to quadrupolar relaxation*];

^{11}B NMR (96 MHz, $CDCl_3$) δ_B = 29.2;

^{77}Se NMR (57 MHz, $CDCl_3$) δ_{Se} = 366.5;

FTIR (neat) ν/cm^{-1} = 2978, 1589, 1476, 1445, 1372, 1338, 1330, 1142, 978, 856, 753, 740;

HRMS (ESI$^+$): calculated $[M+H]^+$ for $C_{20}H_{24}BO_2Se$ = 387.1029, mass found = 387.1029.

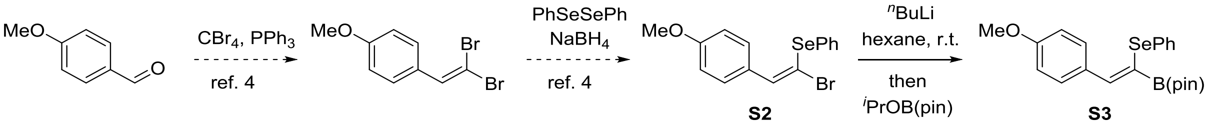

(*Z*)-2-(2-(4-Methoxyphenyl)-1-(phenylselanyl)vinyl)-4,4,5,5-tetramethyl-1,3,2-dioxaborolane, S3

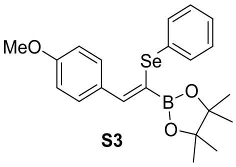

According to **General Procedure A** with α-selenobromide **S2**[4] (800 mg, 2.17 mmol). Purification *via* column chromatography eluting with 90:10 pentane/diethyl ether afforded the title compound **S3** as a white solid (600 mg, 67 %, 99.3:0.7 *Z/E* by GCMS). Recrystallization of the product from boiling hexane/ethyl acetate afforded the title compound **S3** as a white solid (550 mg, 61 %, 99.8:0.2 *Z/E* by GCMS).

Melting point 92–93 °C (hexane/ethyl acetate);

^{1}H NMR (400 MHz, $CDCl_3$) δ_H = 7.70 (s, 1H), 7.53 – 7.48 (m, 4H), 7.21 – 7.19 (m, 3H), 6.92 (d, *J* = 8.8 Hz, 2H), 3.83 (s, 3H), 1.04 (s, 12H);

^{13}C NMR (101 MHz, $CDCl_3$) δ_C = 159.9, 144.7, 133.0, 132.9, 131.4, 130.4, 128.9, 126.9, 113.6, 84.1, 55.4, 24.6 ppm. [*N.B. The carbon attached to boron was not observed due to quadrupolar relaxation*];

^{11}B NMR (96 MHz, $CDCl_3$) δ_B = 29.8;

^{77}Se NMR (57 MHz, $CDCl_3$) δ_{Se} = 371.1;

FTIR (neat) ν/cm^{-1} = 2976, 1586, 1579, 1477, 1445, 1371, 1318, 1267, 1137, 979, 856, 745, 693;

HRMS (ESI$^+$): calculated $[M+H]^+$ for $C_{21}H_{26}BO_3Se$ = 417.1135, mass found = 417.1141;

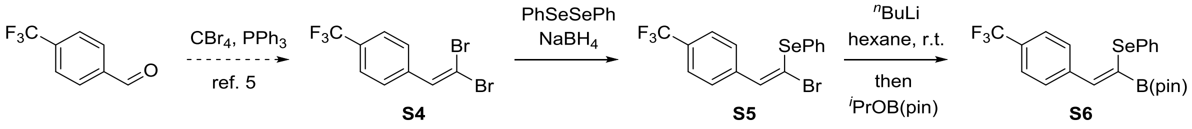

(*E*)-(1-Bromo-2-(4-(trifluoromethyl)phenyl)vinyl)(phenyl)selane, S5

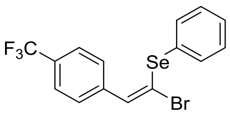

S5

A solution of diphenyl diselenide (473 mg, 1.52 mmol) in PEG-400 (8 mL) was stirred at room temperature under a N_2 atmosphere and sodium borohydride (172 mg, 4.55 mmol) was added portionwise. The resulting mixture was stirred at room temperature for 30 min and then 1,1-dibromoalkene **S4**[5] (1.00 g, 3.03 mmol) was added in a single portion and the temperature was slowly raised to 80 °C. The reaction mixture was stirred at 80 °C for 1 h and then cooled to room temperature and quenched with water and extracted three times with dichloromethane. The combined organic phases were washed with brine, dried over anhydrous magnesium sulfate, filtered and concentrated under reduced pressure. Purification *via* column chromatography eluting with 95:5 hexane/diethyl ether afforded the title compound **S5** as a colourless oil (1.10 g, 90 %).

^{1}H NMR (400 MHz, $CDCl_3$) δ_H = 7.67 – 7.64 (m, 3H), 7.59 – 7.55 (m, 4H), 7.40 – 7.34 (m, 3H).

^{13}C NMR (101 MHz, $CDCl_3$) δ_C = 139.9, 139.2, 134.0, 129.9 (q, *J* = 32.7 Hz), 129.8, 129.4, 128.9, 128.7, 125.3 (q, *J* = 3.8 Hz), 124.0 (q, *J* = 272.2 Hz), 112.52;

^{19}F NMR (377 MHz, $CDCl_3$) δ_F = –62.7;

^{77}Se NMR (76 MHz, $CDCl_3$) δ_{Se} = 523.1;

FTIR (neat) ν/cm^{-1} = 3059, 2999, 1614, 1577, 1476, 1439, 1407, 1319, 1275, 1164, 1120, 1109, 1066, 880, 736;

LRMS (EI^+): calculated $[M]^+$ for $C_{15}H_{10}BrF_3Se$ = 406, mass found = 406;

(*Z*)-4,4,5,5-Tetramethyl-2-(1-(phenylselanyl)-2-(4-(trifluoromethyl)phenyl)vinyl)-1,3,2-dioxaborolane, S6

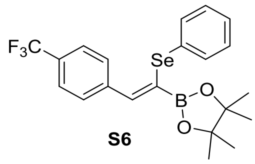

S6

According to **General Procedure A** with α-selenobromide **S5** (1.00 g, 2.46 mmol). Purification *via* column chromatography eluting with 95:5 pentane/diethyl ether afforded the title compound **S6** as a white solid (847 mg, 77%, 99.5:0.5 *Z*/*E* by GCMS).

Melting point 72–74 °C (pentane/diethyl ether);

^{1}H NMR (400 MHz, $CDCl_3$) δ_H = 7.67 (s, 1H), 7.62 (d, *J* = 8.2 Hz, 2H), 7.57 (d, *J* = 8.2 Hz, 2H), 7.50 – 7.47 (m, 2H), 7.24 – 7.20 (m, 3H), 1.03 (s, 12H).

^{13}C NMR (101 MHz, $CDCl_3$) δ_C = 142.0, 140.8, 133.4, 131.7, 129.8 (q, *J* = 32.6 Hz), 129.5, 128.9, 127.3, 125.0 (q, *J* = 3.8 Hz), 124.1 (q, *J* = 272.0 Hz), 84.3, 24.4. [*N.B. The carbon attached to boron was not observed due to quadrupolar relaxation*];

^{19}F NMR (377 MHz, $CDCl_3$) δ_F = –62.5;

^{11}B NMR (96 MHz, $CDCl_3$) δ_B = 29.1;

^{77}Se NMR (76 MHz, $CDCl_3$) δ_{Se} = 374.5;

FTIR (neat) ν/cm^{-1} = 2980, 1578, 1476, 1375, 1320, 1165, 1138, 1123, 1057, 978, 855, 738;

HRMS (ESI^+): calculated $[M+Na]^+$ for $C_{21}H_{22}BF_3O_2SeNa$ = 477.0722, mass found = 477.0718.

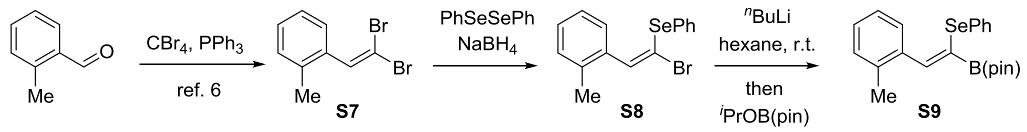

(*E*)-(1-Bromo-2-(*o*-tolyl)vinyl)(phenyl)selane, S8

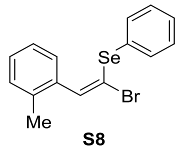

S8

A solution of diphenyl diselenide (2.3 g, 7.2 mmol) in PEG-400 (29 mL) was stirred at room temperature under a N_2 atmosphere and sodium borohydride (840 mg, 21.7 mmol) was added portionwise. The resulting mixture was stirred at room temperature for 30 min and then 1,1-dibromoalkene **S7**[6] (4.00 g, 14.5 mmol) was added in a single portion and the temperature was slowly raised to 80 °C. The resulting solution was stirred at 80 °C for 2h and then cooled to room temperature and quenched with water and extracted three times with dichloromethane. The combined organic phases were washed with brine, dried over anhydrous magnesium sulfate, filtered and concentrated under reduced pressure. Purification *via* column chromatography eluting with 95:5 hexane/diethyl ether afforded the title compound **S8** as a colourless oil (3.88 g, 76 %).

^{1}H NMR (400 MHz, $CDCl_3$) δ_H = 7.54 (s, 1H), 7.47 – 7.38 (m, 2H), 7.26 – 7.19 (m, 4H), 7.17 – 7.04 (m, 3H), 2.21 (s, 3H);

^{13}C NMR (101 MHz, $CDCl_3$) δ_C = 140.5, 136.3, 136.0, 133.9, 130.2, 130.0, 129.2, 129.0, 128.5, 128.3, 125.6, 110.6, 20.0;

^{77}Se NMR (57 MHz, $CDCl_3$) δ_{Se} = 518.2;

FTIR (neat) ν/cm^{-1} = 3011, 2987, 1612, 1563, 1468, 1437, 1401, 1321, 1278, 1164, 1135, 1121, 1103, 736;

HRMS (MALDI): calculated $[M+Na]^+$ for $C_{15}H_{13}BrSeNa$ = 374.9258, mass found = 374.9266.

(*Z*)-4,4,5,5-Tetramethyl-2-(1-(phenylselanyl)-2-(*o*-tolyl)vinyl)-1,3,2-dioxaborolane, S9

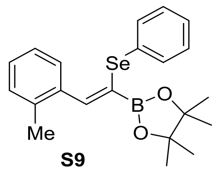

S9

According to **General Procedure A** with *α*-selenobromide **S8** (2.57 g, 7.30 mmol). Purification *via* column chromatography eluting with 95:5 pentane/diethyl ether afforded the title compound **S9** as a white solid (1.84 g, 63 %, 99.6:0.4 *Z*/*E* by GCMS).

Melting point 70–72 °C (pentane/diethyl ether);

^{1}H NMR (400 MHz, $CDCl_3$) = 7.76 (s, 1H), 7.47 – 7.45 (m, 2H), 7.28 – 7.26 (m, 1H), 7.22 – 7.17 (m, 6H), 2.36 (s, 3H), 1.03 (s, 12H);

^{13}C NMR (101 MHz, $CDCl_3$) = 144.2, 137.1, 136.3, 133.0, 132.5, 130.0, 129.0, 128.8, 128.4, 126.8, 125.3, 84.1, 24.5, 20.1 [*N.B. The carbon attached to boron was not observed due to quadrupolar relaxation*];

^{11}B NMR (96 MHz, $CDCl_3$) δ_B = 29.2;

^{77}Se NMR (57 MHz, $CDCl_3$) δ_{Se} = 366.3;

FTIR (neat) ν/cm^{-1} = 2977, 1578, 1476, 1438, 1371, 1329, 1141, 978, 855, 738;

HRMS (ESI$^+$): calculated $[M+Na]^+$ for $C_{21}H_{25}BO_2SeNa$ = 423.1005, mass found = 423.0998;

(*Z*)-4,4,5,5-Tetramethyl-2-(1-(phenylselanyl)pent-1-en-1-yl)-1,3,2-dioxaborolane, S10

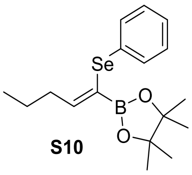

S10

A solution of propylethynyl phenyl selenide[7] (100 mg, 0.45 mmol) in hexane (0.9 mL) was stirred at 0 °C and a solution of DIBAL-H (1.0 M in hexanes, 0.54 mmol) was added dropwise. The resulting mixture was stirred at 0 °C for 1 hour. After this time, the reaction mixture was warmed to room temperature and stirred for 3 hours. The resulting mixture was cooled to -78 °C and 2-isopropoxy-4,4,5,5-tetramethyl-1,3,2-dioxaborolane (0.28 mL, 1.4 mmol) was added dropwise. The resulting solution was warmed to room temperature and stirred for 2 hours. After this time, water and hexane were added. The organic layer was separated and the aqueous layer was extracted twice with ethyl acetate. The combined organic extracts were dried over anhydrous magnesium sulfate, filtered and concentrated under reduced pressure. Purification of the residue *via* column chromatography eluting with 95:5 pentane/diethyl ether afforded the title compound **S10** as a yellow solid (70 mg, 44 %, >99.8:0.2 *Z*/*E* by GCMS). The *Z*/*E* selectivity was confirmed by nOe (see NMR spectra).

Melting point 43–44 °C (hexane);

^{1}H NMR (400 MHz CDCl$_3$) δ_H = 7.37 – 7.34 (m, 2H), 7.15 – 7.08 (m, 3H), 6.80 (t, *J* = 7.0 Hz, 1H), 2.34 – 2.28 (m, 2H), 1.42 (sext, *J* = 7.4 Hz, 2H), 0.99 (s, 12H), 0.88 (t, *J* = 7.4 Hz, 3H);

^{13}C NMR (101 MHz, CDCl$_3$) δ_C = 152.8, 132.7, 132.0, 128.9, 126.3, 83.9, 35.4, 24.6, 22.1, 14.1. [*N.B. The carbon attached to boron was not observed due to quadrupolar relaxation*];

^{11}B NMR (96 MHz, CDCl$_3$) δ_B = 29.1;

^{77}Se NMR (57 MHz, CDCl$_3$) δ_{Se} = 321.3;

FTIR (neat) ν/cm^{-1} = 2977, 2871, 1604, 1578, 1476, 1438, 1379, 1339, 1270, 1145, 976, 851, 736, 690;

HRMS (ESI$^+$): calculated [M+H]$^+$ for $C_{17}H_{26}BO_2Se$ = 353.1186, mass found = 353.1193;

(*Z*)-4,4,5,5-Tetramethyl-2-(2-methyl-1-(phenylselanyl)but-1-en-1-yl)-1,3,2-dioxaborolane, S11

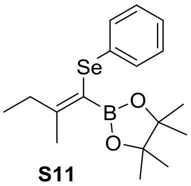

S11

A suspension of zirconocene dichloride (712 mg, 2.39 mmol) in THF (7.9 mL) was stirred under an argon atmosphere at –78 °C and a solution of *n*-BuLi (1.6 M in hexane, 3.0 mL, 4.78 mmol) was added dropwise. The resulting mixture was stirred for 1 h at –78 °C, then an atmosphere of ethylene gas (balloon pressure) was introduced into the vessel for 1 h at –78 °C. The reaction mixture was then slowly warmed to room temperature and 4,4,5,5-tetramethyl-2-(prop-1-yn-1-yl)-1,3,2-dioxaborolane[8] (335 mg, 2.0 mmol) was added and the resulting solution was stirred for 1 h. After this time, isopropanol (120 μL, 1.6 mmol) was added and the reaction mixture was stirred for an additional 1 h. A solution of phenylselenyl chloride (427 mg, 2.20 mmol) in THF (2.2 mL) was then added dropwise and the resulting mixture was stirred overnight at room temperature. The mixture was quenched with 1 M HCl at 0 °C and extracted three times with diethyl ether. The combined organic extracts were washed with brine and then dried over anhydrous magnesium sulfate, filtered and concentrated under reduced pressure. Purification of the residue *via* column chromatography eluting with 98:2 pentane/diethyl ether afforded the title compound **S11** as a brown oil (168 mg, 24 %, >99.8:0.2 *Z*/*E* by GCMS). The *Z*/*E* selectivity was confirmed by nOe (see NMR spectra).

^{1}H NMR (400 MHz CDCl$_3$) δ_H = 7.40 – 7.37 (m, 2H), 7.16 – 7.06 (m, 3H), 2.41 (q, *J* = 7.6 Hz, 2H), 2.01 (s, 3H), 0.97 (t, J = 7.6 Hz, 3H), 0.96 (s, 12 H);

^{13}C NMR (101 MHz, CDCl$_3$) δ_C = 158.7, 132.7, 132.3, 128.9, 126.4, 83.8, 32.3, 24.7, 22.8, 12.8. [*N.B. The carbon attached to boron was not observed due to quadrupolar relaxation*];

^{11}B NMR (96 MHz, CDCl$_3$) δ_B = 28.7;

^{77}Se NMR (57 MHz, $CDCl_3$) δ_{Se} = 353.7;

FTIR (neat) ν/cm^{-1} = 2975, 2931, 1605, 1578, 1477, 1438, 1371, 1335, 1256, 1143, 978, 853, 737, 690;

HRMS (ESI$^+$): calculated $[M+H]^+$ for $C_{17}H_{26}BO_2Se$ = 353.1186, mass found = 353.1203;

(*Z*)-4,4,5,5-Tetramethyl-2-(2-phenyl-1-(phenylselanyl)prop-1-en-1-yl)-1,3,2-dioxaborolane, S12

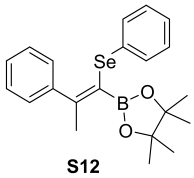

S12

A suspension of zirconocene dichloride (11 mg, 0.04 mmol) in hexane (0.2 mL) was stirred at room temperature under an argon atmosphere and a solution of trimethylaluminium (2.0 M in toluene, 0.49 mL, 0.98 mmol) was added dropwise. The resulting mixture was stirred at room temperature for 5 min. A solution of 2-phenylethynyl phenyl selenide[9] (50 mg, 0.19 mmol) in hexane (45 µL) was added dropwise and the reaction mixture was warmed to 60 °C and stirred for 8 hours. The resulting mixture was cooled to 0 °C and 2-isopropoxy-4,4,5,5-tetramethyl-1,3,2-dioxaborolane (0.16 mL, 0.76 mmol) was added dropwise. The reaction was warmed to room temperature and stirred for 12 hours. The mixture was quenched with 1 M HCl (1.0 mL) at 0 °C and extracted three times with ethyl acetate. The combined organic extracts were dried over anhydrous magnesium sulfate, filtered and concentrated under reduced pressure. Purification of the residue *via* column chromatography eluting with 97:3 pentane/diethyl ether afforded the title compound **S12** as a yellow solid (10 mg, 13 %, >99.8:0.2 *Z*/*E* by GCMS). The *Z*/*E* selectivity was confirmed by nOe (see NMR spectra).

Melting point 64–65 ^{o}C (hexane/ethyl acetate).

^{1}H NMR (400 MHz $CDCl_3$) δ_H = 7.42 – 7.40 (m, 2H), 7.32 – 7.26 (m, 2H), 7.24 – 7.20 (m, 1H), 7.17 – 7.11 (m, 5H), 2.27 (s, 3H), 0.96 (s, 12H);

^{13}C NMR (101 MHz, $CDCl_3$) δ_C = 152.6, 144.6, 133.5, 132.2, 128.8, 128.3, 127.5, 127.2, 126.9, 84.1, 25.9, 24.7 [*N.B. The carbon attached to boron was not observed due to quadrupolar relaxation*];

^{11}B NMR (96 MHz, $CDCl_3$) δ_B = 29.5;

^{77}Se NMR (57 MHz, $CDCl_3$) δ_{Se} = 397.1;

FTIR (neat) ν/cm^{-1} = 3055, 2977, 2930, 1592, 1577, 1476, 1438, 1371, 1321, 1268, 1141, 1022, 699;

HRMS (ESI$^+$): calculated $[M+H]^+$ for $C_{21}H_{26}BO_2Se$ = 401.1186, mass found = 401.1181.

(*E*)-4,4,5,5-Tetramethyl-2-(2-phenyl-1-(phenylselanyl)prop-1-en-1-yl)-1,3,2-dioxaborolane, S13

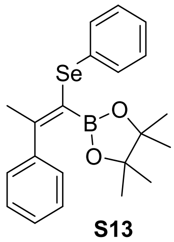

S13

<u>Formation of LiTMP:</u> A stirred solution of 2,2,6,6-tetramethylpiperidine (0.20 mL, 1.18 mmol) in THF (2.0 mL) was cooled to 0 °C and a solution of *n*-butyllithium (1.6 M in hexanes, 0.74 mL, 1.18 mmol) was added dropwise. The resulting mixture was allowed to warm to room temperature and stirred for 30 minutes to form a 0.40 M solution of LiTMP.

A stirred solution of bis[(pinacolato)boryl]methane (100 mg, 0.372 mmol) in THF (0.6 mL) was cooled at 0 °C and a solution of LiTMP (see above, 0.40 M, 1.86 mL, 0.746 mmol) was added dropwise. The resulting solution was stirred at 0 °C for 5 min and then PhSeCl (83 mg, 0.44 mmol) was added in a single portion. The resulting solution was stirred at 0 °C for 10 min and then acetophenone (29 µL, 0.25 mmol) was added. The reaction mixture was stirred at 0 °C for 30 minutes and then allowed to warm to room temperature and stirred for 30 minutes. The crude reaction mixture was filtered through a pad of silica gel washing with diethyl ether and the filtrate was concentrated under reduced pressure. Purification *via* column chromatography eluting with 95:5 pentane/diethyl ether

afforded the title compound **S13** as a colourless solid (43 mg, 43 %, >99.8:0.2 *E*/*Z* by GCMS). The *E*/*Z* selectivity was confirmed by nOe (see NMR spectra).

Melting point 69-71 ^{o}C (pentane/diethyl ether);

^{1}H NMR (500 MHz CDCl$_3$) δ_H = 7.62 – 7.60 (m, 2H), 7.36 – 7.35 (m, 2H), 7.33 – 7.30 (m, 2H), 7.28 – 7.23 (m, 4H), 2.38 (s, 3H), 0.82 (s, 12H).

^{13}C NMR (126 MHz, CDCl$_3$) δ_C = 151.5, 144.6, 133.6, 130.7, 128.8, 128.0, 127.6, 127.4, 127.0, 83.6, 24.8, 24.5. [*N.B. The carbon attached to boron was not observed due to quadrupolar relaxation*];

^{11}B NMR (96 MHz, CDCl$_3$) δ_B = 29.9;

^{77}Se NMR (76 MHz, CDCl$_3$) δ_{Se} = 389.9;

FTIR (neat) ν/cm^{-1} = 2977, 1594, 1475, 1438, 1371, 1323, 1262, 1140, 1022, 981, 851, 763, 735;

HRMS (ESI$^+$): calculated [M+Na]$^+$ for $C_{21}H_{25}BO_2SeNa$ = 423.1005, mass found = 423.0997.

(*S*)-3,7-Dimethyloct-6-en-1-yl diisopropylcarbamate, S14

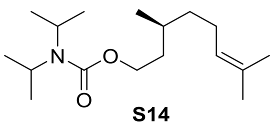

S14

To a flame-dried round-bottomed flask was added *N*,*N*-diisopropylcarbamoyl chloride (5.0 g, 30.7 mmol, 1.2 eq.), triethylamine (4.6 mL, 33.3 mmol, 1.3 eq.), (*S*)-(–)-citronellol (4.0 g, 25.6 mmol), and dichloromethane (25 mL). A reflux condenser was attached, and the reaction mixture was heated at reflux for 24h. After allowing to cool to room temperature, water was added and the mixture was extracted three times with dichloromethane. The combined organic extracts were dried over anhydrous magnesium sulfate, filtered and concentrated. Purification of the residue by column chromatography eluting with 95:5 pentane/diethyl ether afforded the title compound **S14** as a colourless oil (6.9 g, 95 %).

^{1}H NMR (400 MHz, $CDCl_3$) δ_H = 5.02 (t, *J* = 7.1 Hz, 1H), 4.10 – 3.73 (m, 4H), 1.98 – 1.84 (m, 2H), 1.69 – 1.47 (m, 8H), 1.42 – 1.29 (m, 2H), 1.17 – 1.08 (m, 13H), 0.85 (d, *J* = 6.5 Hz, 3H);

^{13}C NMR (101 MHz, $CDCl_3$) δ_C = δ 155.9, 131.2, 124.7, 63.0, 45.8 (br.), 37.0, 36.0, 29.5, 25.7, 25.4, 21.0 (br.), 19.4, 17.6;

FTIR (neat) ν/cm^{-1} = 2965, 2925, 1689, 1434, 1367, 1308, 1288, 1218, 1067, 770, 607;

HRMS (ESI$^+$): calculated $[M+H]^+$ for $C_{17}H_{34}NO_2$ = 284.2584, mass found = 284.2594;

$[\alpha]_D^{21}$ = –0.35 (neat*, density = 1.149 g/mL).

(1*R*,2*S*,4*R*)-5-((*tert*-Butyldiphenylsilyl)oxy)-2,4-dimethyl-1-(trimethylstannyl)pentyl diisopropylcarbamate, S15

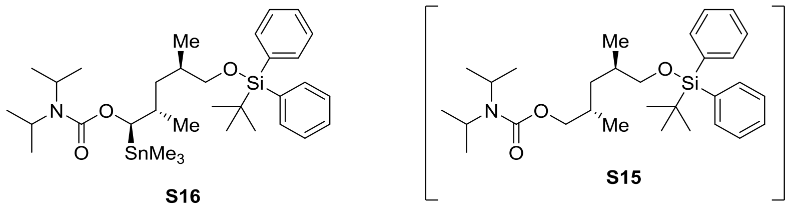

A stirred solution of carbamate **S15**[10] (540 mg, 1.08 mmol) and (+)-sparteine (0.40 mL, 1.73 mmol) in diethyl ether (4.2 mL) was cooled to –78 °C and a solution of *sec*-butyllithium (1.3 M in cyclohexane/hexane, 1.25 mL, 1.63 mmol) was added dropwise. The resulting solution was stirred at –78 °C for 16 hours and then a solution of trimethyltin chloride (1 M in hexanes, 1.84 mL, 1.84 mmol) was added dropwise. The resulting solution was stirred at –78 °C for 1 hour and was then warmed to room temperature and stirred for 30 minutes. After this time, diethyl ether and 3 M aqueous HCl were added. The organic layer was separated and washed with 3 M aqueous HCl, brine, 1 M aqueous NaOH, brine and then dried over anhydrous magnesium sulfate, filtered and concentrated under reduced pressure. Purification of the residue *via* column chromatography

*Measured using a cell with a path length of 0.25 dm.

eluting with 97:3 pentane/diethyl ether afforded the title compound **S16** as a colourless oil (387 mg, 54 %, >95:5 d.r.) along with recovered starting material **S15** (80 mg, 15 %).

^{1}H NMR (500 MHz, $CDCl_3$) δ_H = 7.69 (d, *J* = 7.7 Hz, 4H), 7.47 – 7.37 (m, 6H), 4.54 (d, *J* = 5.9 Hz, 1H), 4.10 (br s, 1H), 3.73 (br s, 1H), 3.56 (dd, *J* = 9.8, 5.1 Hz, 1H), 3.39 (dd, *J* = 9.8, 6.8 Hz, 1H), 2.29 – 2.06 (m, 1H), 1.86 – 1.74 (m, 1H), 1.47 – 1.41 (m, 1H), 1.26 – 1.15 (m, 12H), 1.10 – 1.03 (m, 10H), 1.01 (d, *J* = 6.7 Hz, 3H), 0.97 (d, *J* = 6.6 Hz, 3H), 0.12 (s, 9H);

^{13}C NMR (126 MHz, $CDCl_3$) δ_C = 156.2, 135.6, 135.6, 134.0, 134.0, 129.5, 129.5, 127.6, 78.8, 69.1, 46.3 (br), 45.0 (br), 38.4, 34.8, 33.5, 26.9, 21.5 (br), 20.6 (br), 19.3, 19.2, 18.2, -7.8;

FTIR (neat) ν/cm^{-1} = 2962, 2858, 1674, 1428, 1291, 1111, 1048, 771, 739, 701;

HRMS (ESI$^+$): calculated $[M+Na]^+$ for $C_{33}H_{55}NNaO_3SiSn$ = 684.2865, mass found = 684.2858;

$[\alpha]_D^{23}$ = –14 (*c* = 0.50, $CHCl_3$).

4.2 *Lithiation-Borylation*

(*S*,*Z*)-2-(5-(4-Methoxyphenyl)-1-phenyl-2-(phenylselanyl)pent-1-en-3-yl)-4,4,5,5-tetramethyl-1,3,2-dioxaborolane, 3

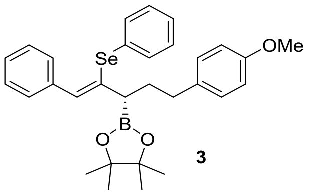

3

According to **General Procedure B** with 3-(4-methoxyphenyl)propyl diisopropylcarbamate[11] (198 mg, 0.675 mmol) and **1** (200 mg, 0.519 mmol). Purification *via* column chromatography eluting with 93:7 pentane/diethyl ether afforded the title compound **3** as a colourless oil (252 mg, 91 %, >95:5 *Z*/*E*, 99.2:0.8 e.r.). The *Z*/*E* selectivity was confirmed by nOe (see NMR spectra).

^{1}H NMR (400 MHz, $CDCl_3$) δ_H = 7.45 – 7.40 (m, 4H), 7.30 – 7.24 (m, 2H), 7.21 – 7.13 (m, 4H), 7.02 (d, *J* = 8.6 Hz, 2H), 6.95 (s, 1H), 6.78 (d, *J* = 8.7 Hz, 2H), 3.78 (s, 3H), 2.48 (t, *J* = 8.0 Hz, 2H), 2.23 (t, *J* = 7.5 Hz, 1H), 2.05 – 1.88 (m, 2H), 1.25 (s, 6H), 1.25 (s, 6H);

^{13}C NMR (101 MHz, $CDCl_3$) δ_C = 157.7, 138.1, 135.9, 134.7, 133.5, 131.9, 130.3, 129.4, 129.1, 128.9, 127.9, 127.0, 126.9, 113.8, 83.6, 55.3, 34.3, 34.1, 24.9, 24.8. [*N.B. The carbon attached to boron was not observed due to quadrupolar relaxation*];

^{11}B NMR (128 MHz, $CDCl_3$) δ_B = 33.6;

^{77}Se NMR (57 MHz, $CDCl_3$) δ_{Se} = 394.8;

FTIR (neat) ν/cm^{-1} = 2978, 2931, 1611, 1511, 1476, 1370, 1360, 1323, 1245, 1142, 1037, 738;

HRMS (ESI$^+$): calculated $[M+Na]^+$ for $C_{30}H_{35}BNaO_3Se$ = 557.1737, mass found = 557.1722;

$[\alpha]_D^{20}$ = –56 (*c* = 0.50, $CHCl_3$);

Chiral HPLC (Chiralpak IB with guard, 1 % IPA, 99 % hexane, 1.0 mL/min, 25 °C, λ = 254 nm, 5 µL injection):

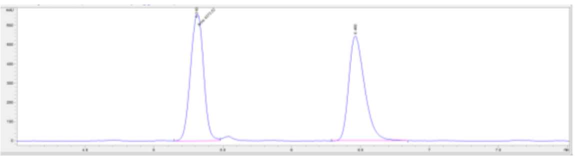

#	Time	Type	Area	Height	Width	Area%	Symmetry
1	5.31	MF	4373.5	659.1	0.1106	49.953	0
2	6.46	VV R	4381.7	545.8	0.0977	50.047	0.688

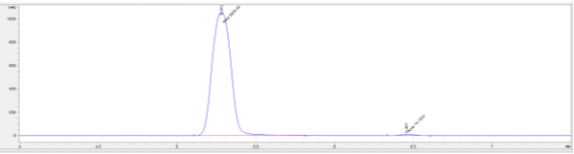

#	Time	Type	Area	Height	Width	Area%	Symmetry
1	5.281	MM	8659.5	1062.9	0.1358	99.150	0.978
2	6.457	MM	74.2	9.9	0.1255	0.850	0.762

(*S*,*Z*)-2-(1,5-Bis(4-methoxyphenyl)-2-(phenylselanyl)pent-1-en-3-yl)-4,4,5,5-tetramethyl-1,3,2-dioxaborolane, 7

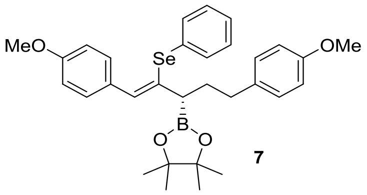

7

According to **General Procedure B** with 3-(4-methoxyphenyl)propyl diisopropylcarbamate[11] (276 mg, 0.940 mmol) and **S3** (300 mg, 0.723 mmol). Purification *via* column chromatography eluting with 93:7 pentane/diethyl ether afforded the title compound **7** as a colourless oil (284 mg, 70 %, >95:5 *Z*/*E*, 98.1:1.9 e.r.).

^{1}H NMR (400 MHz, $CDCl_3$) δ_H = 7.45 – 7.38 (m, 4H), 7.19 – 7.14 (m, 3H), 7.01 (d, *J* = 8.6 Hz, 2H), 6.90 (s, 1H), 6.81 (d, *J* = 8.7 Hz, 2H), 6.77 (d, *J* = 8.6 Hz, 2H), 3.78 (s, 3H), 3.77 (s, 3H), 2.47 (t, *J* = 8.0 Hz, 2H), 2.22 (t, *J* = 7.4 Hz, 1H), 2.04 – 1.87 (m, 2H), 1.24 (s, 6H), 1.23 (s, 6H);

^{13}C NMR (101 MHz, $CDCl_3$) δ_C = 158.6, 157.7, 134.7, 133.5, 133.0, 132.1, 130.6, 130.6, 130.5, 129.4, 128.9, 126.8, 113.8, 113.3, 83.5, 55.3, 55.3, 34.4, 34.1, 24.9, 24.8. [*N.B. The carbon attached to boron was not observed due to quadrupolar relaxation*];

^{11}B NMR (128 MHz, $CDCl_3$) δ_B = 32.6;

^{77}Se NMR (95 MHz, $CDCl_3$) δ_{Se} = 385.1;

FTIR (neat) ν/cm^{-1} = 2977, 2932, 1607, 1509, 1359, 1322, 1247, 1176, 1142, 1036, 823, 737;

HRMS (ESI$^+$): calculated $[M+Na]^+$ for $C_{31}H_{37}BNaO_4Se$ = 587.1842, mass found = 587.1857;

$[\alpha]_D^{24}$ = –48 (*c* = 0.50, $CHCl_3$);

Chiral HPLC (Chiralpak IB with guard, 1 % IPA, 99 % hexane, 1.0 mL/min, 25 °C, λ = 254 nm, 5 μL injection):

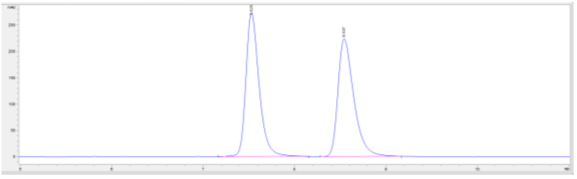

#	Time	Type	Area	Height	Width	Area%	Symmetry
1	7.525	VV R	2763.1	273.9	0.1512	50.575	0.703
2	8.537	VV R	2700.2	223.6	0.1829	49.425	0.598

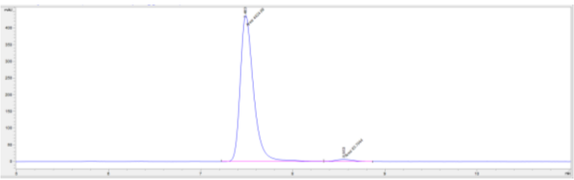

#	Time	Type	Area	Height	Width	Area%	Symmetry
1	7.483	MF	4424.7	439.3	0.1679	98.141	0
2	8.553	FM	83.8	6.3	0.2211	1.859	0.992

(*S*,*Z*)-2-(5-(4-Methoxyphenyl)-2-(phenylselanyl)-1-(4-(trifluoromethyl)phenyl)pent-1-en-3-yl)-4,4,5,5-tetramethyl-1,3,2-dioxaborolane, 9

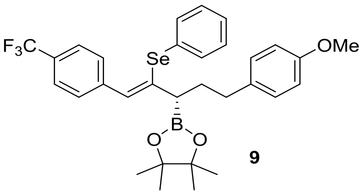

9

According to **General Procedure B** with 3-(4-methoxyphenyl)propyl diisopropylcarbamate[11] (210 mg, 0.717 mmol) and **S6** (250 mg, 0.552 mmol). Purification *via* column chromatography eluting with 93:7 pentane/diethyl ether afforded the title compound **9** as a colourless oil (216 mg, 65 %, >95:5 *Z*/*E*, 98.3:1.7 e.r.).

^{1}H NMR (400 MHz, $CDCl_3$) δ_H = 7.40 (s, 4H), 7.34 – 7.29 (m, 2H), 7.12 – 7.04 (m, 3H), 6.95 (d, *J* = 8.6 Hz, 2H), 6.84 (s, 1H), 6.71 (d, *J* = 8.7 Hz, 2H), 3.71 (s, 3H), 2.43 (t, *J* = 8.0 Hz, 2H), 2.19 (t, *J* = 7.5 Hz, 1H), 1.99 – 1.82 (m, 2H), 1.19 (s, 12H);

^{13}C NMR (101 MHz, $CDCl_3$) δ_C = 157.7, 141.6, 139.2, 134.3, 133.6, 129.9, 129.6, 129.3, 129.2, 128.9, 128.5 (q, *J* = 32.3 Hz), 127.2, 124.7 (q, *J* = 3.7 Hz), 124.3 (q, *J* = 271.8 Hz), 113.7, 83.6, 55.3, 34.2, 33.9, 24.8, 24.7. [*N.B. The carbon attached to boron was not observed due to quadrupolar relaxation*];

^{19}F NMR (377 MHz, $CDCl_3$) δ_F = –62.5;

^{11}B NMR (128 MHz, $CDCl_3$) δ_B = 34.4;

^{77}Se NMR (95 MHz, $CDCl_3$) δ_{Se} = 397.8;

FTIR (neat) ν/cm^{-1} = 2927, 1613, 1512, 1475, 1438, 1371, 1322, 1245, 1164, 1146, 1122, 1066, 822, 737;

HRMS (ESI$^+$): calculated $[M+Na]^+$ for $C_{31}H_{34}BF_3NaO_3Se$ = 625.1611, mass found = 625.1625;

$[\alpha]_D^{23}$ = –56 (*c* = 0.50, $CHCl_3$);

Chiral HPLC (Chiralpak IB with guard, 0.9 % IPA, 99.1 % hexane, 1.0 mL/min, 25 °C, λ = 254 nm, 5 μL injection):

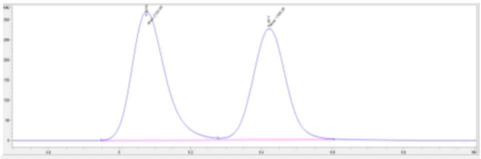

#	Time	Type	Area	Height	Width	Area%	Symmetry
1	5.076	MF	2100.6	319.6	0.1096	54.000	0.761
2	5.421	FM	1789.4	275.2	0.1084	46.000	0.949

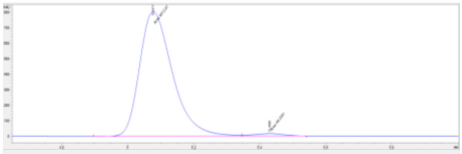

#	Time	Type	Area	Height	Width	Area%	Symmetry
1	5.077	MF	5612.9	808.4	0.1157	98.263	0
2	5.43	FM	99.2	16.3	0.1012	1.737	1.194

(*S*,*Z*)-2-(5-(4-Methoxyphenyl)-2-(phenylselanyl)-1-(o-tolyl)pent-1-en-3-yl)-4,4,5,5-tetramethyl-1,3,2-dioxaborolane, 11

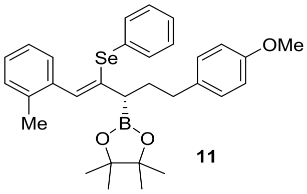

11

According to **General Procedure B** with 3-(4-methoxyphenyl)propyl diisopropylcarbamate[11] (191 mg, 0.651 mmol) and **S9** (200 mg, 0.501 mmol). Purification *via* column chromatography eluting with 93:7 pentane/diethyl ether afforded the title compound **11** as a colourless oil (195 mg, 72 %, >95:5 *Z*/*E*, 98.4:1.6 e.r.).

^1H NMR (400 MHz, $CDCl_3$) δ_H = 7.32 (m, 2H), 7.21 – 7.20 (m, 1H), 7.13 – 7.00 (m, 6H), 6.97 (d, *J* = 8.6 Hz, 2H), 6.82 (s, 1H), 6.72 (d, *J* = 8.6 Hz, 2H), 3.71 (s, 3H), 2.47-2.42 (m, 2H), 2.18 (s, 3H), 2.14 – 2.10 (m, 1H), 1.95-1.87 (m, 2H), 1.20 (s, 12H).

^{13}C NMR (101 MHz, $CDCl_3$)* δ_C = 157.7, 138.0, 137.5, 136.1, 134.6, 134.2, 130.6, 130.1, 129.5, 129.3, 128.7, 127.1, 127.1, 125.2, 113.7, 83.5, 55.3, 34.3, 34.2, 24.9, 24.8, 20.0. [*N.B. The carbon attached to boron was not observed due to quadrupolar relaxation*];

^{11}B NMR (128 MHz, $CDCl_3$) δ_B = 34.3;

^{77}Se NMR (95 MHz, $CDCl_3$) δ_{Se} = 402.2;

FTIR (neat) ν/cm^{-1} = 2976, 2931, 1611, 1511, 1475, 1437, 1328, 1245, 1145, 1034, 736;

HRMS (ESI$^+$): calculated $[M+Na]^+$ for $C_{31}H_{37}BNaO_3Se$ = 571.1893, mass found = 571.1880;

$[\alpha]_D^{23}$ = –46 (*c* = 0.50, $CHCl_3$);

Chiral HPLC (Chiralpak IC, 0.9 % IPA, 99.1 % hexane, 1.0 mL/min, 25 °C, λ = 254 nm, 5 μL injection):

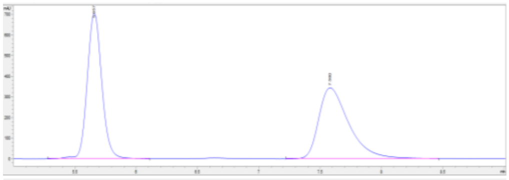

#	Time	Type	Area	Height	Width	Area%	Symmetry
1	5.657	VV R	5843.1	706.5	0.1275	49.799	0.878
2	7.58	BV R	5890.3	344.8	0.2338	50.201	0.572

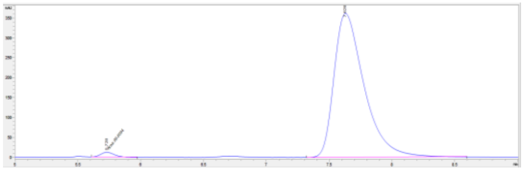

#	Time	Type	Area	Height	Width	Area%	Symmetry
1	5.728	FM	99.6	12.7	0.1306	1.624	0.823
2	7.626	BV R	6035.7	362.4	0.2338	98.376	0.572

(*S*,*Z*)-2-(1-(4-Methoxyphenyl)-4-(phenylselanyl)oct-4-en-3-yl)-4,4,5,5-tetramethyl-1,3,2-dioxaborolane, 13

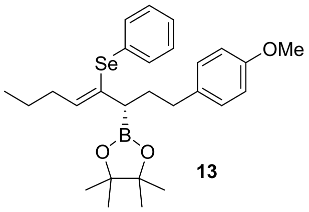

13

According to **General Procedure B** with 3-(4-methoxyphenyl)propyl diisopropylcarbamate[11] (434 mg, 1.48 mmol) and **S10** (400 mg, 1.14 mmol). Purification *via* column chromatography eluting with

* One of the sp^2 carbons could not be observed due to signal overlap.

94:6 pentane/diethyl ether afforded the title compound **13** as a colourless oil (405 mg, 71 %, >95:5 *Z*/*E*, 98.3:1.7 e.r.).

^{1}H NMR (400 MHz CDCl$_3$) δ_H = 7.36 – 7.34 (m, 2H), 7.15 – 7.08 (m, 3H), 6.94 (d, *J* = 8.6 Hz, 2H), 6.70 (d, *J* = 8.6 Hz, 2H), 5.84 (t, *J* = 7.0 Hz, 1H), 3.70 (s, 3H), 2.42 – 2.37 (m, 2H), 2.18 – 2.08 (m, 3H), 1.88 – 1.75 (m, 2H), 1.37 – 1.28 (m, 2H), 1.13 (s, 6H), 1.13 (s, 6H), 0.82 (t, *J* = 7.4 Hz, 3H);

^{13}C NMR (101 MHz, CDCl$_3$) δ_C = 157.8, 136.5, 134.9, 132.7, 131.8, 131.4, 129.4, 129.0, 126.1, 113.9, 83.5, 55.4, 34.6, 34.3, 33.4, 24.9, 24.9, 22.7, 13.9;

^{11}B NMR (96 MHz, CDCl$_3$) δ_B = 32.2;

^{77}Se NMR (57 MHz, CDCl$_3$) δ_{Se} = 400.0;

FTIR (neat) ν/cm^{-1} = 2955, 2929, 1611, 1511, 1476, 1342, 1244, 1140, 1035, 735;

HRMS (ESI$^+$): calculated [M+H]$^+$ for $C_{27}H_{38}BO_3Se$ = 501.2074, mass found = 501.2059.

$[\alpha]_D^{23}$ = +80 (*c* = 0.50, CHCl$_3$).

Chiral HPLC (Chiralpak IC with guard, 2 % IPA, 98 % hexane, 1.0 mL/min, 25 ^{o}C, λ = 254 nm, 5 μL injection):

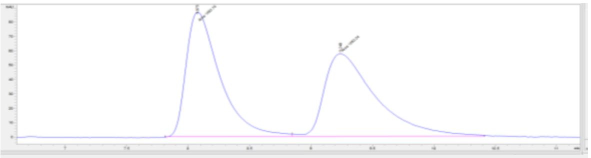

#	Time	Type	Area	Height	Width	Area%	Symmetry
1	8.075	MF	1682.8	87.3	0.3211	49.996	0.485
2	9.24	FM	1683	57.8	0.4853	50.004	0.417

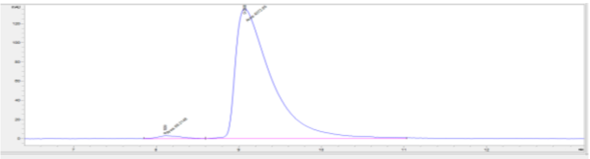

#	Time	Type	Area	Height	Width	Area%	Symmetry
1	8.109	MF	69	3.2	0.3548	1.666	0.498
2	9.068	FM	4072.6	135.5	0.5008	98.334	0.334

(*S*,*Z*)-tert-Butyldimethyl((5-phenyl-4-(phenylselanyl)-3-(4,4,5,5-tetramethyl-1,3,2-dioxaborolan-2-yl)pent-4-en-1-yl)oxy)silane, 15

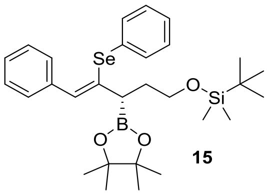

15

According to **General Procedure B** with 3-((tert-butyldimethylsilyl)oxy)propyl diisopropylcarbamate[12] (214 mg, 0.675 mmol) and **1** (200 mg, 0.519 mmol). Purification *via* column chromatography eluting with 93:7 pentane/diethyl ether afforded the title compound **15** as a colourless oil (210 mg, 73 %, >95:5 *Z*/*E*, 98.9:1.1 e.r.).

^{1}H NMR (400 MHz, $CDCl_3$) δ_H = 7.43 – 7.40 (m, 4H), 7.27 – 7.22 (m, 2H), 7.19 – 7.13 (m, 4H), 6.95 (s, 1H), 3.54 (t, *J* = 7.1 Hz, 2H), 2.31 (t, *J* = 7.5 Hz, 1H), 2.00 – 1.89 (m, 2H), 1.24 (s, 6H), 1.23 (s, 6H), 0.86 (s, 9H), 0.00 (s, 6H).

^{13}C NMR (101 MHz, $CDCl_3$) δ_C = 138.0, 135.4, 133.0, 132.3, 130.4, 129.1, 128.8, 127.7, 126.8, 126.8, 83.5, 62.3, 34.7, 26.0, 24.8, 24.7, 18.4, -5.2. [*N.B. The carbon attached to boron was not observed due to quadrupolar relaxation*];

^{11}B NMR (128 MHz, $CDCl_3$) δ_B = 33.2;

^{77}Se NMR (95 MHz, $CDCl_3$) δ_{Se} = 390.3;

FTIR (neat) ν/cm^{-1} = 2958, 2929, 1690, 1578, 1473, 1438, 1370, 1301, 1257, 1141, 1049, 836, 772, 750;

HRMS (ESI$^+$): calculated [M+Na]$^+$ for $C_{29}H_{43}BNaO_3SeSi$ = 581.2132, mass found = 581.2132;

$[\alpha]_D^{23}$ = –16 (*c* = 0.50, $CHCl_3$);

Chiral HPLC (Chiralpak IA, 0.2 % IPA, 99.8 % hexane, 1.0 mL/min, 25 °C, λ = 254 nm, 5 μL injection):

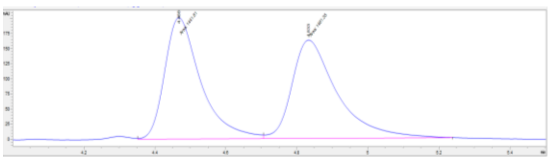

#	Time	Type	Area	Height	Width	Area%	Symmetry
1	4.468	MF	1441.8	202.3	0.1188	49.663	0.621
2	4.833	FM	1461.4	163.9	0.1486	50.337	0.566

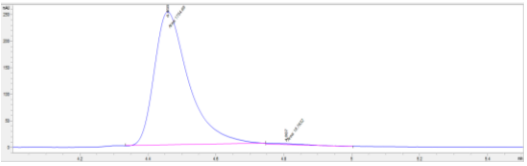

#	Time	Type	Area	Height	Width	Area%	Symmetry
1	4.458	MM	1754.7	250.3	0.1168	98.886	0.621
2	4.807	MM	19.8	2.4	0.1358	1.114	0

2-((3*S*,5*S*,*Z*)-5,9-Dimethyl-1-phenyl-2-(phenylselanyl)deca-1,8-dien-3-yl)-4,4,5,5-tetramethyl-1,3,2-dioxaborolane, 18

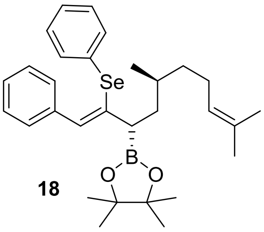

18

According to **General Procedure B** with **S14** (383 mg, 1.35 mmol) and **1** (400 mg, 1.04 mmol). Purification *via* column chromatography eluting with 94:6 pentane/diethyl ether afforded the title compound **18** as a colourless oil (380 mg, 70 %, >95:5 *Z*/*E*, >95:5 d.r.).

^{1}H NMR (400 MHz CDCl$_3$) δ_H = 7.38 – 7.32 (m, 4H), 7.20 – 7.16 (m, 2H), 7.17 – 7.07 (m, 4H), 6.84 (s, 1H), 4.97 – 4.93 (m, 1H), 2.21 (t, *J* = 7.5 Hz, 1H), 1.88 – 1.76 (m, 2H), 1.67 – 1.58 (m, 4H), 1.50 – 1.33 (m, 5H), 1.16 – 1.06 (m, 13H), 0.97 – 0.91 (m, 1H), 0.71 (d, *J* = 7.0 Hz, 3H);

^{13}C NMR (101 MHz, CDCl$_3$) δ_C = 138.3, 136.4, 133.6, 131.7, 131.0, 130.5, 129.2, 128.9, 127.9, 127.0, 126.8, 125.2, 83.5, 39.3, 37.1, 31.4, 25.8, 25.6, 24.8, 24.8, 19.8, 17.8;

^{11}B NMR (96 MHz, CDCl$_3$) δ_B = 31.4;

^{77}Se NMR (57 MHz, CDCl$_3$) δ_{Se} = 398.3;

FTIR (neat) ν/cm^{-1} = 3056, 2975, 2913, 1577, 1476, 1370, 1319, 1267, 1141, 1037, 851, 735;

HRMS (ESI$^+$): calculated [M+H]$^+$ for $C_{30}H_{42}BO_2Se$ = 525.2438, mass found = 525.2443;

$[\alpha]_D^{23}$ = –84 (*c* = 0.50, CHCl$_3$).

***tert*-Butyl(((2*R*,4*S*,5*S*,*Z*)-2,4-dimethyl-7-phenyl-6-(phenylselanyl)-5-(4,4,5,5-tetramethyl-1,3,2-dioxaborolan-2-yl)hept-6-en-1-yl)oxy)diphenylsilane, 21**

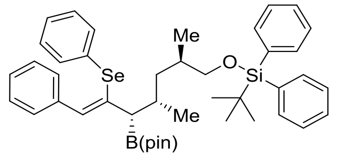

21

A stirred solution of stannane **S16** (200 mg, 0.303 mmol) in diethyl ether (1.5 mL) was cooled to -78 °C and a solution of *n*-butyllithium (1.6 M in hexanes, 0.18 mL, 0.29 mmol) was added dropwise. The resulting solution was stirred at –78 °C for 1 hour and then a solution of boronic ester **1** (93 mg, 0.24 mmol) in diethyl ether (2.2 mL) was added dropwise. The resulting solution was stirred at –78 °C for 1 hour and was then warmed to room temperature. A freshly prepared solution of $MgBr_2.OEt_2$ in diethyl ether [prepared as follows: 1,2-dibromoethane (42 µL, 0.48 mmol) was added to a suspension of magnesium (12 mg, 0.48 mmol) in Et_2O (0.60 mL) at room temperature and the resulting mixture was stirred at room temperature for 1 hour] was added. The resulting solution was heated to 40 °C for 16 hours and the cooled to room temperature and then diluted with diethyl ether and 1 M aqueous HCl. The organic layer was separated and washed with 1 M aqueous HCl, saturated aqueous potassium carbonate and brine and then dried over anhydrous magnesium sulfate, filtered and concentrated under reduced pressure. Purification of the residue *via* column chromatography eluting with 98:2 pentane/diethyl ether afforded the title compound **21** as a colourless oil (171 mg, 96 %, >95:5 *Z*/*E*, >95:5 d.r.).

^{1}H NMR (400 MHz, $CDCl_3$) δ_H = 7.72 – 7.65 (m, 4H), 7.44 – 7.33 (m, 10H), 7.30 – 7.23 (m, 2H), 7.22 – 7.12 (m, 4H), 6.92 (s, 1H), 3.68 (dd, *J* = 9.9, 4.5 Hz, 1H), 3.31 (dd, *J* = 9.9, 7.6 Hz, 1H), 2.03 – 1.91 (m, 1H), 1.79 – 1.69 (m, 1H), 1.36 – 1.26 (m, 1H), 1.20 (s, 6H), 1.17 (s, 6H), 1.06 (s, 9H), 1.01 – 0.86 (m, 5H), 0.82 (d, *J* = 6.3 Hz, 3H);

^{13}C NMR (101 MHz, $CDCl_3$) δ_C = 138.4, 135.8, 135.8, 135.2, 134.3, 134.2, 134.2, 132.3, 130.1, 129.6, 129.5, 129.1, 128.8, 127.9, 127.7, 127.6, 127.2, 126.8, 83.3, 68.9, 40.7, 33.7, 33.3, 27.0, 24.8, 24.8, 19.4, 19.2, 19.1. [*N.B. The carbon attached to boron was not observed due to quadrupolar relaxation*];

^{11}B NMR (128 MHz, $CDCl_3$) δ_B = 32.0;

^{77}Se NMR (57 MHz, $CDCl_3$) δ_{Se} = 415.2;

FTIR (neat) ν/cm^{-1} = 2958, 2929, 2857, 1578, 1474, 1378, 1317, 1142, 1111, 1079, 823, 739, 701;

HRMS (ESI$^+$): calculated [M+Na]$^+$ for $C_{43}H_{55}BNaO_3SeSi$ = 761.3071, mass found = 761.3101;

$[\alpha]_D^{24}$ = +8 (*c* = 0.50, $CHCl_3$).

(*S*,*Z*)-2-(2,4-Diphenyl-3-(phenylselanyl)but-3-en-2-yl)-4,4,5,5-tetramethyl-1,3,2-dioxaborolane, 24

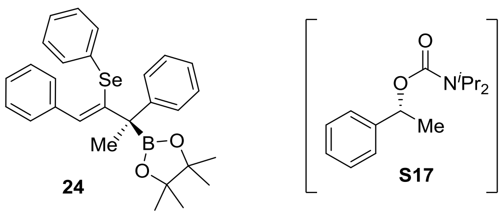

By analogy to a literature procedure,[13] a stirred solution of carbamate **S17**[14] (168 mg, 0.675 mmol, >99:1 e.r.) in diethyl ether (3.4 mL) was cooled to –78 °C and a solution of *sec*-butyllithium (1.3 M in cyclohexane/hexane, 0.50 mL, 0.65 mmol) was added dropwise. The resulting solution was stirred at –78 °C for 15 min and then a solution of **1** (200 mg, 0.519 mmol) in diethyl ether (4.5 mL) was added dropwise. The resulting solution was stirred at –78 °C for 1 hour and then a solution of magnesium bromide (1 M in MeOH, 0.68 mL, 0.68 mmol) was added dropwise. The resulting solution was warmed to room temperature and then heated to 40 °C for 16 hours. The resulting white suspension was cooled to room temperature and diluted with diethyl ether and saturated aqueous sodium carbonate was added. The organic layer was separated and washed with saturated aqueous sodium

carbonate and brine and then dried over anhydrous magnesium sulfate, filtered and concentrated under reduced pressure. Purification of the residue *via* column chromatography eluting with 97.5:2.5 pentane/diethyl ether afforded the title compound **24** as a white solid (223 mg, 88 %, >95:5 *Z*/*E*, >99:1 e.r.).

Melting point 90–92 °C (pentane/diethyl ether);

^{1}H NMR (400 MHz, $CDCl_3$) δ_H = 7.58 (d, *J* = 7.7 Hz, 2H), 7.41 (d, *J* = 7.4 Hz, 2H), 7.35 (t, *J* = 7.6 Hz, 2H), 7.24 (t, *J* = 7.5 Hz, 1H), 7.22 – 7.18 (m, 2H), 7.08 (t, *J* = 7.4 Hz, 2H), 7.03 (t, *J* = 7.1 Hz, 1H), 7.01 – 6.96 (m, 3H), 6.80 (s, 1H), 1.79 (s, 3H), 1.26 (s, 6H), 1.23 (s, 6H);

^{13}C NMR (101 MHz, $CDCl_3$) δ_C = 144.1, 141.9, 137.6, 134.1, 132.6, 130.5, 129.0, 128.7, 128.2, 128.1, 127.4, 126.6, 126.1, 125.5, 84.0, 24.6, 24.5, 23.1. [*N.B. The carbon attached to boron was not observed due to quadrupolar relaxation*];

^{11}B NMR (96 MHz, $CDCl_3$) δ_B = 32.1;

^{77}Se NMR (57 MHz, $CDCl_3$) δ_{Se} = 359.0;

FTIR (neat) ν/cm^{-1} = 3056, 2977, 1578, 1477, 1372, 1321, 1144, 1102, 853, 734;

HRMS (ESI$^+$): calculated $[M+Na]^+$ for $C_{28}H_{31}BNaO_2Se$ = 513.1475, mass found = 513.1479;

$[\alpha]_D^{20}$ = +76 (*c* = 0.50, $CHCl_3$);

Chiral HPLC (Chiralpak IC, 0.1 % IPA, 99.9 % hexane, 1.0 mL/min, 25 °C, λ = 254 nm, 5 μL injection):

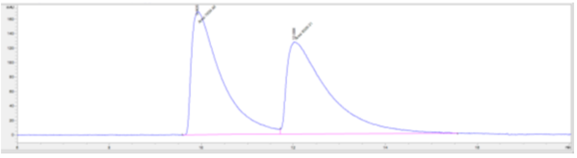

#	Time	Type	Area	Height	Width	Area%	Symmetry
1	9.905	MF	7639.5	170.5	0.7466	48.818	0
2	12.008	FM	8009.3	127.8	1.0445	51.182	0.209

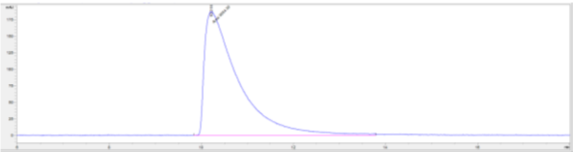

#	Time	Type	Area	Height	Width	Area%	Symmetry
1	10.218	MM	9654.4	188	0.8561	100.000	0.252

(*S*,*Z*)-4,4,5,5-Tetramethyl-2-(1-(2-phenyl-1-(phenylselanyl)vinyl)-1,2,3,4-tetrahydronaphthalen-1-yl)-1,3,2-dioxaborolane, 26

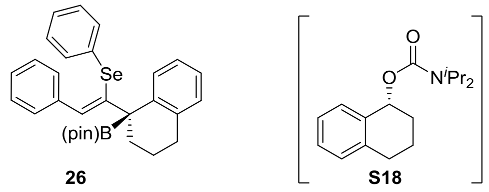

By analogy to a literature procedure,[15] a stirred solution of carbamate **S18**[15] (322 mg, 1.17 mmol, >99:1 e.r.) in diethyl ether (3.2 mL) was cooled to –78 °C and a solution of *sec*-butyllithium (1.3 M in cyclohexane/hexane, 0.84 mL, 1.09 mmol) was added dropwise. The resulting solution was stirred at –78 °C for 1 hour and then a solution of **1** (300 mg, 0.778 mmol) in diethyl ether (7.5 mL) was added dropwise. The resulting solution was stirred at –78 °C for 1 hour and then warmed to –45 °C and stirred for a further 1 hour. After this time, a solution of magnesium bromide (1 M in MeOH, 1.17 mL, 1.17 mmol) was added dropwise. The resulting solution was warmed to room temperature and then heated to 40 °C for 16 hours. The resulting white suspension was cooled to room temperature and diluted with diethyl ether and saturated aqueous sodium carbonate was added. The organic layer was separated and washed with saturated aqueous sodium carbonate and brine and then dried over anhydrous magnesium sulfate, filtered and concentrated under reduced pressure. Purification of the residue *via* column chromatography eluting with 98:2 pentane/diethyl ether afforded the title compound **26** as colourless gum (283 mg, 70 %, >95:5 *Z*/*E*, 98.0:2.0 e.r.).

^{1}H NMR (MHz, $CDCl_3$) δ_H = 7.59 – 7.55 (m, 1H), 7.34 – 7.29 (m, 2H), 7.25 – 7.20 (m, 2H), 7.14 – 7.10 (m, 3H), 7.06 – 6.96 (m, 6H), 6.38 (s, 1H), 2.83 – 2.78 (m, 2H), 2.35 (ddd, *J* = 13.9, 6.2, 2.8 Hz, 1H), 2.21 (ddd, *J* = 13.9, 10.9, 3.0 Hz, 1H), 1.88 – 1.70 (m, 2H), 1.27 (s, 6H), 1.20 (s, 6H);

^{13}C NMR (MHz, $CDCl_3$) δ_C = 142.1, 138.0, 137.7, 137.6, 136.8, 132.6, 131.1, 130.5, 129.7, 129.0, 128.2, 127.4, 126.5, 125.9, 125.5, 125.3, 83.9, 30.6, 30.4, 24.8, 24.5, 18.6. [*N.B. The carbon attached to boron was not observed due to quadrupolar relaxation*];

^{11}B NMR (96 MHz, $CDCl_3$) δ_B = 32.8;

^{77}Se NMR (57 MHz, $CDCl_3$) δ_{Se} = 358.1;

FTIR (neat) ν/cm^{-1} = 2977, 2932, 1578, 1477, 1445, 1371, 1318, 1142, 858, 733;

HRMS (ESI$^+$): calculated $[M+Na]^+$ for $C_{30}H_{33}BNaO_2Se$ = 539.1631, mass found = 539.1629;

$[\alpha]_D^{23}$ = +72 (*c* = 0.50, $CHCl_3$);

Chiral HPLC (Chiralpak IC, 0.1 % IPA, 99.9 % hexane, 1.0 mL/min, 25 °C, λ = 254 nm, 5 μL injection):

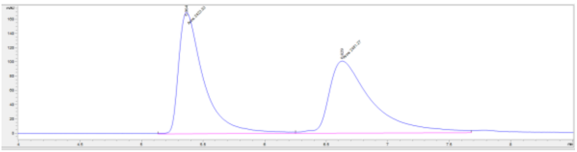

#	Time	Type	Area	Height	Width	Area%	Symmetry
1	5.364	MF	2423.3	171	0.2362	49.409	0.438
2	6.629	FM	2481.3	101.8	0.4062	50.591	0.429

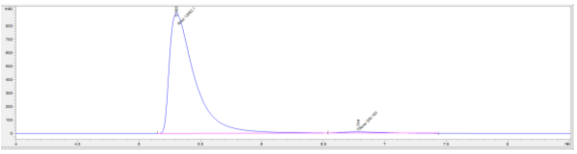

#	Time	Type	Area	Height	Width	Area%	Symmetry
1	5.303	MF	12882.1	898.5	0.2389	97.983	0.351
2	6.784	FM	265.2	11.1	0.398	2.017	0.568

(*S*,*Z*)-4,4,5,5-Tetramethyl-2-(3-methyl-1,5-diphenyl-2-(phenylselanyl)pent-1-en-3-yl)-1,3,2-dioxaborolane, 28

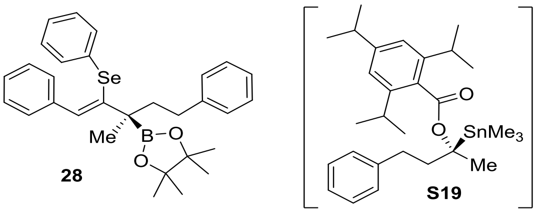

By analogy to a literature procedure,[12] a stirred solution of stannane **S19**[16] (353 mg, 0.649 mmol, >99:1 e.r.) in diethyl ether (3.25 mL) was cooled to –78 °C and a solution of *n*-butyllithium (1.6 M in hexanes, 39 mL, 0.62 mmol) was added dropwise. The resulting solution was stirred at –78 °C for 2 hours and then a solution of **1** (200 mg, 0.519 mmol) in diethyl ether (4.5 mL) was added dropwise. The resulting solution was stirred at –78 °C for 1 hour and then warmed to room temperature and heated to 40 °C for 16 hours. The resulting white suspension was cooled to room temperature and diluted with diethyl ether and saturated aqueous sodium carbonate was added. The organic layer

was separated and washed with saturated aqueous sodium carbonate and brine and then dried over anhydrous magnesium sulfate, filtered and concentrated under reduced pressure. Purification of the residue *via* column chromatography eluting with 97:3 pentane/diethyl ether followed by preparative TLC (95:5 hexane/diethyl ether) afforded the title compound **28** as a colourless oil (210 mg, 78 %, >95:5 *Z*/*E*, >99:1 e.r.).

^{1}H NMR (400 MHz, $CDCl_3$) δ_H = 7.43 – 7.39 (m, 2H), 7.29 – 7.23 (m, 4H), 7.19 – 7.14 (m, 3H), 7.10 – 6.94 (m, 7H), 2.62 (t, *J* = 8.7 Hz, 2H), 2.17 – 1.99 (m, 2H), 1.40 (s, 3H), 1.25 (s, 12H);

^{13}C NMR (101 MHz, $CDCl_3$) δ_C = 143.3, 140.7, 138.0, 132.4, 132.1, 131.0, 129.3, 128.6, 128.4, 128.4, 127.6, 126.6, 125.7, 125.7, 83.8, 39.6, 31.6, 24.9, 24.8, 21.4. [*N.B. The carbon attached to boron was not observed due to quadrupolar relaxation*];

^{11}B NMR (128 MHz, $CDCl_3$) δ_B = 32.8;

^{77}Se NMR (95 MHz, $CDCl_3$) δ_{Se} = 349.3;

FTIR (neat) ν/cm^{-1} = 3058, 3024, 2977, 1578, 1477, 1455, 1438, 1372, 1339, 1317, 1262, 1143, 1098, 966, 853, 735;

HRMS (ESI$^+$): calculated $[M+H]^+$ for $C_{30}H_{36}BO_2Se$ = 519.1968, mass found = 519.1987;

$[\alpha]_D^{24}$ = +38 (*c* = 0.50, $CHCl_3$);

Chiral HPLC (2 x Chiralpak IC, 0.1 % IPA, 99.9 % hexane, 0.5 mL/min, 25 °C, λ = 254 nm, 5 μL injection):

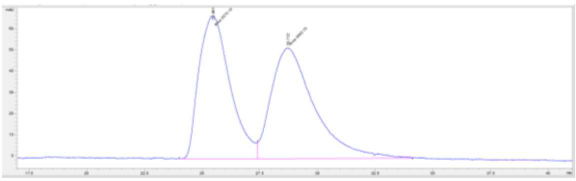

#	Time	Type	Area	Height	Width	Area%	Symmetry
1	25.481	MF	6375.2	67.9	1.5649	48.155	0
2	28.732	FM	6863.8	52.5	2.1788	51.845	0.608

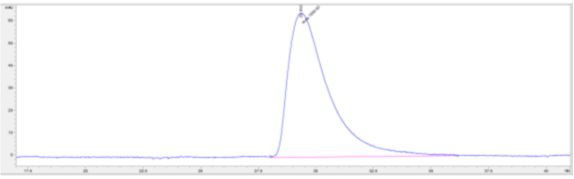

#	Time	Type	Area	Height	Width	Area%	Symmetry
1	29.352	MM	7856.6	64.5	2.03	100.000	0.46

(*S*,*Z*)-2-(1-(4-Methoxyphenyl)-5-methyl-4-(phenylselanyl)hept-4-en-3-yl)-4,4,5,5-tetramethyl-1,3,2-dioxaborolane, 30

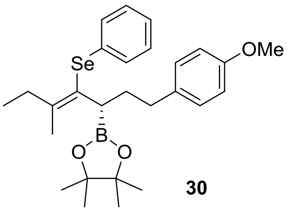

30

According to **General Procedure B** with 3-(4-methoxyphenyl)propyl diisopropylcarbamate[11] (109 mg, 0.371 mmol) and **S11** (100 mg, 0.285 mmol). Purification *via* column chromatography eluting with 95:5 pentane/diethyl ether afforded the title compound **30** as a colourless oil (92 mg, 65 %, >95:5 *Z*/*E*, 98.8:1.2 e.r.).

^{1}H NMR (400 MHz CDCl$_3$) δ_H = 7.33 – 7.30 (m, 2H), 7.13 – 7.03 (m, 3H), 6.92 (d, *J* = 8.5, 2H), 6.69 (d, *J* = 8.5, 2H), 3.69 (s, 3H), 2.54 – 2.25 (m, 5H), 1.99 – 1.90 (m, 1H), 1.77 – 1.69 (m, 4H), 1.08 (s, 6H), 1.06 (s, 6H), 0.87 (t, *J* = 7.5 Hz, 3H);

^{13}C NMR (101 MHz, CDCl$_3$) δ_C = 157.7, 146.0, 135.1, 133.9, 129.9, 129.5, 128.8, 125.4, 125.2, 113.7, 83.4, 55.4, 34.5, 33.3, 33.2, 25.0, 24.8, 18.7, 12.9 [*N.B. The carbon attached to boron was not observed due to quadrupolar relaxation*];

^{11}B NMR (96 MHz, CDCl$_3$) δ_B = 32.9;

^{77}Se NMR (57 MHz, CDCl$_3$) δ_{Se} = 401.6;

FTIR (neat) ν/cm^{-1} = 2975, 2932, 1611, 1511, 1476, 1371, 1351, 1318, 1245, 1143, 1037, 735;

HRMS (ESI$^+$): calculated $[M+Na]^+$ for $C_{27}H_{37}BNaO_3Se$ = 523.1893, mass found = 523.1899;

$[\alpha]_D^{22}$ = +56 (*c* = 0.50, $CHCl_3$);

Chiral HPLC (Chiralpak IB with guard, 1 % IPA, 99 % hexane, 0.5 mL/min, 25 °C, λ = 254 nm, 5 μL injection):

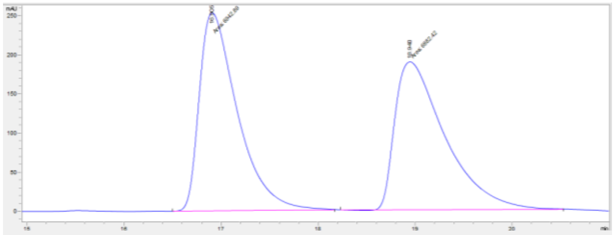

#	Time	Type	Area	Height	Width	Area%	Symmetry
1	16.905	MM	6942.9	253.4	0.4567	50.219	0.488
2	18.94	MM	6882.4	189.9	0.6039	49.781	0.415

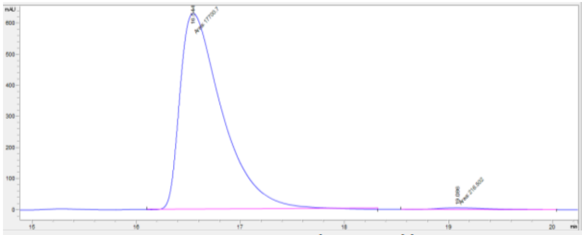

#	Time	Type	Area	Height	Width	Area%	Symmetry
1	16.544	MM	17700.7	630.2	0.4681	98.792	0.434
2	19.086	MM	216.5	5.6	0.6449	1.208	0.622

(*S*,*E*)-2-(1-(4-Methoxyphenyl)-5-phenyl-4-(phenylselanyl)hex-4-en-3-yl)-4,4,5,5-tetramethyl-1,3,2-dioxaborolane, 32

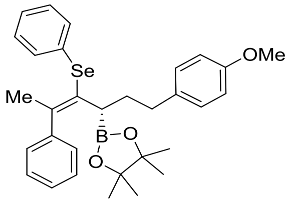

32

According to **General Procedure B** with 3-(4-methoxyphenyl)propyl diisopropylcarbamate[11] (134 mg, 0.457 mmol) and **S13** (140 mg, 0.351 mmol). Purification *via* column chromatography eluting with 93:7 pentane/diethyl ether afforded the title compound **32** as a colourless oil (132 mg, 69%, >95:5 *E*/*Z*, 97.3:2.7 e.r.).

^{1}H NMR (400 MHz, $CDCl_3$) δ_H = 7.51 – 7.48 (m, 2H), 7.27 – 7.15 (m, 7H), 7.11 – 7.06 (m, 1H), 6.77 (d, *J* = 8.6 Hz, 2H), 6.62 (d, *J* = 8.7 Hz, 2H), 3.67 (s, 3H), 2.53 – 2.46 (m, 1H), 2.44 – 2.35 (m, 1H), 2.16 – 2.10 (m, 1H), 2.08 (s, 3H), 1.81 – 1.70 (m, 2H), 1.10 (s, 6H), 1.07 (s, 6H);

^{13}C NMR (101 MHz, $CDCl_3$) δ_C = 157.5, 144.6, 143.6, 135.0, 133.0, 130.4, 129.7, 129.2, 128.8, 128.2, 128.1, 126.6, 125.5, 113.5, 83.4, 55.2, 34.3, 32.9, 27.2, 25.1, 24.5. [*N.B. The carbon attached to boron was not observed due to quadrupolar relaxation*];

^{11}B NMR (128 MHz, $CDCl_3$) δ_B = 33.9;

^{77}Se NMR (95 MHz, $CDCl_3$) δ_{Se} = 338.4;

FTIR (neat) ν/cm^{-1} = 2991, 2933, 1613, 1512, 1476, 1370, 1350, 1320, 1275, 1142, 1023, 750;

HRMS (ESI$^+$): calculated $[M+Na]^+$ for $C_{31}H_{37}BNaO_3Se$ = 571.1893, mass found = 571.1911;

$[\alpha]_D^{26}$ = +81 (*c* = 1, $CHCl_3$);

Chiral HPLC (Chiralpak IB with guard, 0.01 % IPA, 99.99 % hexane, 1.0 mL/min, 25 ^{o}C, λ = 254 nm, 5 μL injection):

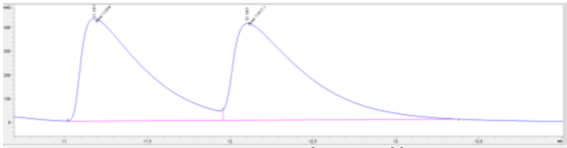

#	Time	Type	Area	Height	Width	Area%	Symmetry
1	11.181	MF	11304	431.5	0.4366	48.680	0
2	12.103	FM	11917.1	406.6	0.4885	51.320	0.234

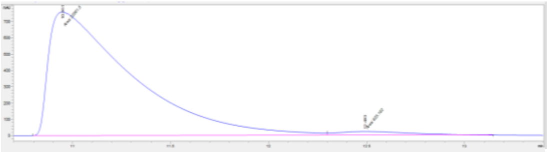

#	Time	Type	Area	Height	Width	Area%	Symmetry
1	10.951	MF	22561.5	763.1	0.4927	97.304	0
2	12.493	FM	625.2	24	0.4346	2.696	0.563

(*S*,*Z*)-2-(2-(4-Methoxyphenyl)-4-methyl-3-(phenylselanyl)hex-3-en-2-yl)-4,4,5,5-tetramethyl-1,3,2-dioxaborolane, 34

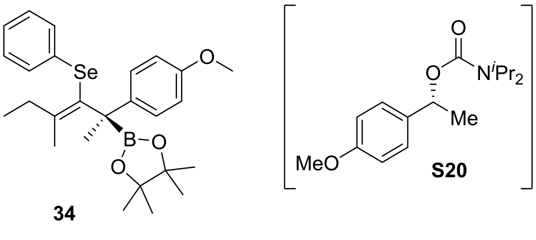

34

By analogy to a literature procedure,[13] a stirred solution of carbamate **S20**[17] (103 mg, 0.369 mmol, >99:1 e.r.) in diethyl ether (1.0 mL) was cooled to –78 ^{o}C and a solution of *sec*-butyllithium (1.3 M in cyclohexane/hexane, 0.26 mL, 0.34 mmol) was added dropwise. The resulting solution was stirred at

–78 ^{o}C for 15 minutes and then a solution of **S11** (100 mg, 0.285 mmol) in diethyl ether (2.7 mL) was added dropwise. The resulting solution was stirred at –78 ^{o}C for 1 hour and a solution of magnesium bromide (1 M in MeOH, 0.43 mL, 0.43 mmol) was added dropwise at the same temperature. The resulting solution was warmed to room temperature and then heated to 40 ^{o}C for 16 hours. The resulting white suspension was cooled to room temperature and diluted with diethyl ether and saturated aqueous sodium carbonate was added. The organic layer was separated and washed with saturated aqueous sodium carbonate and brine and then dried over anhydrous magnesium sulfate, filtered and concentrated under reduced pressure. Purification of the residue *via* column chromatography eluting with 98:2 pentane/diethyl ether afforded the title compound **34** as colourless oil (115 mg, 83 %, >95:5 *Z*/*E*, 99.2:0.8 e.r.).

^{1}H NMR (400 MHz CDCl$_3$) δ_H = 7.29 – 7.23 (m, 4H), 7.13 – 7.02 (m, 3H), 6.72 (d, *J* = 8.9 Hz, 2H), 3.71 (s, 3H), 2.29 – 1.14 (m, 2H), 1.57 (s, 3H), 1.46 (s, 3H), 1.00 (s, 12H), 0.78 (t, *J* = 7.5 Hz, 3H).

^{13}C NMR (101 MHz, CDCl$_3$) δ_C = 157.4, 146.0, 137.9, 135.7, 132.2, 129.2, 128.9, 128.8, 125.3, 113.3, 83.7, 55.3, 34.3, 24.7, 24.5, 21.6, 20.7, 12.4 [*N.B. The carbon attached to boron was not observed due to quadrupolar relaxation*];

^{11}B NMR (96 MHz, CDCl$_3$) δ_B = 32.0;

^{77}Se NMR (57 MHz, CDCl$_3$) δ_{Se} = 401.1;

FTIR (neat) ν/cm^{-1} = 2975, 2931, 1606, 1508, 1476, 1371, 1371, 1318, 1247, 1144, 1087, 734;

HRMS (ESI^{+}): calculated [M+Na]$^{+}$ for $C_{26}H_{35}BNaO_3Se$ = 509.1737, mass found = 509.1742;

$[\alpha]_D^{22}$ = +85 (*c* = 0.50, CHCl$_3$);

Chiral HPLC (Chiralpak IB with guard, 1 % IPA, 99 % hexane, 0.5 mL/min, 25 °C, λ = 254 nm, 5 μL injection):

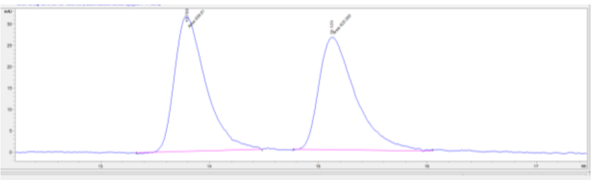

#	Time	Type	Area	Height	Width	Area%	Symmetry
1	13.793	MM	639.8	31.7	0.3362	50.571	0.664
2	15.123	MM	625.4	26.5	0.3927	49.429	0.548

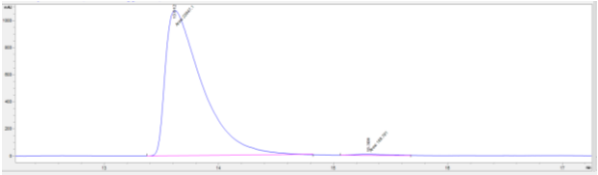

#	Time	Type	Area	Height	Width	Area%	Symmetry
1	13.612	MM	23947.1	1074.9	0.3713	99.218	0.325
2	15.309	MM	188.8	8.4	0.3761	0.782	0.486

(*S*,*E*)-2-(2-(4-Methoxyphenyl)-4-phenyl-3-(phenylselanyl)pent-3-en-2-yl)-4,4,5,5-tetramethyl-1,3,2-dioxaborolane, 36

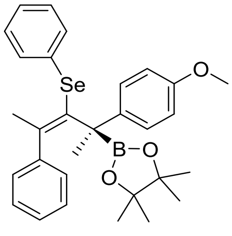

36

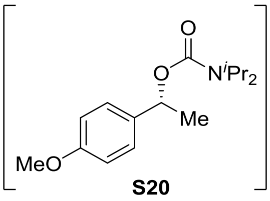

By analogy to a literature procedure,[13] a stirred solution of carbamate **S20**[17] (84 mg, 0.30 mmol, >99:1 e.r.) in diethyl ether (1.4 mL) was cooled to –78 °C and a solution of *sec*-butyllithium (1.3 M in cyclohexane/hexane, 0.25 mL, 0.30 mmol) was added dropwise. The resulting solution was stirred at –78 °C for 15 minutes and then a solution of boronic ester **S13** (100 mg, 0.251 mmol) in diethyl ether (1.3 mL) was added dropwise. The resulting solution was stirred at –78 °C for 1 hour and then a solution of magnesium bromide (1 M in MeOH, 0.38 mL, 0.38 mmol) was added dropwise at the same temperature. The resulting solution was warmed to room temperature and then heated to 40 °C for 16 hours. The resulting white suspension was cooled to room temperature and diluted with diethyl ether and saturated aqueous sodium carbonate was added. The organic layer was separated and washed with saturated aqueous sodium carbonate and brine and then dried over anhydrous magnesium sulfate, filtered and concentrated under reduced pressure. Purification of the residue *via* column chromatography eluting with 97:3 pentane/diethyl ether afforded the title compound **36** (87 mg, 65 %, >95:5 *E*/*Z*, 99.0:1.0 e.r.) as a colourless oil.

^{1}H NMR (400 MHz CDCl$_3$) δ_H = 7.39 (d, *J* = 7.5 Hz, 2H), 7.20 – 7.11 (m, 5H), 7.07 – 7.02 (m, 3H), 7.00 – 6.96 (m, 2H), 6.55 (d, *J* = 8.9 Hz, 2H), 3.66 (s, 3H), 1.97 (s, 3H), 1.73 (s, 3H), 0.89 (s, 6H), 0.75 (s, 6H);

^{13}C NMR (101 MHz, CDCl$_3$) δ_C = 157.4, 144.3, 143.4, 138.4, 136.4, 135.0, 129.6, 129.2, 128.9, 128.6, 127.8, 126.3, 125.5, 113.0, 83.5, 55.2, 30.8, 24.7, 24.1, 22.7.

^{11}B NMR (96 MHz, CDCl$_3$) δ_B = 32.9;

^{77}Se NMR (57 MHz, CDCl$_3$) δ_{Se} = 397.9;

FTIR (neat) ν/cm^{-1} = 2976, 2932, 1604, 1507, 1476, 1371, 1318, 1246, 1143, 1028, 908, 849, 731;

HRMS (ESI$^+$): calculated [M+Na]$^+$ for $C_{30}H_{35}NaBO_3Se$ = 557.1737, mass found = 557.1746;

$[\alpha]_D^{26}$ = +60 (*c* = 1.00, CHCl$_3$);

Chiral HPLC (Chiralpak IC, 0.03 % IPA, 99.97 % hexane, 1.0 mL/min, 25 °C, λ = 254 nm, 5 μL injection):

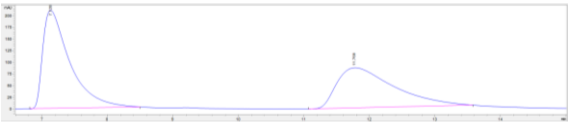

#	Time	Type	Area	Height	Width	Area%	Symmetry
1	7.128	BV R	6048.4	210.4	0.3904	53.672	0.367
2	11.769	BV R	5220.8	86.2	0.7084	46.328	0.442

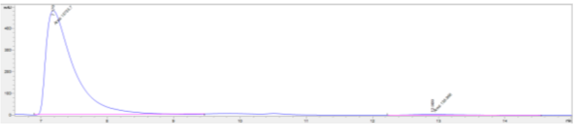

#	Time	Type	Area	Height	Width	Area%	Symmetry
1	7.178	MM	13753.7	480.8	0.4767	98.993	0.339
2	12.909	MM	140	2.5	0.9443	1.007	0.603

(*S*,*Z*)-2-(2-(4-Methoxyphenyl)-4-phenyl-3-(phenylselanyl)pent-3-en-2-yl)-4,4,5,5-tetramethyl-1,3,2-dioxaborolane, 38

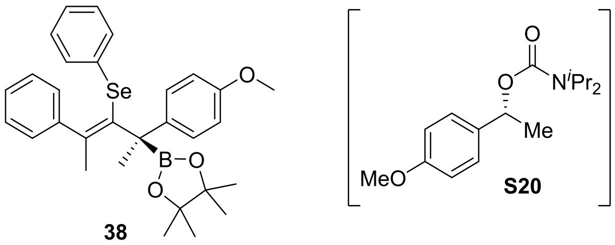

By analogy to a literature procedure,[13] a stirred solution of carbamate **S20**[17] (91 mg, 0.33 mmol, >99:1 e.r.) in diethyl ether (0.9 mL) was cooled to –78 °C and a solution of *sec*-butyllithium (1.3 M in cyclohexane/hexane, 0.230 mL, 0.3 mmol) was added dropwise. The resulting solution was stirred at

–78 °C for 15 minutes and then a solution of **S12** (100 mg, 0.25 mmol) in diethyl ether (2.4 mL) was added dropwise. The resulting solution was stirred at -78 °C for 1 hour and a solution of magnesium bromide (1 M in MeOH, 0.38 mL, 0.38 mmol) was added dropwise at the same temperature. The resulting solution was warmed to room temperature and then heated to 40 °C for 16 hours. The resulting white suspension was cooled to room temperature and diluted with diethyl ether and saturated aqueous sodium carbonate was added. The organic layer was separated and washed with saturated aqueous sodium carbonate and brine and then dried over anhydrous magnesium sulfate, filtered and concentrated under reduced pressure. Purification of the residue *via* column chromatography eluting with 97:3 pentane/diethyl ether afforded the title compound **38** as colourless oil (81 mg, 61 %, 99.0:1.0 e.r.).

^{1}H NMR (400 MHz $CDCl_3$) δ_H = 7.40 (d, *J* = 8.9 Hz, 2H), 6.97 – 6.77 (m, 10H), 6.78 (d, *J* = 8.9 Hz, 2H), 3.73 (s, 3H), 1.75 (s, 3H), 1.71 (s, 3H), 1.03 (s, 12H).

^{13}C NMR (101 MHz, $CDCl_3$) δ_C = 157.5, 146.1, 142.9, 138.9, 137.0, 134.9, 132.0, 128.9, 128.4, 128.1, 127.2, 125.7, 125.5, 113.3, 83.6, 55.2, 24.6, 24.5, 24.4, 20.8 [*N.B. The carbon attached to boron was not observed due to quadrupolar relaxation*];

^{11}B NMR (96 MHz, $CDCl_3$) δ_B = 32.6;

^{77}Se NMR (57 MHz, $CDCl_3$) δ_{Se} = 399.9;

FTIR (neat) ν/cm^{-1} = 3055, 2979, 2981, 1577, 1507, 1476, 1371, 1319, 1247, 1143, 1030, 851, 735;

HRMS (ESI$^+$): calculated $[M+Na]^+$ for $C_{30}H_{38}NaBO_3Se$ = 559.1899, mass found = 559.1744;

$[\alpha]_D^{23}$ = +80 (*c* = 0.50, $CHCl_3$);

Chiral HPLC (Chiralpak IB with guard, 100 % hexane, 1.0 mL/min, 25 °C, λ = 254 nm, 5 μL injection):

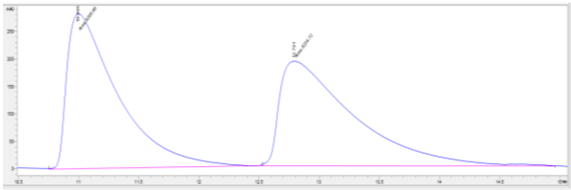

#	Time	Type	Area	Height	Width	Area%	Symmetry
1	10.995	MM	8266	282.5	0.4876	50.053	0.279
2	12.791	MM	8204.7	190.6	0.7175	49.682	0.242

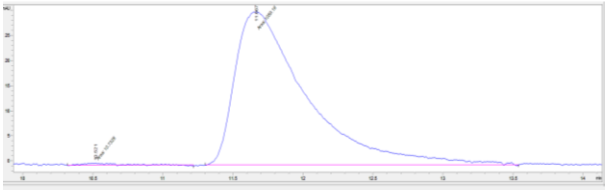

#	Time	Type	Area	Height	Width	Area%	Symmetry
1	10.521	MM	10.7	5.3E-1	0.3406	0.976	0.525
2	11.667	MM	1089.2	30.5	0.5956	99.024	0.407

4.3 *Enantiodivergent Synthesis of Allenes*

(*P*)-1-Methoxy-4-(5-phenylpenta-3,4-dien-1-yl)benzene, (*P*)-**4**

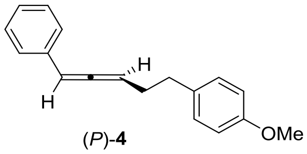

(*P*)-**4**

According to **General Procedure C** with **3** (80 mg, 0.15 mmol, 99.2:0.8 e.r.). Purification *via* column chromatography eluting with 99:1 pentane/diethyl ether afforded the title compound (*P*)-**4** as a colourless oil (33 mg, 88 %, 99.1:0.9 e.r.).

^{1}H NMR (400 MHz, $CDCl_3$) δ_H = 7.28 – 7.22 (m, 2H), 7.19 – 7.10 (m, 5H), 6.85 – 6.80 (m, 2H), 6.11 (dt, *J* = 6.2, 3.0 Hz, 1H), 5.57 (q, *J* = 6.6 Hz, 1H), 3.79 (s, 3H), 2.82 – 2.68 (m, 2H), 2.51 – 2.34 (m, 2H);

^{13}C NMR (101 MHz, $CDCl_3$) δ_C = 205.4, 158.0, 134.9, 133.7, 129.6, 128.6, 126.7, 126.7, 113.9, 94.9, 94.4, 55.3, 34.6, 30.9;

FTIR (neat) ν/cm^{-1} = 2932, 1948, 1611, 1511, 1459, 1300, 1245, 1177, 1037, 822, 778;

HRMS (ESI$^+$): calculated $[M+Na]^+$ for $C_{18}H_{18}NaO$ = 273.1250, mass found = 273.1241;

$[\alpha]_D^{23}$ = +228 (*c* = 0.50, $CHCl_3$);

Chiral HPLC (Chiralpak IB with guard, 0.2 % IPA, 99.8 % hexane, 1.0 mL/min, 25 °C, λ = 254 nm, 5 μL injection):

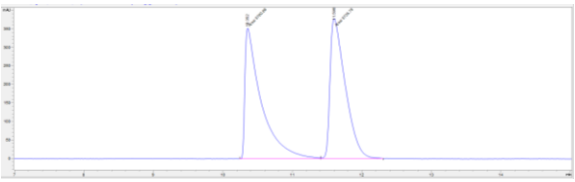

#	Time	Type	Area	Height	Width	Area%	Symmetry
1	10.352	MF	5760.7	352	0.2728	50.108	0.197
2	11.598	FM	5735.8	377.7	0.2531	49.892	0.383

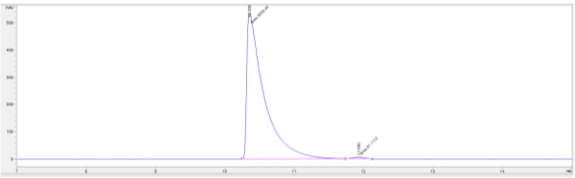

#	Time	Type	Area	Height	Width	Area%	Symmetry
1	10.35	MF	9254.3	536.7	0.2874	99.131	0
2	11.93	FM	81.1	6.8	0.1989	0.869	1.293

(*M*)-1-Methoxy-4-(5-phenylpenta-3,4-dien-1-yl)benzene, (*M*)-**4**

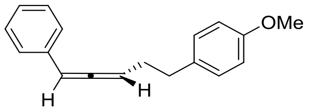

(*M*)-**4**

According to **General Procedure D** with **3** (80 mg, 0.15 mmol, 99.2:0.8 e.r.) and methyl triflate (34 μL, 0.30 mmol). Purification *via* column chromatography eluting with 99:1 pentane/diethyl ether afforded the title compound (*M*)-**4** as a colourless oil (31 mg, 83 %, 98.4:1.6 e.r.). The spectral data was identical to that described above.

$[\alpha]_D^{23}$ = –232 (c = 0.50, $CHCl_3$);

Chiral HPLC (Chiralpak IB with guard, 0.2 % IPA, 99.8 % hexane, 1.0 mL/min, 25 °C, λ = 254 nm, 5 μL injection):

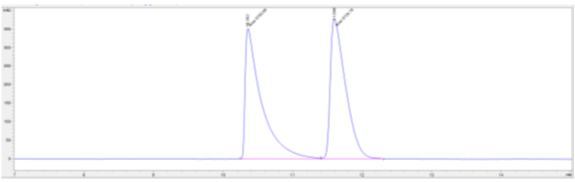

#	Time	Type	Area	Height	Width	Area%	Symmetry
1	10.352	MF	5760.7	352	0.2728	50.108	0.197
2	11.598	FM	5735.8	377.7	0.2531	49.892	0.383

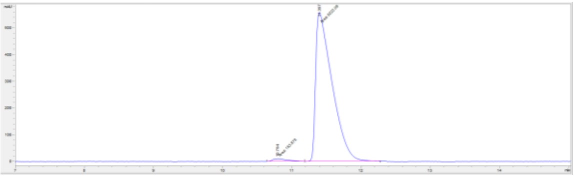

#	Time	Type	Area	Height	Width	Area%	Symmetry
1	10.794	MF	153.9	10.4	0.2471	1.641	0.487
2	11.397	FM	9222.1	559.5	0.2747	98.359	0.287

(*P*)-4,4'-(Penta-1,2-diene-1,5-diyl)bis(methoxybenzene), (*P*)-8

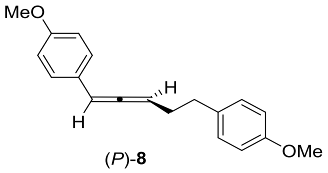

(*P*)-**8**

According to **General Procedure C** with **7** (85 mg, 0.15 mmol, 98.1:1.9 e.r.). Purification *via* column chromatography eluting with 98:2 pentane/diethyl ether afforded the title compound (*P*)-**8** as a white solid (32 mg, 76 %, 97.8:2.2 e.r.).

Melting point 36–37 ^{o}C (pentane/diethyl ether);

^{1}H NMR (400 MHz, $CDCl_3$) δ_H = 7.12 (d, *J* = 8.6 Hz, 2H), 7.06 (d, *J* = 8.7 Hz, 2H), 6.82 (d, *J* = 8.7 Hz, 2H), 6.80 (d, *J* = 8.8 Hz, 2H), 6.07 (dt, *J* = 6.2, 3.0 Hz, 1H), 5.53 (q, *J* = 6.6 Hz, 1H), 3.79 (s, 6H), 2.81 – 2.66 (m, 2H), 2.49 – 2.32 (m, 2H);

^{13}C NMR (101 MHz, $CDCl_3$) δ_C = 204.7, 158.7, 157.9, 133.8, 129.6, 127.8, 127.2, 114.1, 113.9, 94.4, 94.3, 55.4, 55.3, 34.6, 31.1;

FTIR (neat) ν/cm^{-1} = 2933, 1835, 1949, 1608, 1511, 1464, 1301, 1246, 1173, 1035, 834;

HRMS (ESI$^+$): calculated $[M+Na]^+$ for $C_{19}H_{20}NaO_2$ = 303.1356, mass found = 303.1356;

$[\alpha]_D^{22}$ = +232 (*c* = 0.50, $CHCl_3$);

Chiral HPLC (Chiralpak IA, 0.5 % IPA, 99.5 % hexane, 1.0 mL/min, 25 ^{o}C, λ = 254 nm, 5 μL injection):

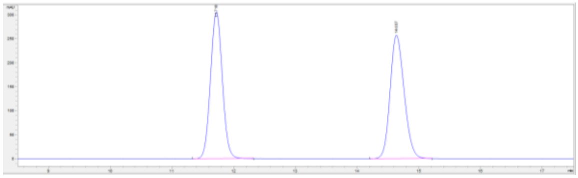

#	Time	Type	Area	Height	Width	Area%	Symmetry
1	11.718	BV R	4072.7	305.1	0.2083	49.992	0.953
2	14.637	BV R	4074	257.5	0.2391	50.008	0.798

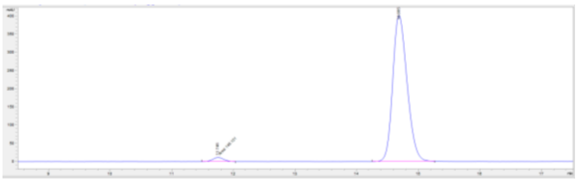

#	Time	Type	Area	Height	Width	Area%	Symmetry
1	11.745	MM	148.1	11.5	0.2153	2.225	0.826
2	14.685	BV R	6507.1	402	0.2298	97.775	0.74

(*M*)-4,4'-(Penta-1,2-diene-1,5-diyl)bis(methoxybenzene), (*M*)-**8**

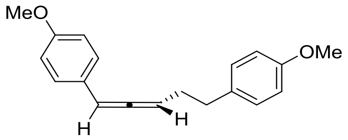

(*M*)-**8**

According to **General Procedure D** with **7** (85 mg, 0.15 mmol, 98.1:1.9 e.r.) and methyl triflate (34 μL, 0.30 mmol). Purification *via* column chromatography eluting with 98:2 pentane/diethyl ether afforded the title compound (*M*)-**8** as a colourless oil (35 mg, 83 %, 98.1:1.9 e.r.). The spectral data was identical to that described above.

$[\alpha]_D^{23}$ = –232 (c = 0.50, $CHCl_3$);

Chiral HPLC (Chiralpak IA with guard, 0.5 % IPA, 99.5 % hexane, 1.0 mL/min, 25 °C, λ = 254 nm, 5 μL injection):

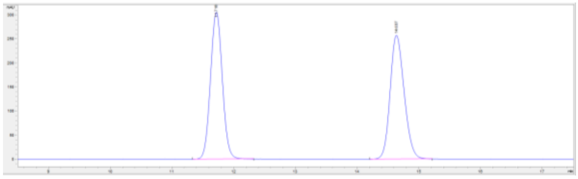

#	Time	Type	Area	Height	Width	Area%	Symmetry
1	11.718	BV R	4072.7	305.1	0.2083	49.992	0.953
2	14.637	BV R	4074	257.5	0.2391	50.008	0.798

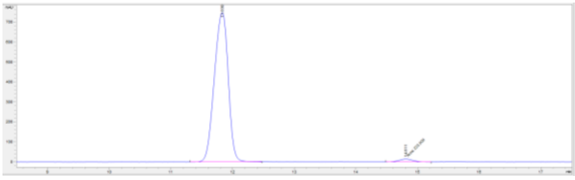

#	Time	Type	Area	Height	Width	Area%	Symmetry
1	11.83	VV R	11713.3	747.5	0.1883	98.124	1.33
2	14.811	MM	223.9	14.5	0.2578	1.876	1.023

(*P*)-1-Methoxy-4-(5-(4-(trifluoromethyl)phenyl)penta-3,4-dien-1-yl)benzene, (*P*)-**10**

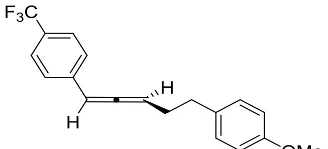

(*P*)-**10**

According to **General Procedure C** with **9** (100 mg, 0.167 mmol, 98.3:1.7 e.r.). Purification *via* column chromatography eluting with 98:2 pentane/diethyl ether afforded the title compound (*P*)-**10** as a colourless liquid (46 mg, 87 %, 98.5:1.5 e.r.).

^{1}H NMR (400 MHz, $CDCl_3$) δ_H = 7.39 (d, *J* = 7.7 Hz, 2H), 7.07 (d, *J* = 8.1 Hz, 2H), 7.03 (d, *J* = 8.6 Hz, 2H), 6.74 (d, *J* = 8.6 Hz, 2H), 6.03 (dt, *J* = 6.1, 2.9 Hz, 1H), 5.53 (q, *J* = 6.6 Hz, 1H), 3.70 (s, 3H), 2.87 – 2.57 (m, 2H), 2.51 – 2.25 (m, 2H);

^{13}C NMR (101 MHz, $CDCl_3$) δ_C = 206.3, 158.0, 138.8, 133.3, 129.5, 128.5 (q, *J* = 32.3 Hz), 126.7, 125.3 (q, *J* = 3.9 Hz), 124.3 (q, *J* = 271.7 Hz), 113.8, 94.8, 94.0, 55.2, 34.3, 30.5;

^{19}F NMR (377 MHz, $CDCl_3$) δ_F = –62.4;

FTIR (neat) ν/cm^{-1} = 2935, 1950, 1613, 1512, 1465, 1323, 1301, 1247, 1164, 1121, 1066, 826, 748;

HRMS (MALDI): calculated $[M+Na]^+$ for $C_{19}H_{17}F_3NaO$ = 341.1124, mass found = 341.1124;

$[\alpha]_D^{23}$ = +160 (*c* = 0.50, $CHCl_3$);

Chiral HPLC (Chiralpak IB with Guard, 0.2 % IPA, 99.8 % hexane, 1.0 mL/min, 25 °C, λ = 254 nm, 5 µL injection):

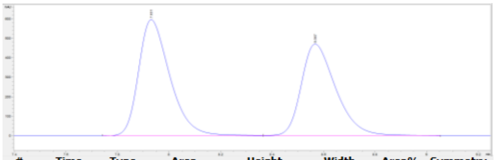

#	Time	Type	Area	Height	Width	Area%	Symmetry
1	7.931	BV	5022	594.3	0.1276	53.479	0.642
2	8.567	VB	4368.6	469	0.1417	46.521	0.638

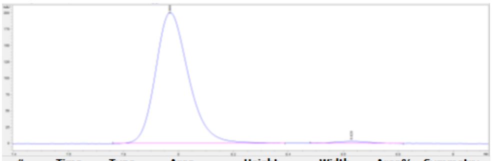

#	Time	Type	Area	Height	Width	Area%	Symmetry
1	7.969	BV R	1675.6	201.3	0.1276	98.488	0.763
2	8.629	VV R	25.7	3.1	0.1	1.512	0.912

(*M*)-1-Methoxy-4-(5-(4-(trifluoromethyl)phenyl)penta-3,4-dien-1-yl)benzene, (*M*)-**10**

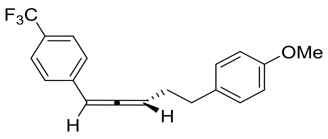

(*M*)-**10**

A solution of *β*-selenoboronic ester **9** (85 mg, 0.14 mmol, 98.3:1.7 e.r.) in dichloromethane (0.05 mL) was stirred in a 7 mL vial at room temperature and methyl triflate (32 µL, 0.28 mmol) was added in a single portion. The vial was tightly capped and was stirred at room temperature for 16 h. The

reaction mixture was then heated to 40 °C for 4 h. The reaction mixture was directly purified by preparative TLC (1% Et_2O in Pentane) to afford the title compound (*M*)-**10** as a colourless oil (25 mg, 56 %, 96.3:3.7 e.r.). The spectral data was identical to that described above.

$[\alpha]_D^{26}$ = –160 (*c* = 0.50, $CHCl_3$);

Chiral HPLC (Chiralpak IB with Guard, 0.2 % IPA, 99.8 % hexane, 1.0 mL/min, 25 °C, λ = 254 nm, 5 μL injection):

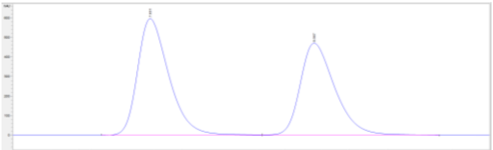

#	Time	Type	Area	Height	Width	Area%	Symmetry
1	7.931	BV	5022	594.3	0.1276	53.479	0.642
2	8.567	VB	4368.6	469	0.1417	46.521	0.638

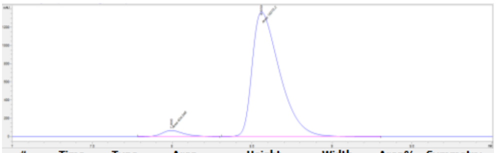

#	Time	Type	Area	Height	Width	Area%	Symmetry
1	7.995	MM	634.5	67.5	0.1566	3.730	0.741
2	8.559	MM	16375.2	1370.8	0.1991	96.270	0.503

(*P*)-1-(5-(4-Methoxyphenyl)penta-1,2-dien-1-yl)-2-methylbenzene, (*P*)-**12**

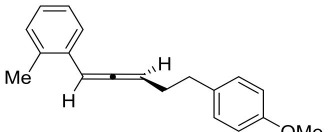

(*P*)-**12**

According to **General Procedure C** with **11** (80 mg, 0.15 mmol, 98.4:1.6 e.r.). Purification *via* column chromatography eluting with 98:2 pentane/diethyl ether afforded the title compound (*P*)-**12** as a colourless liquid (34 mg, 88 %, 98.1:1.9 e.r.).

^{1}H NMR (400 MHz, $CDCl_3$) δ_H = 7.11 – 7.09 (m, 1H), 7.06 – 6.99 (m, 5H), 6.75 (d, *J* = 8.6 Hz, 2H), 6.22 (dt, *J* = 6.2, 3.0 Hz, 1H), 5.46 (q, *J* = 6.6 Hz, 1H), 3.72 (s, 3H), 2.71 – 2.65 (m, 2H), 2.39 – 2.31 (m, 2H), 2.26 (s, 3H);

^{13}C NMR (101 MHz, $CDCl_3$) δ_C = 206.0, 157.9, 134.8, 133.7, 133.0, 130.4, 129.5, 127.1, 126.6, 126.0, 113.8, 93.4, 92.1, 55.3, 34.5, 30.9, 19.8;

FTIR (neat) ν/cm^{-1} = 2933, 1949, 1611, 1572, 1456, 1300, 1245, 1177, 1035, 829, 742;

HRMS (ESI$^+$): calculated $[M+H]^+$ for $C_{19}H_{21}O$ = 265.1587, mass found = 265.1599;

$[\alpha]_D^{24}$ = +160 (*c* = 0.50, $CHCl_3$);

Chiral HPLC (2 x Chiralpak ADH, 0.2 % IPA, 99.8 % hexane, 1.0 mL/min, 25 ^oC, λ = 254 nm, 5 µL injection):

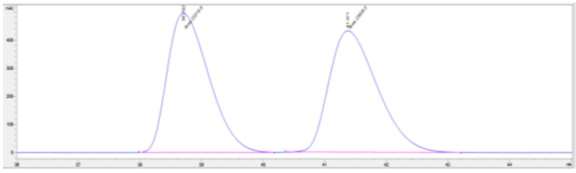

#	Time	Type	Area	Height	Width	Area%	Symmetry
1	38.702	MM	23316.9	498.2	0.7801	49.688	0.628
2	41.371	MM	23609.5	434.2	0.9063	50.312	0.633

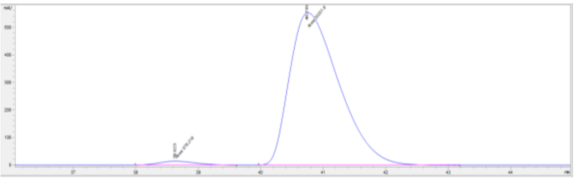

#	Time	Type	Area	Height	Width	Area%	Symmetry
1	38.623	MM	578.2	14.3	0.6727	1.879	0.76
2	40.735	MM	30201.8	552.2	0.9115	98.121	0.541

(*M*)-1-(5-(4-Methoxyphenyl)penta-1,2-dien-1-yl)-2-methylbenzene, (*M*)-**12**

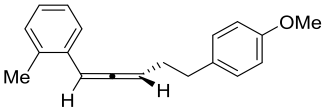

(*M*)-**12**

According to **General Procedure D** with **11** (84 mg, 0.15 mmol, 98.4:1.6 e.r.) and methyl triflate (34 µL, 0.30 mmol). Purification *via* column chromatography eluting with 98:2 pentane/diethyl ether afforded the title compound (*M*)-**12** as a colourless oil (36 mg, 88 %, 98.1:1.9 e.r.). The spectral data was identical to that described above.

$[\alpha]_D^{23}$ = –144 (*c* = 0.50, $CHCl_3$);

Chiral HPLC (2 x Chiralpak ADH, 0.2 % IPA, 99.8 % hexane, 1.0 mL/min, 25 °C, λ = 254 nm, 5 μL injection):

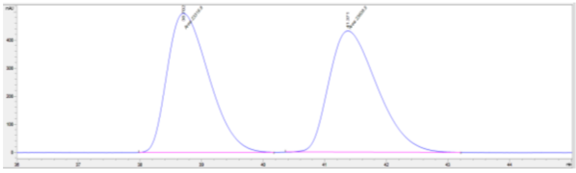

#	Time	Type	Area	Height	Width	Area%	Symmetry
1	38.702	MM	23316.9	498.2	0.7801	49.688	0.628
2	41.371	MM	23609.5	434.2	0.9063	50.312	0.633

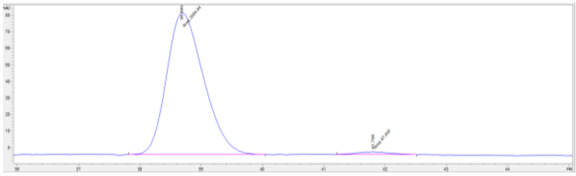

#	Time	Type	Area	Height	Width	Area%	Symmetry
1	38.685	MM	3584.9	85.8	0.6965	98.148	0.703
2	41.798	MM	67.6	1.8	0.6266	1.852	0.888

(*P*)-1-Methoxy-4-(octa-3,4-dien-1-yl)benzene, (*P*)-**14**

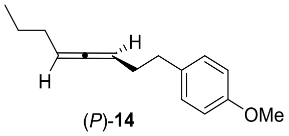

(*P*)-**14**

According to **General Procedure C** with **13** (150 mg, 0.30 mmol, 98.3:1.7 e.r.). Purification *via* column chromatography eluting with 98:2 pentane/diethyl ether afforded the title compound (*P*)-**14** as a white solid (50 mg, 77 %, 98.2:1.8 e.r.).

^{1}H NMR (400 MHz $CDCl_3$) δ_H = 7.03 (d, *J* = 8.7 Hz, 2H), 6.75 (d, *J* = 8.7 Hz, 2H), 5.06 – 4.97 (m, 2H), 3.70 (s, 3H), 2.58 (t, *J* = 7.7 Hz, 2H), 2.22 – 2.16 (m, 2H), 1.87 – 1.82 (m, 2H), 1.31 (sext, *J* = 7.3 Hz, 2H), 0.83 (t, *J* = 7.4 Hz, 3H);

^{13}C NMR (101 MHz, $CDCl_3$) δ_C = 204.2, 157.9, 134.2, 129.5, 113.8, 91.3, 90.3, 55.4, 34.8, 31.2, 31.2, 22.5, 13.8;

FTIR (neat) ν/cm^{-1} = 2956, 2930, 1961, 1611, 1511, 1463, 1300, 1243, 1176, 1037, 820, 740;

HRMS (ESI$^+$): calculated $[M+H]^+$ for $C_{15}H_{21}O$ = 217.1587, mass found = 217.1592;

$[\alpha]_D^{22}$ = +50 (*c* = 0.50, $CHCl_3$);

Chiral HPLC (Chiralpak AS, 2 % IPA, 98 % hexane, 1.0 mL/min, 25 °C, λ = 254 nm, 5 μL injection):

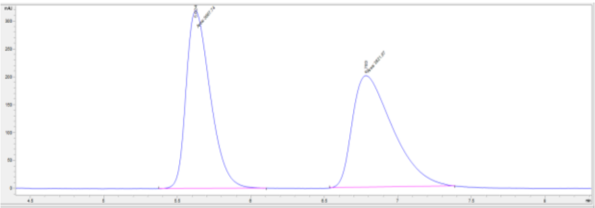

#	Time	Type	Area	Height	Width	Area%	Symmetry
1	5.624	MM	3667.7	320.1	0.1909	48.970	0.625
2	6.783	MM	3822	200.5	0.3178	51.030	0.489

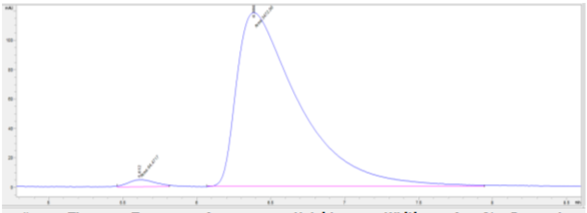

#	Time	Type	Area	Height	Width	Area%	Symmetry
1	5.612	MM	64.5	4.9	0.2171	1.823	0.699
2	6.386	MM	3472.6	117.9	0.4911	98.177	0.364

(*M*)-1-Methoxy-4-(octa-3,4-dien-1-yl)benzene, (*M*)-**14**

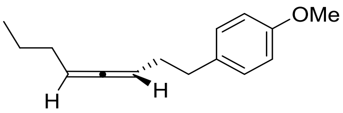

(*M*)-**14**

According to **General Procedure D** with **13** (140 mg, 0.28 mmol, 98.3:1.7 e.r.) and methyl triflate (63 μL, 0.56 mmol). Purification *via* column chromatography eluting with 98:2 pentane/diethyl ether afforded the title compound (*M*)-**14** as a colourless oil (50 mg, 82 %, 98.2:1.8 e.r.). The spectral data was identical to that described above.

$[\alpha]_D^{22}$ = –50 (c = 0.50, $CHCl_3$).

Chiral HPLC (Chiralpak AS, 2 % IPA, 98 % hexane, 1.0 mL/min, 25 °C, λ = 254 nm, 5 μL injection):

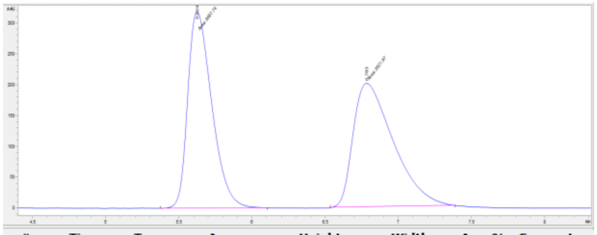

#	Time	Type	Area	Height	Width	Area%	Symmetry
1	5.624	MM	3667.7	320.1	0.1909	48.970	0.625
2	6.783	MM	3822	200.5	0.3178	51.030	0.489

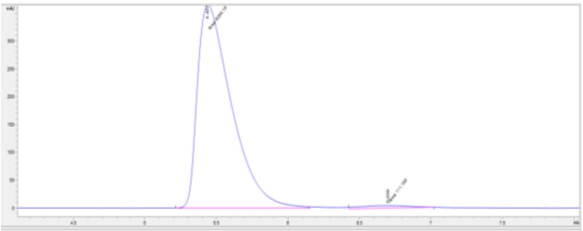

#	Time	Type	Area	Height	Width	Area%	Symmetry
1	5.437	MM	6084.2	366.9	0.2764	98.206	0.42
2	6.698	MM	111.2	4.6	0.4041	1.794	1.431

(*P*)-*tert*-Butyldimethyl((5-phenylpenta-3,4-dien-1-yl)oxy)silane, (*P*)-**16**

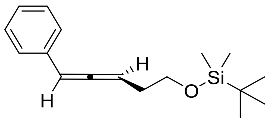

(*P*)-**16**

According to **General Procedure C** with **15** (83 mg, 0.15 mmol, 98.9:1.1 e.r.). Purification *via* column chromatography eluting with 98:2 pentane/diethyl ether afforded the title compound (*P*)-**16** as a colourless liquid (30 mg, 74 %, 98.2:1.8 e.r.).

^{1}H NMR (400 MHz, $CDCl_3$) δ_H = 7.23 – 7.21 (m, 4H), 7.14 – 7.09 (m, 1H), 6.05 (dt, *J* = 6.6, 2.8 Hz, 1H), 5.51 (q, *J* = 6.9 Hz, 1H), 3.68 (t, *J* = 6.6 Hz, 2H), 2.28 (qd, *J* = 6.8, 2.8 Hz, 2H), 0.84 (s, 9H), 0.00 (s, 6H);

^{13}C NMR (101 MHz, $CDCl_3$) δ_C = 205.7, 134.9, 128.5, 126.7, 126.7, 94.5, 91.8, 62.8, 32.5, 26.0, 18.4, -5.2;

FTIR (neat) ν/cm^{-1} = 2928, 1949, 1598, 1495, 1459, 1275, 1260, 1098, 1047, 876, 764, 750;

HRMS (ESI$^+$): calculated [M+Na]$^+$ for $C_{17}H_{26}NaOSi$ = 297.1645, mass found = 297.1641;

$[\alpha]_D^{23}$ = +92 (*c* = 1.00, $CHCl_3$);

Chiral HPLC (Chiralpak IB with Guard, 0.15 % IPA, 99.85 % hexane, 0.3 mL/min, 25 °C, λ = 254 nm, 5 μL injection):

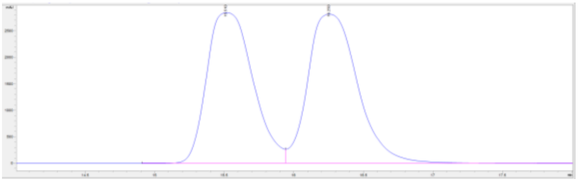

#	Time	Type	Area	Height	Width	Area%	Symmetry
1	15.51	BV	65255.3	2838.9	0.2729	48.343	0.694
2	16.25	VB	69729.7	2811.3	0.2941	51.657	0.708

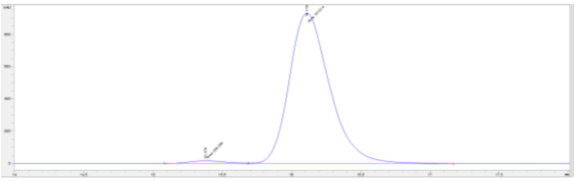

#	Time	Type	Area	Height	Width	Area%	Symmetry
1	15.379	MF	356.4	18.6	0.3189	1.830	0.798
2	16.11	FM	19123.4	933.1	0.3416	98.170	0.754

(*M*)-5-(4-Methoxyphenyl)penta-3,4-dien-1-ol, (*M*)-**17**

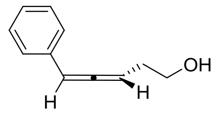

(*M*)-**17**

According to **General Procedure D** with **15** (83.4 mg, 0.15 mmol, 98.9:1.1 e.r.) and methyl triflate (34 µL, 0.30 mmol). Purification *via* column chromatography eluting with 75:25 pentane/diethyl ether afforded the title compound (*M*)-**17** as a colourless oil (18 mg, 76 %, 98.4:1.6 e.r.). The spectral data matched that previously reported in the literature.[18]

^{1}H NMR (400 MHz, $CDCl_3$) δ_H = 7.23 – 7.21 (m, 4H), 7.15 – 7.10 (m, 1H), 6.14 – 6.09 (m, 1H), 5.53 (q, *J* = 6.6 Hz, 1H), 3.71 (t, *J* = 6.2 Hz, 2H), 2.33 (tdd, *J* = 6.2, 5.6, 2.7 Hz, 2H), 1.50 (br s, 1H, O*H*);

^{13}C NMR (101 MHz, $CDCl_3$) δ_C = 205.7, 134.4, 128.7, 127.0, 126.7, 95.2, 91.5, 62.0, 32.1.;

FTIR (neat) ν/cm^{-1} = 3307, 2957, 1942, 1643, 1497, 1451, 1275, 1261, 1114, 1015, 764, 749;

HRMS (ESI$^+$): calculated $[M+Na]^+$ for $C_{11}H_{12}NaO$ = 183.0780, mass found = 183.0780;

$[\alpha]_D^{24}$ = –234 (*c* = 0.59, $CHCl_3$), lit. $[\alpha]_D^{25}$ = –219.7 (*c* = 0.59, $CHCl_3$);[19]

Chiral HPLC (Chiralpak IB with Guard, 1 % IPA, 99 % hexane, 1.0 mL/min, 25 °C, λ = 254 nm, 5 µL injection):

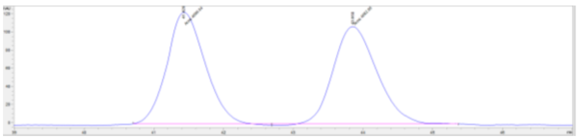

#	Time	Type	Area	Height	Width	Area%	Symmetry
1	41.426	MF	4699.5	123.3	0.6354	50.088	0
2	43.85	FM	4683	107.6	0.7255	49.912	0.784

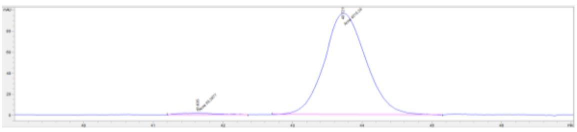

#	Time	Type	Area	Height	Width	Area%	Symmetry
1	41.635	MM	63.3	1.7	0.6057	1.552	0.911
2	43.721	MM	4015.2	96.8	0.6916	98.448	0.831

(*M*)-*tert*-Butyldimethyl((5-phenylpenta-3,4-dien-1-yl)oxy)silane, (*M*)-**16**

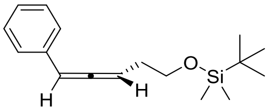

(*M*)-**16**

TBS protection of (*M*)-**17** was carried out in order to verify that (*M*)-**17** is in the opposite enantiomeric series to (*P*)-**16**.

To a stirred solution of (*M*)-**17** (14 mg, 0.089 mmol, 98.4:1.6 e.r.) in dichloromethane (1 mL) at room temperature, tert-butyldimethylsilyl chloride (28 mg, 0.19 mmol) and imidazole (36 mg, 0.54 mmol) were added. The reaction mixture was stirred at room temperature for 22 hrs and then the solvent was removed under reduced pressure. Purification of the residue *via* column chromatography eluting with 98:2 pentane/diethyl ether afforded the title compound (*M*)-**16** as a colourless oil (19 mg, 78%, 98.1:1.9 e.r.). The spectral data was identical to that described above for (*P*)-**16**.

$[\alpha]_D^{24}$ = −112 (c = 1.00, $CHCl_3$);

Chiral HPLC (Chiralpak IB with Guard, 0.15 % IPA, 99.85 % hexane, 0.3 mL/min, 25 °C, λ = 254 nm, 5 μL injection):

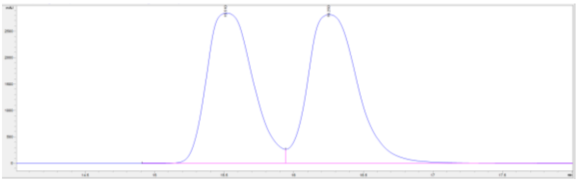

#	Time	Type	Area	Height	Width	Area%	Symmetry
1	15.51	BV	65255.3	2838.9	0.2729	48.343	0.694
2	16.25	VB	69729.7	2811.3	0.2941	51.657	0.708

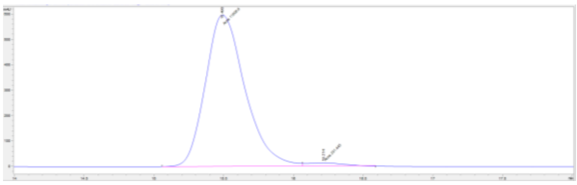

#	Time	Type	Area	Height	Width	Area%	Symmetry
1	15.49	MF	11806.9	596.6	0.3298	98.077	0.746
2	16.214	FM	231.4	12.6	0.3058	1.923	0.825

((2*P*,5*S*)-5,9-Dimethyldeca-1,2,8-trien-1-yl)benzene, (*P*,*S*)-**19**

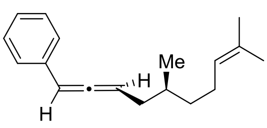

(*P*,*S*)-**19**

According to **General Procedure C** with **18** (100 mg, 0.19 mmol, >95:5 d.r.). Purification *via* column chromatography eluting with a gradient from pentane to 98:2 pentane/diethyl ether afforded the title compound (*P*,*S*)-**19** as a colourless oil (39 mg, 85 %, >95:5 d.r.).

^{1}H NMR (400 MHz CDCl$_3$) δ_H = 7.29 – 7.17 (m, 4H), 7.20 – 7.15 (m, 1H), 6.10 (dt, *J* = 6.4, 2.6 Hz, 1H), 5.51 (q, *J* = 7.0 Hz, 1H), 5.11 – 5.06 (m, 1H), 2.20 – 2.12 (m, 1H), 2.07 – 1.91 (m, 3H), 1.68 – 1.59 (m, 7H), 1.48 – 1.40 (m, 1H), 1.27 – 1.18 (m, 1H), 0.98 (d, *J* = 6.7 Hz, 3H);

^{13}C NMR (101 MHz, CDCl$_3$) δ_C = 205.8, 135.3, 131.4, 128.7, 126.8, 126.7, 124.9, 94.1, 93.5, 36.7, 36.4, 32.9, 25.9, 25.7, 19.7, 17.8;

FTIR (neat) ν/cm^{-1} = 3031, 2961, 2912, 1949, 1598, 1495, 1457, 1376, 1263, 1111, 1070, 874, 770;

HRMS (ESI$^+$): calculated [M+H]$^+$ for $C_{18}H_{25}$ = 241.1951, mass found = 241.1949;

$[\alpha]_D^{23}$ = +218 (*c* = 0.50, CHCl$_3$);

((2*M*,5*S*)-5,9-Dimethyldeca-1,2,8-trien-1-yl)benzene, (*M*,*S*)-**20**

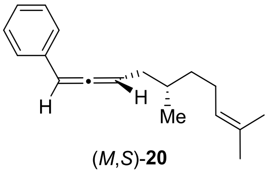

(*M*,*S*)-**20**

According to **General Procedure D** with **18** (100 mg, 0.19 mmol, >95:5 d.r.) and methyl triflate (43 µL, 0.38 mmol). Purification *via* column chromatography eluting with 98:2 pentane/diethyl ether afforded the title compound (*M*,*S*)-**20** as a colourless oil (38 mg, 83 %, >95:5 d.r.).

^{1}H NMR (400 MHz CDCl$_3$) δ_H = 7.28 – 7.27 (m, 4H), 7.19 – 7.14 (m, 1H), 6.09 (dt, *J* = 6.5, 2.7 Hz, 1H), 5.50 (q, *J* = 7.2 Hz, 1H), 5.12 – 5.07 (m, 1H), 2.18 – 2.11 (m, 1H), 2.04 – 1.94 (m, 3H), 1.67 – 1.58 (m, 7H), 1.48 – 1.39 (m, 1H), 1.26 – 1.17 (m, 1H), 0.95 (d, *J* = 6.7 Hz, 3H);

^{13}C NMR (101 MHz, CDCl$_3$) δ_C = 205.7, 135.1, 131.2, 128.5, 126.6, 126.6, 124.7, 93.9, 93.3, 36.6, 36.2, 32.8, 25.7, 25.6, 19.5, 17.7;

FTIR (neat) ν/cm^{-1} = 3029, 2957, 2890, 1945, 1577, 1483, 1456, 1314, 1201, 1025, 840, 680;

HRMS (ESI$^+$): calculated for [M+H]$^+$ $C_{18}H_{25}$ = 241.1951, mass found = 241.1945;

$[\alpha]_D^{23}$ = –220 (*c* = 0.50, CHCl$_3$).

***tert*-Butyl(((6*P*,4*S*,2*R*)-2,4-dimethyl-7-phenylhepta-5,6-dien-1-yl)oxy)diphenylsilane,** (*P*,*S*,*R*)-**22**

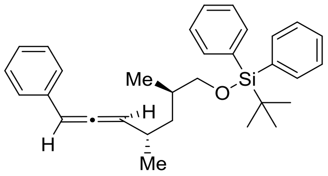

(*P*,*S*,*R*)-**22**

According to **General Procedure C** with **21** (111 mg, 0.15 mmol, >95:5 d.r.). Purification *via* column chromatography eluting with a gradient from pentane to 99:1 pentane/diethyl ether afforded the title compound (*P*,*S*,*R*)-**22** as a colourless oil (61 mg, 89 %, >95:5 d.r.).

^{1}H NMR (400 MHz, CDCl$_3$) δ_H = 7.70 – 7.65 (m, 4H), 7.44 – 7.34 (m, 6H), 7.28 (d, *J* = 4.4 Hz, 4H), 7.20 – 7.14 (m, 1H), 6.13 (dd, *J* = 6.5, 2.2 Hz, 1H), 5.44 (t, *J* = 6.8 Hz, 1H), 3.53 (dd, *J* = 9.8, 5.7 Hz, 1H), 3.47 (dd, *J* = 9.8, 6.4 Hz, 1H), 2.43 – 2.31 (m, 1H), 1.96 – 1.83 (m, 1H), 1.54 (ddd, *J* = 13.8, 9.1, 5.1 Hz, 1H), 1.12 (ddd, *J* = 13.8, 8.8, 5.7 Hz, 1H), 1.08 – 1.05 (m, 12H), 0.93 (d, *J* = 6.6 Hz, 3H);

^{13}C NMR (101 MHz, CDCl$_3$) δ_C = 204.1, 135.7, 135.3, 134.2, 134.1, 129.6, 128.6, 127.7, 126.7, 126.6, 100.7, 95.3, 69.3, 40.9, 33.8, 31.7, 27.0, 21.7, 19.4, 16.9;

FTIR (neat) ν/cm^{-1} = 2958, 2929, 2857, 1947, 1427, 1112, 824, 781, 739, 701, 691;

HRMS (ESI$^+$): calculated $[M+Na]^+$ for $C_{31}H_{38}NaOSi$ = 477.2584, mass found = 477.2564;

$[\alpha]_D^{24}$ = +124 (c = 0.50, $CHCl_3$).

***tert*-Butyl(((6*M*,4*S*,2*R*)-2,4-dimethyl-7-phenylhepta-5,6-dien-1-yl)oxy)diphenylsilane,** (*M*,*S*,*R*)-**23**

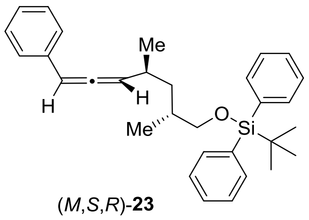

(*M*,*S*,*R*)-**23**

According to **General Procedure D** with **21** (111 mg, 0.15 mmol, >95:5 d.r.) and methyl triflate (34 µL, 0.30 mmol). Purification *via* column chromatography eluting with a gradient from pentane to 99:1 pentane/diethyl ether afforded the title compound (*M*,*S*,*R*)-**23** as a colourless oil (60 mg, 88 %, >95:5 d.r.).

^{1}H NMR (400 MHz, $CDCl_3$) δ_H = 7.69 – 7.64 (m, 4H), 7.44 – 7.34 (m, 6H), 7.30 – 7.26 (m, 4H), 7.21 – 7.14 (m, 1H), 6.14 (dd, *J* = 6.3, 2.3 Hz, 1H), 5.46 (t, *J* = 6.8 Hz, 1H), 3.51 (dd, *J* = 9.8, 5.7 Hz, 1H), 3.45 (dd, *J* = 9.8, 6.3 Hz, 1H), 2.43 – 2.31 (m, 1H), 1.90 – 1.78 (m, 1H), 1.56 (ddd, *J* = 14.0, 8.9, 5.3 Hz, 1H), 1.15 (ddd, *J* = 14.0, 8.6, 5.8 Hz, 1H), 1.08 (d, *J* = 6.7 Hz, 3H), 1.06 (s, 9H), 0.95 (d, *J* = 6.7 Hz, 3H);

^{13}C NMR (101 MHz, $CDCl_3$) δ_C = 204.1, 135.7, 135.2, 134.1, 134.1, 129.6, 128.6, 127.7, 126.7, 126.6, 100.9, 95.2, 69.2, 41.3, 33.7, 31.7, 27.0, 21.7, 19.4, 17.0;

FTIR (neat) ν/cm^{-1} = 2958, 2929, 2857, 1948, 1428, 1111, 824, 780, 739, 701, 691;

HRMS (ESI$^+$): calculated $[M+Na]^+$ for $C_{31}H_{38}NaOSi$ = 477.2584, mass found = 477.2562;

$[\alpha]_D^{24}$ = –106 (c = 0.50, $CHCl_3$).

(*M*)-Buta-1,2-diene-1,3-diyldibenzene, (*M*)-**25**

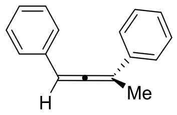

(*M*)-**25**

According to **General Procedure C** with **24** (73 mg, 0.15 mmol, >99:1 e.r.). Purification *via* column chromatography eluting with pentane afforded the title compound (*M*)-**25** as a colourless oil (27 mg, 87 %, 98.6:1.4 e.r.). The spectral data matched that previously reported in the literature.[20]

^{1}H NMR (400 MHz, $CDCl_3$) δ_H = 7.49 – 7.44 (m, 2H), 7.36 – 7.28 (m, 6H), 7.26 – 7.18 (m, 2H), 6.48 (q, *J* = 2.9 Hz, 1H), 2.23 (d, *J* = 2.9 Hz, 3H);

^{13}C NMR (101 MHz, $CDCl_3$) δ_C = 206.9, 136.4, 134.6, 128.8, 128.5, 127.1, 127.1, 127.0, 125.9, 104.6, 96.7, 16.8;

FTIR (neat) ν/cm^{-1} = 3060, 3027, 1934, 1597, 1493, 1448, 1067, 1027, 911, 824, 762, 744;

LRMS (EI$^+$): calculated [M]$^+$ for $C_{16}H_{14}$ = 206, mass found = 206;

$[\alpha]_D^{20}$ = –650 (*c* = 0.10, $CHCl_3$), lit. $[\alpha]_D^{20}$ = –758 (*c* = 0.10, $CHCl_3$)[20];

Chiral HPLC (Chiralpak IB with guard, 0 % IPA, 100 % hexane, 1.0 mL/min, 25 °C, λ = 254 nm, 5 µL injection):

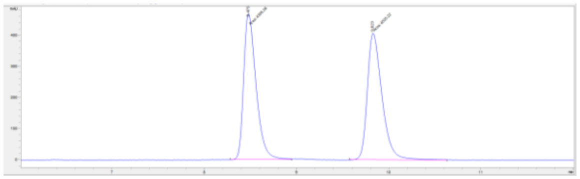

#	Time	Type	Area	Height	Width	Area%	Symmetry
1	8.475	MF	4395.1	468.1	0.1565	49.381	0.603
2	9.823	MM	4505.2	406.4	0.1848	50.619	0.584

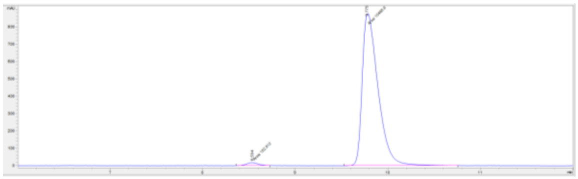

#	Time	Type	Area	Height	Width	Area%	Symmetry
1	8.534	MM	152.9	17.3	0.1473	1.440	0.906
2	9.775	MM	10468.9	875.5	0.1993	98.560	0.443

(*P*)-Buta-1,2-diene-1,3-diyldibenzene, (*P*)-**25**

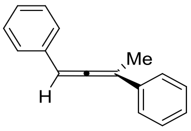

(*P*)-**25**

According to **General Procedure D** with **24** (73 mg, 0.15 mmol, >99:1 e.r.) and methyl triflate (34 µL, 0.30 mmol). Purification *via* column chromatography eluting with pentane afforded an inseparable mixture of the title compound (*P*)-**25** along with methyl(phenyl)selane. The residue was dissolved in THF (1.5 mL) and stirred at room temperature. Aqueous hydrogen peroxide (30 % w/v, 0.20 mL) was added and the resulting solution was stirred at room temperature for 1 h and then diluted with diethyl ether and water. The organic phase was separated and the aqueous phase was extracted twice with diethyl ether. The combined organic extracts were washed with brine, dried over anhydrous magnesium sulfate, filtered and concentrated under reduced pressure. Purification of the residue *via* column chromatography eluting with pentane afforded the title compound (*P*)-**25** as a colourless oil (26 mg, 84 %, 99.0:1.0 e.r.) The spectral data was identical to that described above.

$[\alpha]_D^{20}$ = +610 (c = 0.10, $CHCl_3$);

Chiral HPLC (Chiralpak IB with guard, 0 % IPA, 100 % hexane, 1.0 mL/min, 25 °C, λ = 254 nm, 5 µL injection):

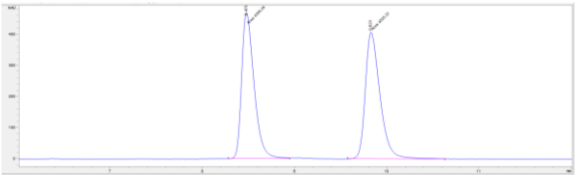

#	Time	Type	Area	Height	Width	Area%	Symmetry
1	8.475	MF	4395.1	468.1	0.1565	49.381	0.603
2	9.823	MM	4505.2	406.4	0.1848	50.619	0.584

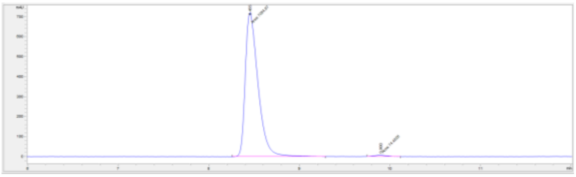

#	Time	Type	Area	Height	Width	Area%	Symmetry
1	8.455	MM	7084.7	722	0.1635	98.960	0.58
2	9.9	MM	74.5	6.9	0.1796	1.040	1.004

(*M*)-1-(2-Phenylvinylidene)-1,2,3,4-tetrahydronaphthalene, (*M*)-**27**

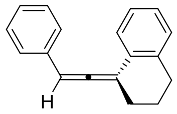

(*M*)-**27**

According to **General Procedure C** with **26** (97 mg, 0.19 mmol, 98.0:2.0 e.r.). Purification *via* column chromatography eluting with pentane afforded the title compound (*M*)-**27** contaminated with 5 mol % diphenyldiselenide as a colourless oil (35 mg, 76 %, 97.2:2.8 e.r.). The spectral data matched that previously reported in the literature.[20]

^{1}H NMR (500 MHz, $CDCl_3$)* δ_H = 7.51 (d, *J* = 6.8 Hz, 1H), 7.38 – 7.31 (m, 4H), 7.23 (t, *J* = 7.1 Hz, 1H), 7.20 – 7.11 (m, 3H), 6.55 (t, *J* = 3.1 Hz, 1H), 2.90 (t, *J* = 6.2 Hz, 2H), 2.78 – 2.66 (m, 2H), 2.07 – 1.96 (m, 2H);

^{13}C NMR (126 MHz, $CDCl_3$) δ_C = 204.4, 136.7, 134.7, 130.8, 129.4, 128.7, 127.1, 127.0, 126.9, 126.9, 126.2, 105.8, 97.5, 30.1, 28.7, 23.1;

FTIR (neat) ν/cm^{-1} = 3027, 2931, 1935, 1598, 1488, 1451, 1283, 1261, 823, 760, 744, 693;

LRMS (EI$^+$): calculated $[M]^+$ for $C_{18}H_{16}$ = 232, mass found = 232;

$[\alpha]_D^{24}$ = –43 (*c* = 0.10, $CHCl_3$), lit. $[\alpha]_D^{20}$ = –109 (*c* = 0.10, $CHCl_3$)[20];

* **27** is somewhat unstable in solution in chloroform. $CDCl_3$ was neutralised over K_2CO_3 and NMR spectra were recorded as rapidly as possible to minimise decomposition.

Chiral HPLC (Chiralpak IB with guard, 0 % IPA, 100 % hexane, 1.0 mL/min, 25 °C, λ = 254 nm, 5 μL injection):

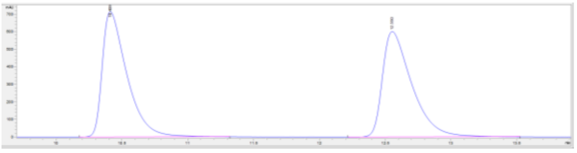

#	Time	Type	Area	Height	Width	Area%	Symmetry
1	10.409	BV R	9292.2	714.7	0.1812	49.923	0.447
2	12.55	VV R	9320.8	601	0.2219	50.077	0.468

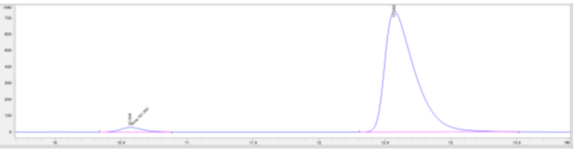

#	Time	Type	Area	Height	Width	Area%	Symmetry
1	10.569	MM	342	28.3	0.2016	2.844	0.794
2	12.568	BV R	11682.4	740.8	0.206	97.156	0.425

(*P*)-1-(2-Phenylvinylidene)-1,2,3,4-tetrahydronaphthalene, (*P*)-**27**

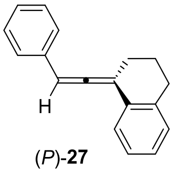

(*P*)-**27**

According to **General Procedure D** with **26** (77 mg, 0.15 mmol, 98.0:2.0 e.r.) and methyl triflate (85 μL, 0.75 mmol). Purification *via* column chromatography eluting with pentane afforded the title compound (*P*)-**27** as a colourless oil (32 mg, 92 %, 98.3:1.7 e.r.) The spectral data was identical to that described above.

$[\alpha]_D^{24}$ = +45 (c = 0.10, $CHCl_3$);

Chiral HPLC (Chiralpak IB with guard, 0 % IPA, 100 % hexane, 1.0 mL/min, 25 °C, λ = 254 nm, 5 μL injection):

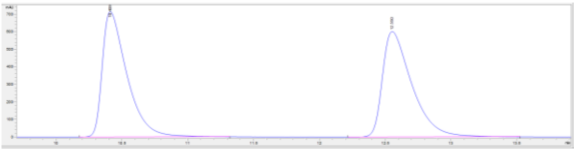

#	Time	Type	Area	Height	Width	Area%	Symmetry
1	10.409	BV R	9292.2	714.7	0.1812	49.923	0.447
2	12.55	VV R	9320.8	601	0.2219	50.077	0.468

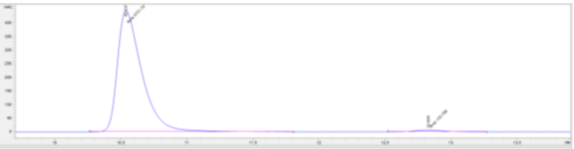

#	Time	Type	Area	Height	Width	Area%	Symmetry
1	10.537	MM	5731.8	444.1	0.2151	98.272	0.518
2	12.828	MM	100.8	6.5	0.2588	1.728	0.943

(*M*)-(3-Methylpenta-1,2-diene-1,5-diyl)dibenzene, (*M*)-**29**

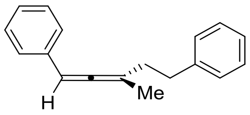

(*M*)-**29**

According to **General Procedure C** with **28** (89 mg, 0.17 mmol, >99:1 e.r.). Purification *via* column chromatography eluting with hexane afforded the title compound (*M*)-**29** as a colourless oil (34 mg, 84 %, 99.2:0.8 e.r.). The spectral data matched that previously reported in the literature.[21]

^{1}H NMR (500 MHz, $CDCl_3$) δ_H = 7.30 – 7.25 (m, 4H), 7.23 – 7.15 (m, 6H), 6.08 (sext, *J* = 2.9 Hz, 1H), 2.87 – 2.77 (m, 2H), 2.48 – 2.37 (m, 2H), 1.86 (d, *J* = 2.9 Hz, 3H);

^{13}C NMR (121 MHz, $CDCl_3$) δ_C = 202.8, 141.9, 135.8, 128.4, 128.4, 128.3, 126.6, 126.4, 125.8, 103.1, 94.4, 35.8, 33.8, 18.9;

FTIR (neat) ν/cm^{-1} = 3026, 2915, 1950, 1598, 1496, 1453, 1073, 1029, 910, 818, 741;

LRMS (EI$^+$): calculated [M]$^+$ for $C_{18}H_{18}$ = 234, mass found = 234;

$[\alpha]_D^{24}$ = –216 (*c* = 0.50, $CHCl_3$);

Chiral HPLC (2 x Chiralpak IB, 0 % IPA, 100 % hexane, 0.5 mL/min, 25 °C, λ = 254 nm, 5 μL injection):

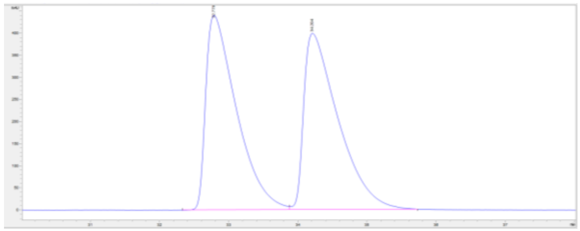

#	Time	Type	Area	Height	Width	Area%	Symmetry
1	32.779	BV	13612.2	439.6	0.3627	49.708	0.345
2	34.204	VV R	13771.8	398	0.4052	50.292	0.323

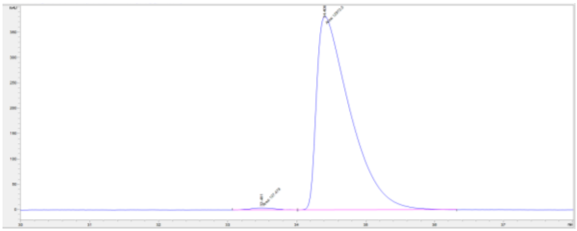

#	Time	Type	Area	Height	Width	Area%	Symmetry
1	33.491	MF	107.5	4.1	0.4386	0.822	0.888
2	34.406	FM	12973.5	383.5	0.5639	99.178	0.321

(*P*)-(3-Methylpenta-1,2-diene-1,5-diyl)dibenzene, (*P*)-**29**

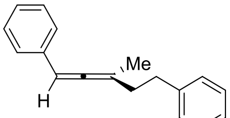

(*P*)-**29**

According to **General Procedure D** with **28** (79 mg, 0.15 mmol, >99:1 e.r.) and methyl triflate (85 µL, 0.75 mmol). Purification *via* column chromatography eluting with hexane afforded the title compound (*P*)-**29** as a colourless oil (30 mg, 85 %, 98.9:1.1 e.r.). The spectral data was identical to that described above.

$[\alpha]_D^{24}$ = +224 (c = 0.50, $CHCl_3$);

Chiral HPLC (2 x Chiralpak IB, 0 % IPA, 100 % hexane, 0.5 mL/min, 25 °C, λ = 254 nm, 5 µL injection):

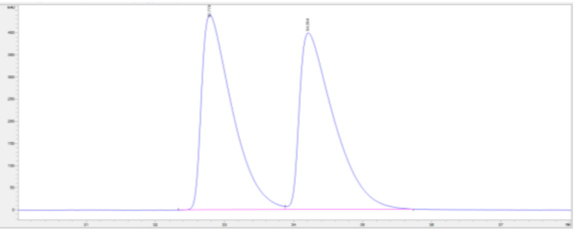

#	Time	Type	Area	Height	Width	Area%	Symmetry
1	32.779	BV	13612.2	439.6	0.3627	49.708	0.345
2	34.204	VV R	13771.8	398	0.4052	50.292	0.323

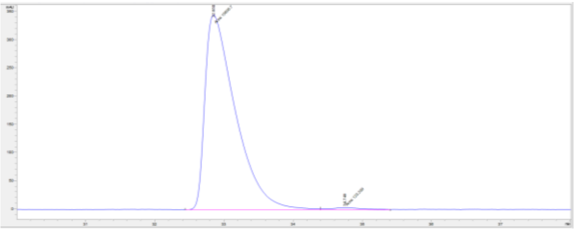

#	Time	Type	Area	Height	Width	Area%	Symmetry
1	32.85	MF	10606.7	345.5	0.5116	98.851	0.392
2	34.749	FM	123.3	3.4	0.6078	1.149	0.723

(*P*)-1-Methoxy-4-(5-methylhepta-3,4-dien-1-yl)benzene, (*P*)-**31**

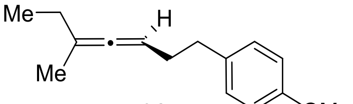

(*P*)-**31**

According to **General Procedure C** with **30** (40 mg, 0.08 mmol, 98.8:1.2 e.r.). Purification *via* column chromatography eluting with 98:2 pentane/diethyl ether afforded the title compound (*P*)-**31** as a colourless oil (13 mg, 75 %, 97.3:2.7 e.r.).

^{1}H NMR (400 MHz $CDCl_3$) δ_H = 7.04 (d, J = 8.6 Hz, 2H), 6.75 (d, J = 8.6 Hz, 2H), 5.03 – 4.98 (m, 1H), 3.71 (s, 3H), 2.57 (t, J = 7.3 Hz, 2H), 2.20 – 2.16 (m, 2H), 1.83 (qd, J = 7.3, 3.2 Hz, 2H), 1.57 (s, 3H), 0.88 (t, J = 7.4 Hz, 3H);

^{13}C NMR (101 MHz, $CDCl_3$) δ_C = 201.1, 157.8, 134.4, 129.5, 113.8, 101.8, 90.3, 55.4, 34.9, 31.5, 27.2, 19.3, 12.4;

FTIR (neat) ν/cm^{-1} = 3030, 2963, 2931, 1964, 1588, 1511, 1440, 1300, 1243, 1037, 821, 749;

HRMS (ESI^+): calculated $[M+H]^+$ for $C_{15}H_{21}O$ = 217.1587, mass found = 217.1585;

$[\alpha]_D^{22}$ = –32 (*c* = 0.50, $CHCl_3$);

Chiral HPLC (Chiralpak AS 100 % hexane, 0.1 mL/min, 0 °C, λ = 254 nm, 5 μL injection):

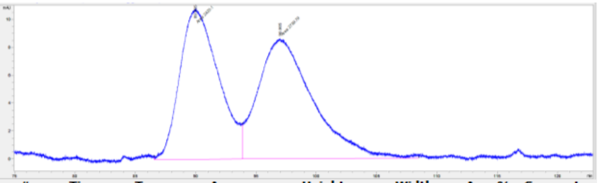

#	Time	Type	Area	Height	Width	Area%	Symmetry
1	89.965	MF	2420.1	10.9	3.7127	46.902	0.667
2	96.985	FM	2739.8	8.7	5.2608	53.098	0.637

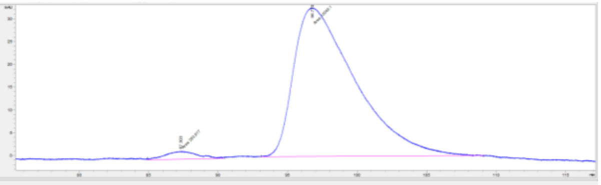

#	Time	Type	Area	Height	Width	Area%	Symmetry
1	87.303	MM	283.6	1.8	2.632	2.746	0.932
2	96.77	MM	10045.1	32.6	5.1412	97.254	0.428

(*M*)-1-Methoxy-4-(5-methylhepta-3,4-dien-1-yl)benzene, (*M*)-**31**

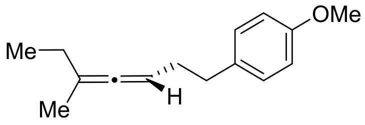

(*M*)-**31**

According to **General Procedure D** with **30** (40 mg, 0.08 mmol, 98.8:1.2 e.r.) and methyl triflate (19 µL, 0.16 mmol). Purification *via* column chromatography eluting with 98:2 pentane/diethyl ether afforded the title compound (*M*)-**31** as a colourless oil (12 mg, 70 %, 96.8:3.1 e.r.). The spectral data was identical to that described above.

$[\alpha]_D^{23}$ = +24 (*c* = 0.50, $CHCl_3$);

Chiral HPLC (Chiralpak AS 100 % hexane, 0.1 mL/min, 0 °C, λ = 254 nm, 5 μL injection):

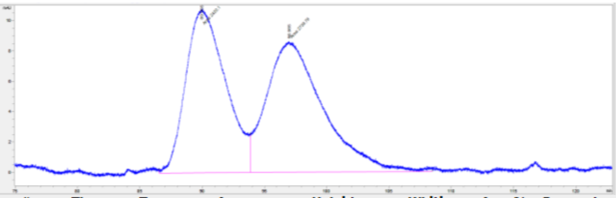

#	Time	Type	Area	Height	Width	Area%	Symmetry
1	89.965	MF	2420.1	10.9	3.7127	46.902	0.667
2	96.985	FM	2739.8	8.7	5.2608	53.098	0.637

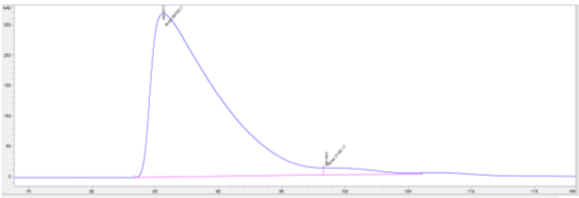

#	Time	Type	Area	Height	Width	Area%	Symmetry
1	85.677	MF	96762.7	269.7	5.9804	96.849	0.228
2	98.581	FM	3148.2	11.9	4.4042	3.151	7.35E-2

(*M*)-1-Methoxy-4-(5-phenylhexa-3,4-dien-1-yl)benzene, (*M*)-33

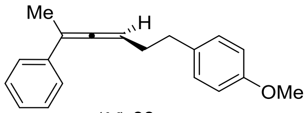

(*M*)-**33**

According to **General Procedure C** with **32** (40 mg, 0.07 mmol, 97.3:2.7 e.r.). Purification *via* column chromatography eluting with 98:2 pentane/diethyl ether afforded the title compound (*M*)-**33** as a colourless oil (16 mg, 81 %, 95.4:4.6 e.r.).

^{1}H NMR (400 MHz CDCl$_3$) δ_H = 7.22 – 7.19 (m, 4H), 7.13 – 7.07 (m, 1H), 7.05 (d, *J* = 8.6 Hz, 2H), 6.75 (d, *J* = 8.6 Hz, 2H), 5.37 (tq, *J* = 6.1, 2.9 Hz, 1H), 3.72 (s, 3H), 2.72 – 2.60 (m, 2H), 2.41 – 2.26 (m, 2H), 1.96 (d, *J* = 2.9 Hz, 3H);

^{13}C NMR (101 MHz, CDCl$_3$) δ_C = 204.3, 157.8, 137.5, 133.8, 129.5, 128.2, 126.3, 125.6, 113.7, 100.6, 92.3, 55.3, 34.6, 31.0, 17.1;

FTIR (neat) ν/cm^{-1} = 2916, 1949, 1611, 1511, 1464, 1300, 1244, 1177, 1036, 822, 755;

HRMS (ESI$^+$): calculated [M+Na]$^+$ for $C_{19}H_{20}NaO$ = 287.1406, mass found = 287.1395;

$[\alpha]_D^{25}$ = –164 (*c* = 1.00, CHCl$_3$);

Chiral HPLC (Chiralpak AS, 100 % hexane, 1 mL/min, 25 °C, λ = 254 nm, 5 μL injection):

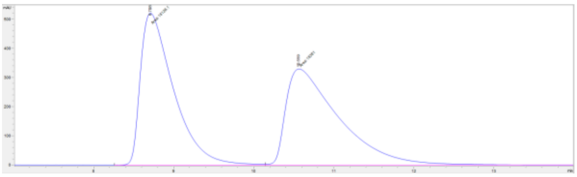

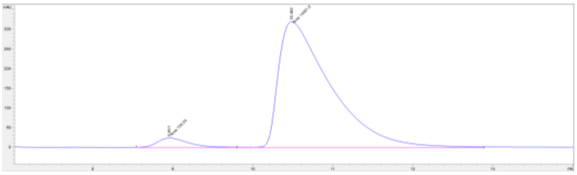

#	Time	Type	Area	Height	Width	Area%	Symmetry
1	8.708	MF	15126.1	522.2	0.4828	49.582	0
2	10.559	FM	15381	330.5	0.7757	50.418	0.31

#	Time	Type	Area	Height	Width	Area%	Symmetry
1	8.951	MF	706.5	24.9	0.4738	4.649	0.61
2	10.482	FM	14491.8	319.9	0.7551	95.351	0.331

Alternative preparation of (*M*)-**33**:

According to **General Procedure D** with **32** (40 mg, 0.07 mmol, 97.3:2.7 e.r) and methyl triflate (17 µL, 0.15 mmol). Purification *via* column chromatography eluting with 98:2 pentane/diethyl ether afforded the title compound (*M*)-**33** as a colourless oil (16 mg, 84 %, 93.6:6.4 e.r.). The spectral data was identical to that described above.

$[\alpha]_D^{24}$ = –160 (c = 1.00, $CHCl_3$);

Chiral HPLC (Chiralpak AS, 100 % hexane, 1 mL/min, 25 °C, λ = 254 nm, 5 µL injection):

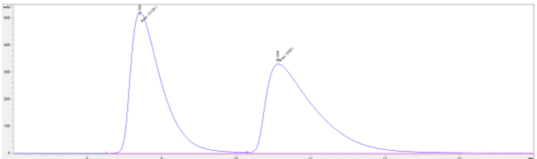

#	Time	Type	Area	Height	Width	Area%	Symmetry
1	8.708	MF	15126.1	522.2	0.4828	49.582	0
2	10.559	FM	15381	330.5	0.7757	50.418	0.31

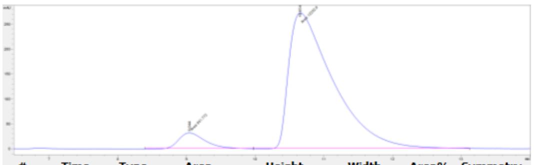

#	Time	Type	Area	Height	Width	Area%	Symmetry
1	9.044	MF	841.8	31.7	0.4431	6.438	0.7
2	10.654	FM	12232.9	272.5	0.7483	93.562	0.344

(*M*)-1-Methoxy-4-(4-methylhepta-2,3-dien-2-yl)benzene, (*M*)-**35**

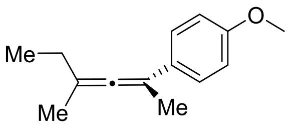

(*M*)-**35**

According to **General Procedure C** with **34** (30 mg, 0.062 mmol, 99.2:0.8 e.r.). Purification *via* column chromatography eluting with 99:1 pentane/diethyl ether afforded the title compound (*M*)-**35** as a colourless oil (10 mg, 80 %, 98.5:1.5 e.r.).

^{1}H NMR (400 MHz $CDCl_3$) δ_H = 7.23 (d, J = 8.9 Hz, 2H), 6.78 (d, J = 8.9 Hz, 2H), 3.73 (s, 3H), 2.00 – 1.97 (m, 5H), 1.70 (s, 3H), 0.95 (t, J = 7.4 Hz, 3H);

^{13}C NMR (101 MHz, $CDCl_3$) δ_C = 200.6, 158.3, 131.4, 126.7, 113.8, 103.2, 99.7, 55.5, 27.7, 19.1, 17.7, 12.5;

FTIR (neat) ν/cm^{-1} = 2962, 2930, 1968, 1577, 1508, 1439, 1372, 1290, 1175, 1034, 829, 735;

HRMS (ESI^+): calculated $[M+H]^+$ for $C_{14}H_{19}O$ = 203.1430, mass found = 203.1428;

$[\alpha]_D^{22}$ = +56 (*c* = 0.50, $CHCl_3$);

Chiral HPLC (Chiralpak IB with guard, 100 % hexane, 0.5 mL/min, 0 °C, λ = 254 nm, 5 µL injection):

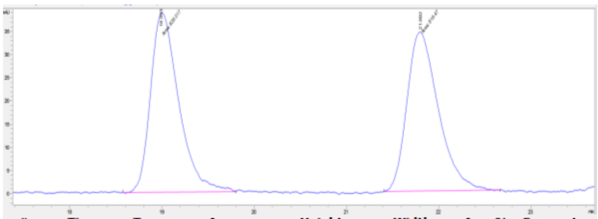

#	Time	Type	Area	Height	Width	Area%	Symmetry
1	18.993	MM	826	38.8	0.3552	50.291	0.618
2	21.802	MM	816.5	34.4	0.396	49.709	0.732

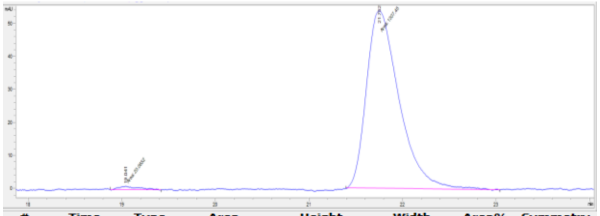

#	Time	Type	Area	Height	Width	Area%	Symmetry
1	19.041	MM	20.1	1.2	0.2775	1.511	0.486
2	21.752	MM	1307.4	53.7	0.4061	98.489	0.64

(*P*)-1-Methoxy-4-(4-methylhexa-2,3-dien-2-yl)benzene, (*P*)-**35**

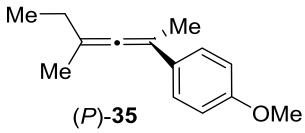

According to **General Procedure D** with **34** (40 mg, 0.082 mmol, 99.2:0.8 e.r.) and methyl triflate (19 µL, 0.16 mmol). Purification *via* column chromatography eluting with 99:1 pentane/diethyl ether afforded the title compound (*P*)-**35** as a colourless oil (13 mg, 78 %, 94.6:5.4 e.r.). The spectral data was identical to that described above.

$[\alpha]_D^{22}$ = –48 (*c* = 0.50, $CHCl_3$);

Chiral HPLC (Chiralpak IB with guard, 100 % hexane, 0.5 mL/min, 0 °C, λ = 254 nm, 5 µL injection):

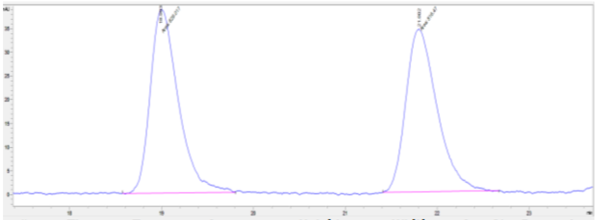

#	Time	Type	Area	Height	Width	Area%	Symmetry
1	18.993	MM	826	38.8	0.3552	50.291	0.618
2	21.802	MM	816.5	34.4	0.396	49.709	0.732

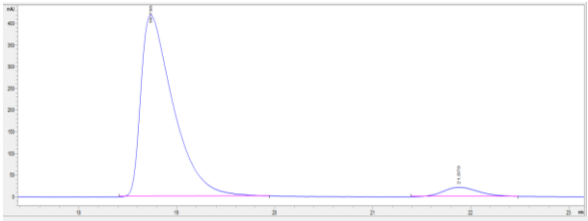

#	Time	Type	Area	Height	Width	Area%	Symmetry
1	18.735	VV R	9643.3	418.8	0.3006	94.633	0.399
2	21.879	VV R	546.9	21.8	0.2955	5.367	0.784

(*P*)-1-Methoxy-4-(4-phenylpenta-2,3-dien-2-yl)benzene, (*P*)-**37**

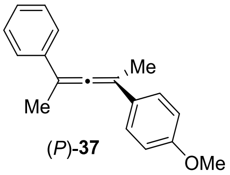

According to **General Procedure C** with **36** (40 mg, 0.08 mmol, 99.0:1.0 e.r.). Purification *via* column chromatography eluting with 99:1 pentane/diethyl ether afforded the title compound (*P*)-**37** as a colourless oil (11.4 mg, 71 %, 89.7:10.3 e.r.).

^{1}H NMR (400 MHz CDCl$_3$) δ_H = 7.37 – 7.34 (m, 2H), 7.29 – 7.22 (m, 4H), 7.15 – 7.10 (m, 1H), 6.79 (d, J = 8.8 Hz, 2H), 3.73 (s, 3H), 2.11 (s, 6H);

^{13}C NMR (101 MHz, CDCl$_3$) δ_C = 205.2, 158.6, 137.5, 129.5, 128.4, 126.9, 126.6, 125.8, 113.9, 102.2, 101.9, 55.3, 17.0, 16.9;

FTIR (neat) ν/cm^{-1} = 3078, 2980, 2939, 1945, 1575, 1511, 1442, 1325, 1254, 1181, 1026, 833, 763;

HRMS (ESI$^+$): calculated [M+H]$^+$ for $C_{18}H_{19}O$ = 251.1430, mass found = 251.1429;

$[\alpha]_D^{23}$ = +232 (c = 0.50, CHCl$_3$);

Chiral HPLC (Chiralpak IB with guard, 100 % hexane, 1.0 mL/min, 25 °C, λ = 254 nm, 5 μL injection):

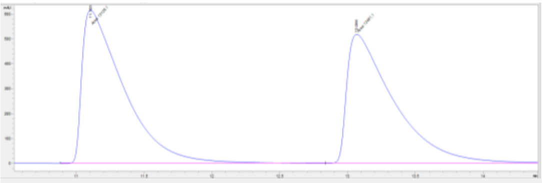

#	Time	Type	Area	Height	Width	Area%	Symmetry
1	11.105	MF	13128.1	613.4	0.3567	51.263	0.253
2	13.066	FM	12481.1	516.1	0.4031	48.737	0.253

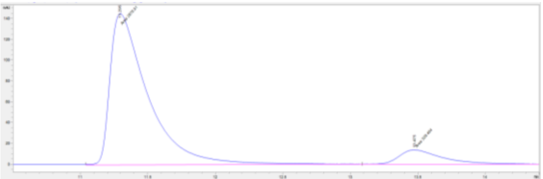

#	Time	Type	Area	Height	Width	Area%	Symmetry
1	11.295	MF	2878.5	146	0.3287	89.730	0
2	13.475	FM	329.5	14	0.3909	10.270	0.541

Alternative preparation of (*P*)-**37**:

According to **General Procedure D** with **36** (40 mg, 0.08 mmol, 99.0:1.0 e.r.) and methyl triflate (17 µL, 0.15 mmol). Purification *via* column chromatography eluting with 99:1 pentane/diethyl ether afforded the title compound (*P*)-**37** as a colourless oil (16 mg, 83 %, 93.8:6.2 e.r.). The spectral data was identical to that described above.

$[\alpha]_D^{23}$ = +238 (*c* = 0.50, $CHCl_3$);

Chiral HPLC (Chiralpak IB with guard, 100 % hexane, 1.0 mL/min, 25 °C, λ = 254 nm, 5 µL injection):

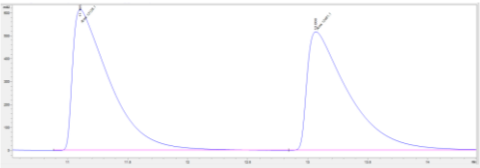

#	Time	Type	Area	Height	Width	Area%	Symmetry
1	11.105	MF	13128.1	613.4	0.3567	51.263	0.253
2	13.066	FM	12481.1	516.1	0.4031	48.737	0.253

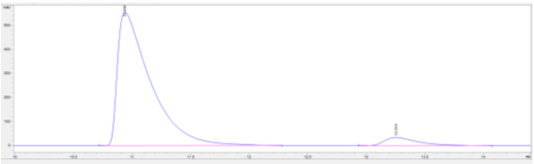

#	Time	Type	Area	Height	Width	Area%	Symmetry
1	10.936	BV R	10922.1	555.8	0.2638	93.817	0.264
2	13.254	BV R	719.8	34.6	0.2457	6.183	0.517

Alternative preparation of (*P*)-**37**:

According to **General Procedure D** with **38** (30 mg, 0.056 mmol, 99.0:1.0 e.r.) and methyl triflate (13 µL, 0.11 mmol). Purification *via* column chromatography eluting with 99:1 pentane/diethyl ether afforded the title compound (*P*)-**37** as a colourless oil (12 mg, 82 %, 89.4:10.6 e.r.). The spectral data was identical to that described above.

$[\alpha]_D^{22}$ = +230 (*c* = 0.50, $CHCl_3$);

Chiral HPLC (Chiralpak IB with guard, 100 % hexane, 1.0 mL/min, 25 °C, λ = 254 nm, 5 µL injection):

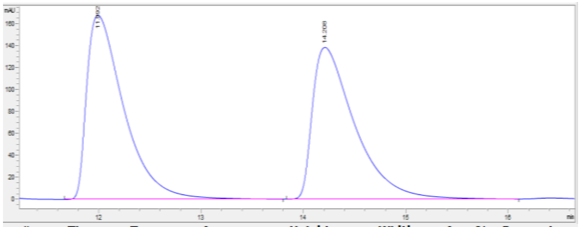

#	Time	Type	Area	Height	Width	Area%	Symmetry
1	11.992	BB	4227	167.8	0.3744	51.581	0.4
2	14.208	BB	3967.9	138.3	0.4215	48.419	0.384

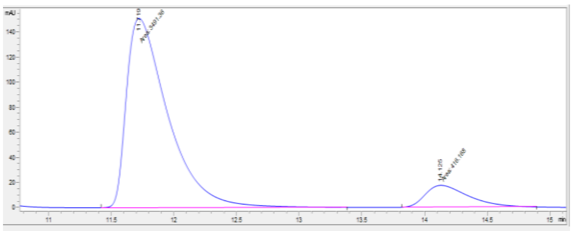

#	Time	Type	Area	Height	Width	Area%	Symmetry
1	11.719	MM	3491.4	151	0.3854	89.350	0.419
2	14.125	MM	416.2	17.4	0.3992	10.650	0.552

(*M*)-1-Methoxy-4-(4-phenylpenta-2,3-dien-2-yl)benzene, (*M*)-**37**

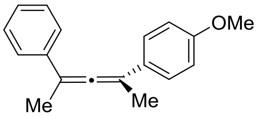

(*M*)-**37**

According to **General Procedure C** with **38** (30 mg, 0.056 mmol, 99.0:1.0 e.r.). Purification *via* column chromatography eluting with 99:1 pentane/diethyl ether afforded the title compound (*M*)-**37** as a colourless oil (11 mg, 75 %, 94.0:6.0 e.r.). The spectral data was identical to that described above.

$[\alpha]_D^{22} = -232$ (c = 0.50, $CHCl_3$);

Chiral HPLC (Chiralpak IB with guard, 100 % hexane, 1.0 mL/min, 25 °C, λ = 254 nm, 5 μL injection):

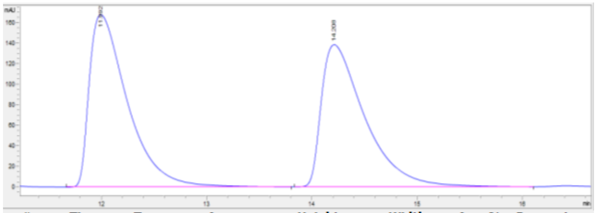

#	Time	Type	Area	Height	Width	Area%	Symmetry
1	11.992	BB	4227	167.8	0.3744	51.581	0.4
2	14.208	BB	3967.9	138.3	0.4215	48.419	0.384

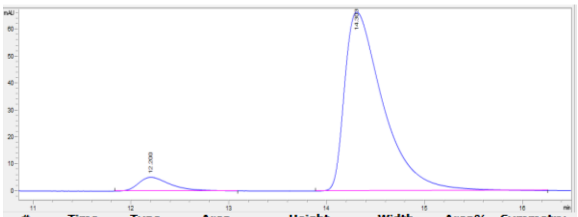

#	Time	Type	Area	Height	Width	Area%	Symmetry
1	12.2	BB	116.2	5.1	0.2712	6.008	0.616
2	14.303	BB	1818.6	66.6	0.3912	93.992	0.427

5. *DFT Simulation of Electronic Circular Dichroism (ECD) Spectra*

Methodoology: An MM2-derived conformer distribution of allene (*P*)-**37** was obtained by using Spartan. Low-energy geometries were optimised at both the B3LYP/6-311G(d,p) and the CAM-B3LYP/6-311(d,p) level. The effect of solvent was included in all calculations using the polarisable continuum model (PCM) with solvent parameters appropriate for methanol. CD spectra were calculated using time-dependent DFT at both the B3LYP/6-311G(d,p) and the CAM-B3LYP/6-311(d,p) level. The simulated ECD spectra obtained at both levels of theory were similar to each other. The data is shown below:

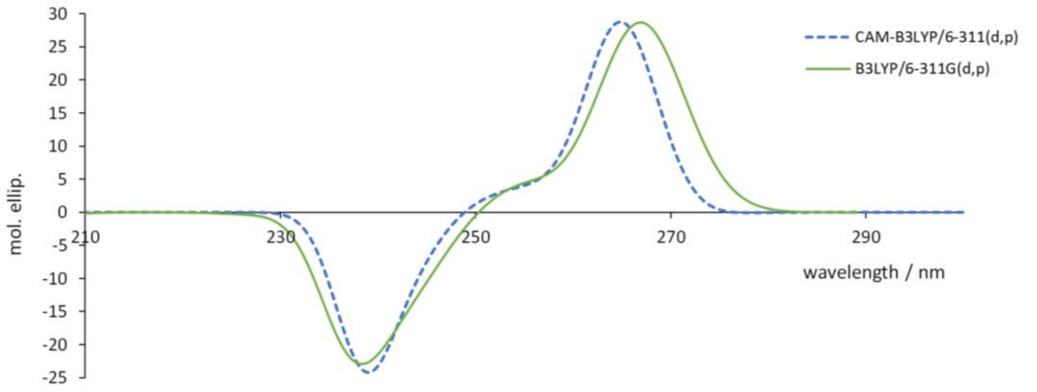

Simulated ECD spectra for allene (*P*)-**37**

Experimental: The experimental ECD spectrum of (*P*)-**37** was recorded on a JASCO J-815 CD spectrometer in methanol (concentration = 0.23 mM) at 25 °C using a cuvette with a path length of 1 mm. The experimental ECD spectra for (*P*)-**37** is shown below overlaid with the DFT simulated spectrum (CAM-B3LYP/6-311). The dominant features of the simulated spectrum are a good match for the experimental data enabling assignment of absolute configuration.

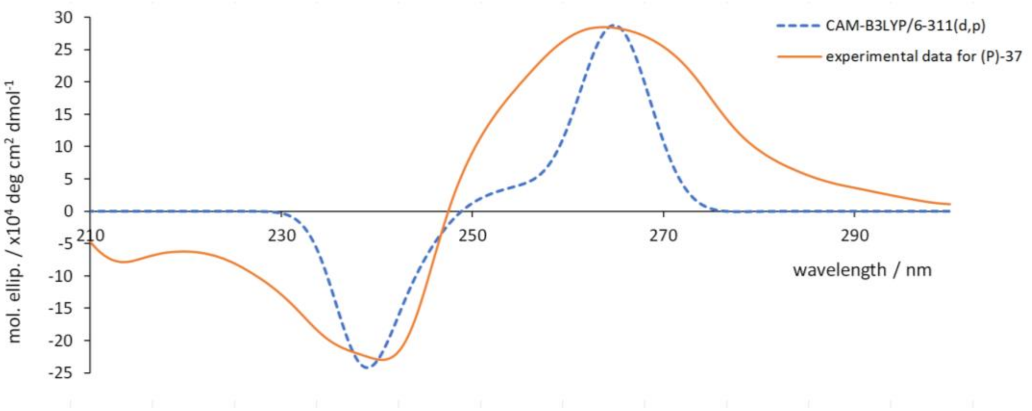

Experimental and simulated ECD spectra of allene (*P*)-**37**

6. *References*

[1] A. B. Pangborn, M. A. Gairdello, R. H. Grubbs, R. K. Rosen, F. J. Timmers, *Organometallics*, **1996**, *15*, 1518.

[2] A. F. Burchat, J. M. Chong, N. Nielsen, *J. Organomet. Chem.* **1997**, *542*, 281.

[3] *Purification of Laboratory Chemicals*, 3rd edition. D.D. Perrin, W. L. F. Armarego, *Pergamon Press*, Oxford, **1988**.

[4] R. Webber, T. J. Peglow, P. C. Nobre, A. M. Barcellos, J. A. Roehrs, R. F. Schumacher, G. Perin, *Tetrahedron Lett.* **2016**, *57*, 4128.

[5] I. N. Michaelides, B. Darses, D. J. Dixon, *Org. Lett.* **2011**, *13*, 664.

[6] X. Y. Chen, L. Wang, M. Frings, C. Bolm, *Org. Lett.* **2014**, *16*, 3796.

[7] J.V. Comasseto, J. T.B. Ferreira, N. Petragnani, *J. Organomet. Chem.* **1981**, 216, 287.

[8] I. Gazić Smilović, E. Casas-Arcé, S. J. Roseblade, U. Nettekoven, A. Zanotti-Gerosa, M. Kovačevič, Z. Časar, *Angew. Chem. Int. Ed.* **2012**, *51*, 1014.

[9] J.V. Comasseto, S. S. Silveira, J. T.B. Ferreira, V. Catani, *Synthetic Communications* **1986**, 16, 283.

[10] R. Rasappan, V. K. Aggarwal, *Nature Chem.* **2014**, *6*, 810.

[11] R. Larouche-Gauthier, T. G. Elford, V. K. Aggarwal, *J. Am. Chem. Soc.* **2011**, *133*, 16794.

[12] A. Fawcett, D. Nitsch, M. Ali, J. M. Bateman, E. L. Myers, V. K. Aggarwal, *Angew. Chem. Int. Ed.* **2016**, *55*, 14663.

[13] M. J. Hesse, S. Essafi, C. G. Watson, J. N. Harvey, D. Hirst, C. L. Willis, V. K. Aggarwal, *Angew. Chem. Int. Ed.* **2014**, *53*, 6145.

[14] D. J. Blair, C. J. Fletcher, K. M. P. Wheelhouse, V. K. Aggarwal, *Angew. Chem. Int. Ed.* **2014**, *53*, 5552.

[15] Z. Wu, X. Sun, K. Potter, Y. Cao, L. N. Zakharov, P. R. Blakemore, *Angew. Chem. Int. Ed.* **2016**, *55*, 12285.

[16] C. G. Watson, A. Balanta, T. G. Elford, S. Essafi, J. N. Harvey, V. K. Aggarwal, *J. Am. Chem. Soc.* **2014**, *136*, 17370.

[17] S. Roesner, D. Blair, V. K. Aggarwal, *Chem. Sci.* **2015**, 6, 3718.

[18] B. D. Sherry, F. D. Toste, *J. Am. Chem. Soc.* **2004**, *126*, 15978.

[19] M. Periasamy, P. O. Reddy, N. Sanjeevakumar, *Tetrahedron: Asymmetry* **2014**, *25*, 1634.

[20] W.-D. Chu, L. Zhang, Z. Zhang, Q. Zhou, F. Mo, Y. Zhang, J. Wang, *J. Am. Chem. Soc.* **2016**, *138*, 14558.

[21] H. Ohmiya, M. Yang, Y. Yamauchi, Y. Ohtsuka, M. Sawamura, *Org. Lett.* **2010**, *12*, 1796.

7. NMR Spectra

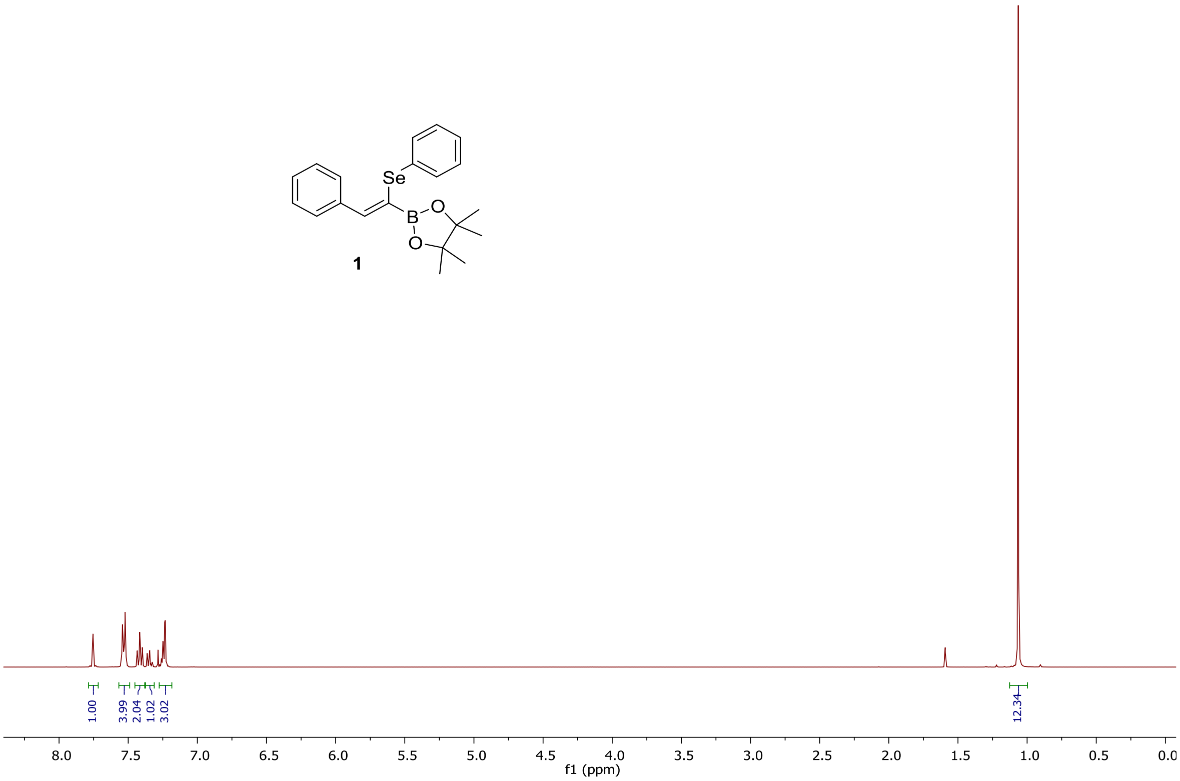

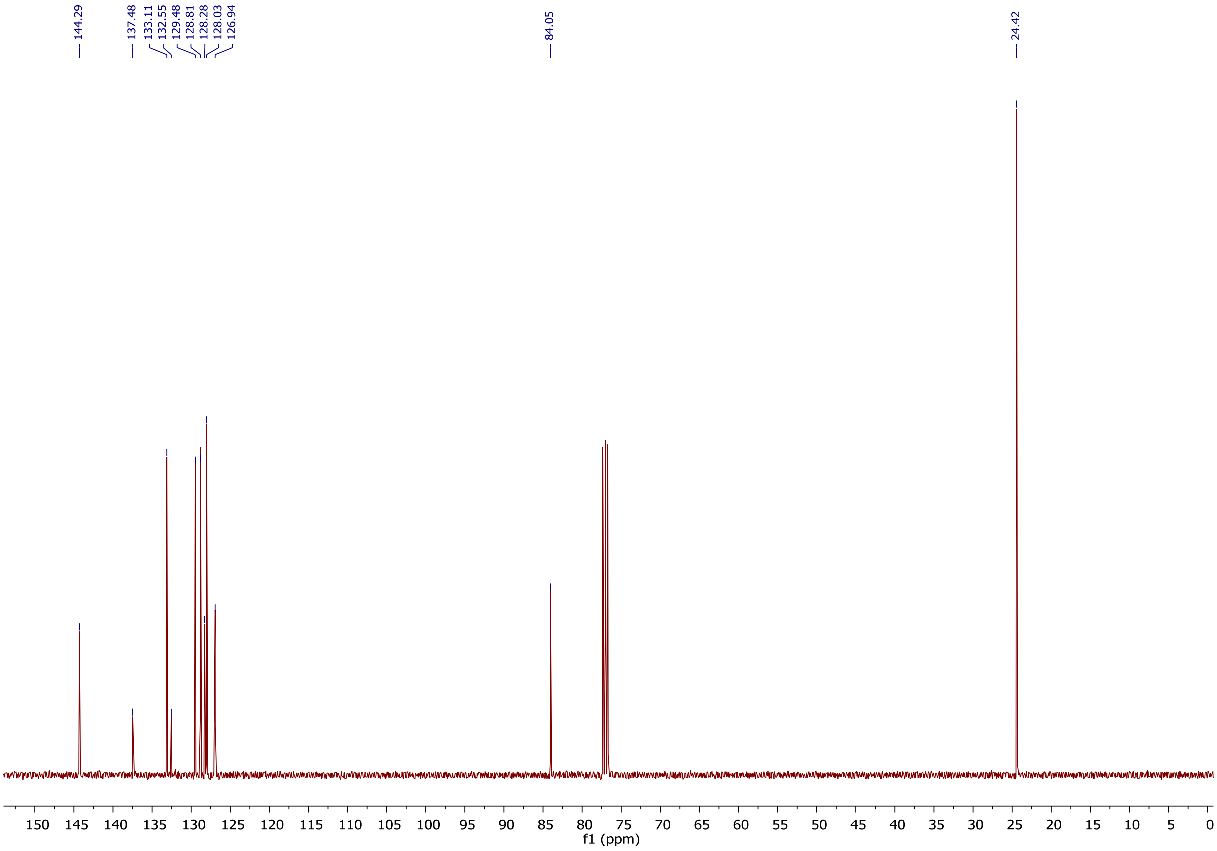

NOESY data for 1 with key correlations highlighted

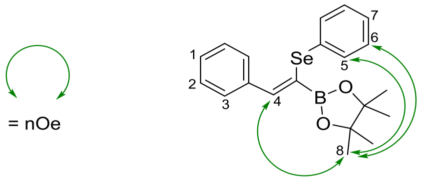

1

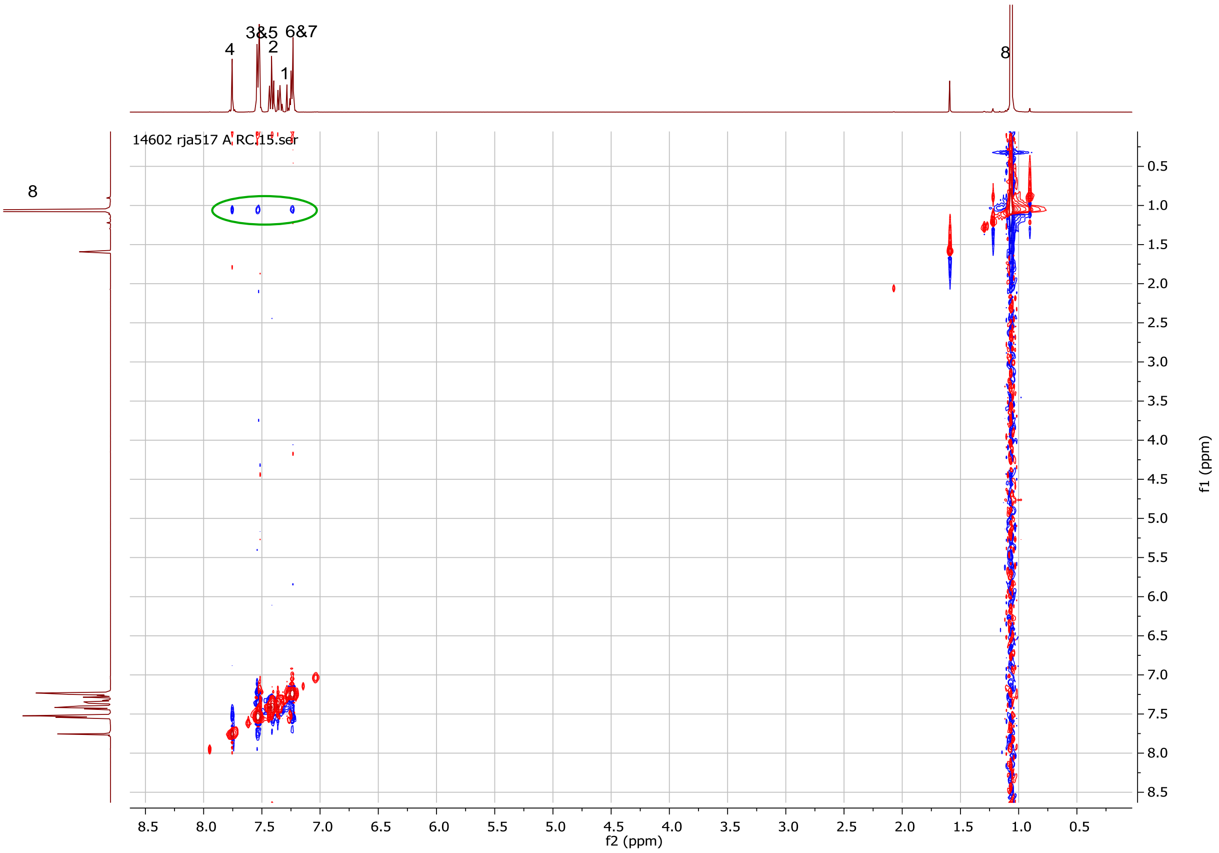

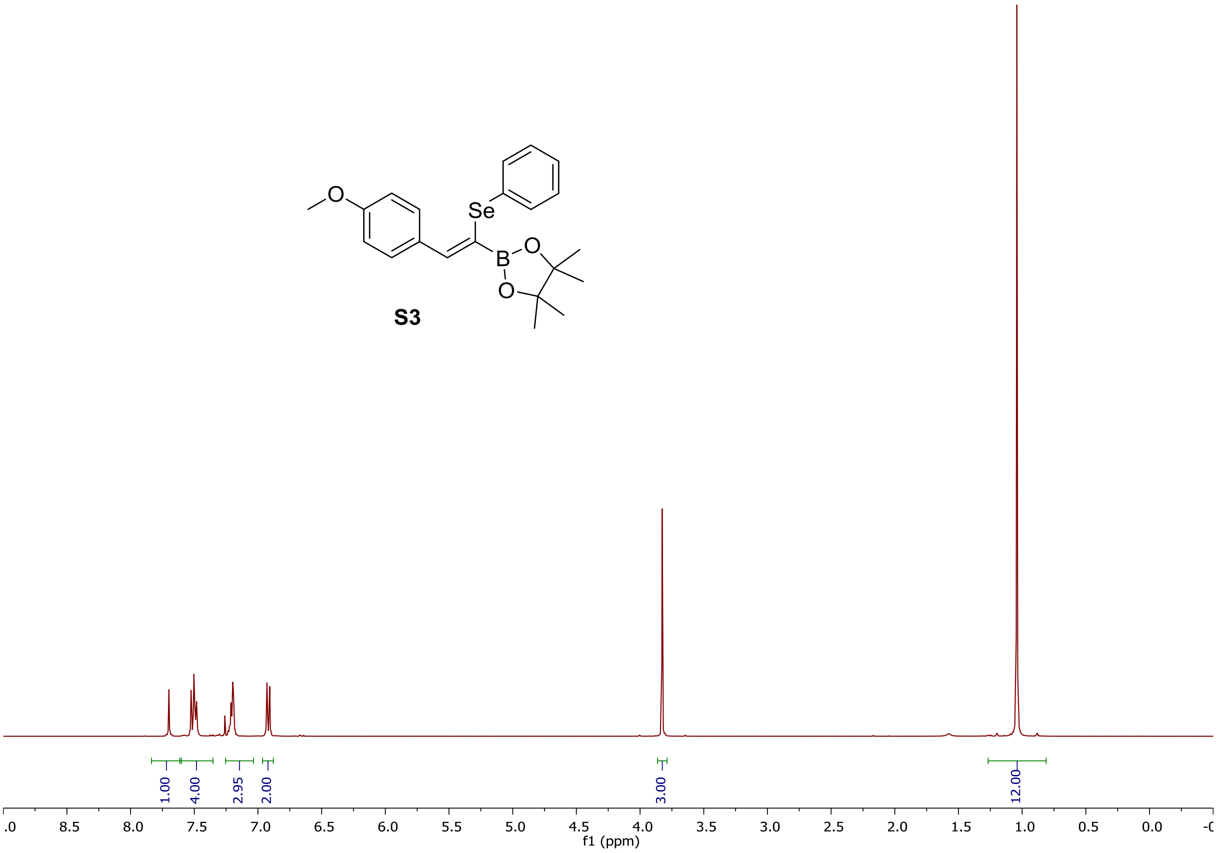
O
Se
B
O
O
1.00
4.00
2.95
2.00
3.00
12.00
f1 (ppm)

S3

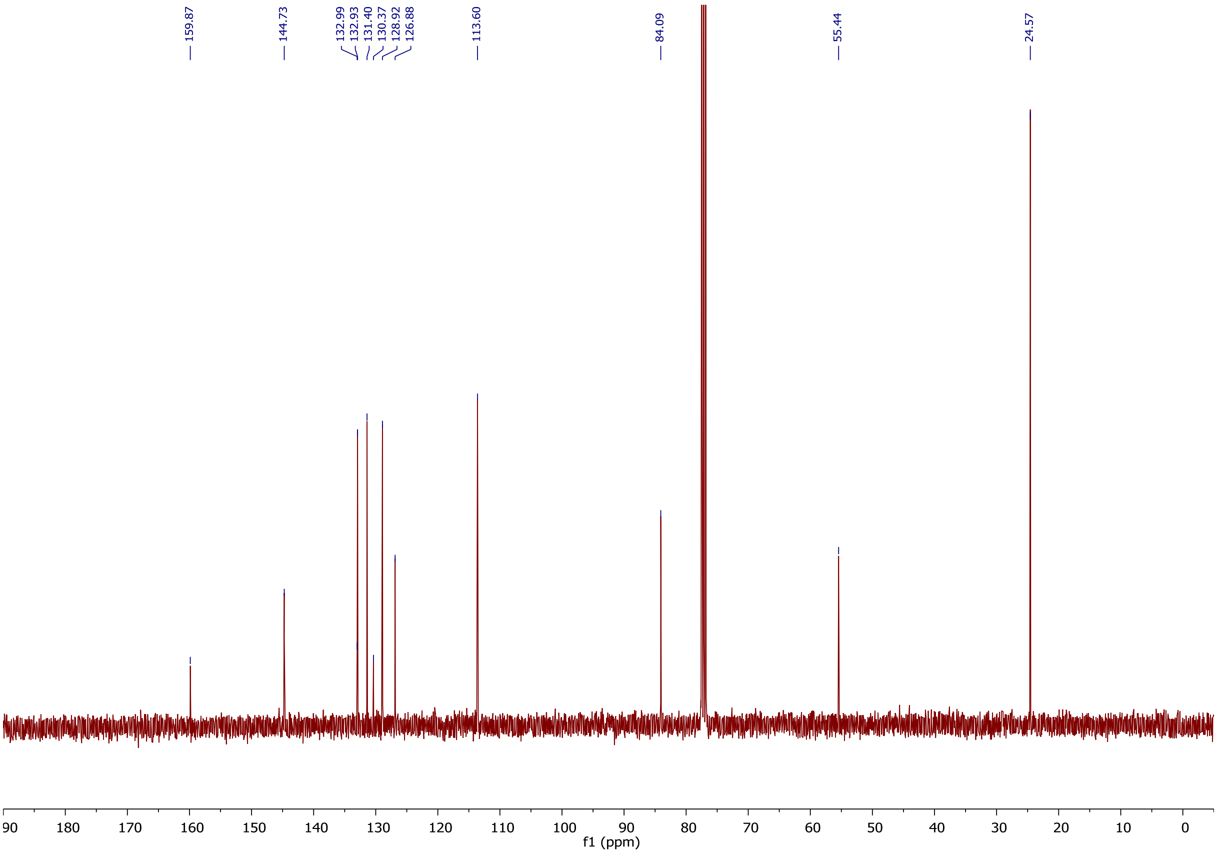
159.87
144.73
132.99
132.93
131.40
130.37
128.92
126.88
113.60
84.09
55.44
24.57
f1 (ppm)

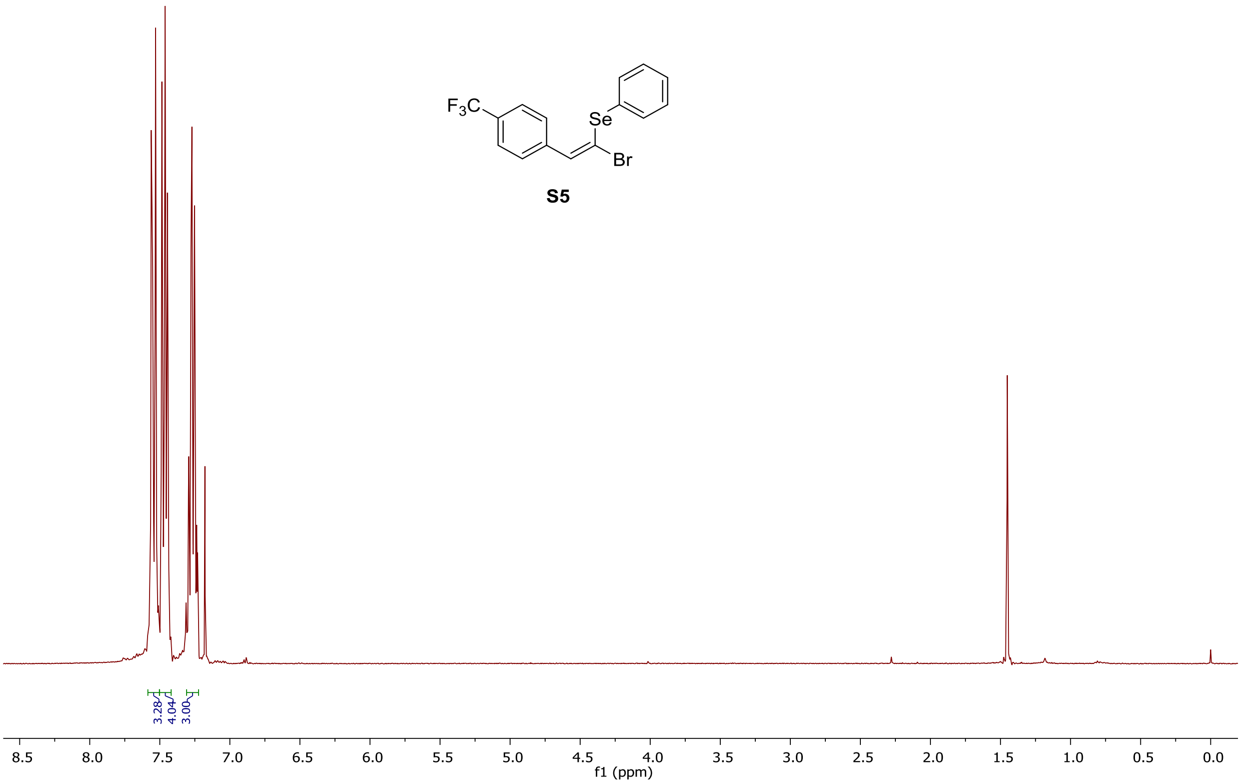
F3C
Se
Br
S5
3.28
4.04
3.00
8.5 8.0 7.5 7.0 6.5 6.0 5.5 5.0 4.5 4.0 3.5 3.0 2.5 2.0 1.5 1.0 0.5 0.0
f1 (ppm)

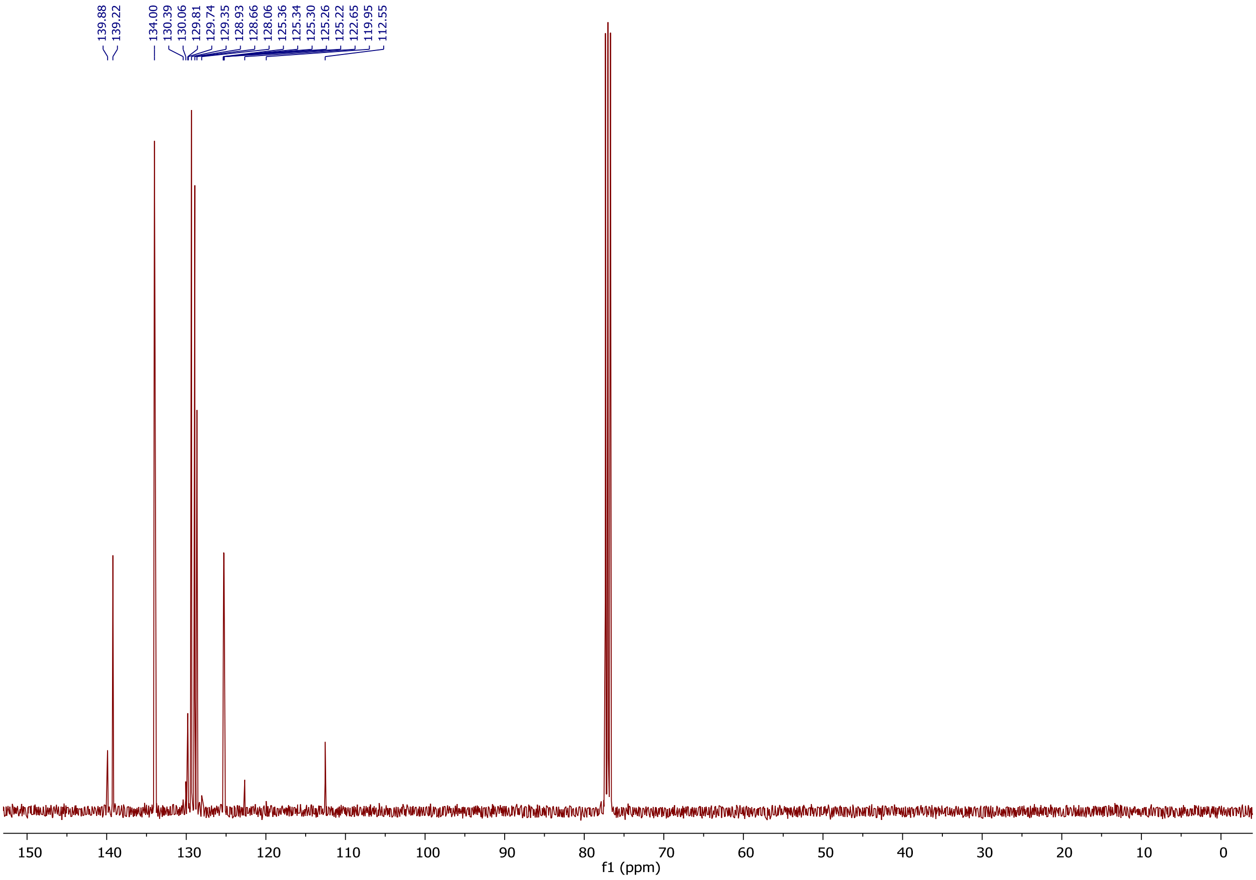
139.88
139.22
134.00
130.39
130.06
129.81
129.74
129.35
128.93
128.66
128.06
125.36
125.34
125.30
125.26
125.22
122.65
119.95
112.55
150 140 130 120 110 100 90 80 70 60 50 40 30 20 10 0
f1 (ppm)

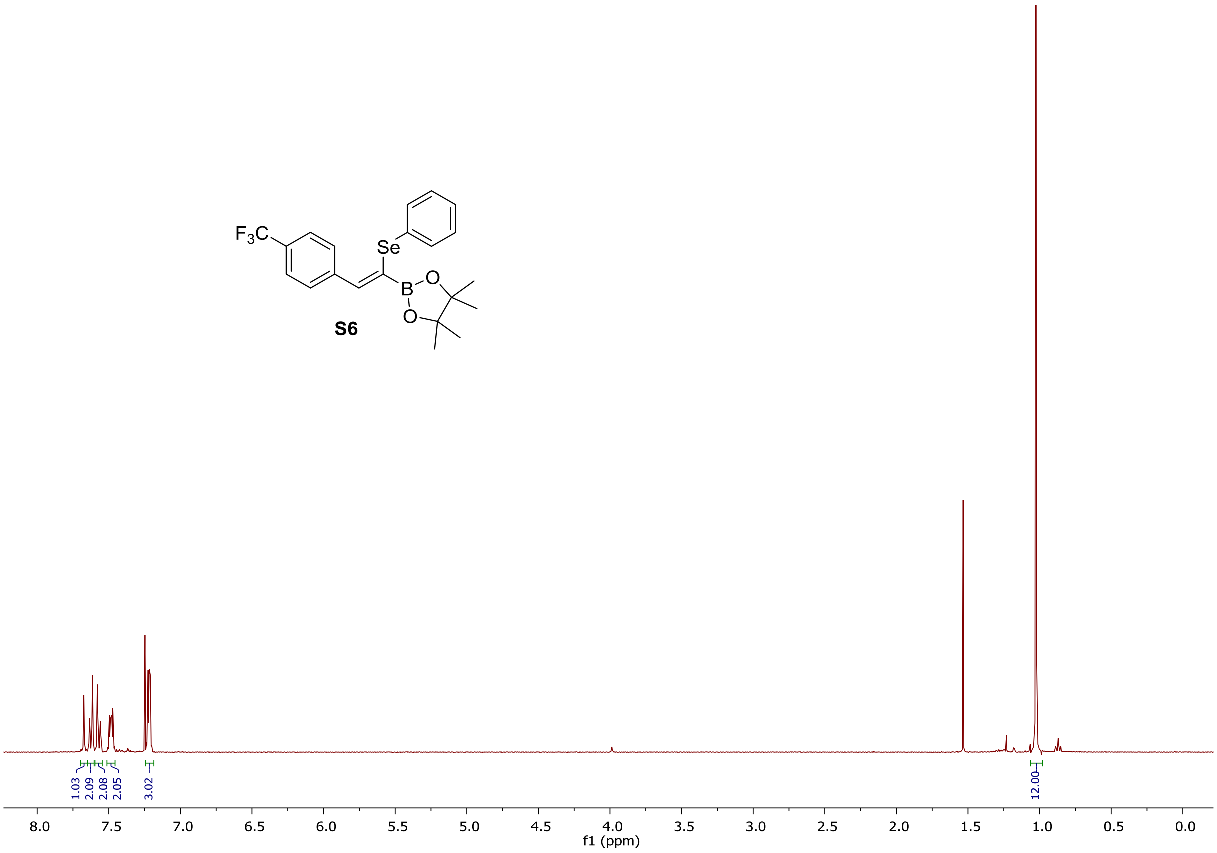
F3C
Se
B
O
O
S6
1.03
2.09
2.08
2.05
3.02
12.00
8.0 7.5 7.0 6.5 6.0 5.5 5.0 4.5 4.0 3.5 3.0 2.5 2.0 1.5 1.0 0.5 0.0
f1 (ppm)

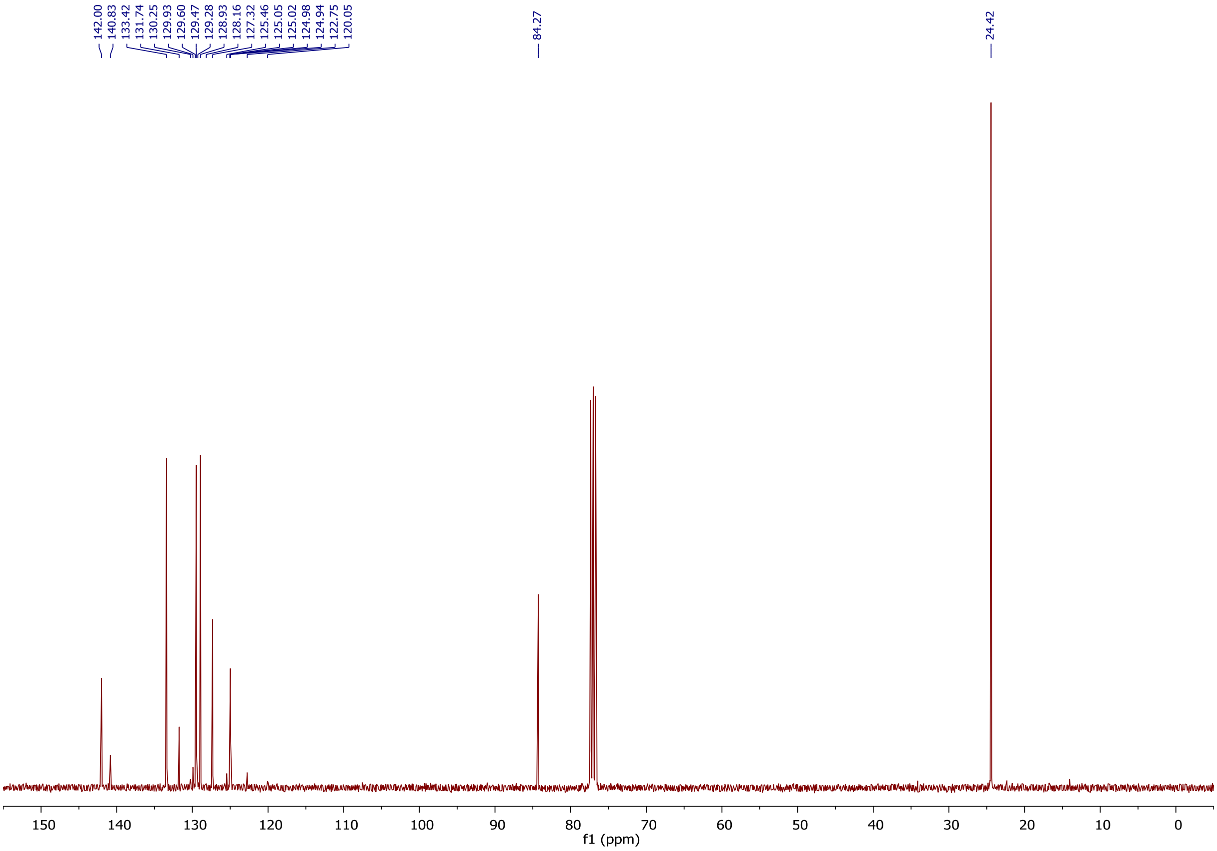
142.00
140.83
133.42
131.74
130.25
129.93
129.60
129.47
129.28
128.93
128.16
127.32
125.46
125.05
125.02
124.98
124.94
122.75
120.05
84.27
24.42
150 140 130 120 110 100 90 80 70 60 50 40 30 20 10 0
f1 (ppm)

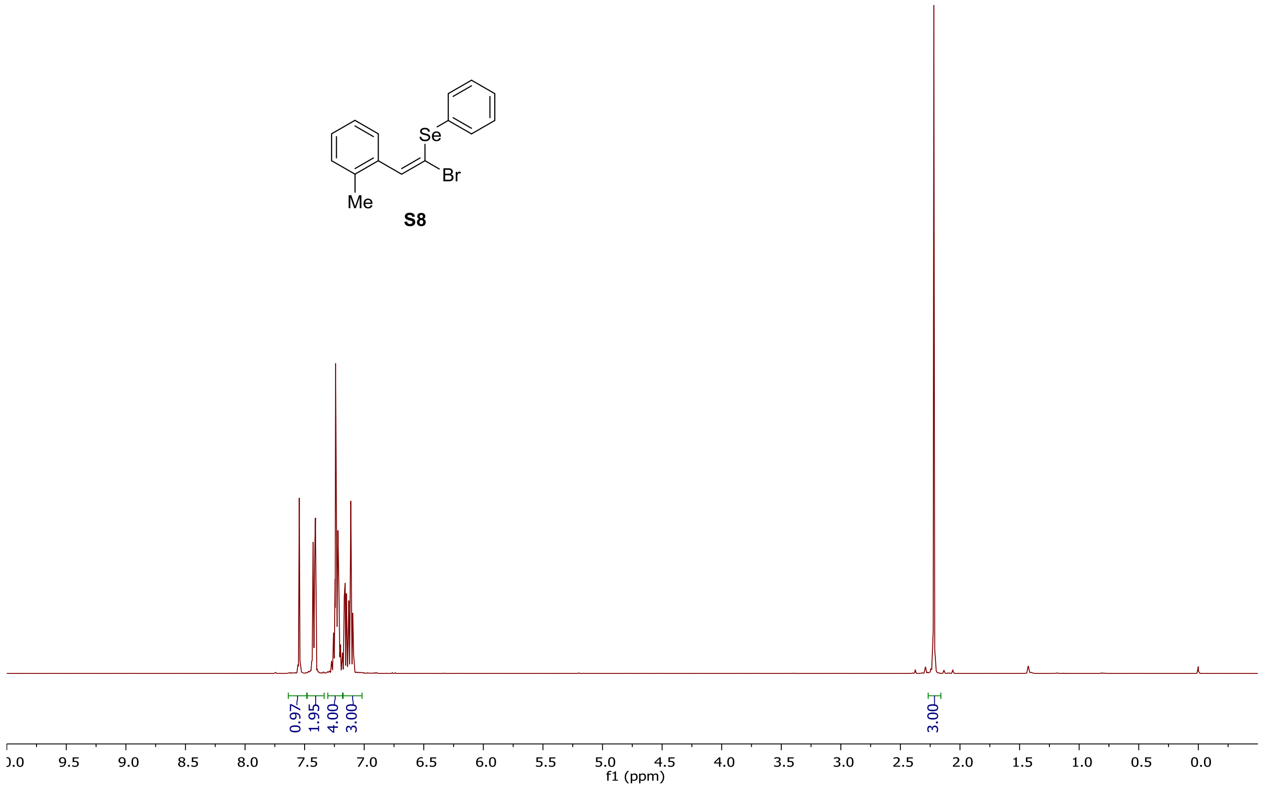
Se
Br
Me
S8
0.97
1.95
4.00
3.00
3.00
0.0 9.5 9.0 8.5 8.0 7.5 7.0 6.5 6.0 5.5 5.0 4.5 4.0 3.5 3.0 2.5 2.0 1.5 1.0 0.5 0.0
f1 (ppm)

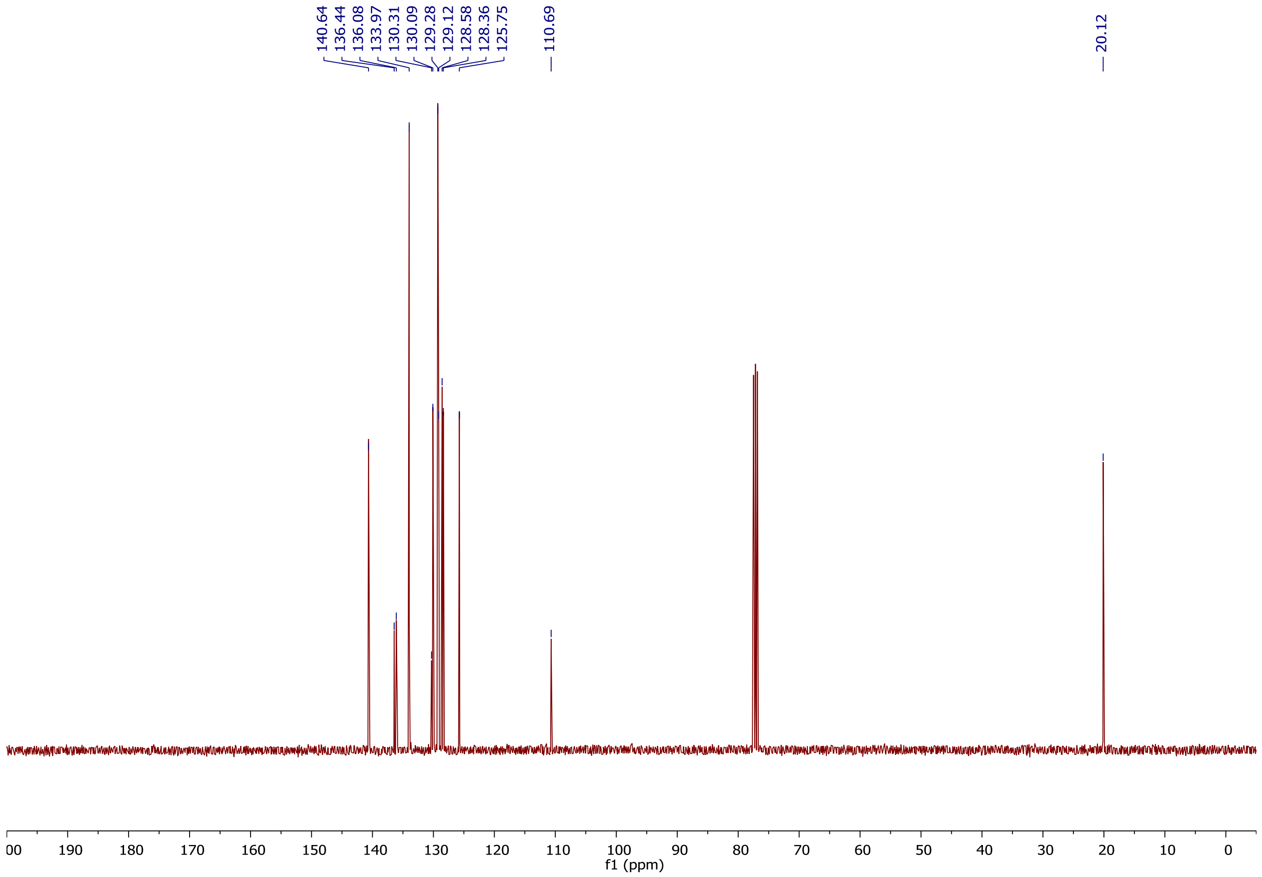
140.64
136.44
136.08
133.97
130.31
130.09
129.28
129.12
128.58
128.36
125.75
110.69
20.12
00 190 180 170 160 150 140 130 120 110 100 90 80 70 60 50 40 30 20 10 0
f1 (ppm)

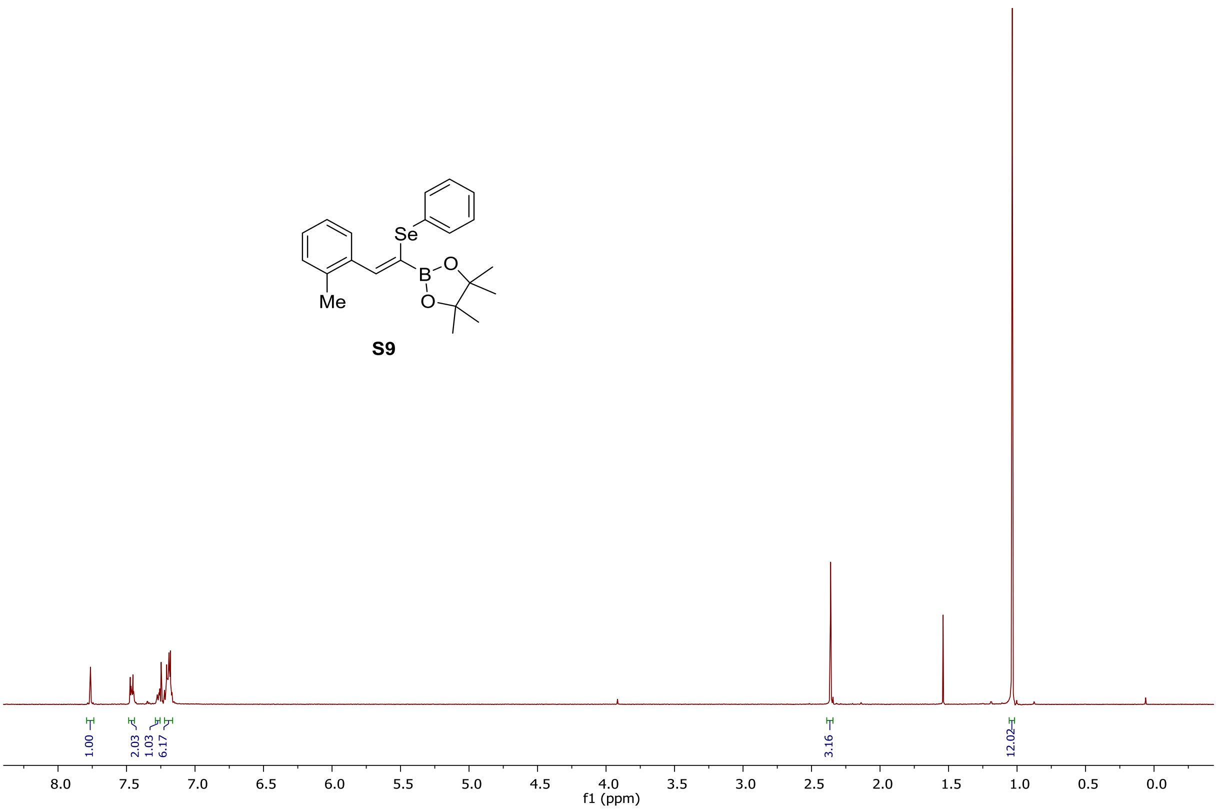

Se
B
O
O
Me
S9
1.00
2.03
1.03
6.17
3.16
12.02
8.0
7.5
7.0
6.5
6.0
5.5
5.0
4.5
4.0
3.5
3.0
2.5
2.0
1.5
1.0
0.5
0.0
f1 (ppm)

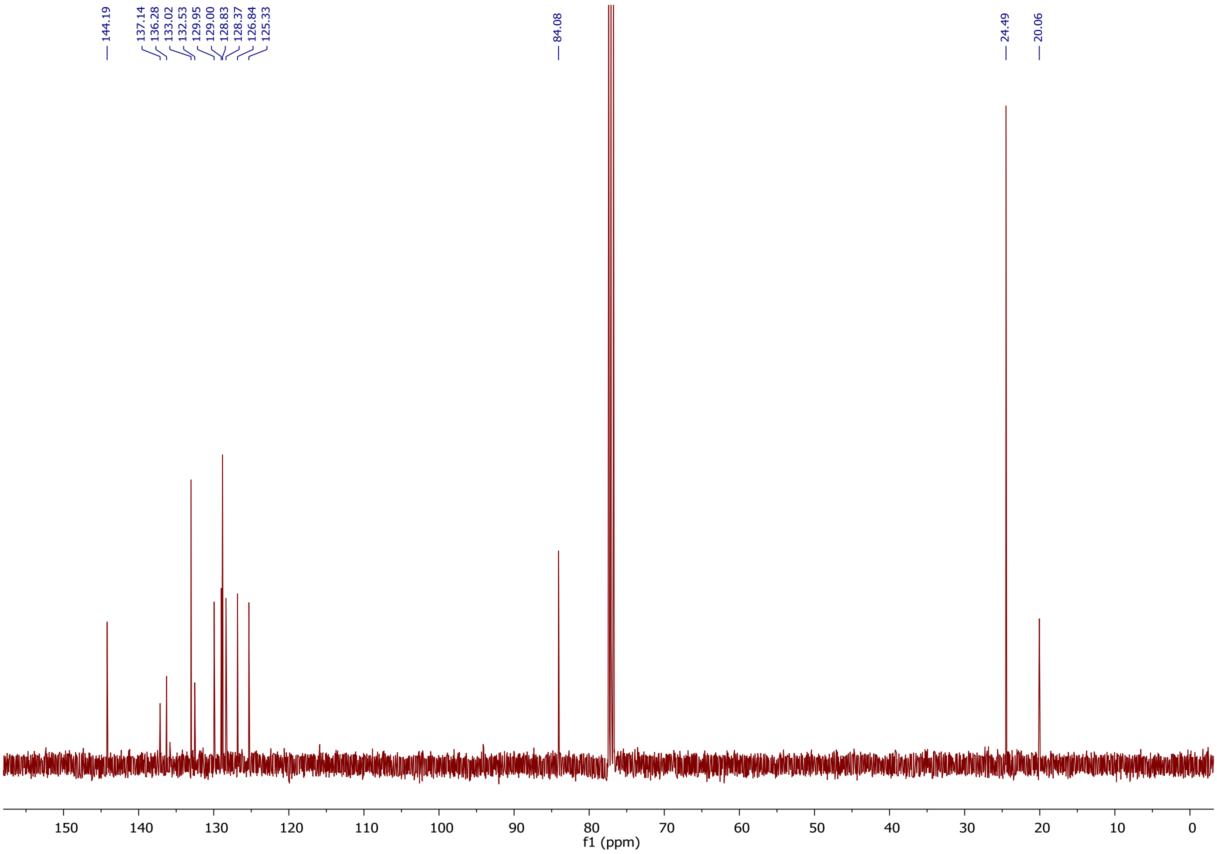

144.19
137.14
136.28
133.02
132.53
129.95
129.00
128.83
128.37
126.84
125.33
84.08
24.49
20.06
150
140
130
120
110
100
90
80
70
60
50
40
30
20
10
0
f1 (ppm)

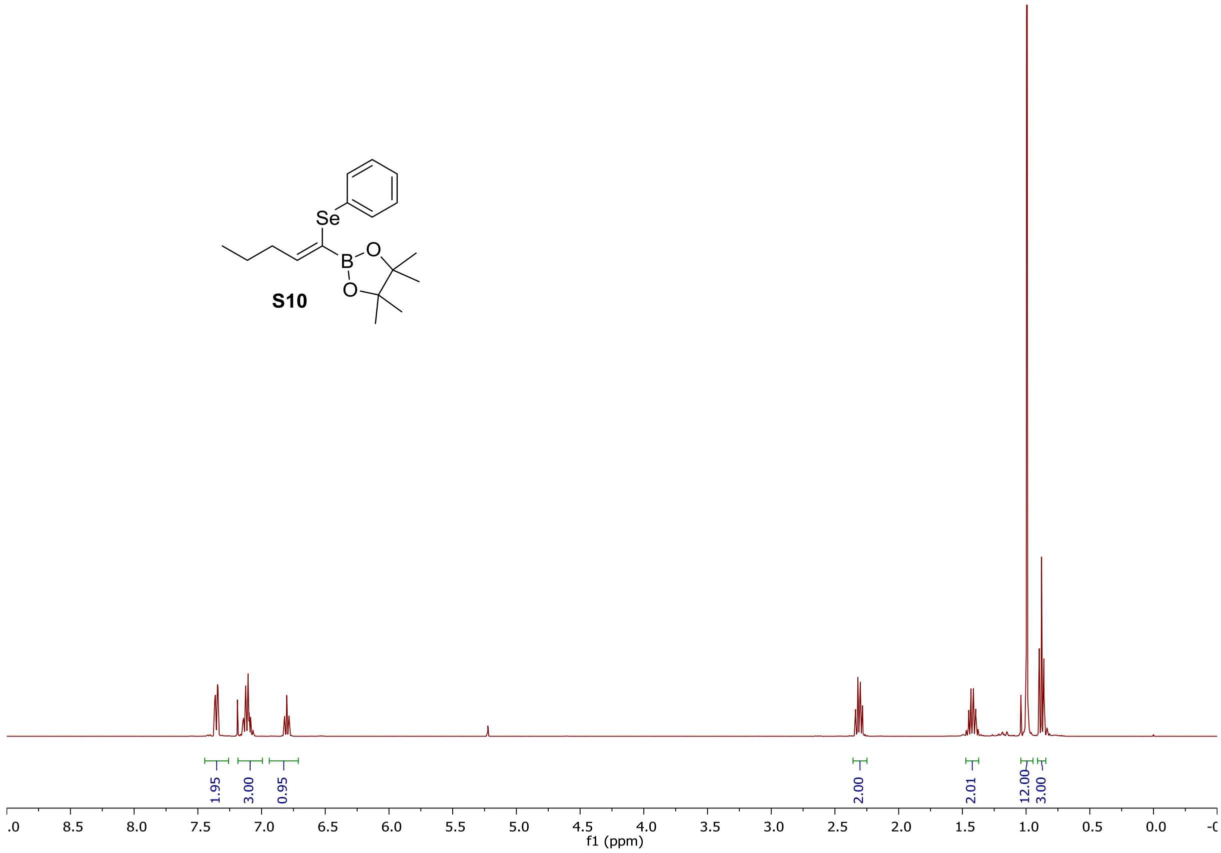
Se
B
O
O
S10
1.95
3.00
0.95
2.00
2.01
12.00
3.00
f1 (ppm)

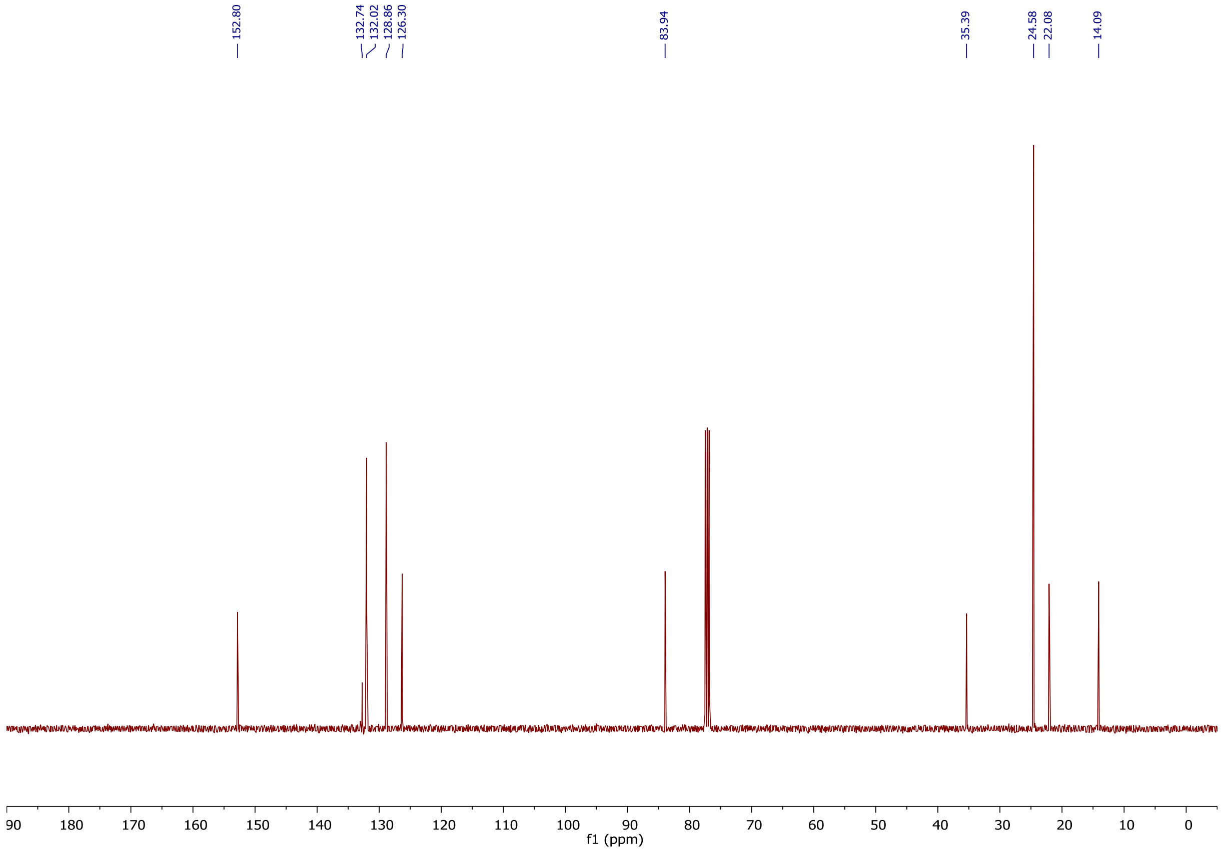
152.80
132.74
132.02
128.86
126.30
83.94
35.39
24.58
22.08
14.09
f1 (ppm)

NOESY data for S10 with key correlations highlighted

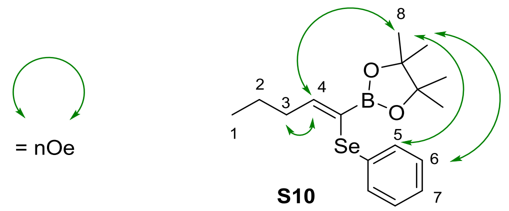

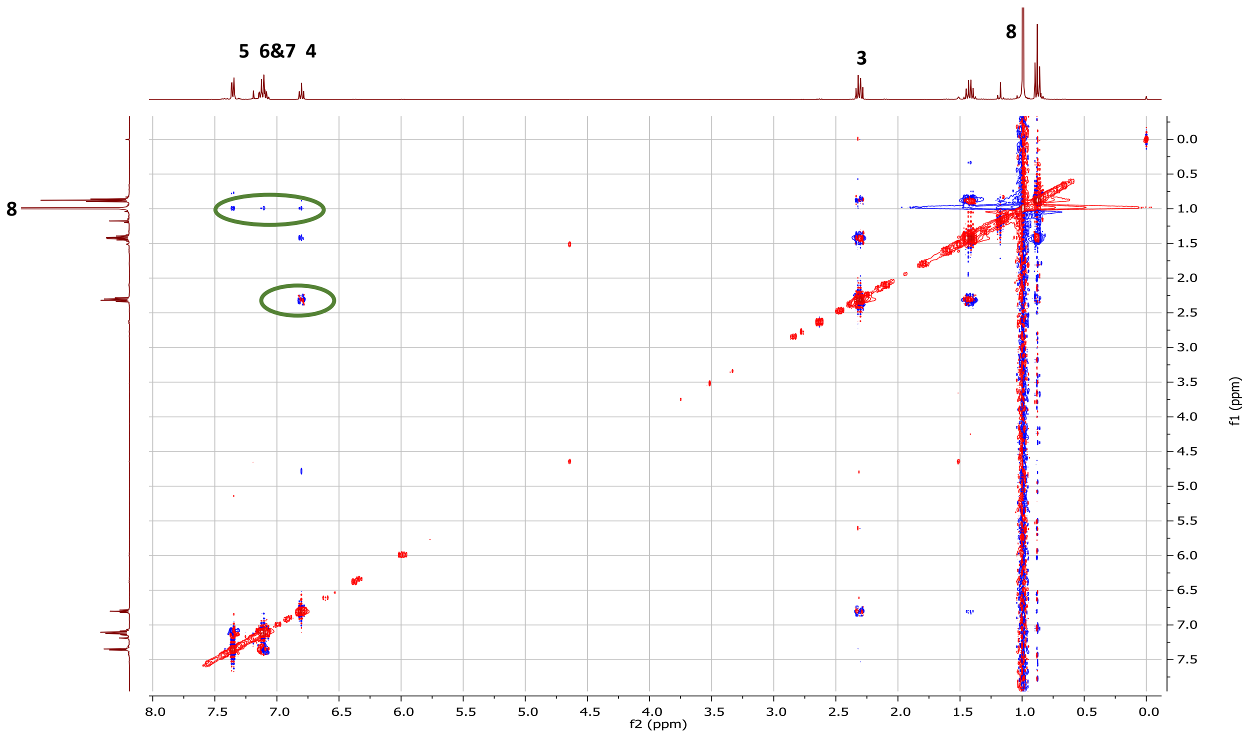

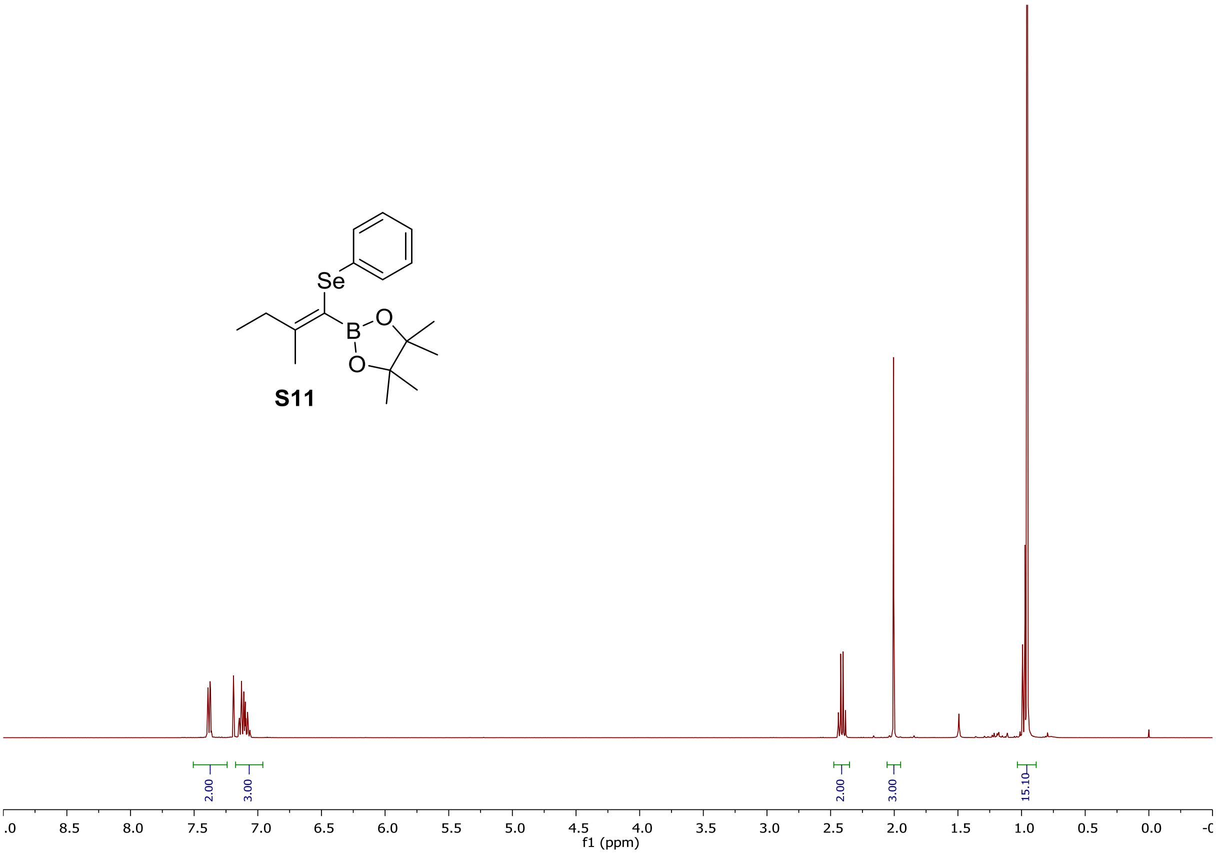
Se
B
O
O
2.00
3.00
2.00
3.00
15.10
f1 (ppm)

S11

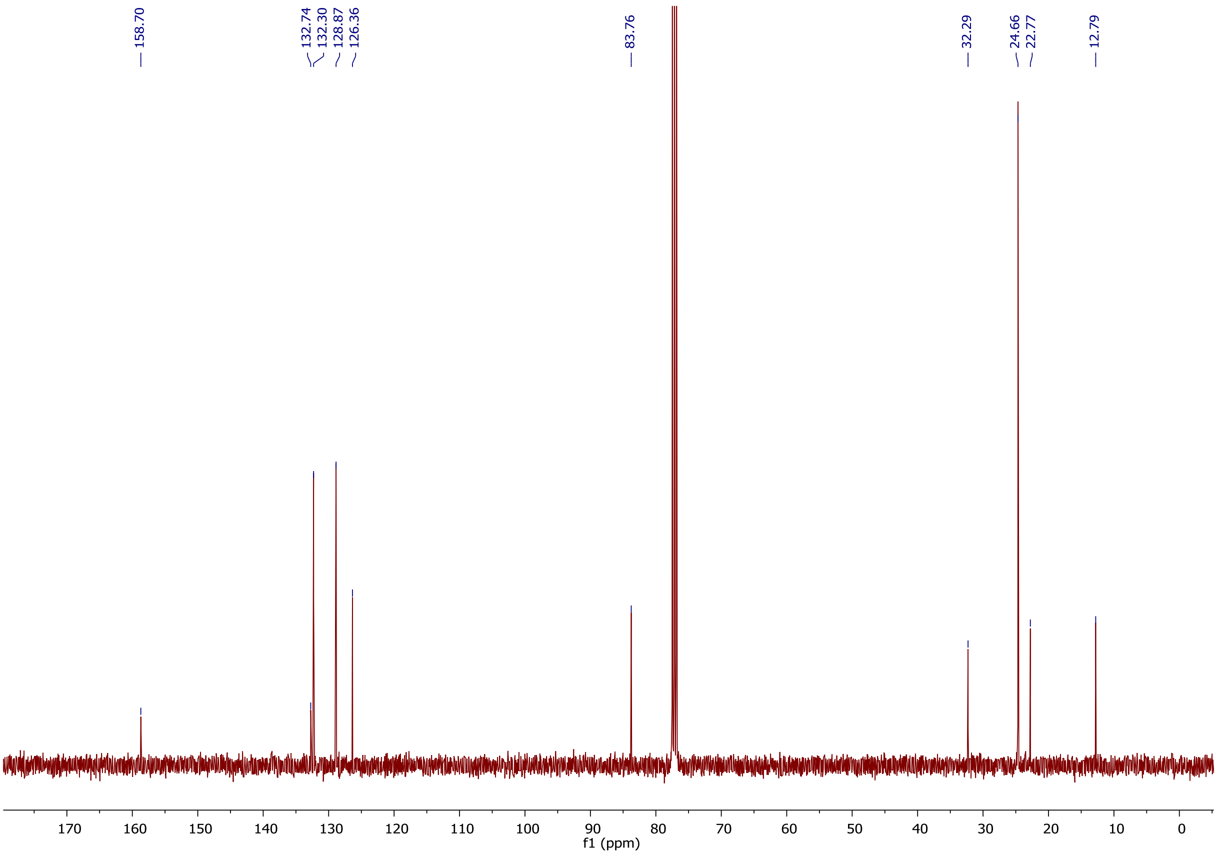
158.70
132.74
132.30
128.87
126.36
83.76
32.29
24.66
22.77
12.79
f1 (ppm)

NOESY data for S11 with key correlations highlighted

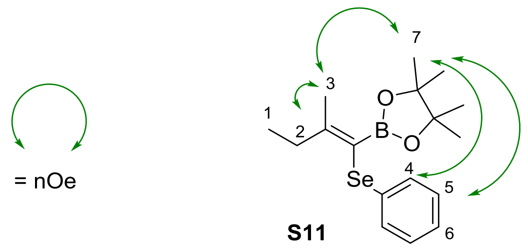

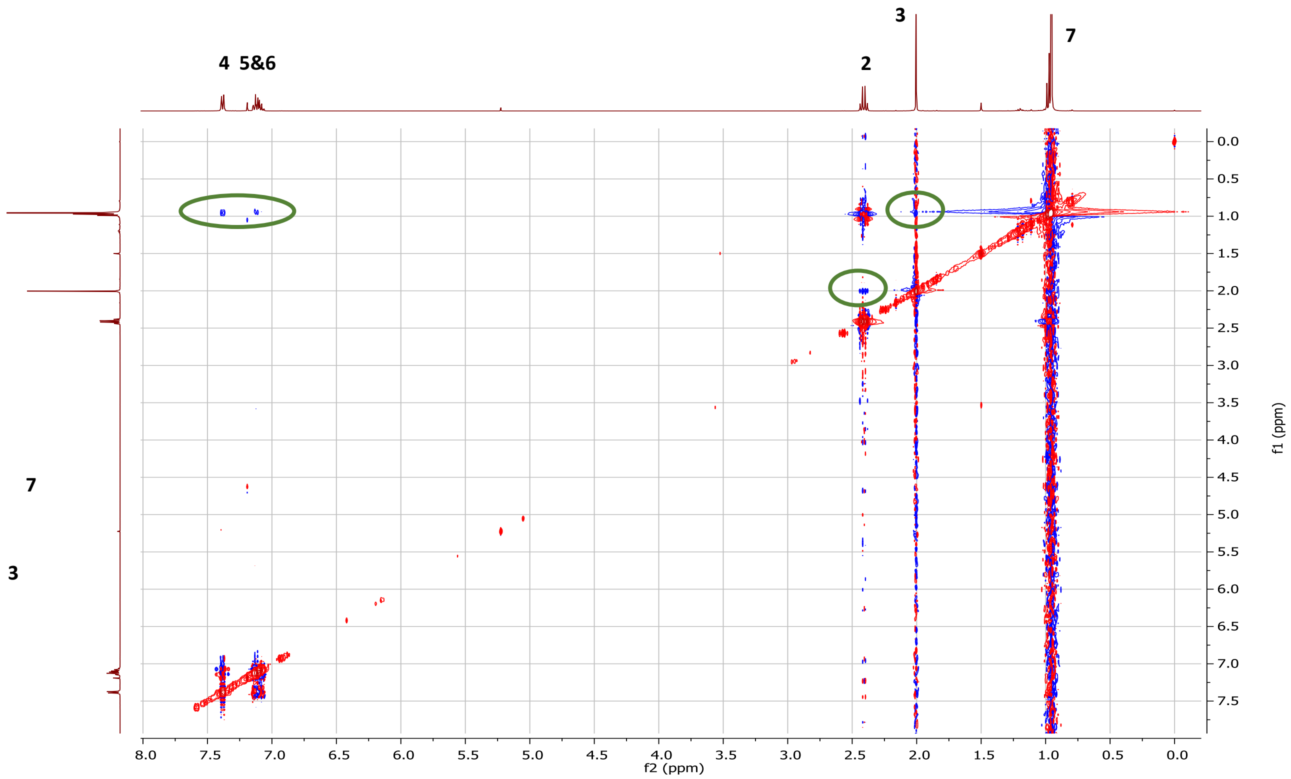

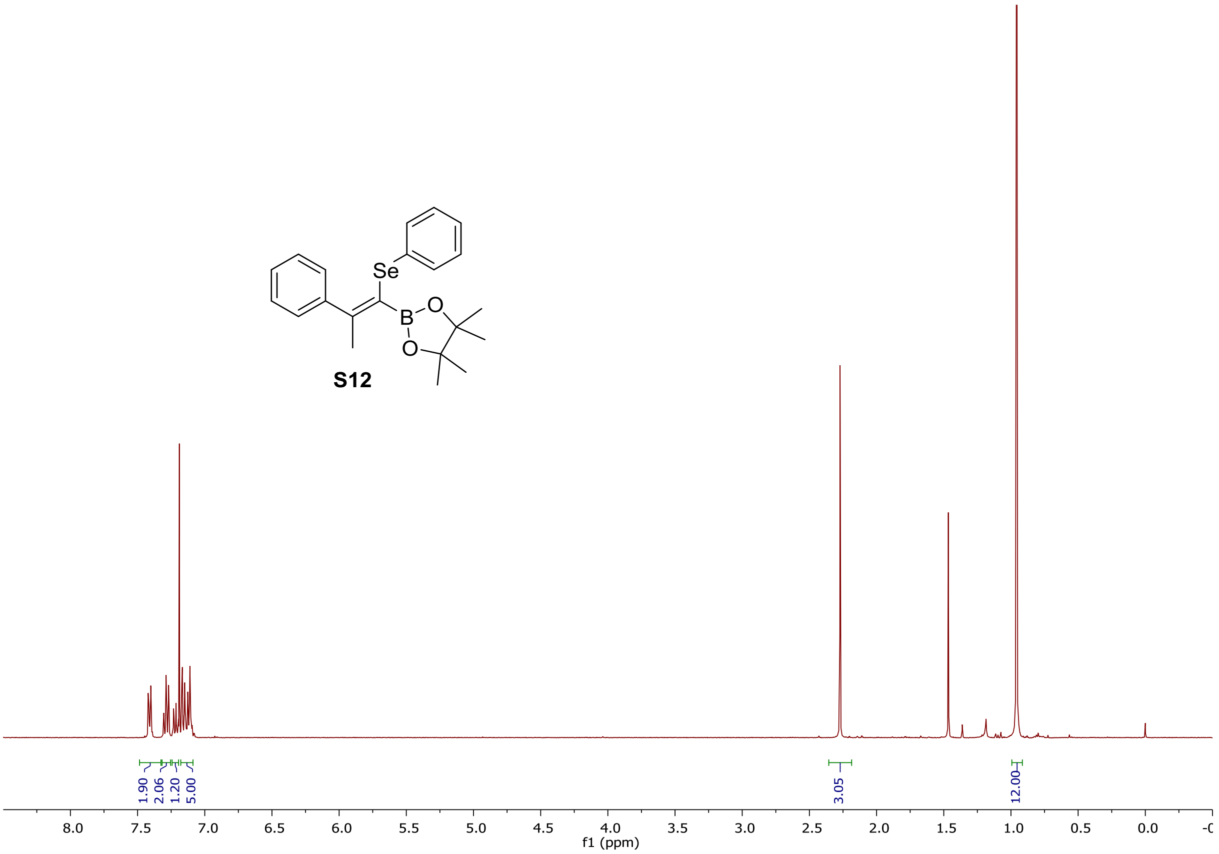
Se
B
O
O
S12
1.90
2.06
1.20
5.00
3.05
12.00
8.0 7.5 7.0 6.5 6.0 5.5 5.0 4.5 4.0 3.5 3.0 2.5 2.0 1.5 1.0 0.5 0.0
f1 (ppm)

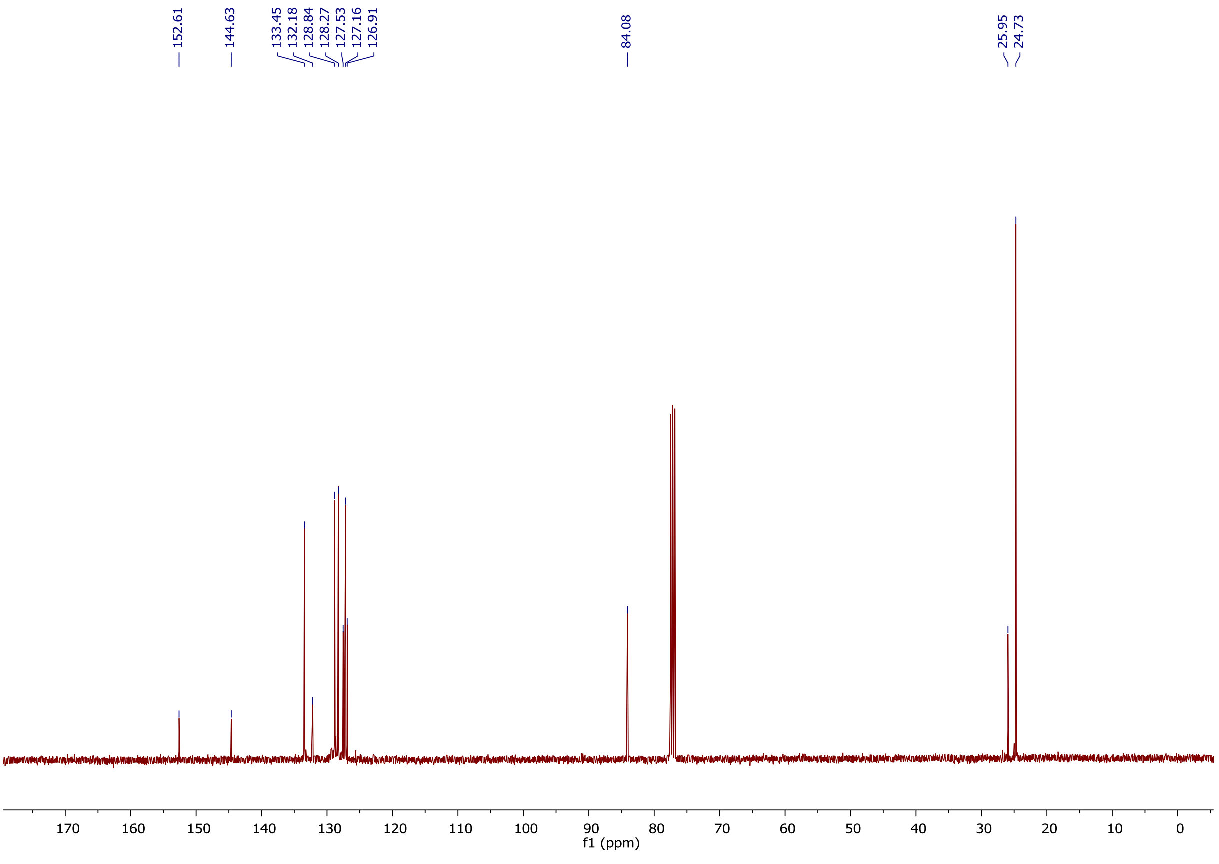
152.61
144.63
133.45
132.18
128.84
128.27
127.53
127.16
126.91
84.08
25.95
24.73
170 160 150 140 130 120 110 100 90 80 70 60 50 40 30 20 10 0
f1 (ppm)

NOESY data for S12 with key correlations highlighted

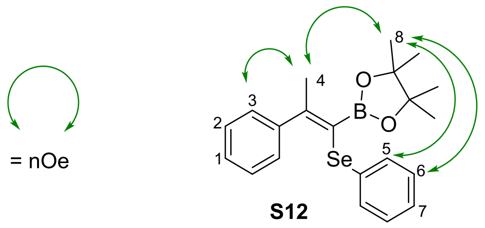

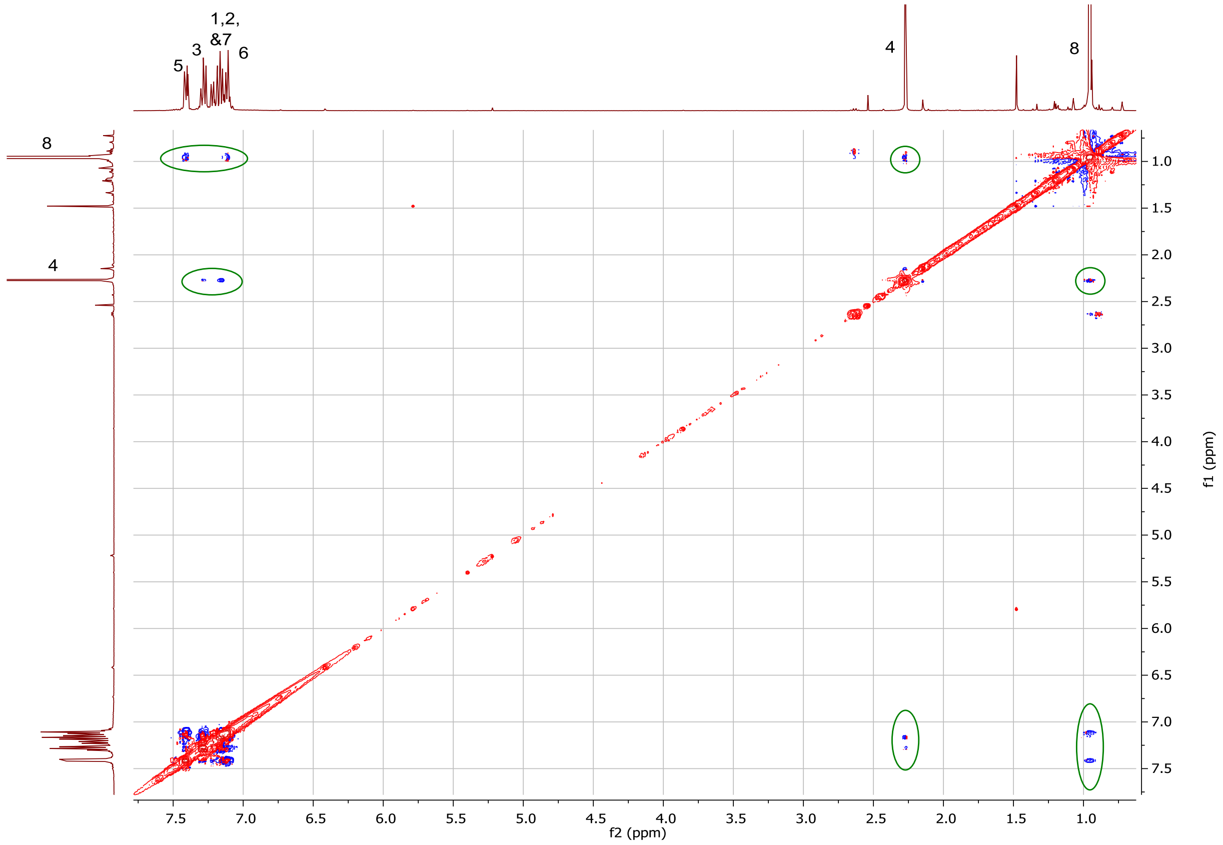

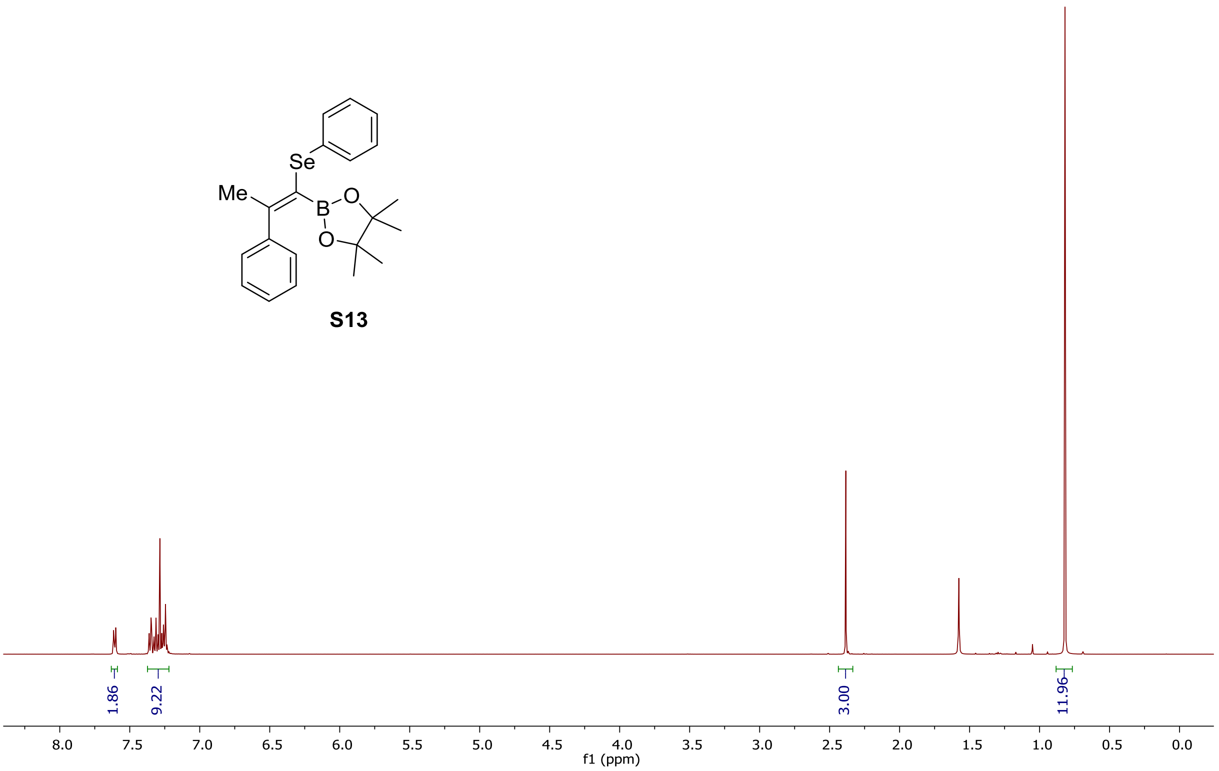
Se
Me
B
O
O
1.86
9.22
3.00
11.96
8.0
7.5
7.0
6.5
6.0
5.5
5.0
4.5
4.0
3.5
3.0
2.5
2.0
1.5
1.0
0.5
0.0
f1 (ppm)

S13

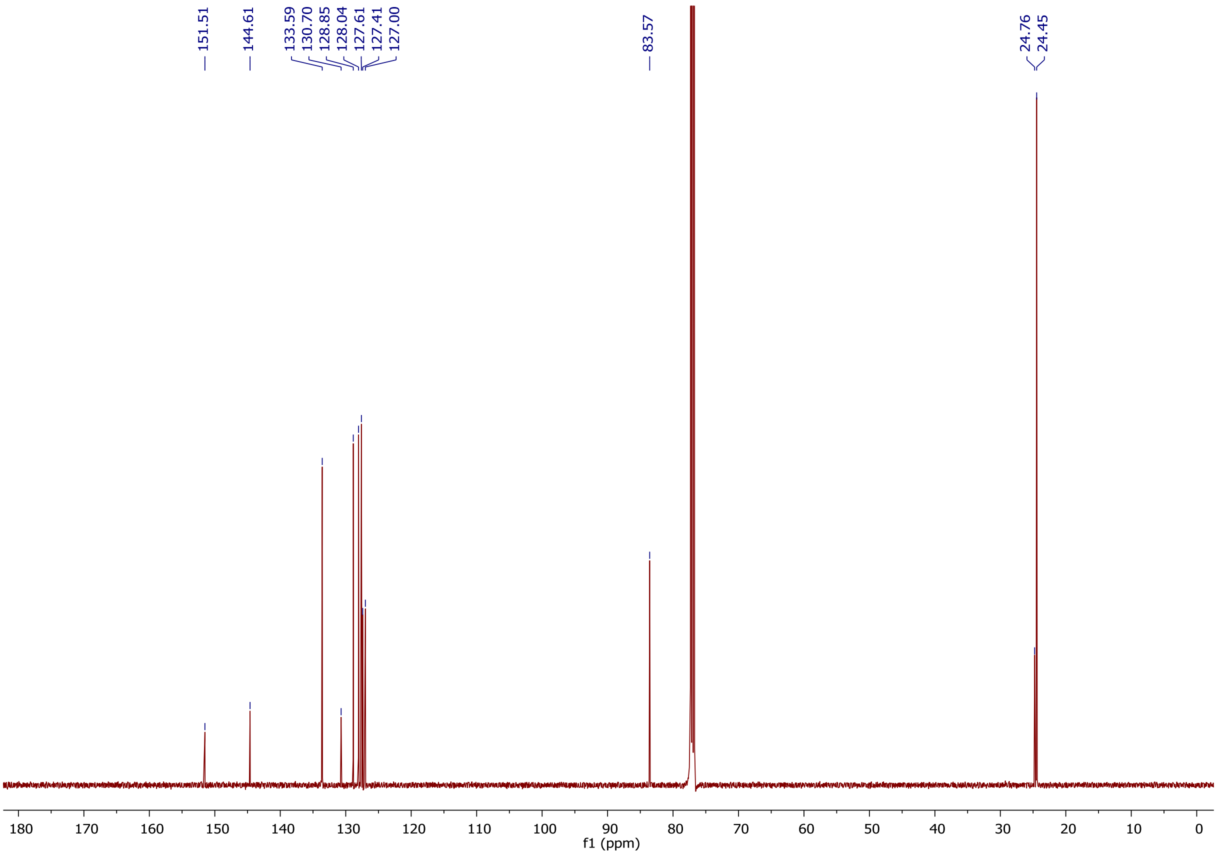
151.51
144.61
133.59
130.70
128.85
128.04
127.61
127.41
127.00
83.57
24.76
24.45
180
170
160
150
140
130
120
110
100
90
80
70
60
50
40
30
20
10
0
f1 (ppm)

NOESY data for S13 with key correlations highlighted

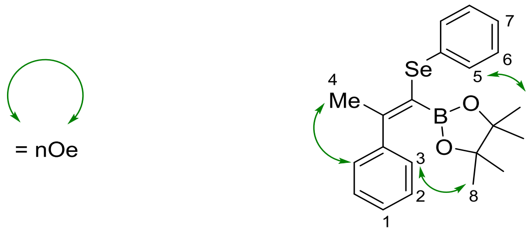

S13

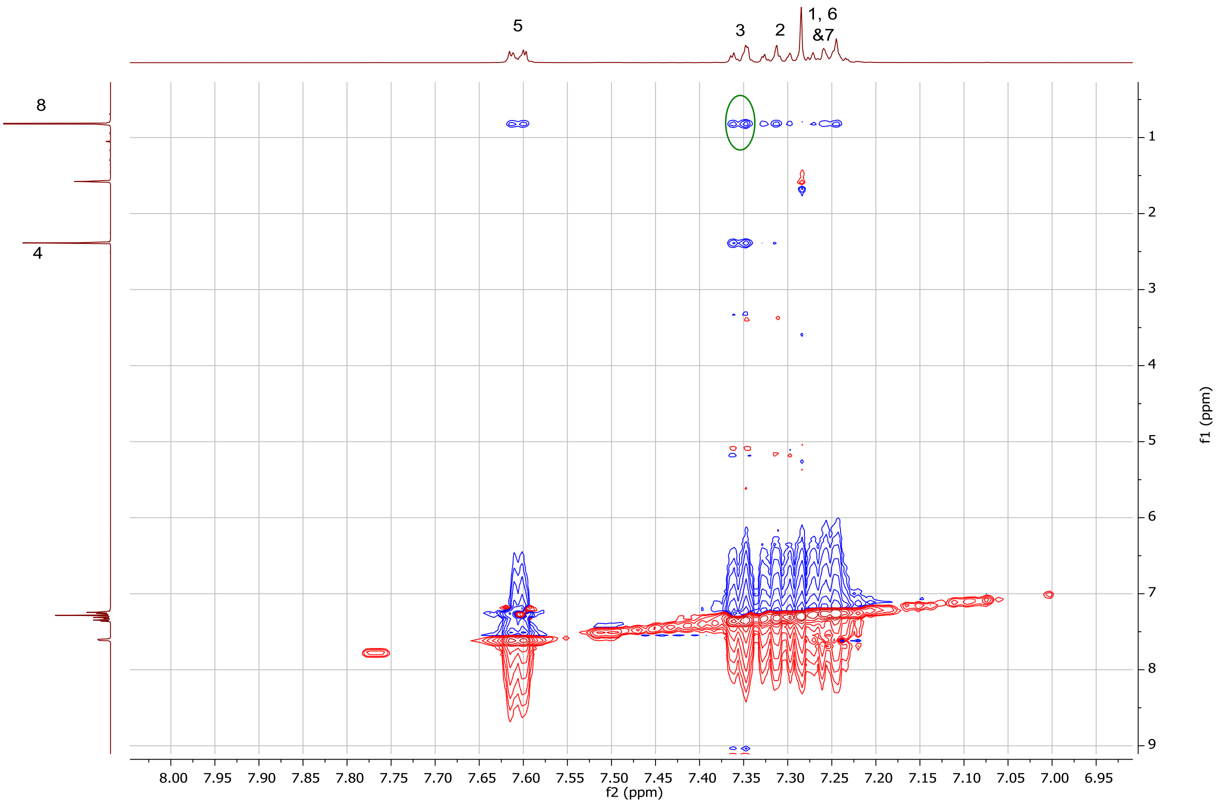

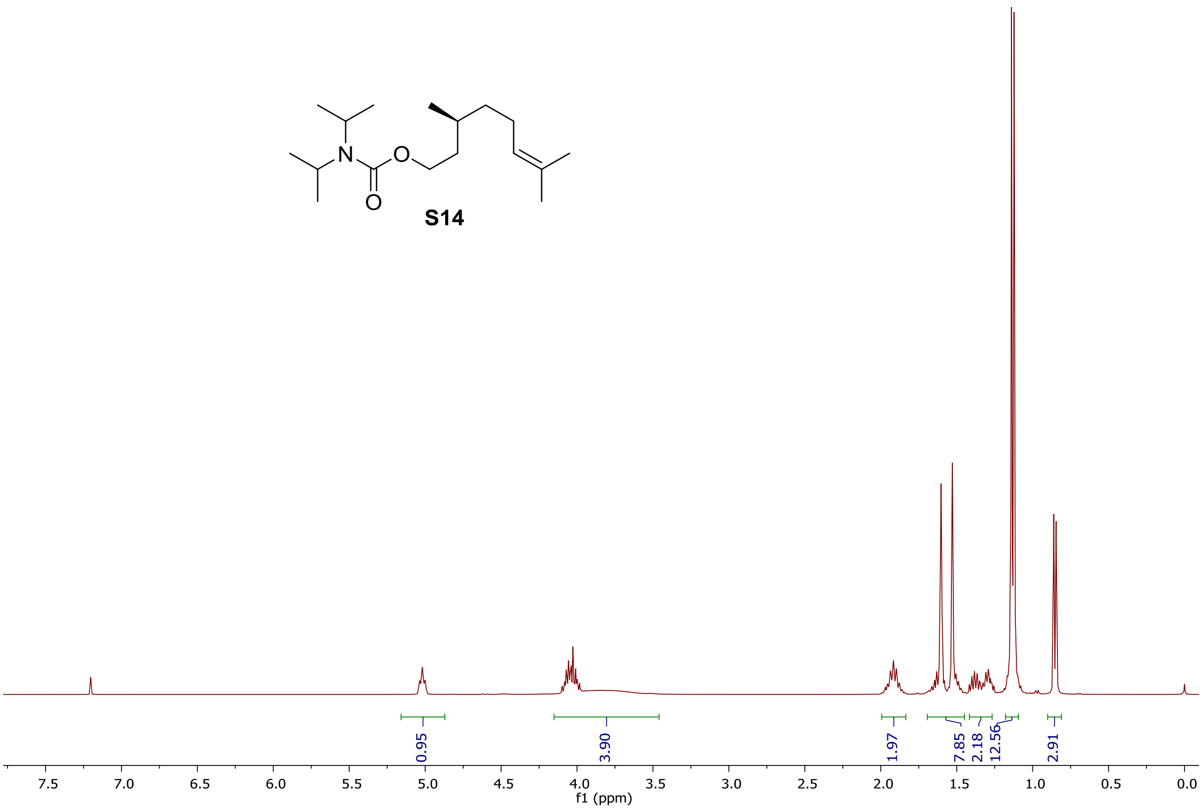
N
O
O
S14
0.95
3.90
1.97
7.85
2.18
12.56
2.91
7.5
7.0
6.5
6.0
5.5
5.0
4.5
4.0
3.5
3.0
2.5
2.0
1.5
1.0
0.5
0.0
f1 (ppm)

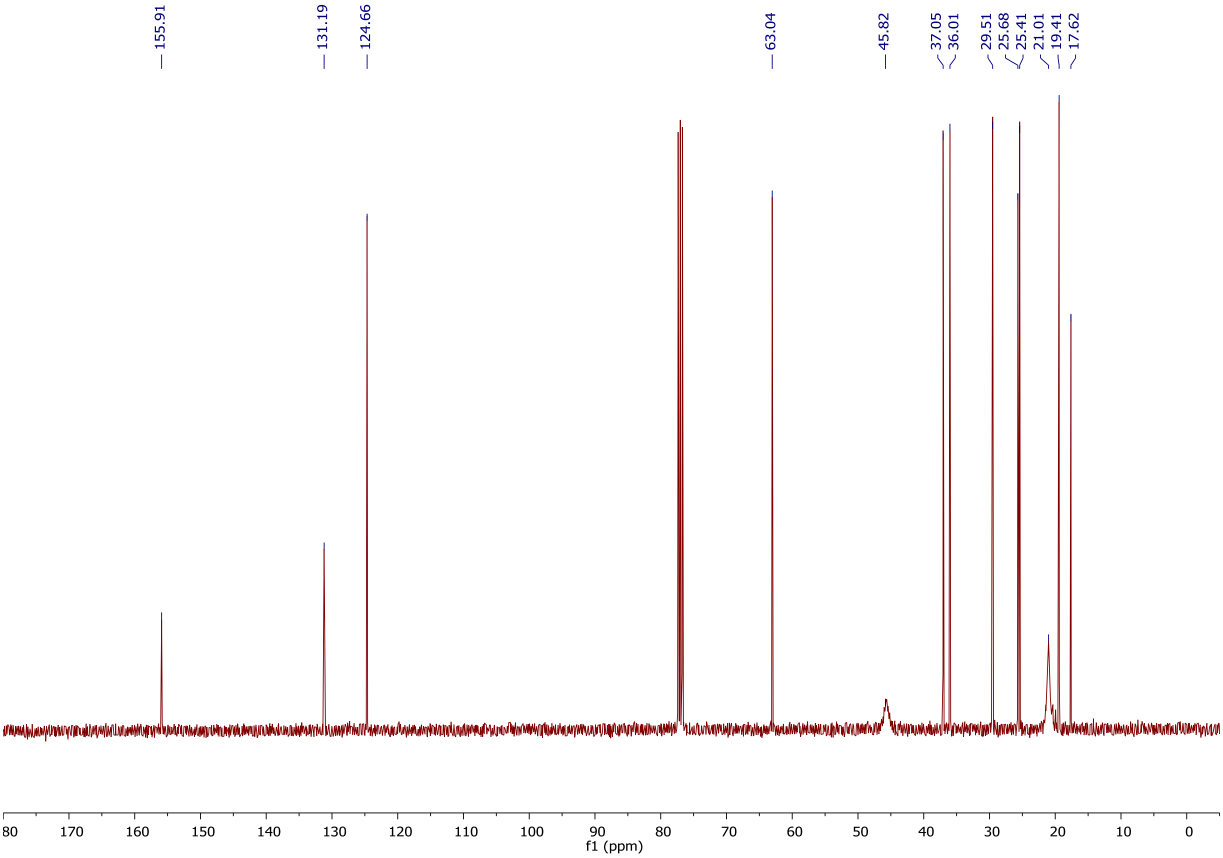
155.91
131.19
124.66
63.04
45.82
37.05
36.01
29.51
25.68
25.41
21.01
19.41
17.62
80
170
160
150
140
130
120
110
100
90
80
70
60
50
40
30
20
10
0
f1 (ppm)

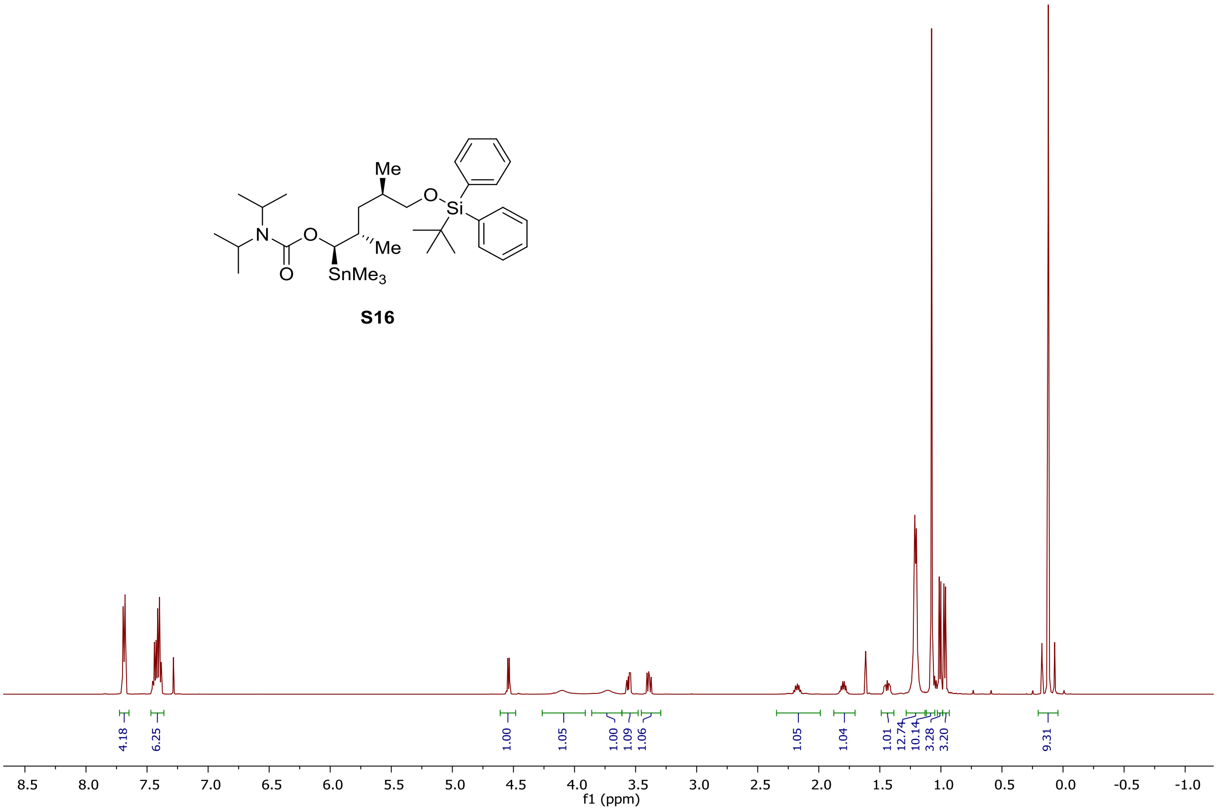
Me
O
Si
N
O
O
Me
SnMe3
S16
4.18
6.25
1.00
1.05
1.00
1.09
1.06
1.05
1.04
1.01
12.74
10.14
3.28
3.20
9.31
f1 (ppm)

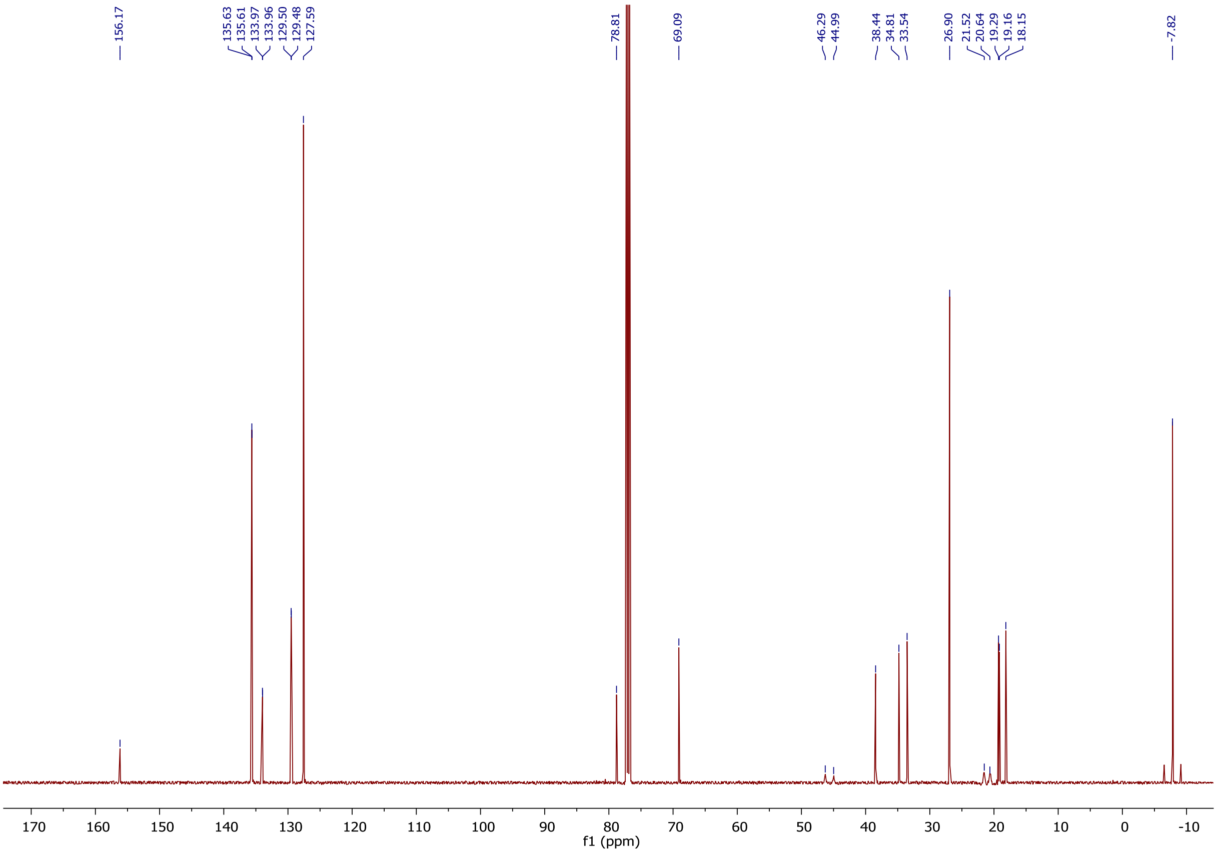
156.17
135.63
135.61
133.97
133.96
129.50
129.48
127.59
78.81
69.09
46.29
44.99
38.44
34.81
33.54
26.90
21.52
20.64
19.29
19.16
18.15
-7.82
f1 (ppm)

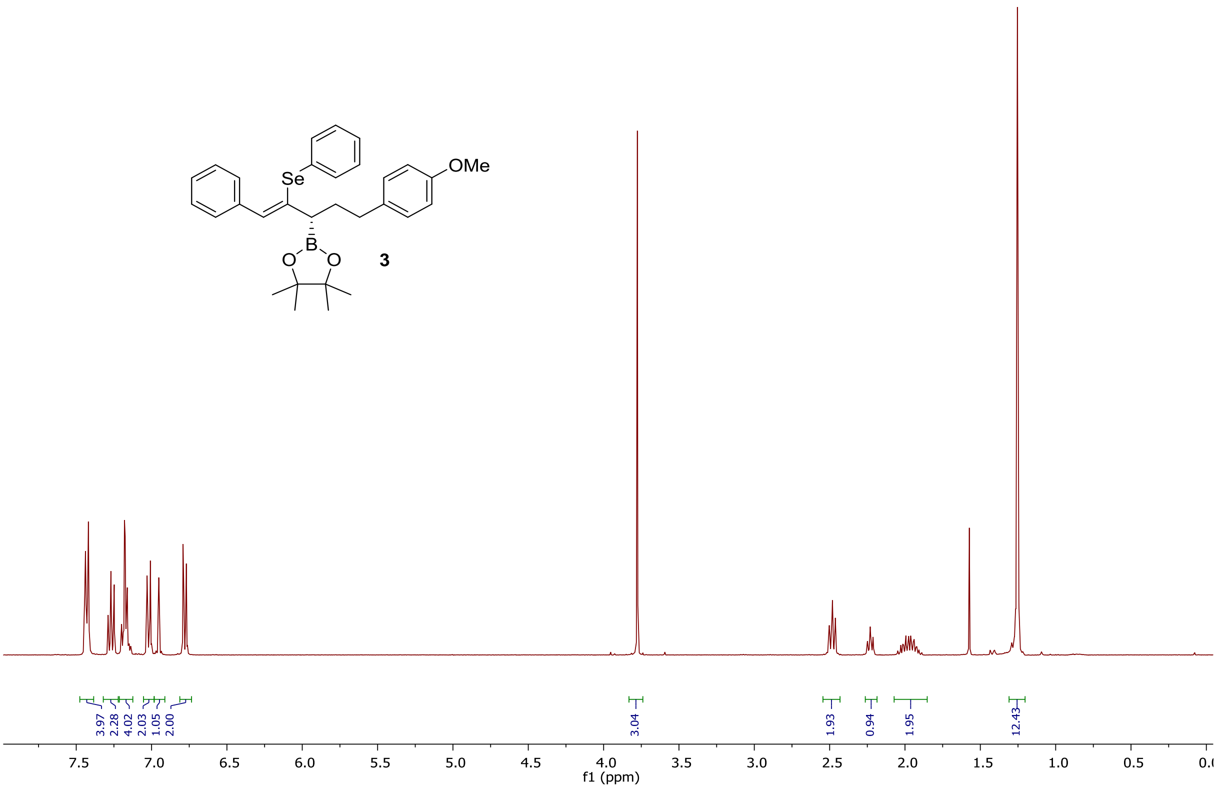
Se
OMe
B
O
O
3
3.97
2.28
4.02
2.03
1.05
2.00
3.04
1.93
0.94
1.95
12.43
7.5 7.0 6.5 6.0 5.5 5.0 4.5 4.0 3.5 3.0 2.5 2.0 1.5 1.0 0.5
f1 (ppm)

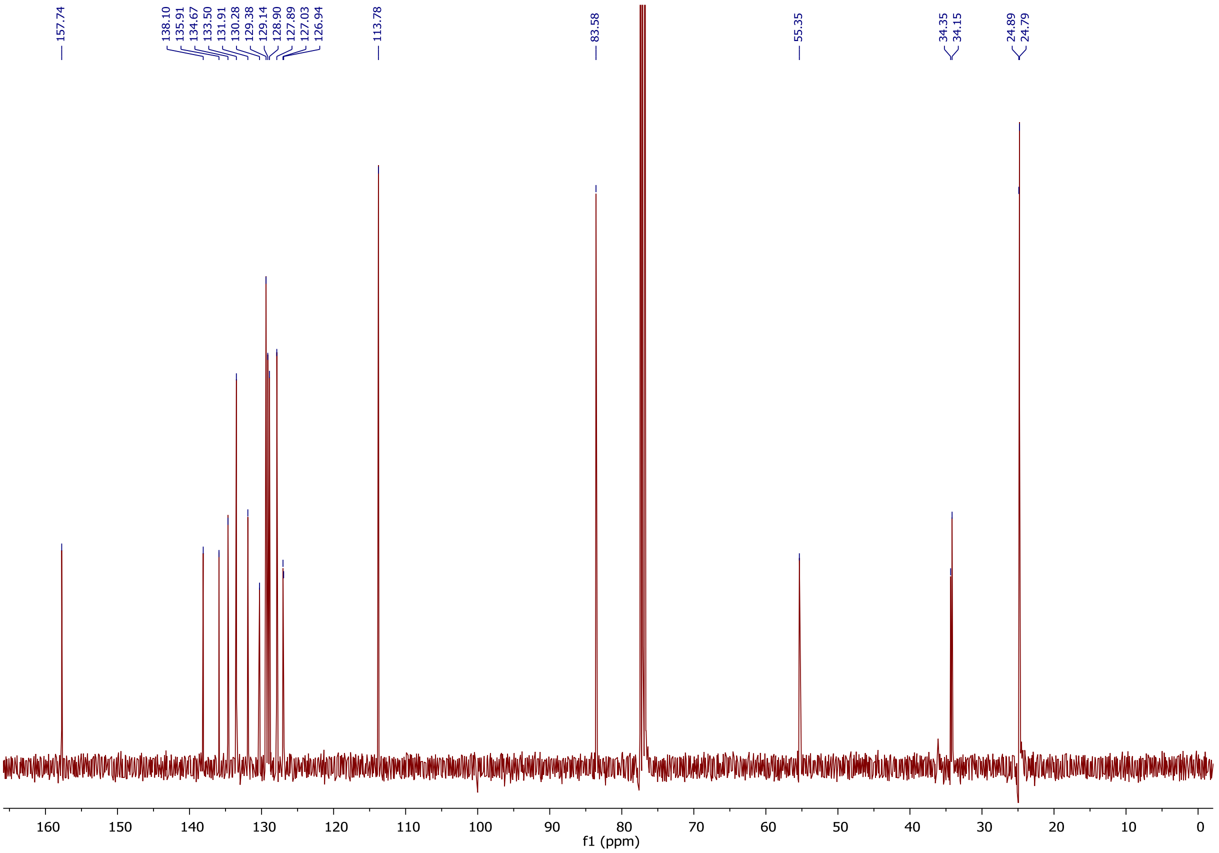
157.74
138.10
135.91
134.67
133.50
131.91
130.28
129.38
129.14
128.90
127.89
127.03
126.94
113.78
83.58
55.35
34.35
34.15
24.89
24.79
160 150 140 130 120 110 100 90 80 70 60 50 40 30 20 10 0
f1 (ppm)

NOESY data for 3 with key correlations highlighted

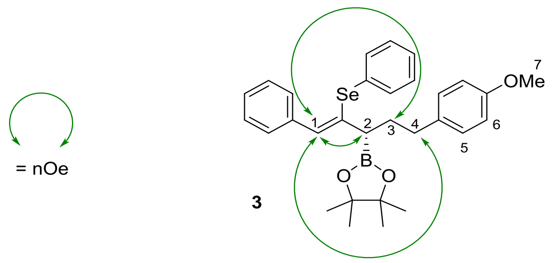

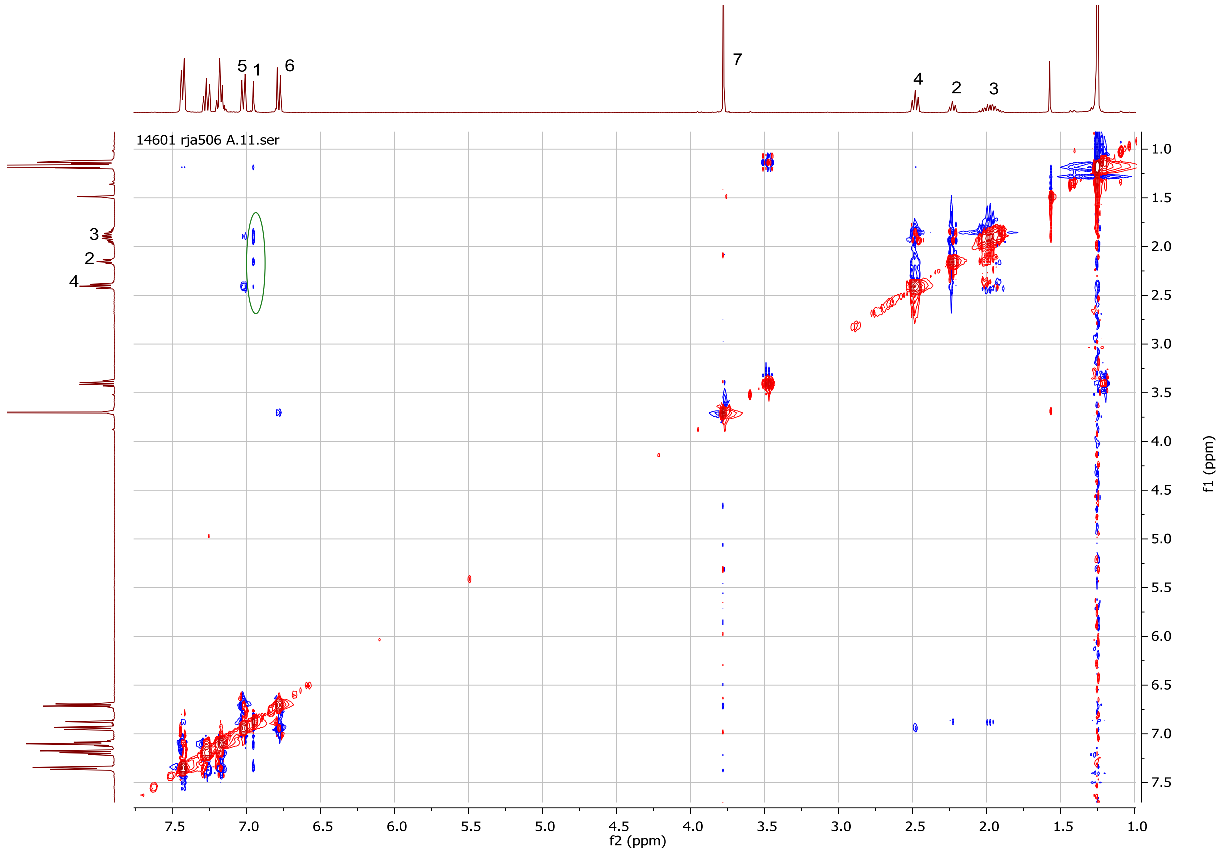

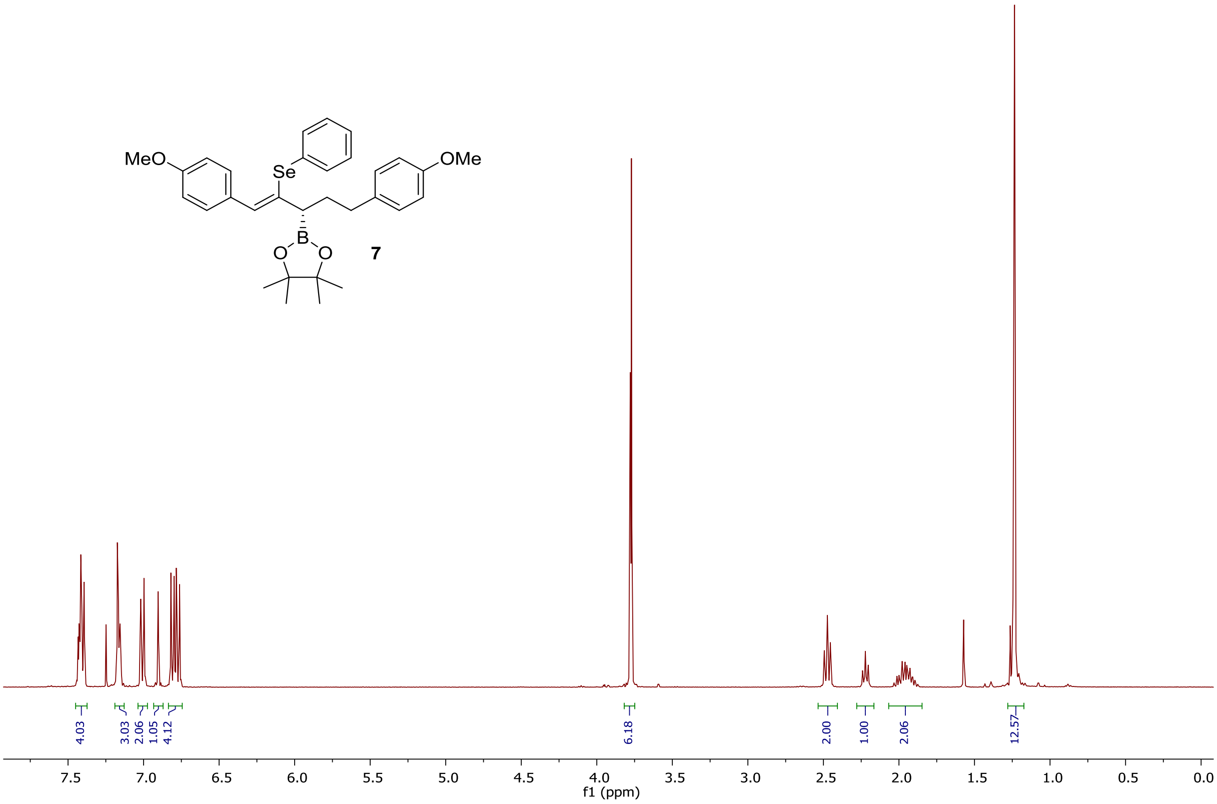
MeO
Se
OMe
B
O
O
7
4.03
3.03
2.06
1.05
4.12
6.18
2.00
1.00
2.06
12.57
7.5 7.0 6.5 6.0 5.5 5.0 4.5 4.0 3.5 3.0 2.5 2.0 1.5 1.0 0.5 0.0
f1 (ppm)

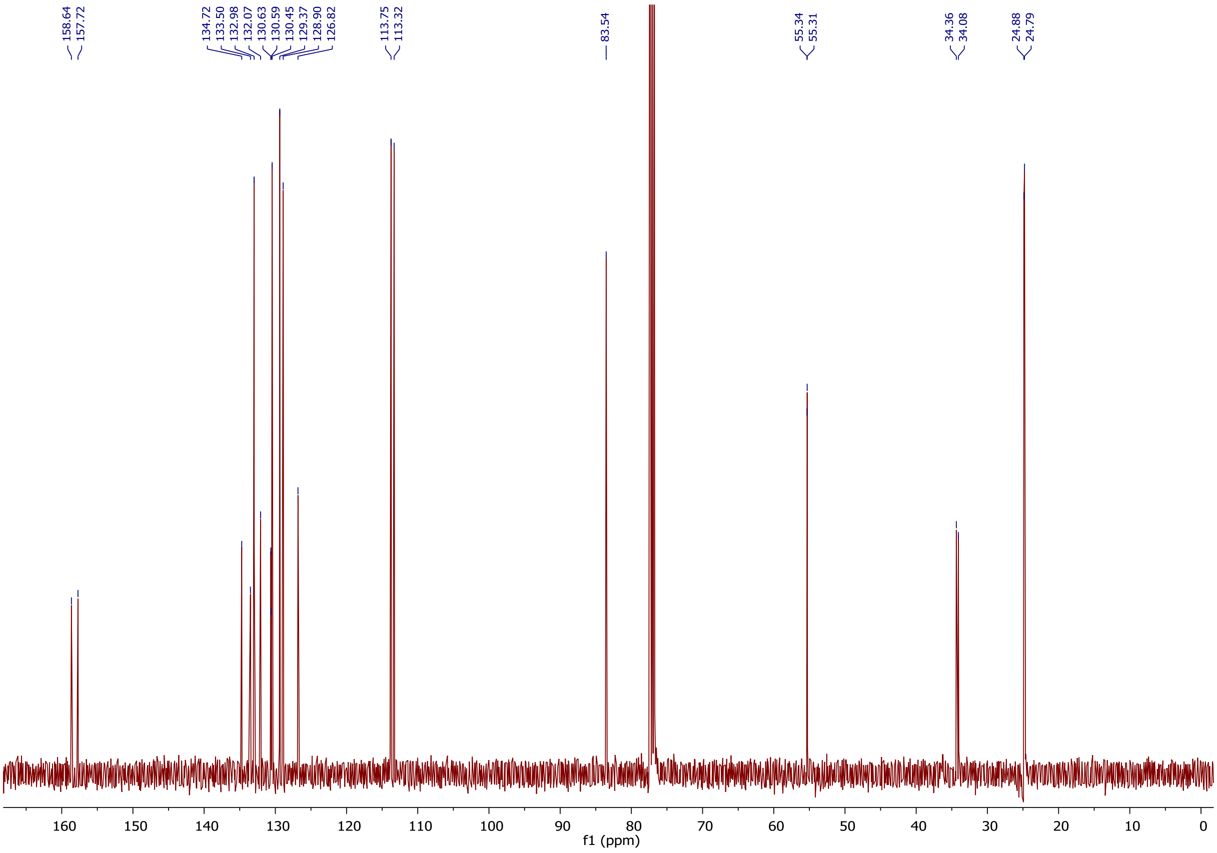
158.64
157.72
134.72
133.50
132.98
132.07
130.63
130.59
130.45
129.37
128.90
126.82
113.75
113.32
83.54
55.34
55.31
34.36
34.08
24.88
24.79
160 150 140 130 120 110 100 90 80 70 60 50 40 30 20 10 0
f1 (ppm)

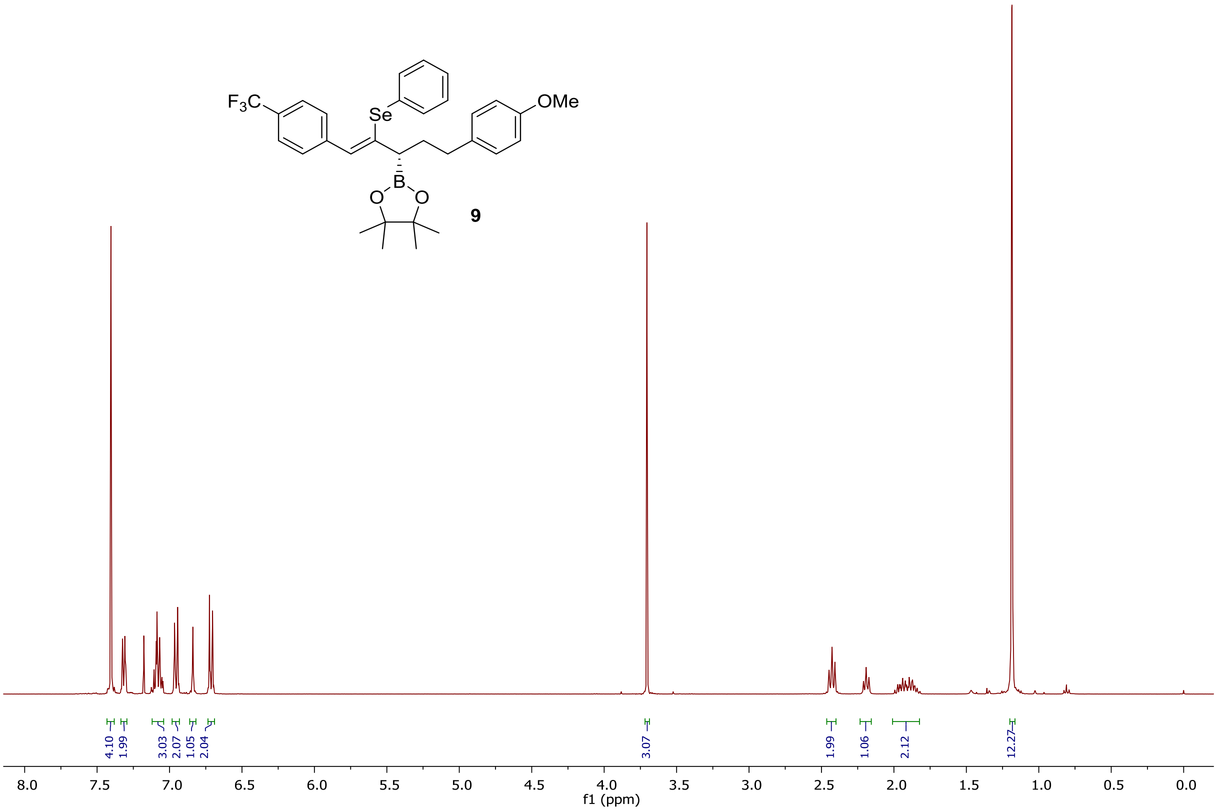
F3C
Se
OMe
B
O
O
9
4.10
1.99
3.03
2.07
1.05
2.04
3.07
1.99
1.06
2.12
12.27
8.0 7.5 7.0 6.5 6.0 5.5 5.0 4.5 4.0 3.5 3.0 2.5 2.0 1.5 1.0 0.5 0.0
f1 (ppm)

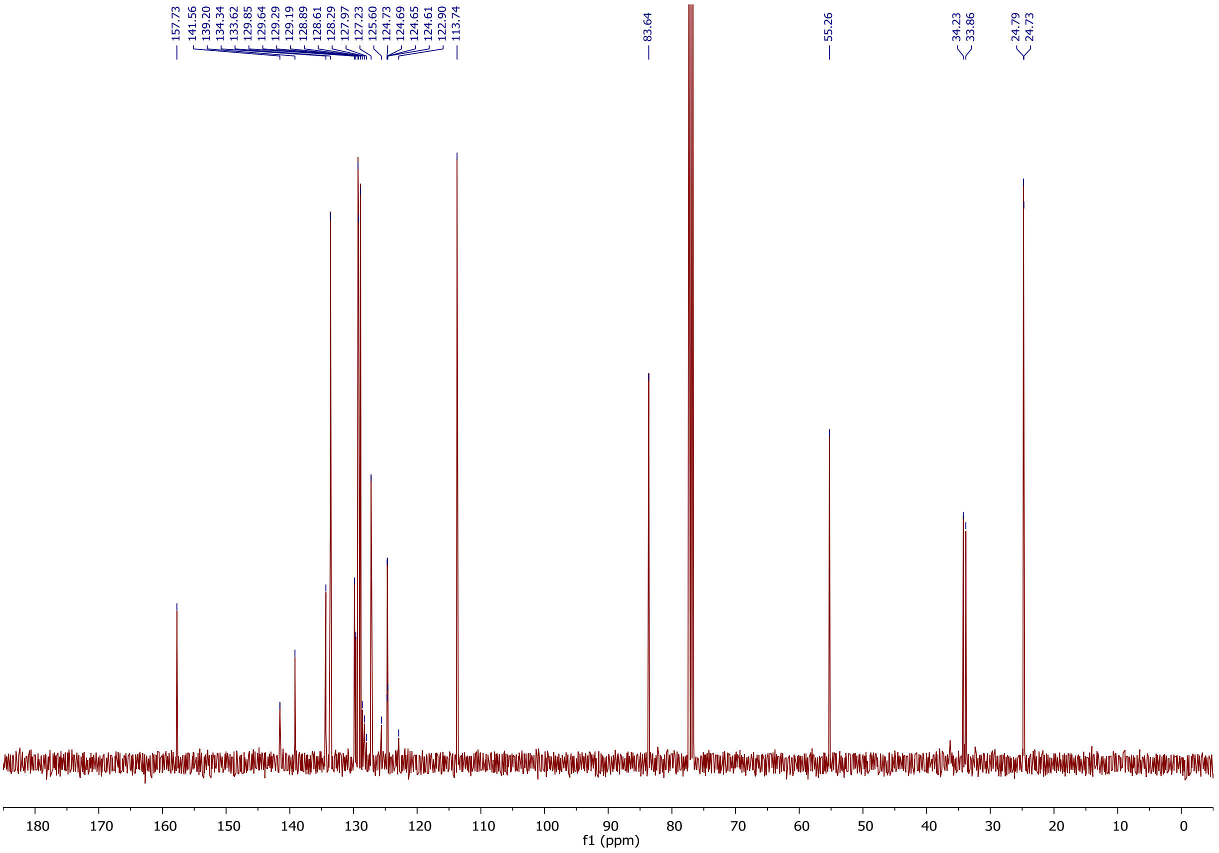
157.73
141.56
139.20
134.34
133.62
129.85
129.64
129.29
129.19
128.89
128.61
128.29
127.97
127.23
125.60
124.73
124.69
124.65
124.61
122.90
113.74
83.64
55.26
34.23
33.86
24.79
24.73
180 170 160 150 140 130 120 110 100 90 80 70 60 50 40 30 20 10 0
f1 (ppm)

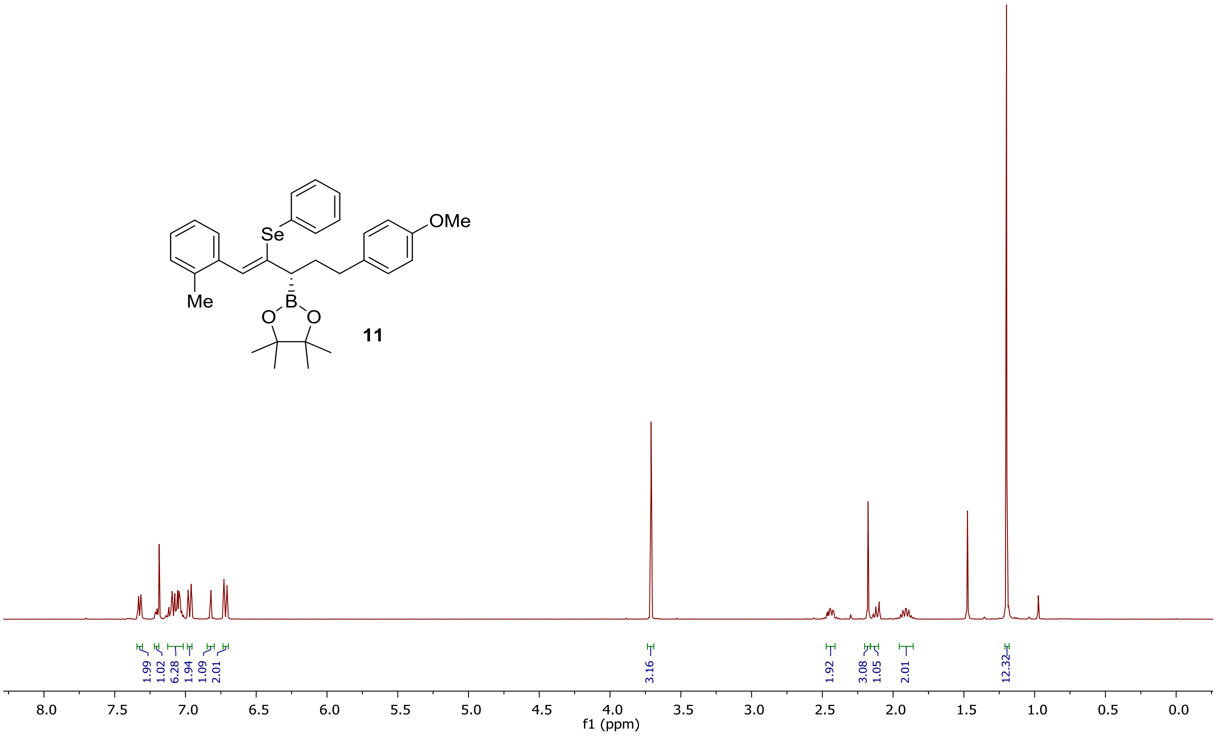
Se
OMe
Me
B
O
O

11

1.99
1.02
6.28
1.94
1.09
2.01
3.16
1.92
3.08
1.05
2.01
12.32
8.0
7.5
7.0
6.5
6.0
5.5
5.0
4.5
4.0
3.5
3.0
2.5
2.0
1.5
1.0
0.5
0.0
f1 (ppm)

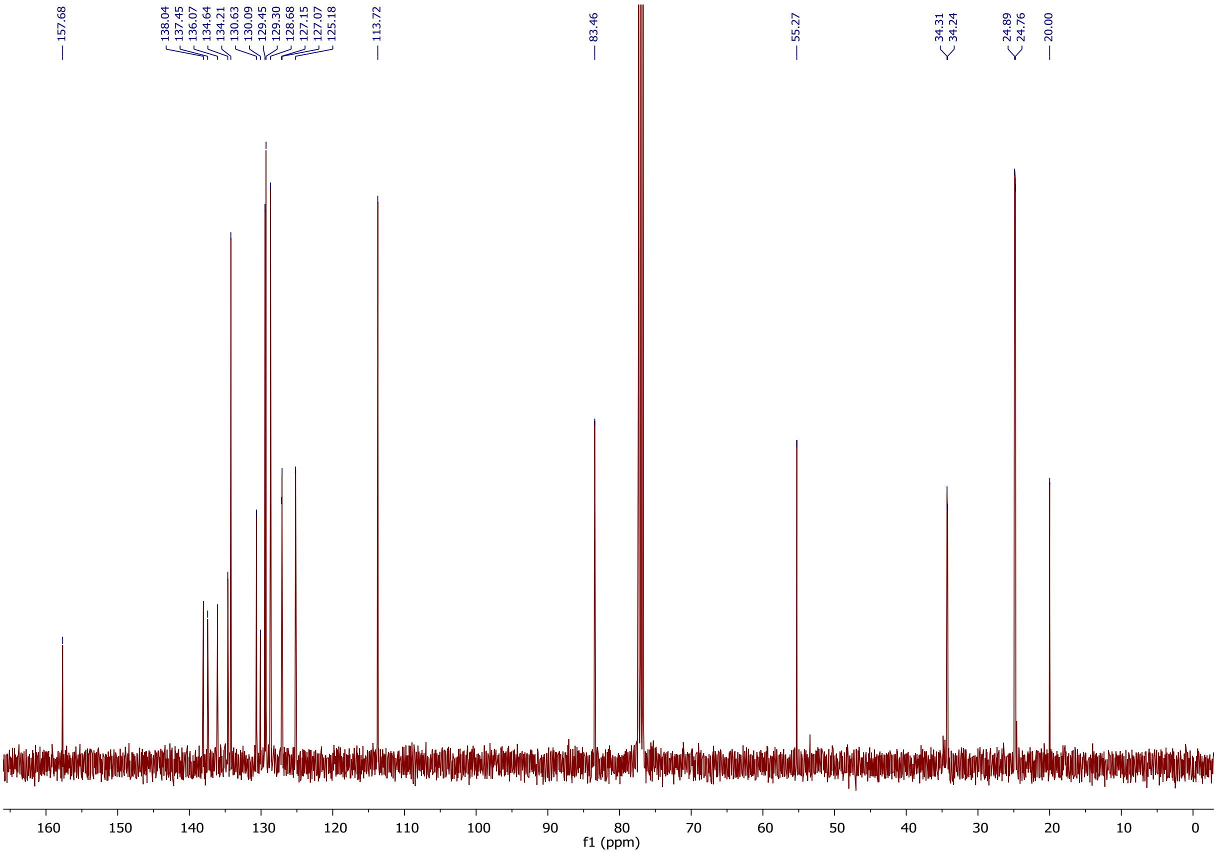
157.68
138.04
137.45
136.07
134.64
134.21
130.63
130.09
129.45
129.30
128.68
127.15
127.07
125.18
113.72
83.46
55.27
34.31
34.24
24.89
24.76
20.00
160
150
140
130
120
110
100
90
80
70
60
50
40
30
20
10
0
f1 (ppm)

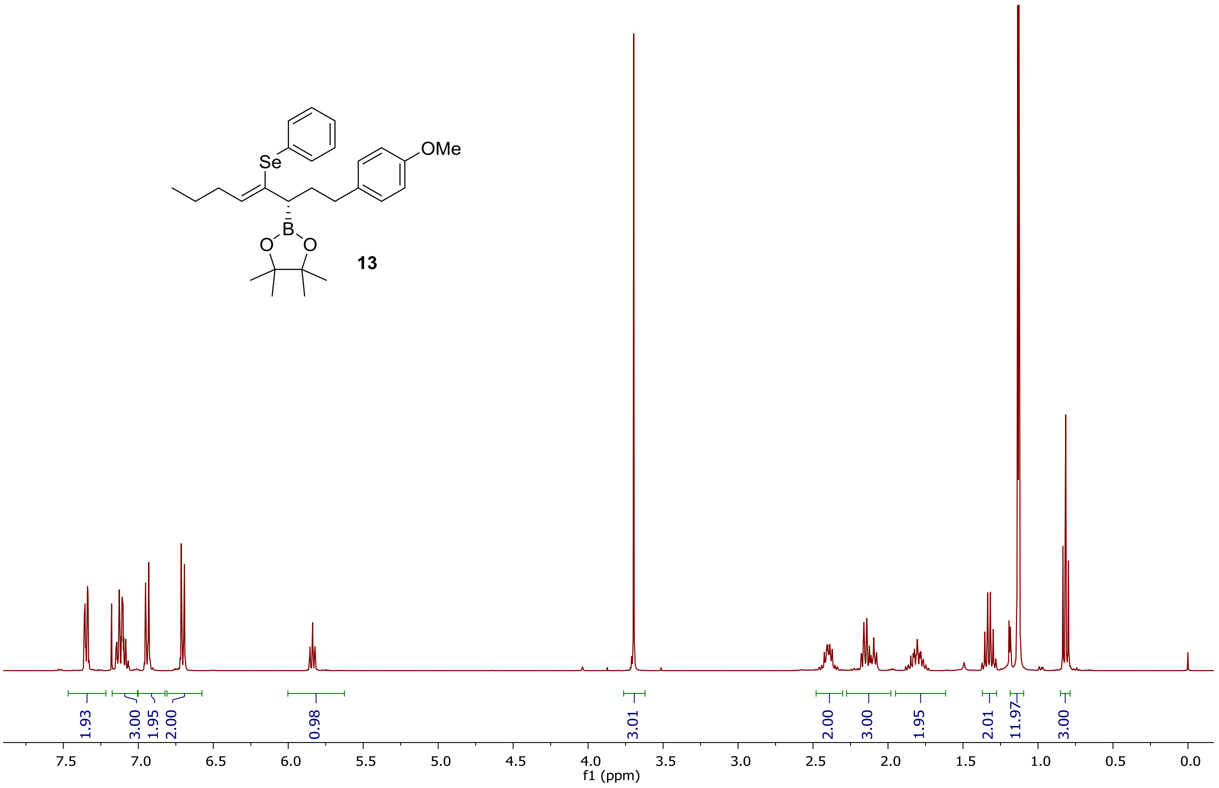
Se
OMe
B
O
O
13
1.93
3.00
1.95
2.00
0.98
3.01
2.00
3.00
1.95
2.01
11.97
3.00
7.5 7.0 6.5 6.0 5.5 5.0 4.5 4.0 3.5 3.0 2.5 2.0 1.5 1.0 0.5 0.0
f1 (ppm)

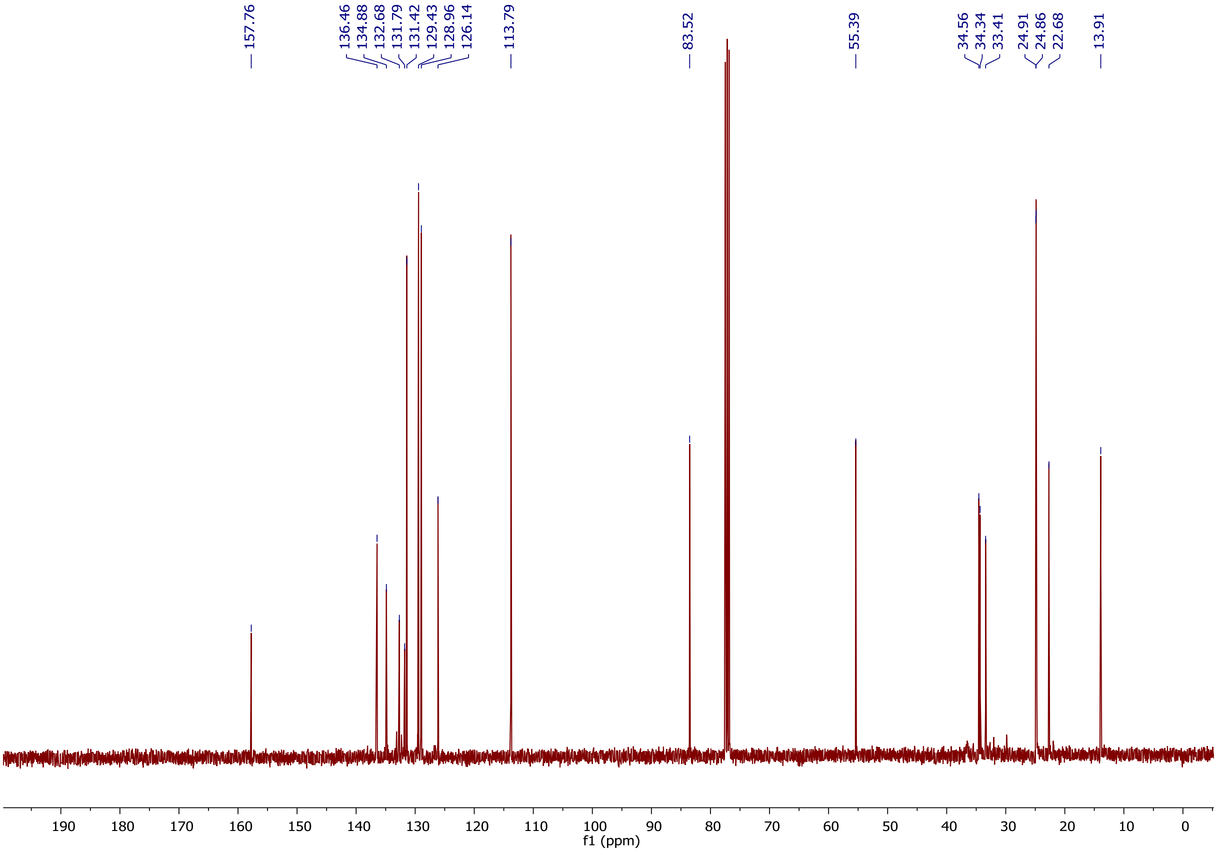
157.76
136.46
134.88
132.68
131.79
131.42
129.43
128.96
126.14
113.79
83.52
55.39
34.56
34.34
33.41
24.91
24.86
22.68
13.91
190 180 170 160 150 140 130 120 110 100 90 80 70 60 50 40 30 20 10 0
f1 (ppm)

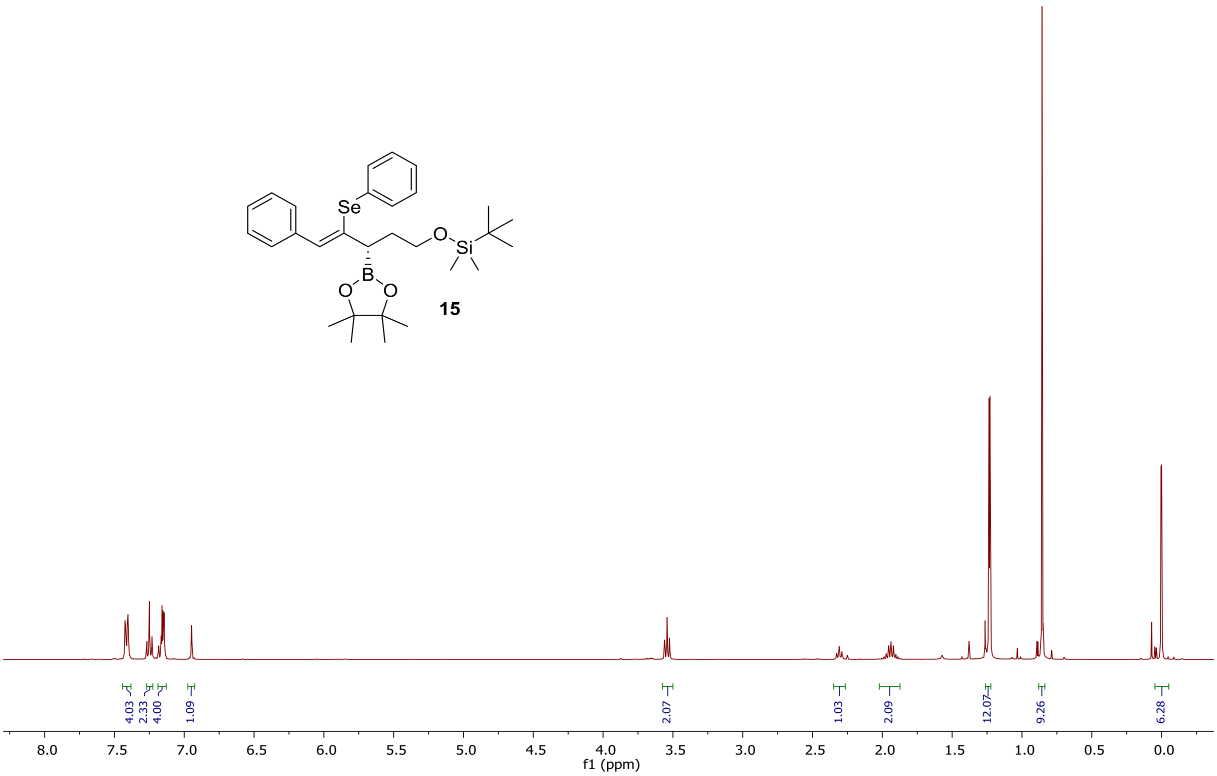
Se
O
Si
B
O
O
15
4.03
2.33
4.00
1.09
2.07
1.03
2.09
12.07
9.26
6.28
8.0 7.5 7.0 6.5 6.0 5.5 5.0 4.5 4.0 3.5 3.0 2.5 2.0 1.5 1.0 0.5 0.0
f1 (ppm)

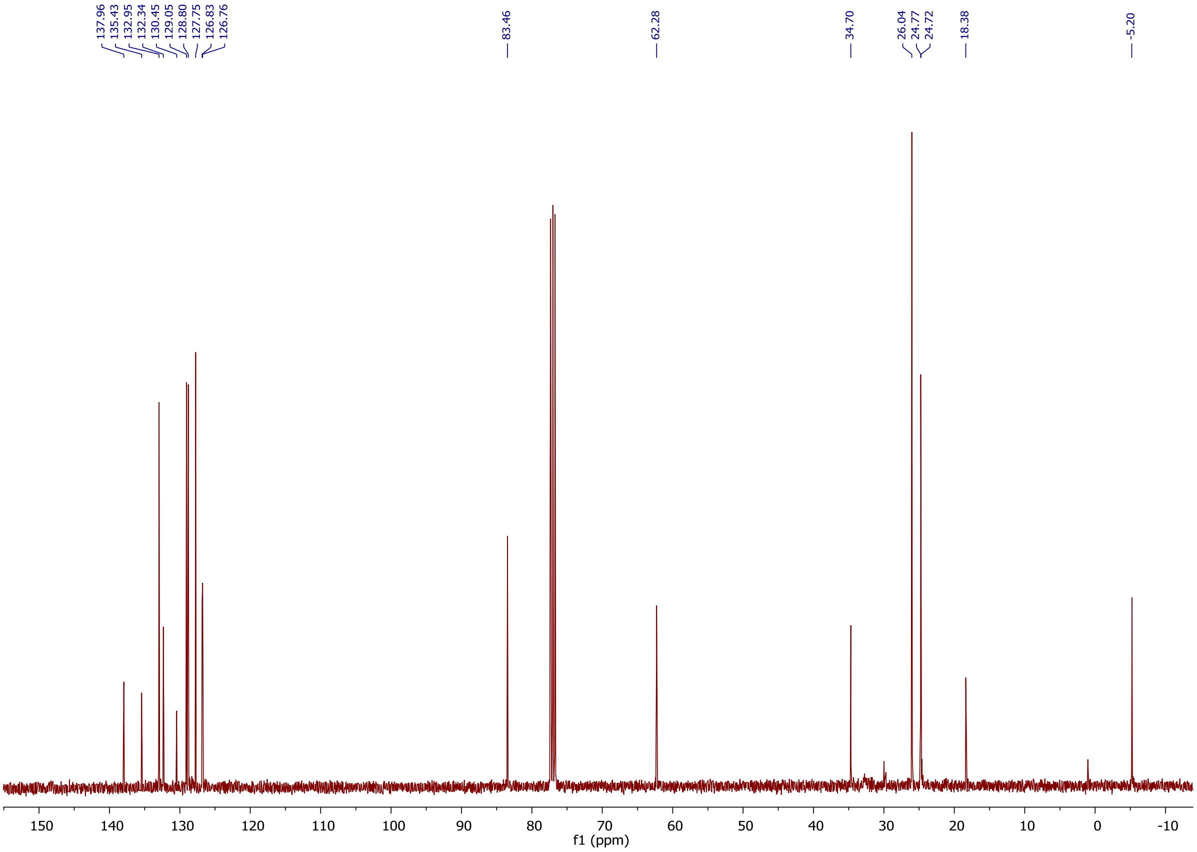
137.96
135.43
132.95
132.34
130.45
129.05
128.80
127.75
126.83
126.76
83.46
62.28
34.70
26.04
24.77
24.72
18.38
-5.20
150 140 130 120 110 100 90 80 70 60 50 40 30 20 10 0 -10
f1 (ppm)

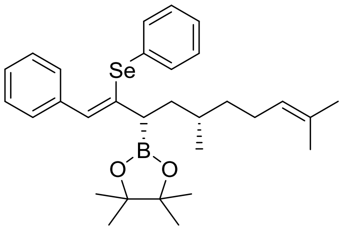
Se
B
O
O

18

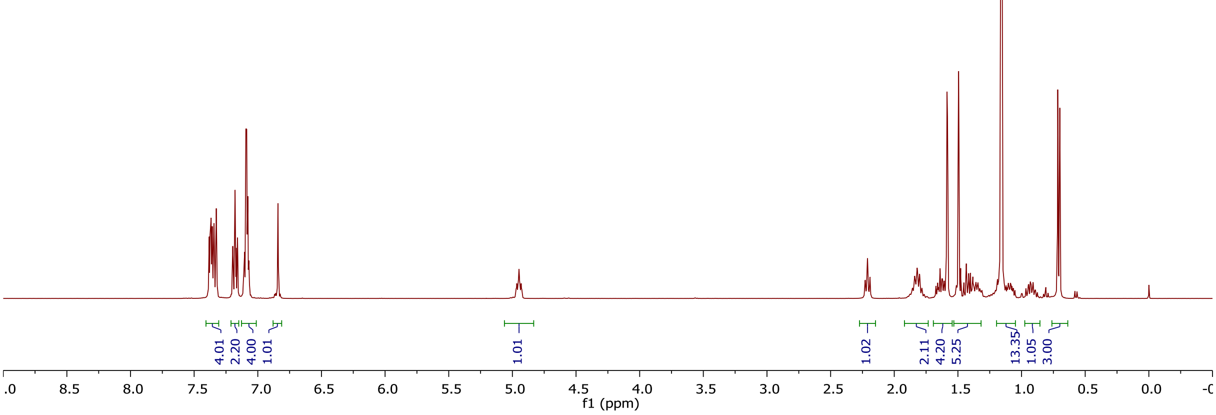
4.01
2.20
4.00
1.01
1.01
1.02
2.11
4.20
5.25
13.35
1.05
3.00
f1 (ppm)

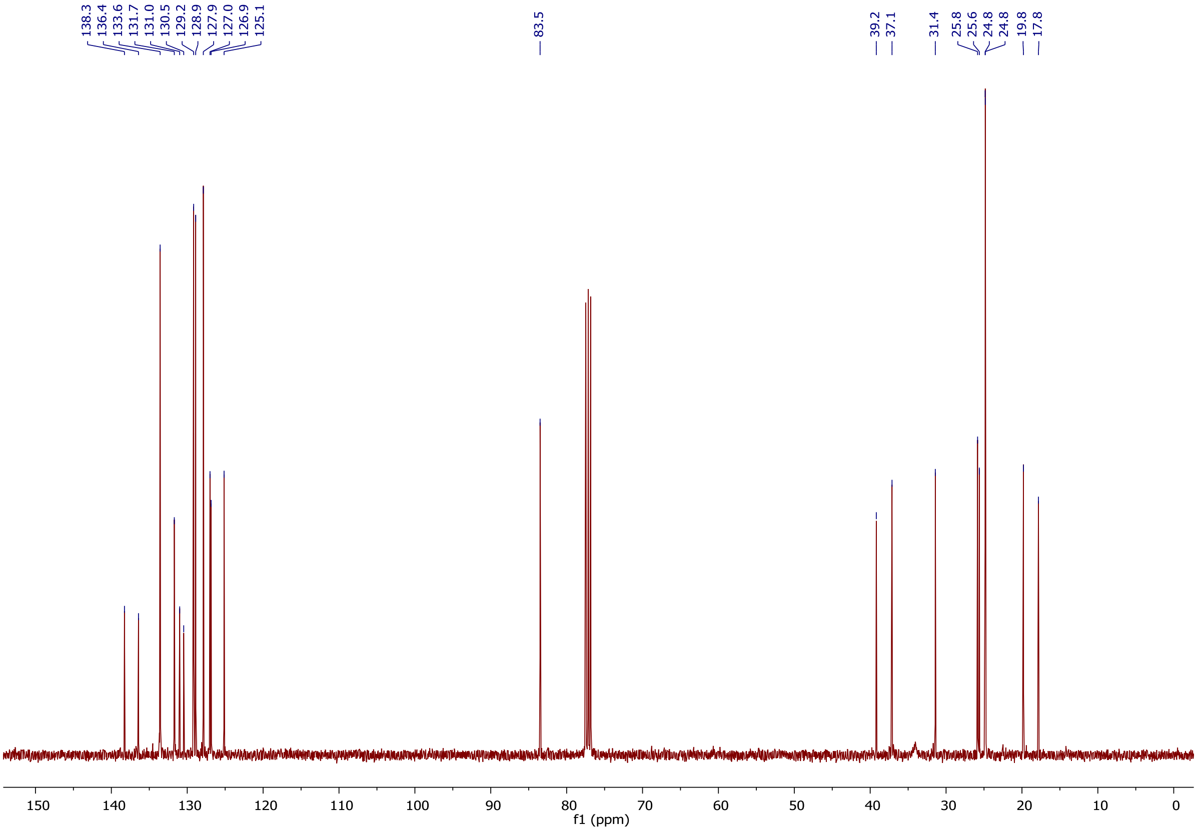
138.3
136.4
133.6
131.7
131.0
130.5
129.2
128.9
127.9
127.0
126.9
125.1
83.5
39.2
37.1
31.4
25.8
25.6
24.8
24.8
19.8
17.8
f1 (ppm)

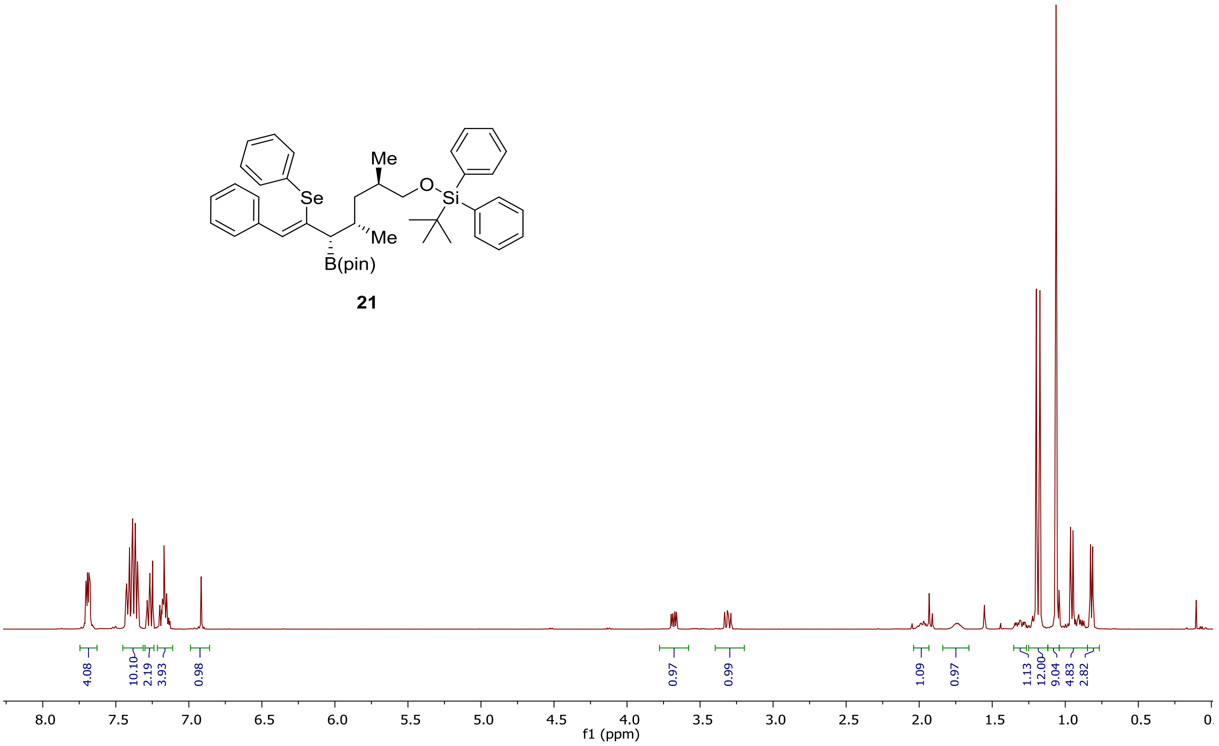
Se
Me
O
Si
Me
B(pin)
21
4.08
10.10
2.19
3.93
0.98
0.97
0.99
1.09
0.97
1.13
12.00
9.04
4.83
2.82
8.0 7.5 7.0 6.5 6.0 5.5 5.0 4.5 4.0 3.5 3.0 2.5 2.0 1.5 1.0 0.5
f1 (ppm)

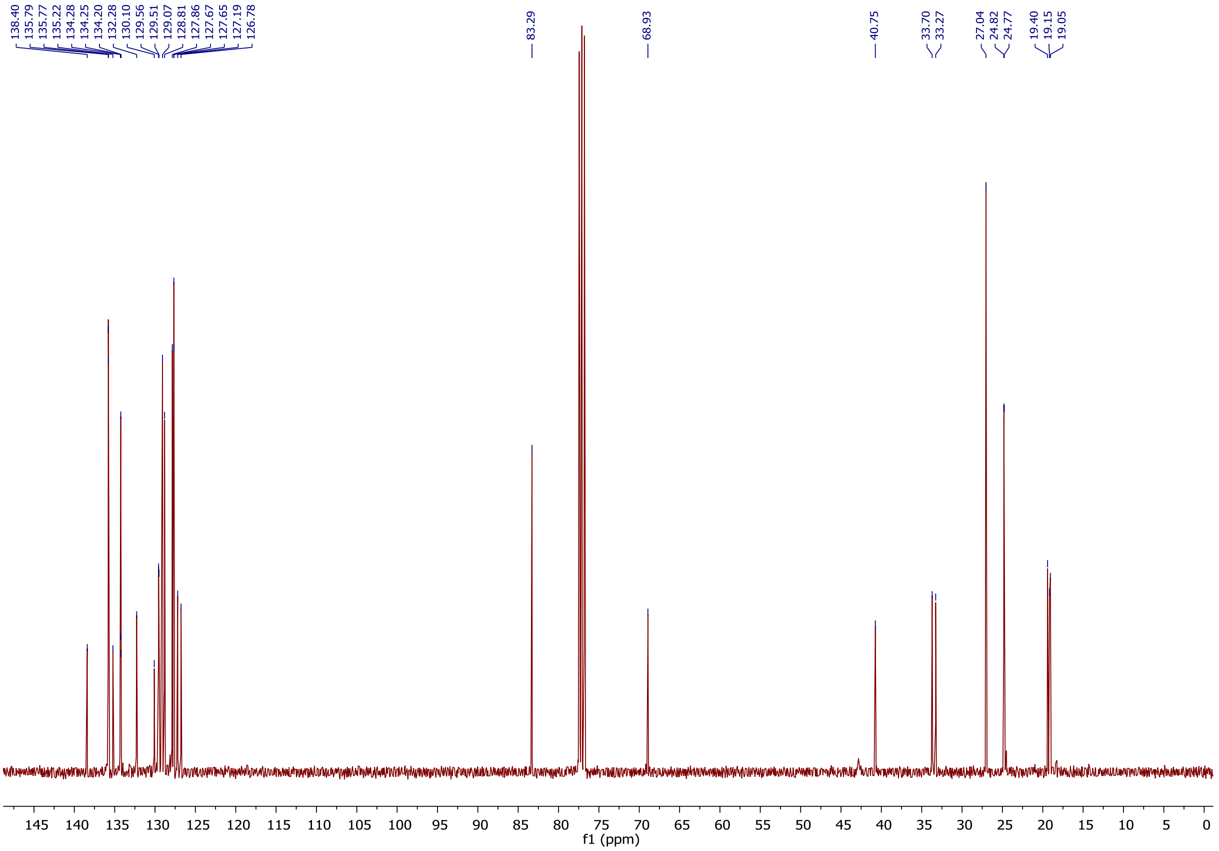
138.40
135.79
135.77
135.22
134.28
134.25
134.20
132.28
130.10
129.56
129.51
129.07
128.81
127.86
127.67
127.65
127.19
126.78
83.29
68.93
40.75
33.70
33.27
27.04
24.82
24.77
19.40
19.15
19.05
145 140 135 130 125 120 115 110 105 100 95 90 85 80 75 70 65 60 55 50 45 40 35 30 25 20 15 10 5 0
f1 (ppm)

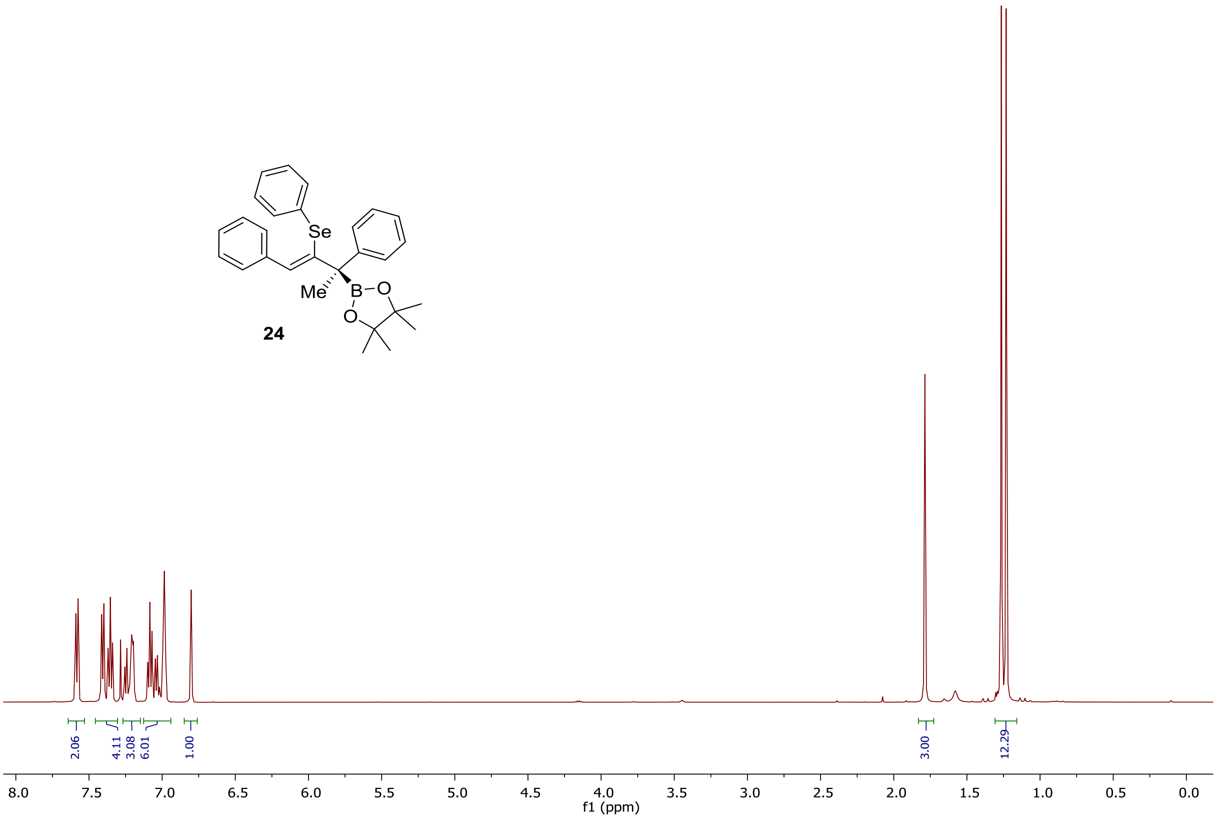
Se
Me
B
O
O
24
2.06
4.11
3.08
6.01
1.00
3.00
12.29
8.0 7.5 7.0 6.5 6.0 5.5 5.0 4.5 4.0 3.5 3.0 2.5 2.0 1.5 1.0 0.5 0.0
f1 (ppm)

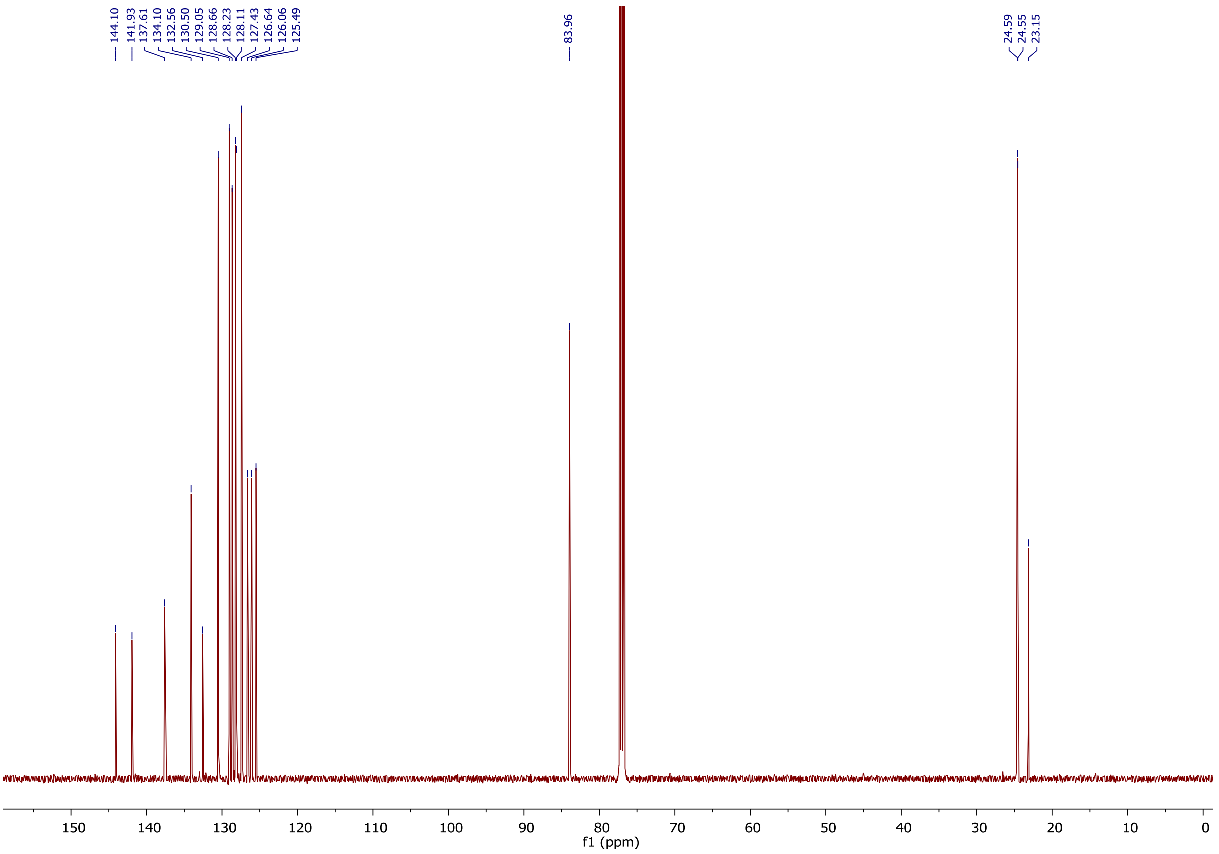
144.10
141.93
137.61
134.10
132.56
130.50
129.05
128.66
128.23
128.11
127.43
126.64
126.06
125.49
83.96
24.59
24.55
23.15
150 140 130 120 110 100 90 80 70 60 50 40 30 20 10 0
f1 (ppm)

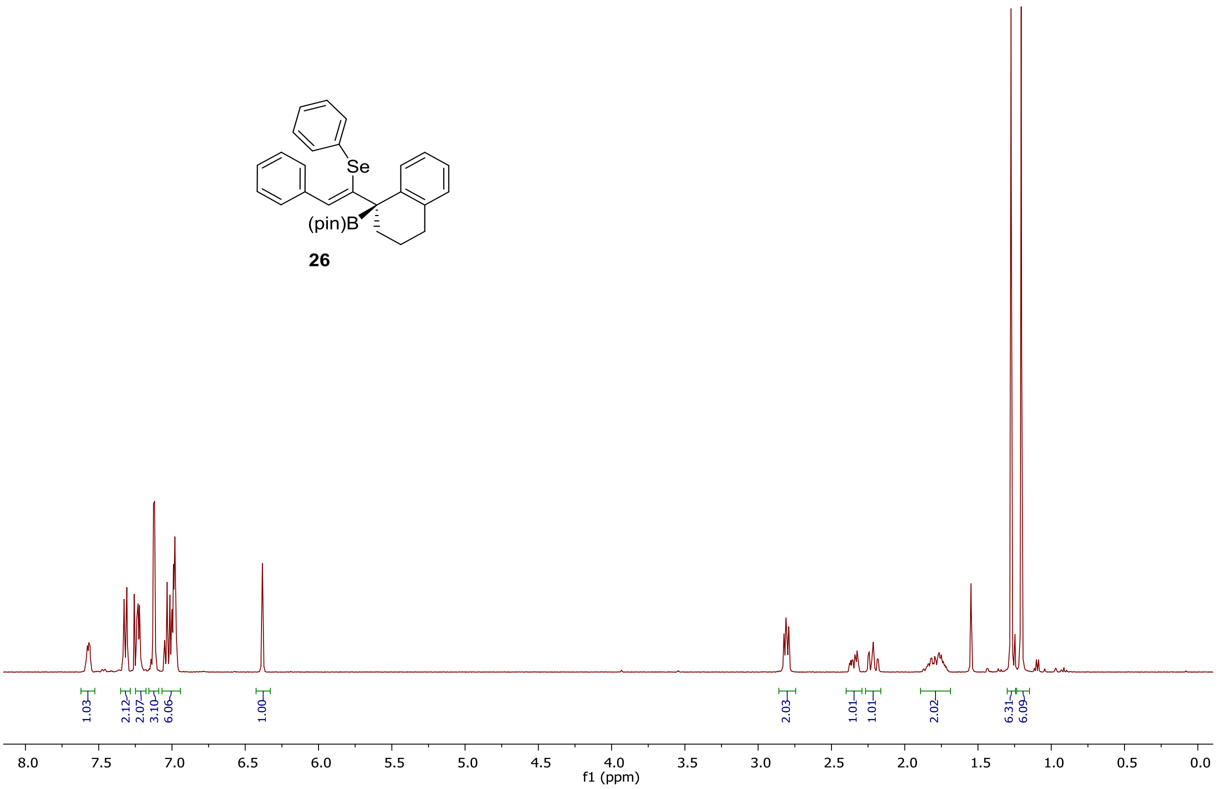
Se
(pin)B
26
1.03
2.12
2.07
3.10
6.06
1.00
2.03
1.01
1.01
2.02
6.31
6.09
8.0 7.5 7.0 6.5 6.0 5.5 5.0 4.5 4.0 3.5 3.0 2.5 2.0 1.5 1.0 0.5 0.0
f1 (ppm)

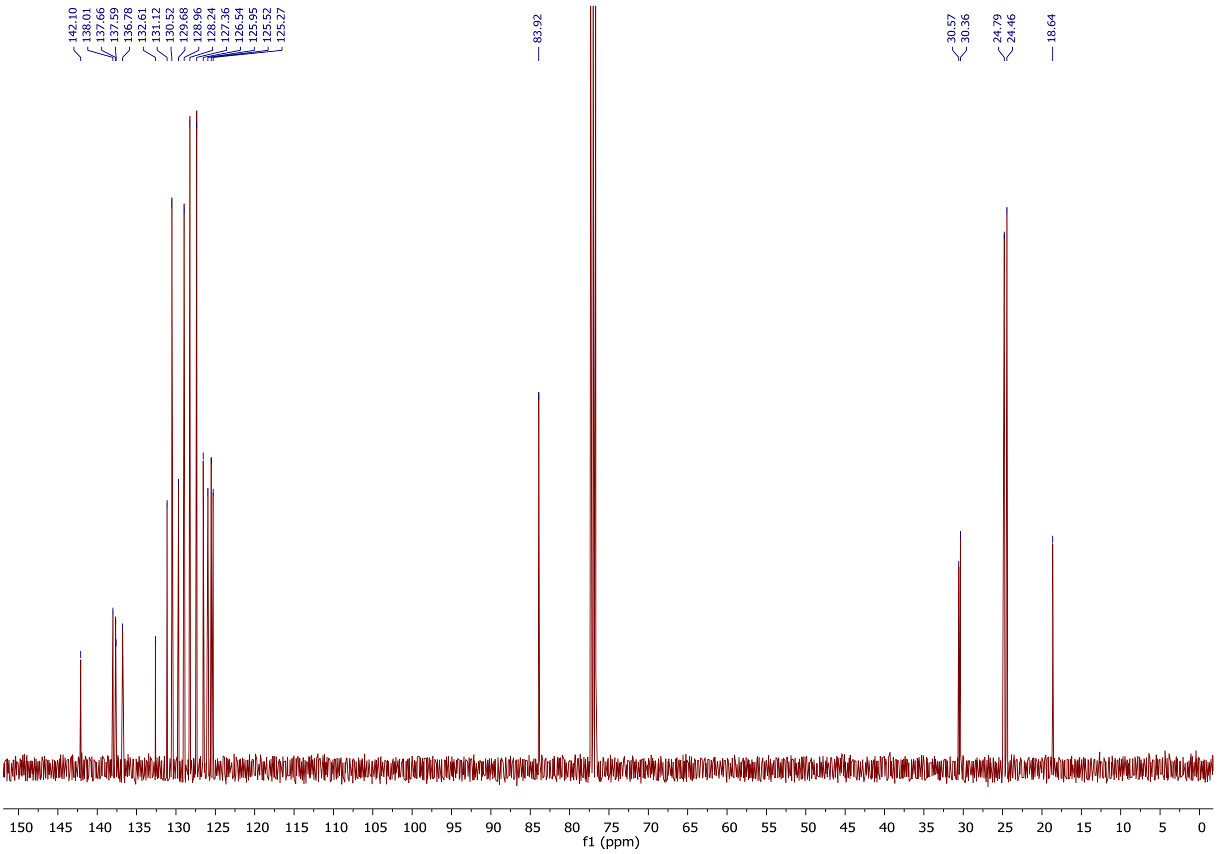
142.10
138.01
137.66
137.59
136.78
132.61
131.12
130.52
129.68
128.96
128.24
127.36
126.54
125.95
125.52
125.27
83.92
30.57
30.36
24.79
24.46
18.64
150 145 140 135 130 125 120 115 110 105 100 95 90 85 80 75 70 65 60 55 50 45 40 35 30 25 20 15 10 5 0
f1 (ppm)

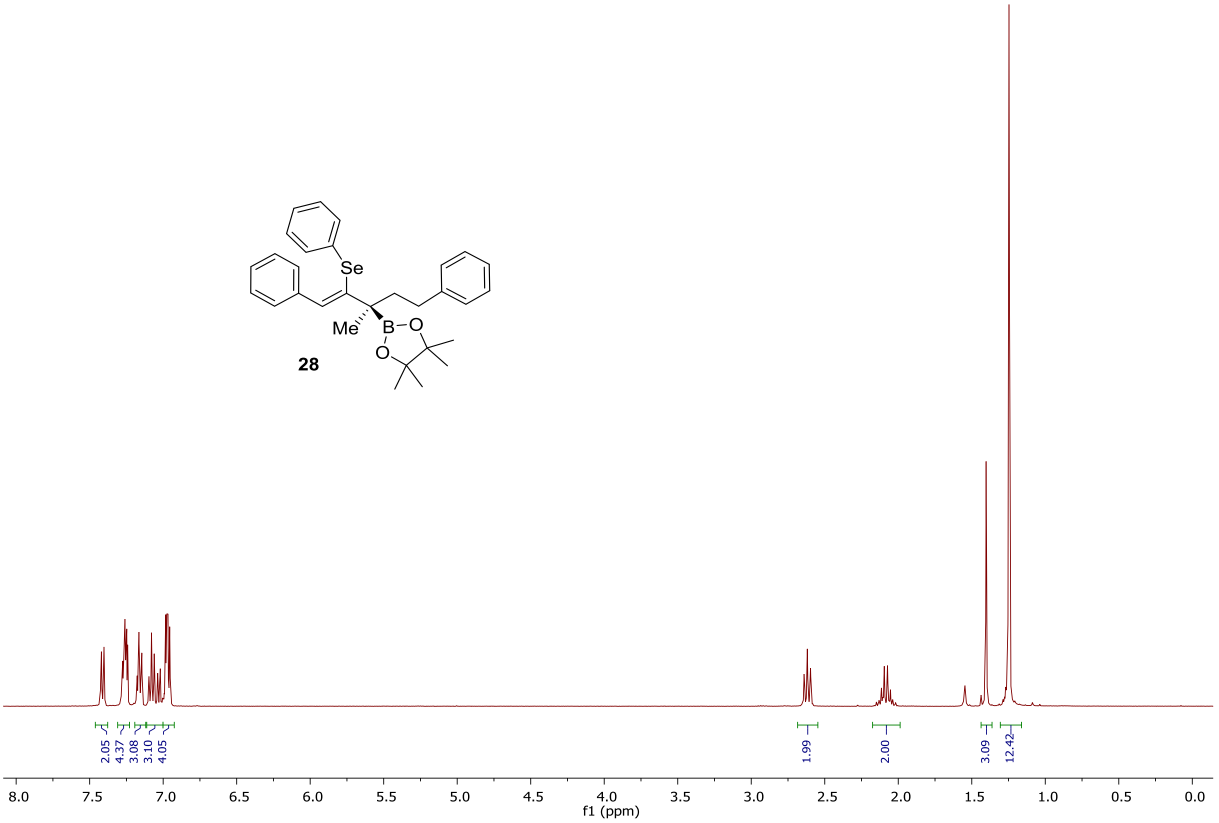
Se
Me
B
O
O
28
2.05
4.37
3.08
3.10
4.05
1.99
2.00
3.09
12.42
8.0 7.5 7.0 6.5 6.0 5.5 5.0 4.5 4.0 3.5 3.0 2.5 2.0 1.5 1.0 0.5 0.0
f1 (ppm)

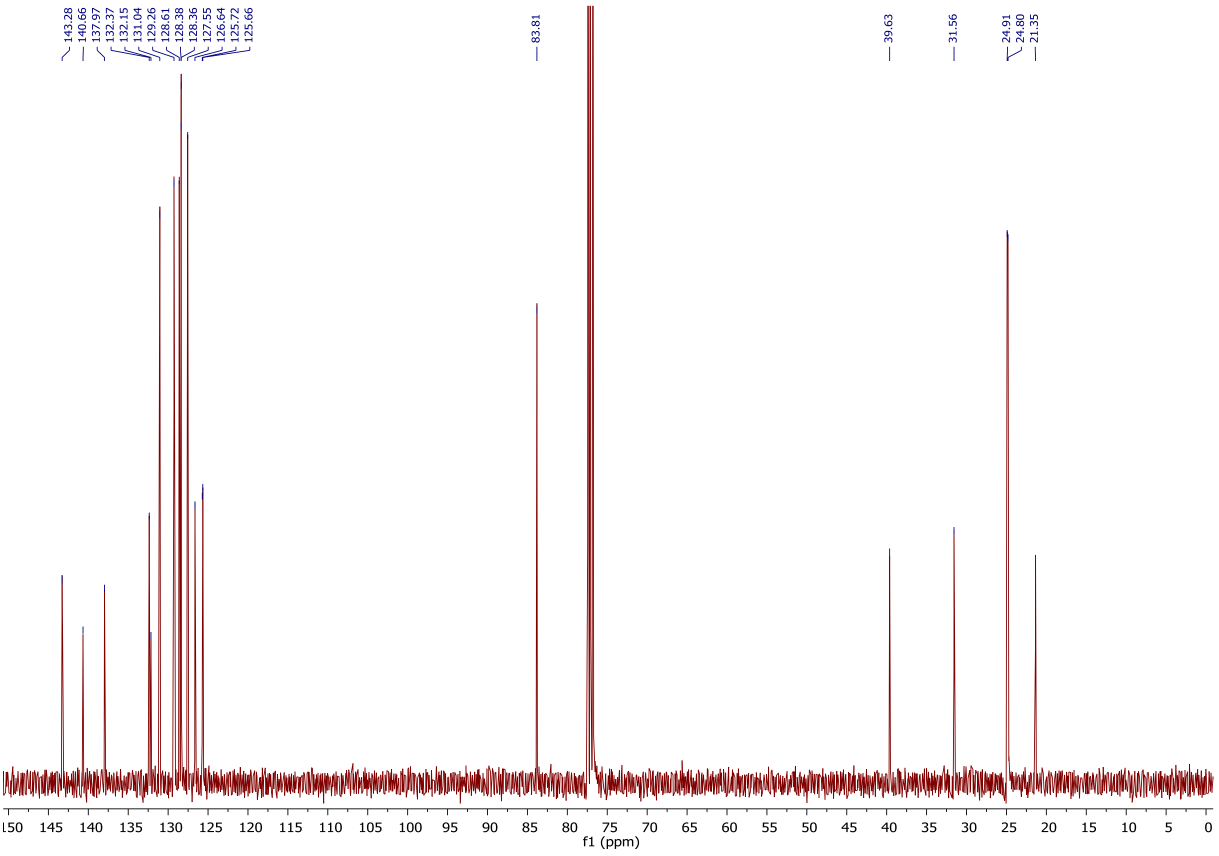
143.28
140.66
137.97
132.37
132.15
131.04
129.26
128.61
128.38
128.36
127.55
126.64
125.72
125.66
83.81
39.63
31.56
24.91
24.80
21.35
150 145 140 135 130 125 120 115 110 105 100 95 90 85 80 75 70 65 60 55 50 45 40 35 30 25 20 15 10 5 0
f1 (ppm)

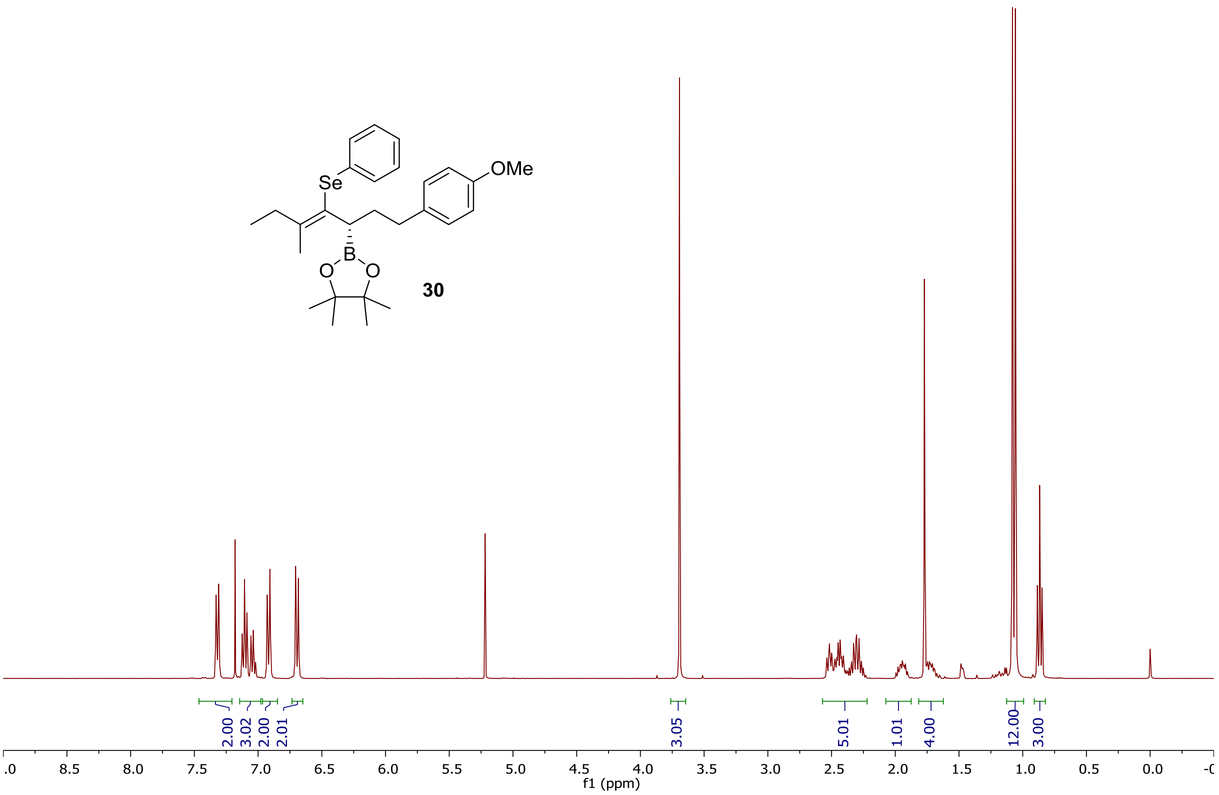

Se
OMe
B
O
O
30
2.00
3.02
2.00
2.01
3.05
5.01
1.01
4.00
12.00
3.00
f1 (ppm)

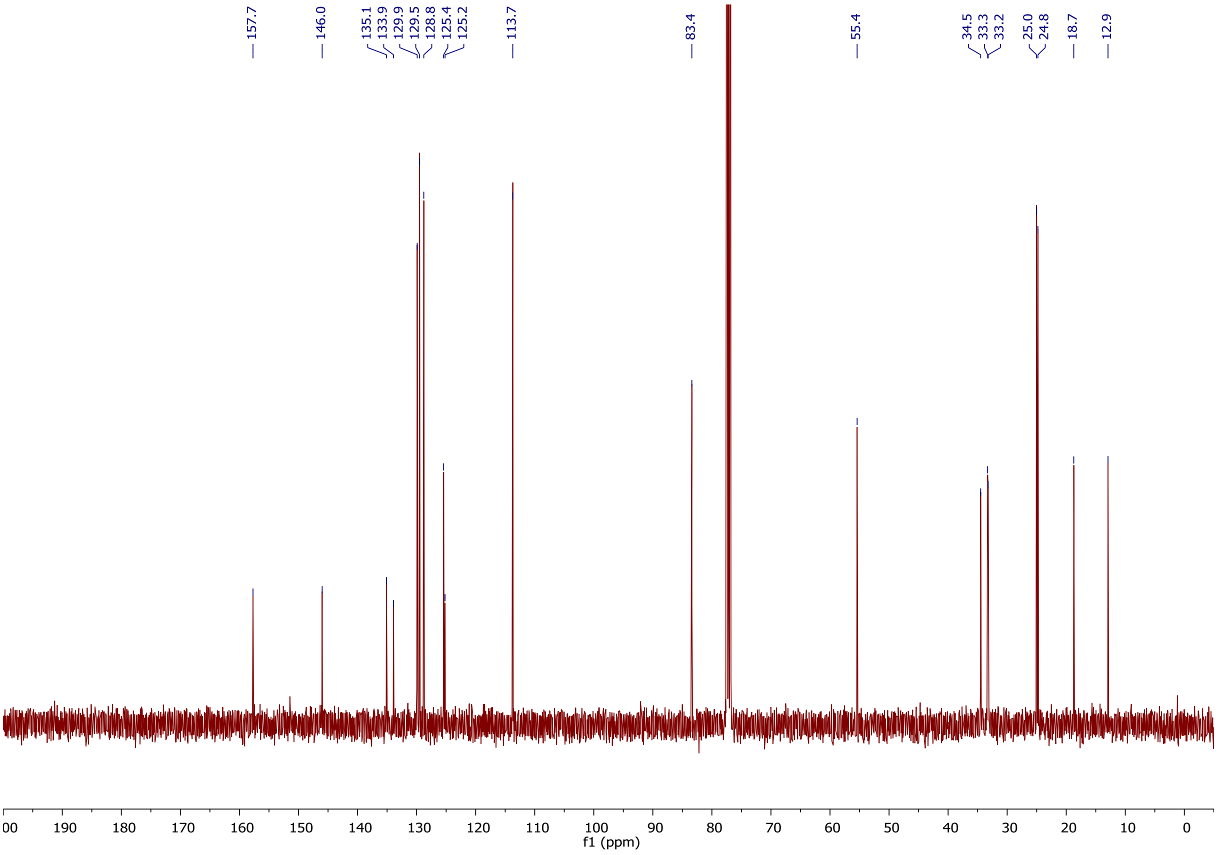

157.7
146.0
135.1
133.9
129.9
129.5
128.8
125.4
125.2
113.7
83.4
55.4
34.5
33.3
33.2
25.0
24.8
18.7
12.9
f1 (ppm)

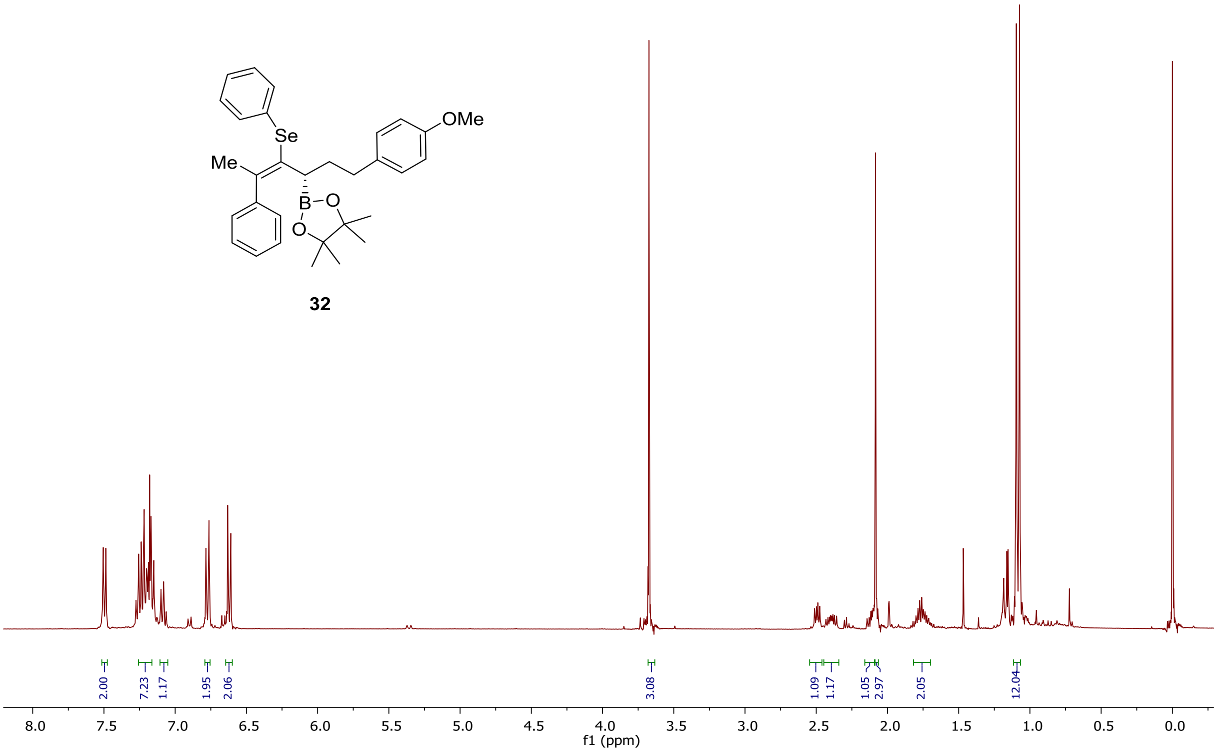
Se
OMe
Me
B
O
O
32
2.00
7.23
1.17
1.95
2.06
3.08
1.09
1.17
1.05
2.97
2.05
12.04
8.0 7.5 7.0 6.5 6.0 5.5 5.0 4.5 4.0 3.5 3.0 2.5 2.0 1.5 1.0 0.5 0.0
f1 (ppm)

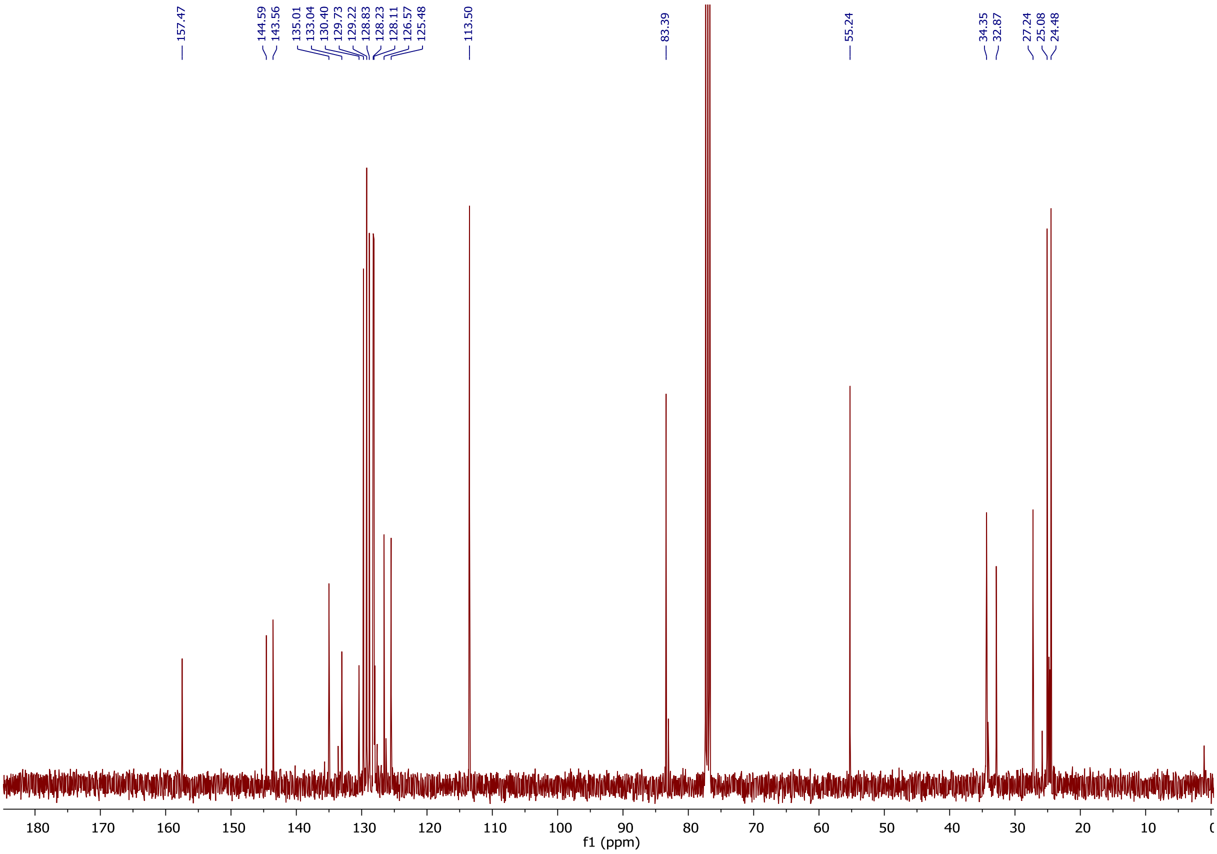
157.47
144.59
143.56
135.01
133.04
130.40
129.73
129.22
128.83
128.23
128.11
126.57
125.48
113.50
83.39
55.24
34.35
32.87
27.24
25.08
24.48
180 170 160 150 140 130 120 110 100 90 80 70 60 50 40 30 20 10
f1 (ppm)

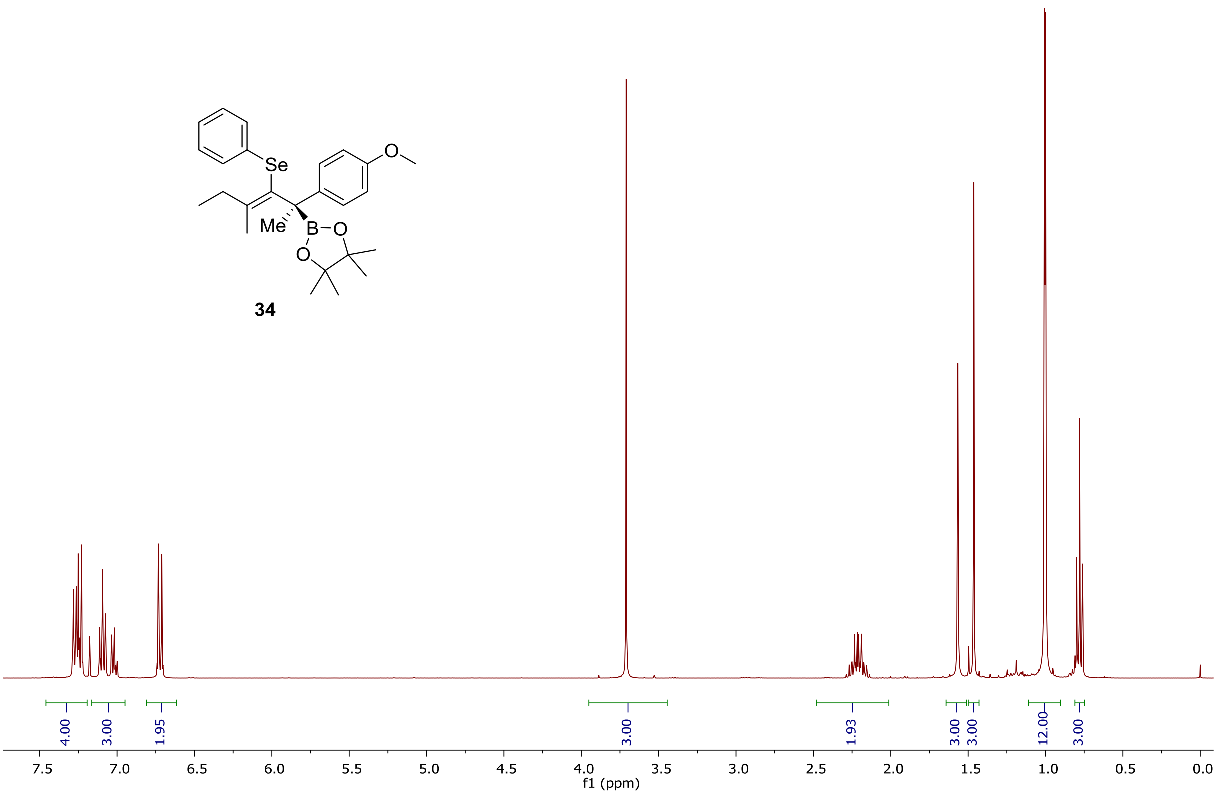
Se
O
Me
B
O
O
34
4.00
3.00
1.95
3.00
1.93
3.00
3.00
12.00
3.00
7.5
7.0
6.5
6.0
5.5
5.0
4.5
4.0
3.5
3.0
2.5
2.0
1.5
1.0
0.5
0.0
f1 (ppm)

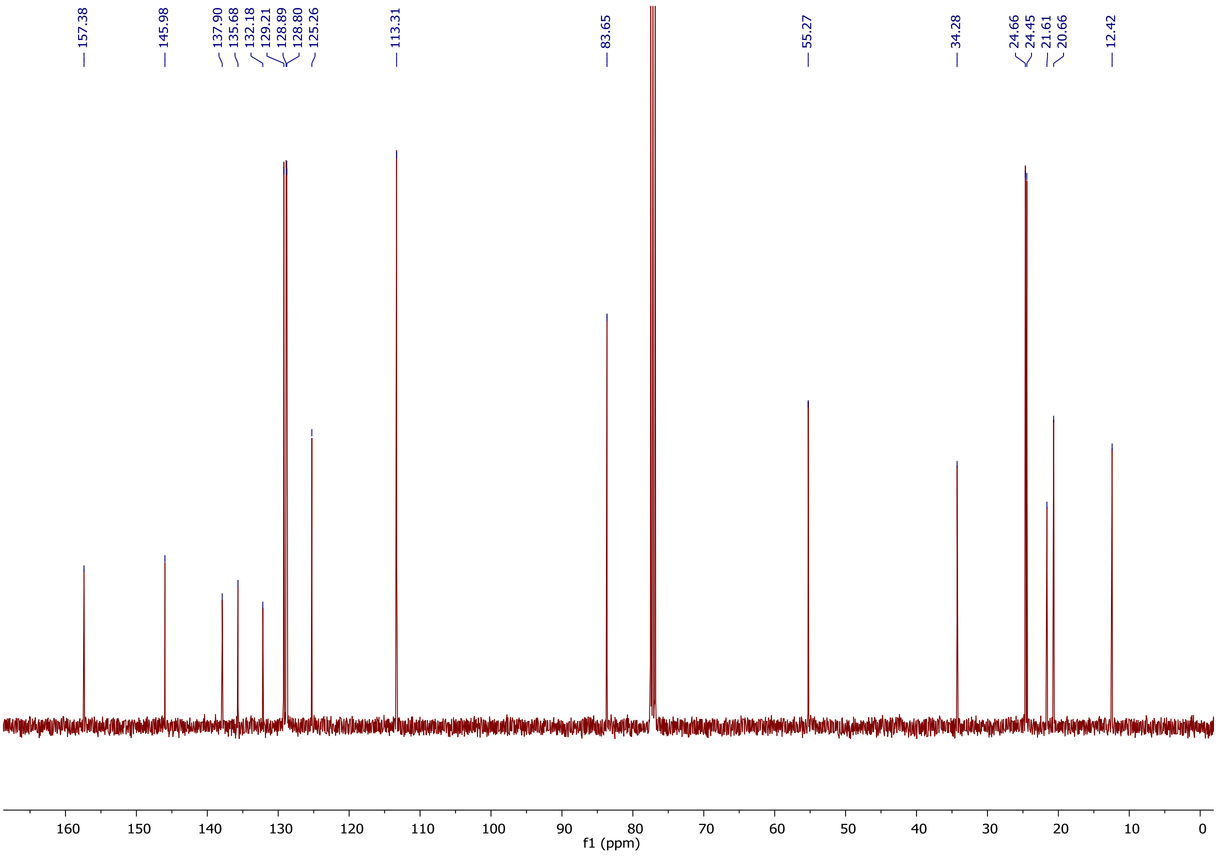
157.38
145.98
137.90
135.68
132.18
129.21
128.89
128.80
125.26
113.31
83.65
55.27
34.28
24.66
24.45
21.61
20.66
12.42
160
150
140
130
120
110
100
90
80
70
60
50
40
30
20
10
0
f1 (ppm)

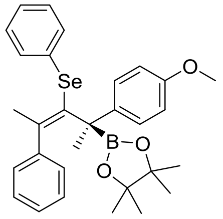
Se
O
B
O
O

36

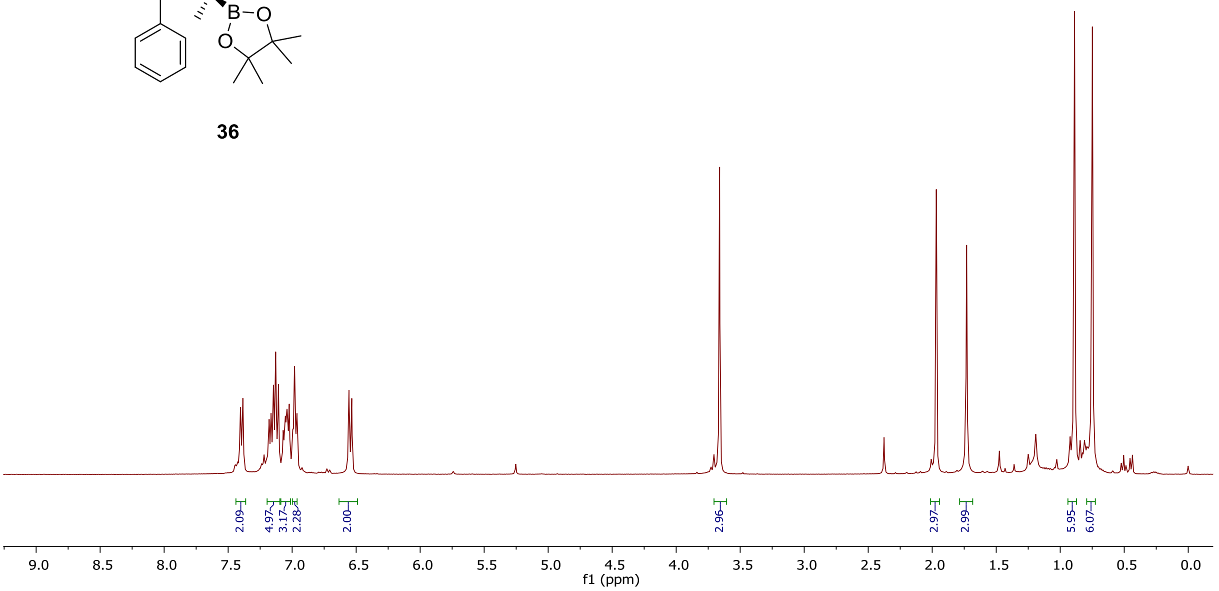
2.09
4.97
3.17
2.28
2.00
2.96
2.97
2.99
5.95
6.07
9.0 8.5 8.0 7.5 7.0 6.5 6.0 5.5 5.0 4.5 4.0 3.5 3.0 2.5 2.0 1.5 1.0 0.5 0.0
f1 (ppm)

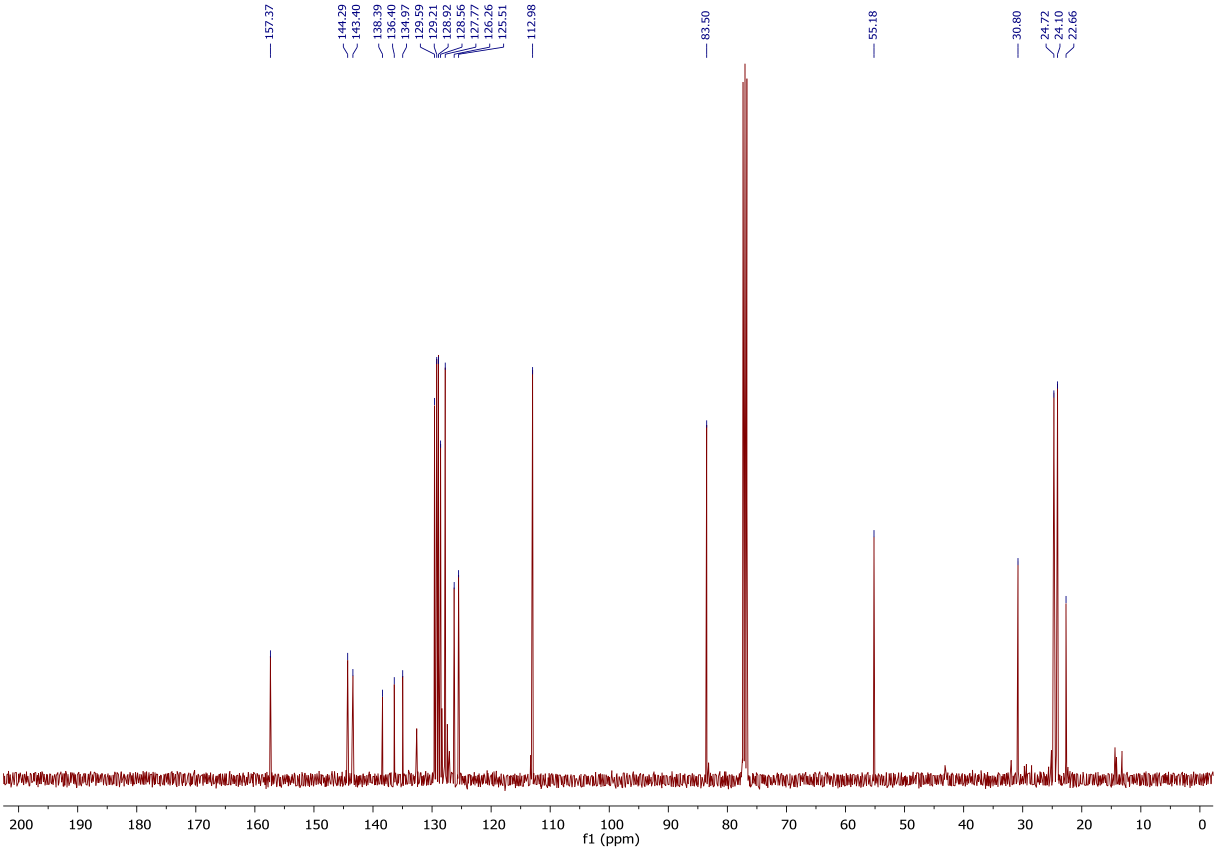
157.37
144.29
143.40
138.39
136.40
134.97
129.59
129.21
128.92
128.56
127.77
126.26
125.51
112.98
83.50
55.18
30.80
24.72
24.10
22.66
200 190 180 170 160 150 140 130 120 110 100 90 80 70 60 50 40 30 20 10 0
f1 (ppm)

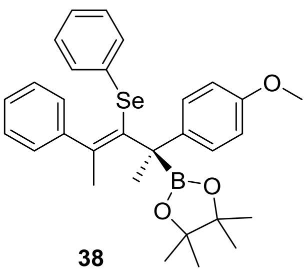
Se
B
O
O

38

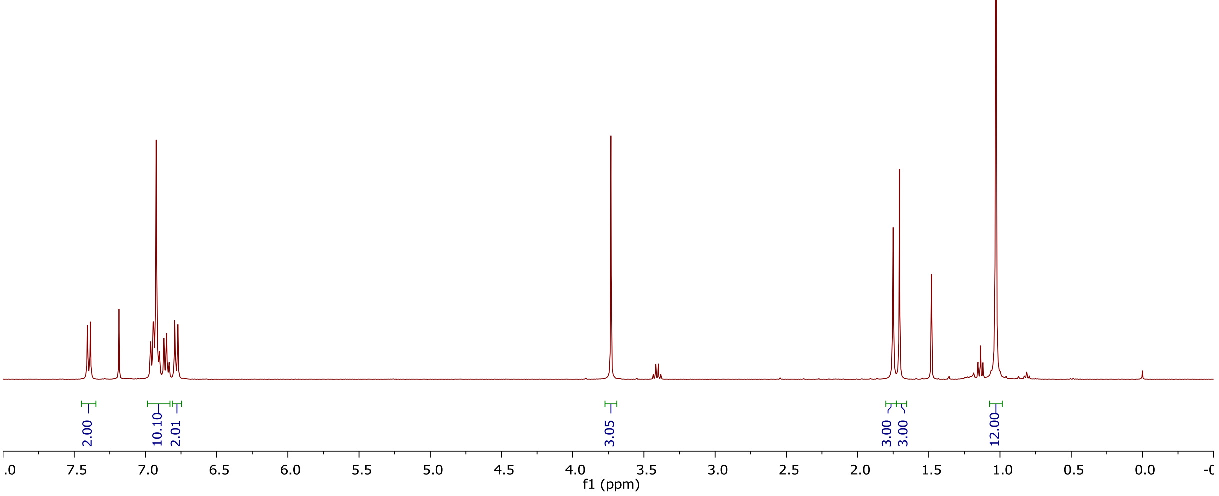
2.00
10.10
2.01
3.05
3.00
3.00
12.00
7.5
7.0
6.5
6.0
5.5
5.0
4.5
4.0
3.5
3.0
2.5
2.0
1.5
1.0
0.5
0.0
f1 (ppm)

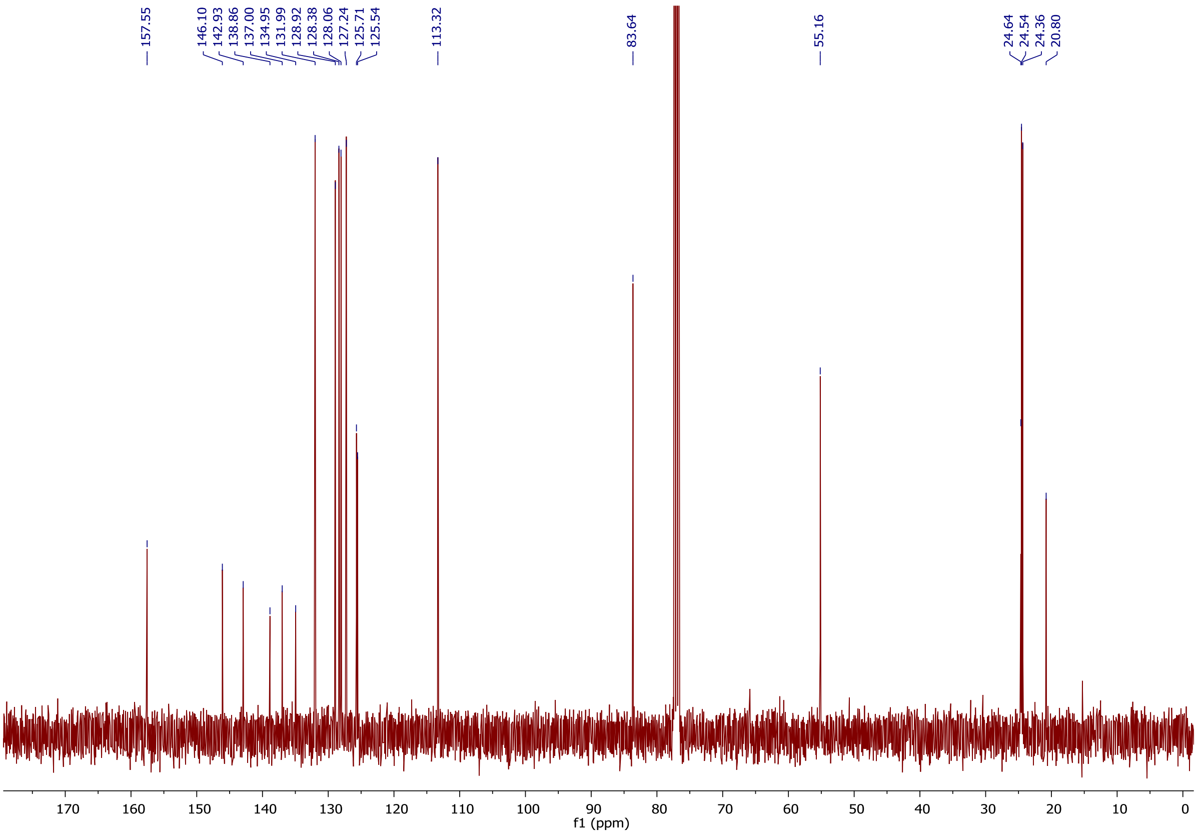
157.55
146.10
142.93
138.86
137.00
134.95
131.99
128.92
128.38
128.06
127.24
125.71
125.54
113.32
83.64
55.16
24.64
24.54
24.36
20.80
170
160
150
140
130
120
110
100
90
80
70
60
50
40
30
20
10
0
f1 (ppm)

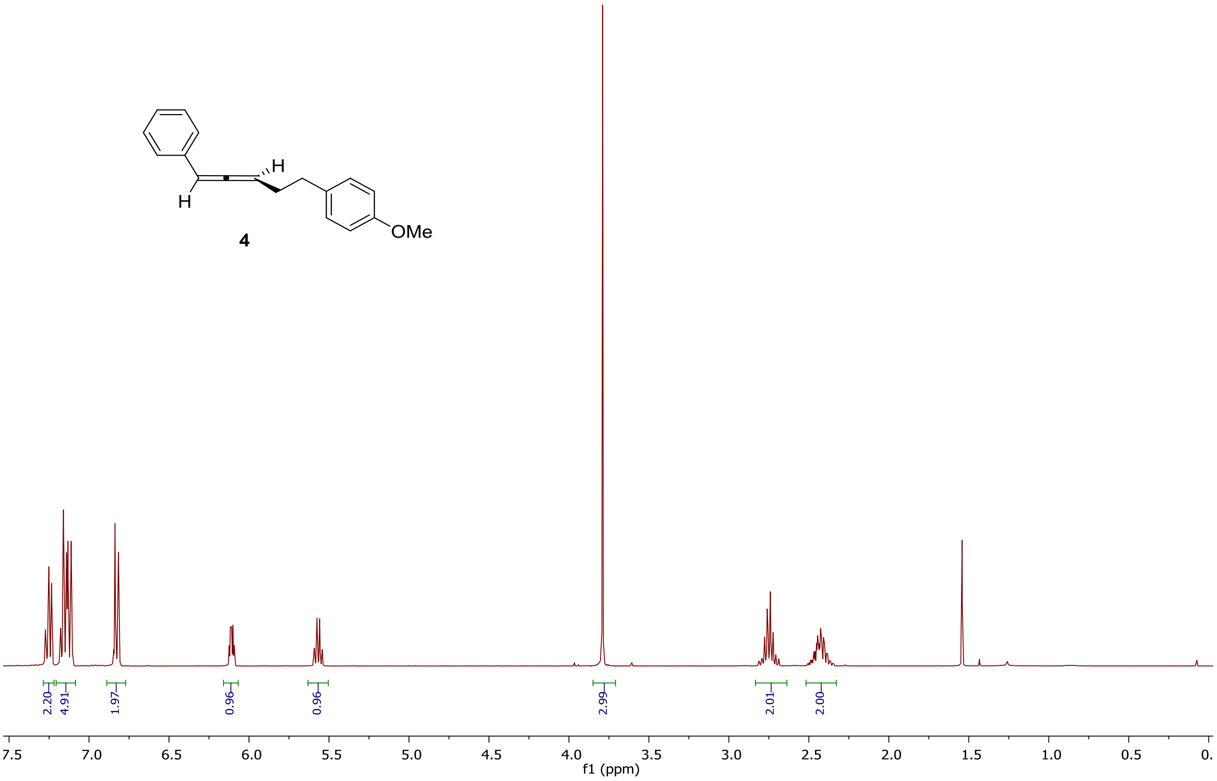
H
H
OMe
2.20
4.91
1.97
0.96
0.96
2.99
2.01
2.00
7.5 7.0 6.5 6.0 5.5 5.0 4.5 4.0 3.5 3.0 2.5 2.0 1.5 1.0 0.5 0.
f1 (ppm)

4

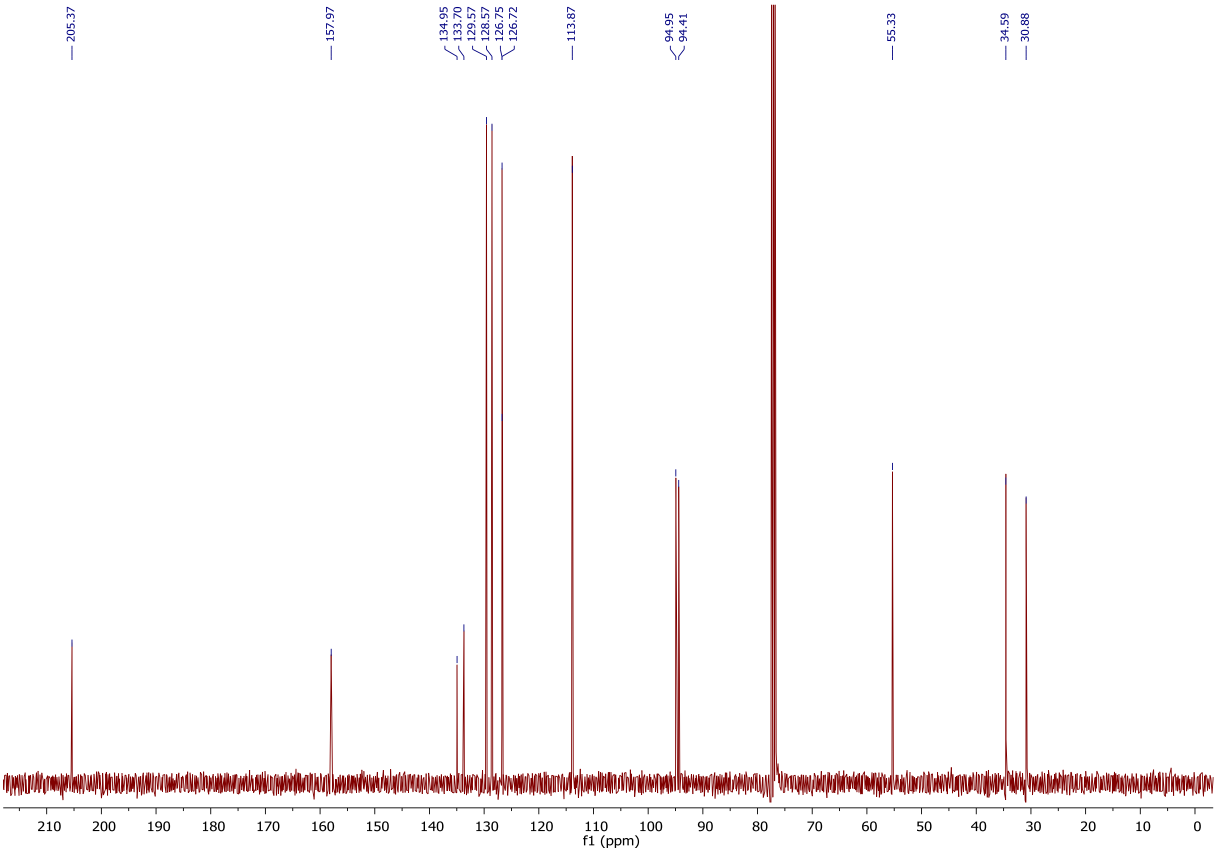
205.37
157.97
134.95
133.70
129.57
128.57
126.75
126.72
113.87
94.95
94.41
55.33
34.59
30.88
210 200 190 180 170 160 150 140 130 120 110 100 90 80 70 60 50 40 30 20 10 0
f1 (ppm)

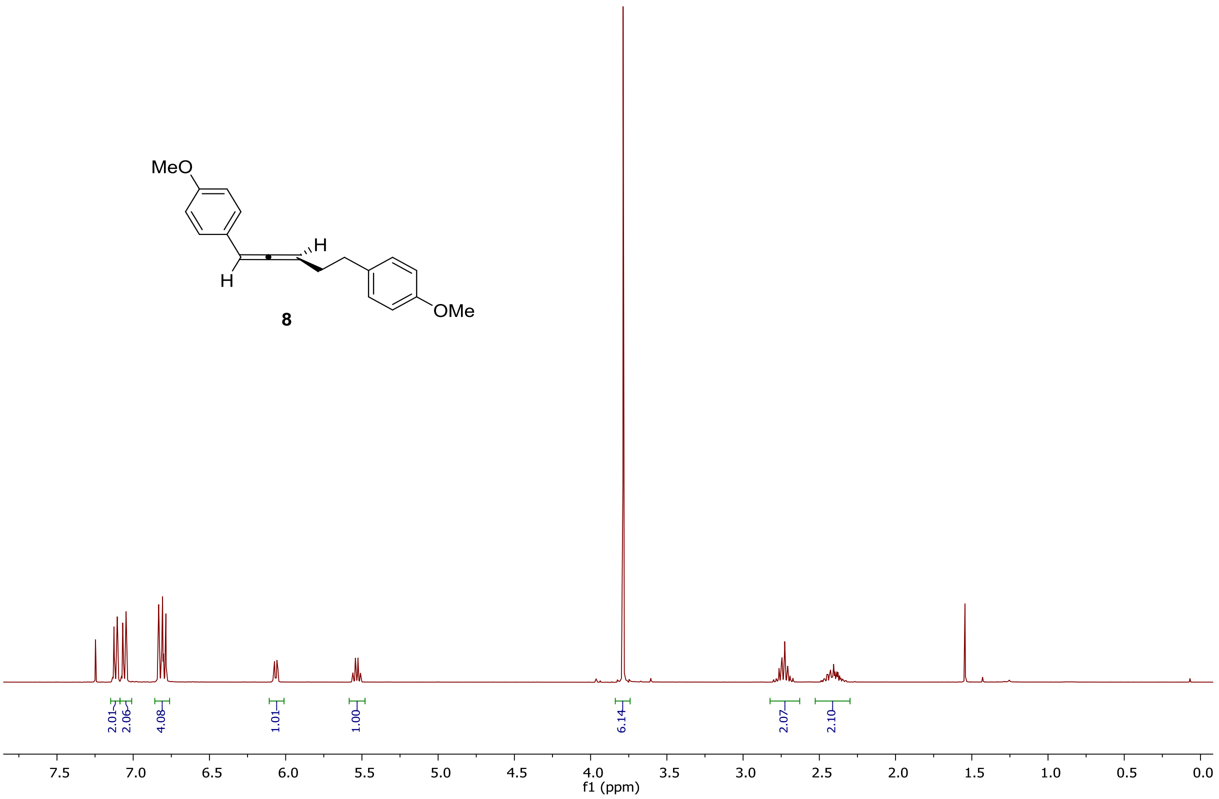
MeO
H
H
8
OMe
2.01
2.06
4.08
1.01
1.00
6.14
2.07
2.10
7.5 7.0 6.5 6.0 5.5 5.0 4.5 4.0 3.5 3.0 2.5 2.0 1.5 1.0 0.5 0.0
f1 (ppm)

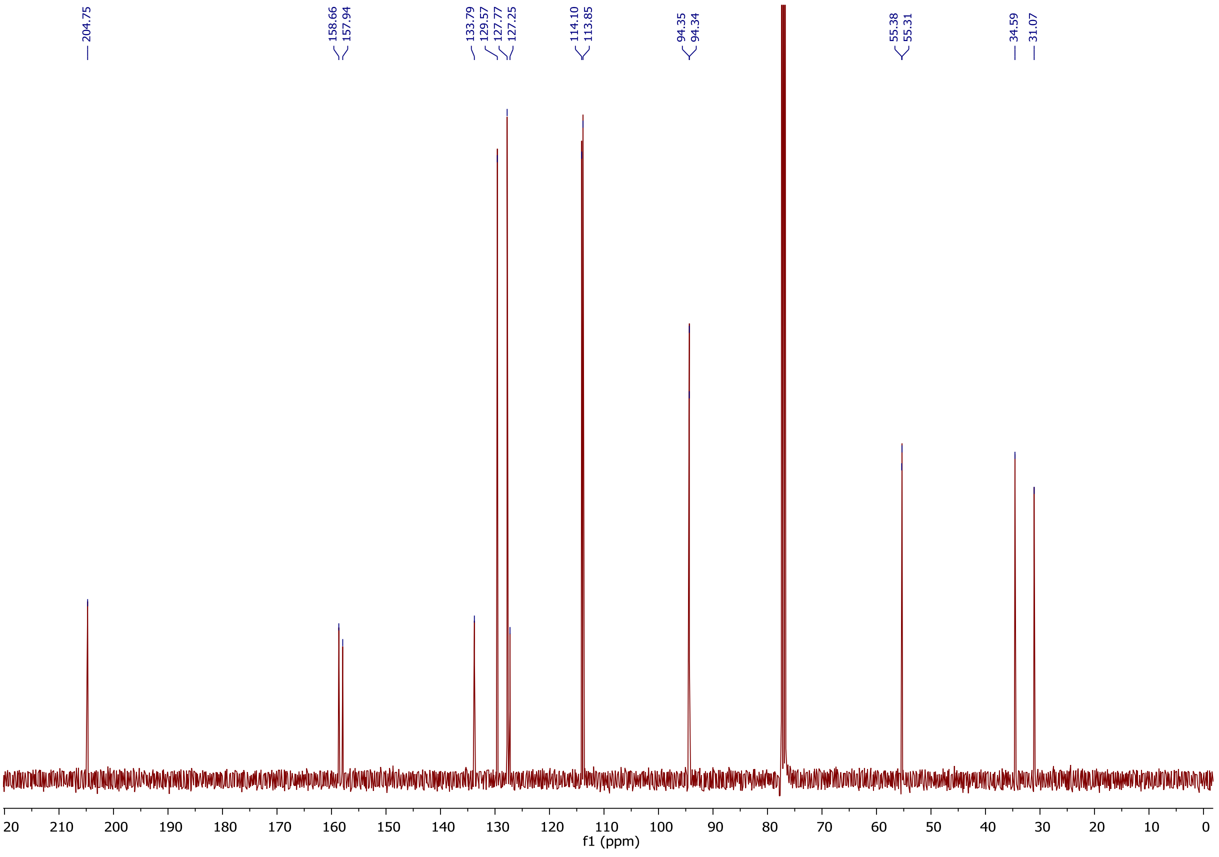
204.75
158.66
157.94
133.79
129.57
127.77
127.25
114.10
113.85
94.35
94.34
55.38
55.31
34.59
31.07
20 210 200 190 180 170 160 150 140 130 120 110 100 90 80 70 60 50 40 30 20 10 0
f1 (ppm)

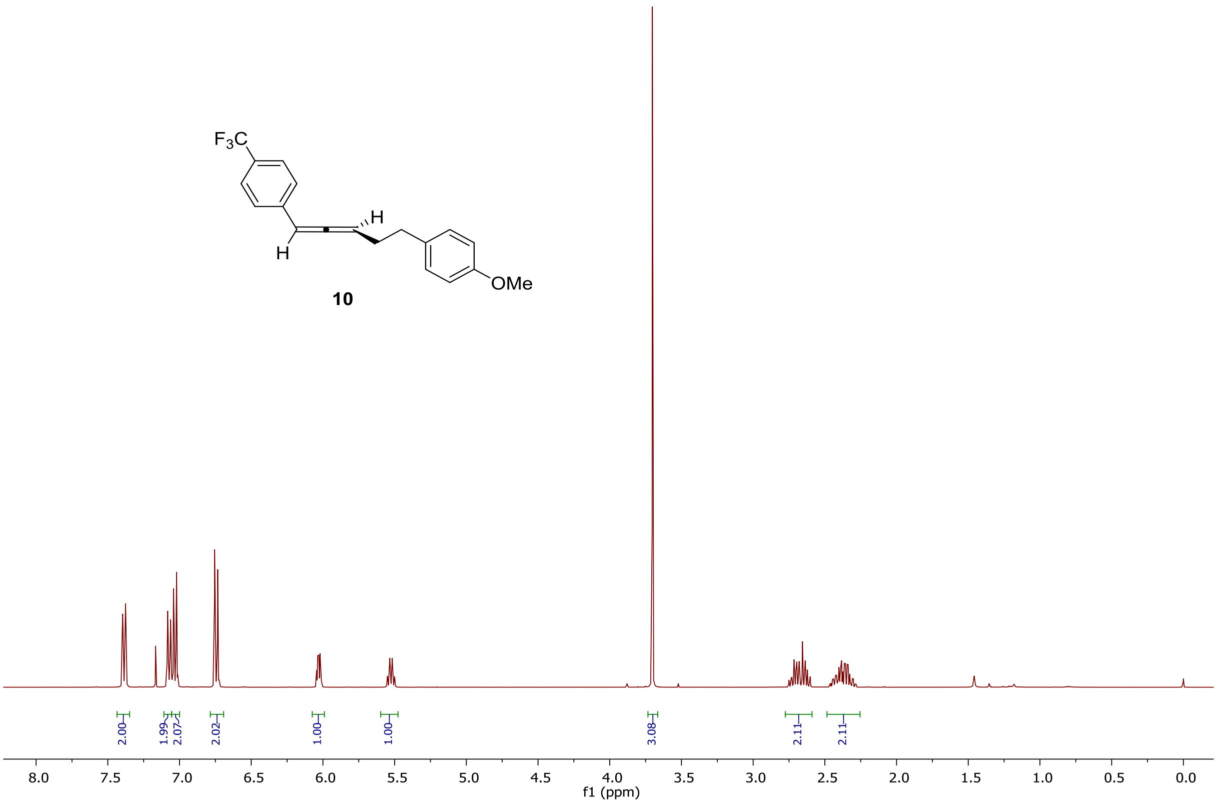
F3C
H
H
OMe
10
2.00
1.99
2.07
2.02
1.00
1.00
3.08
2.11
2.11
8.0 7.5 7.0 6.5 6.0 5.5 5.0 4.5 4.0 3.5 3.0 2.5 2.0 1.5 1.0 0.5 0.0
f1 (ppm)

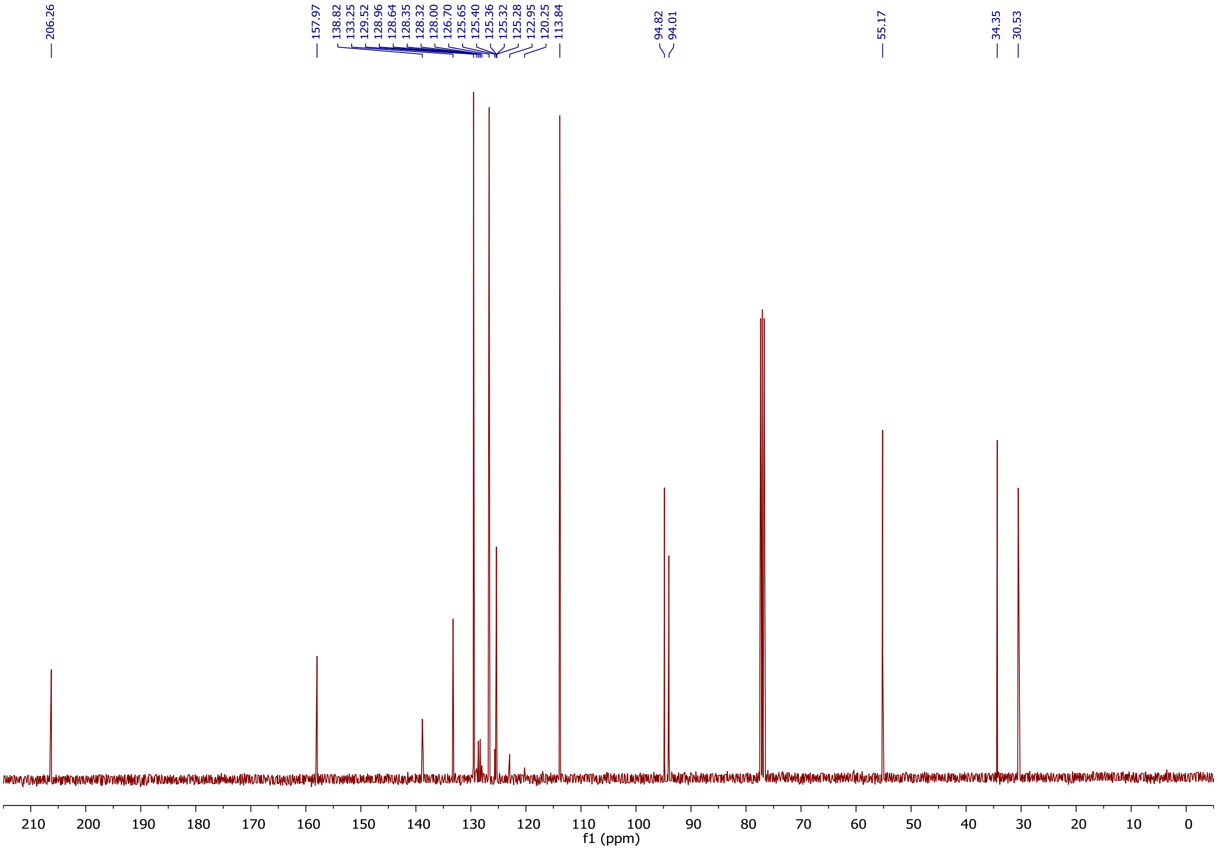
206.26
157.97
138.82
133.25
129.52
128.96
128.64
128.35
128.32
128.00
126.70
125.65
125.40
125.36
125.32
125.28
122.95
120.25
113.84
94.82
94.01
55.17
34.35
30.53
210 200 190 180 170 160 150 140 130 120 110 100 90 80 70 60 50 40 30 20 10 0
f1 (ppm)

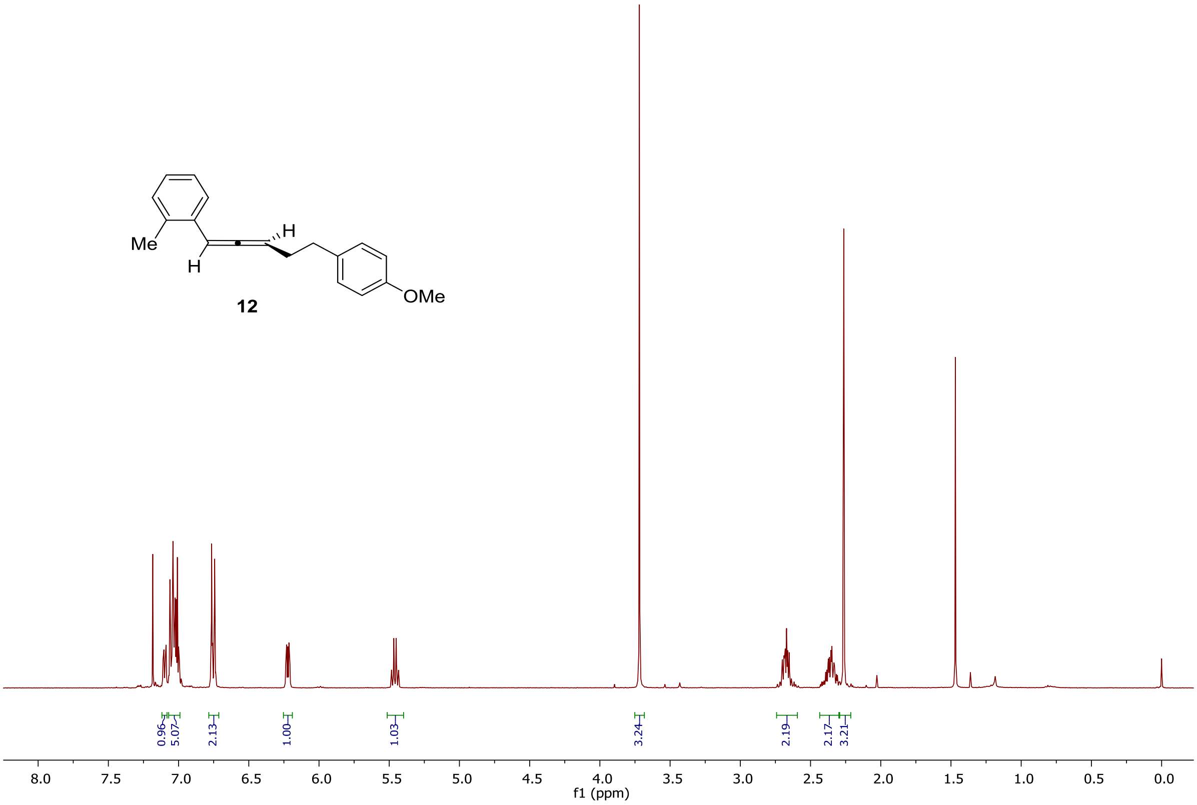
Me
H
H
OMe
12
0.96
5.07
2.13
1.00
1.03
3.24
2.19
2.17
3.21
8.0 7.5 7.0 6.5 6.0 5.5 5.0 4.5 4.0 3.5 3.0 2.5 2.0 1.5 1.0 0.5 0.0
f1 (ppm)

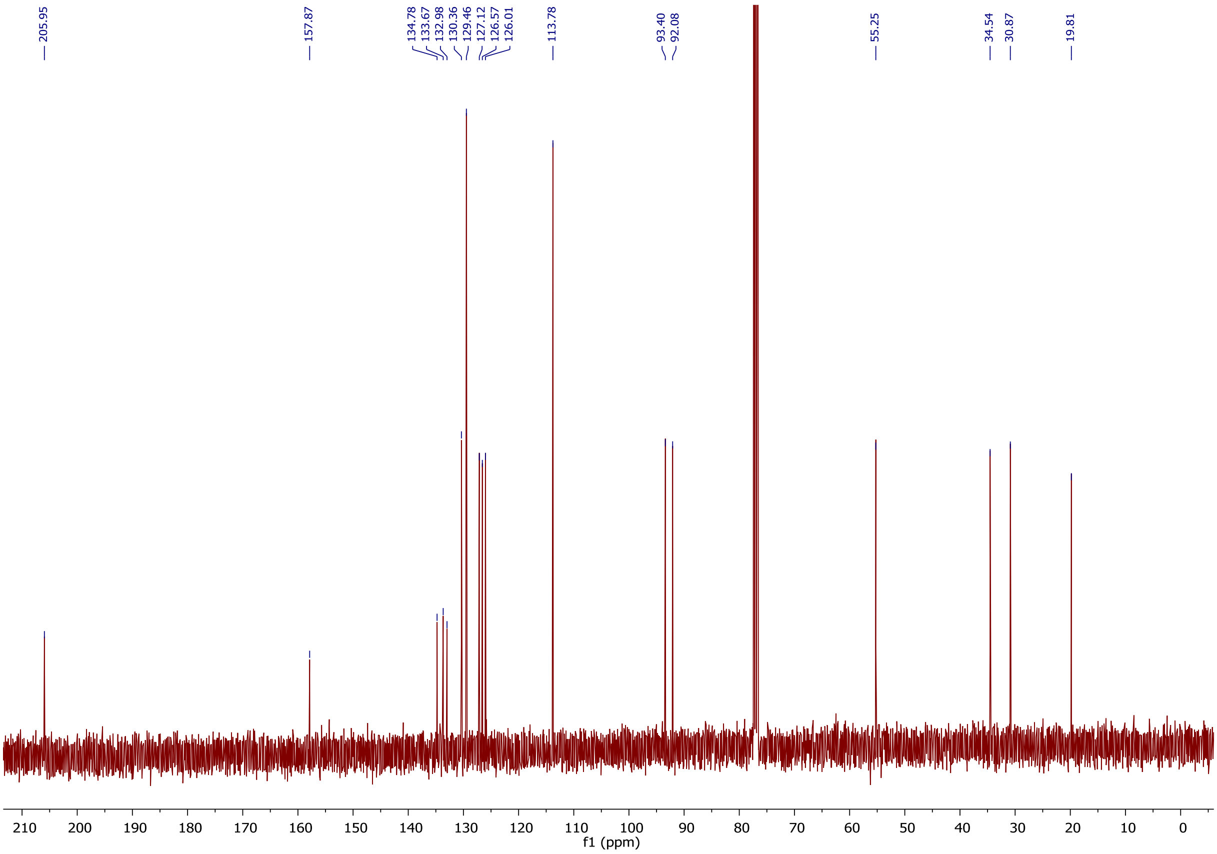
205.95
157.87
134.78
133.67
132.98
130.36
129.46
127.12
126.57
126.01
113.78
93.40
92.08
55.25
34.54
30.87
19.81
210 200 190 180 170 160 150 140 130 120 110 100 90 80 70 60 50 40 30 20 10 0
f1 (ppm)

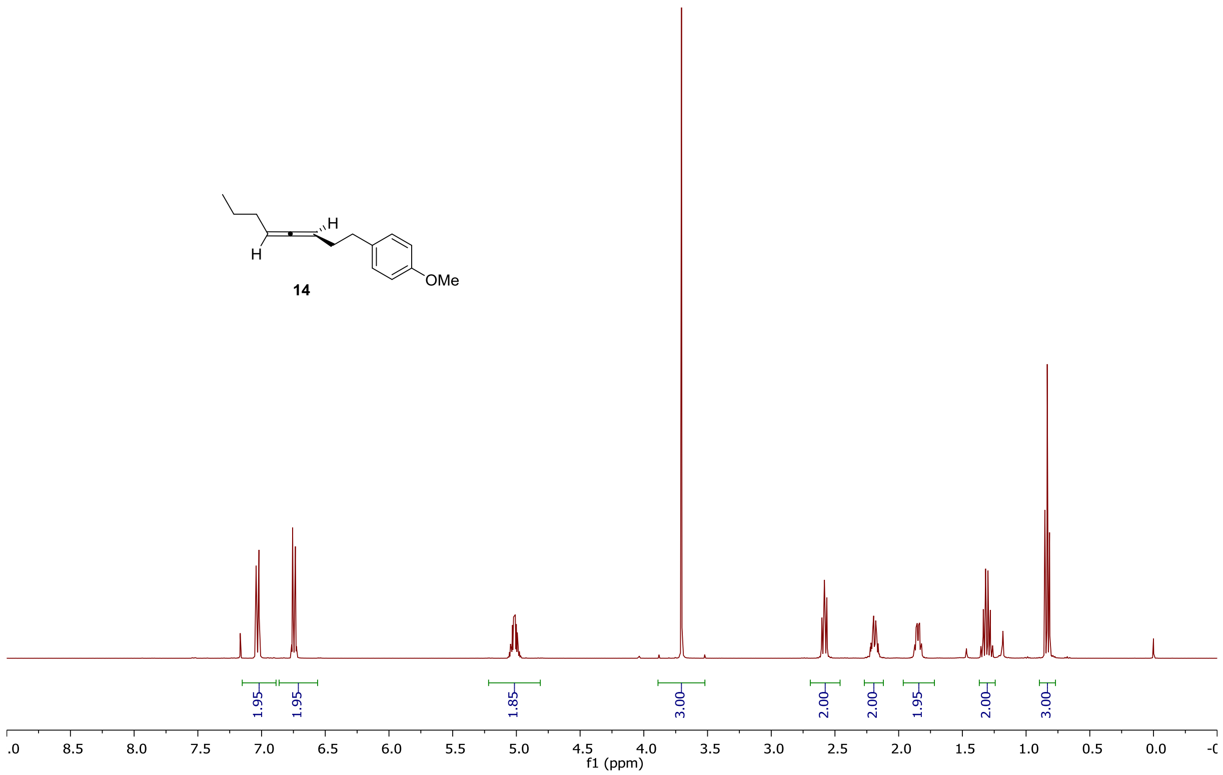
H
H
OMe
14
1.95
1.95
1.85
3.00
2.00
2.00
1.95
2.00
3.00
f1 (ppm)

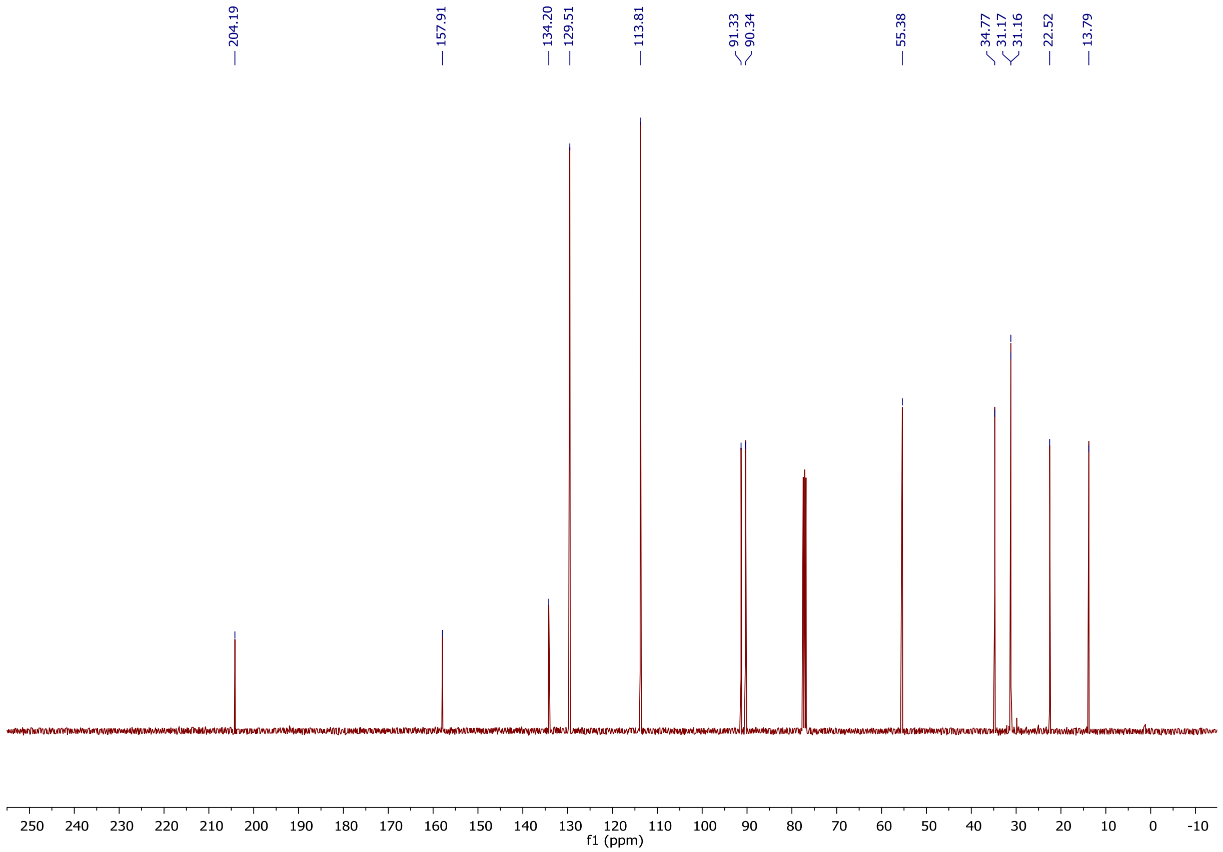
204.19
157.91
134.20
129.51
113.81
91.33
90.34
55.38
34.77
31.17
31.16
22.52
13.79
f1 (ppm)

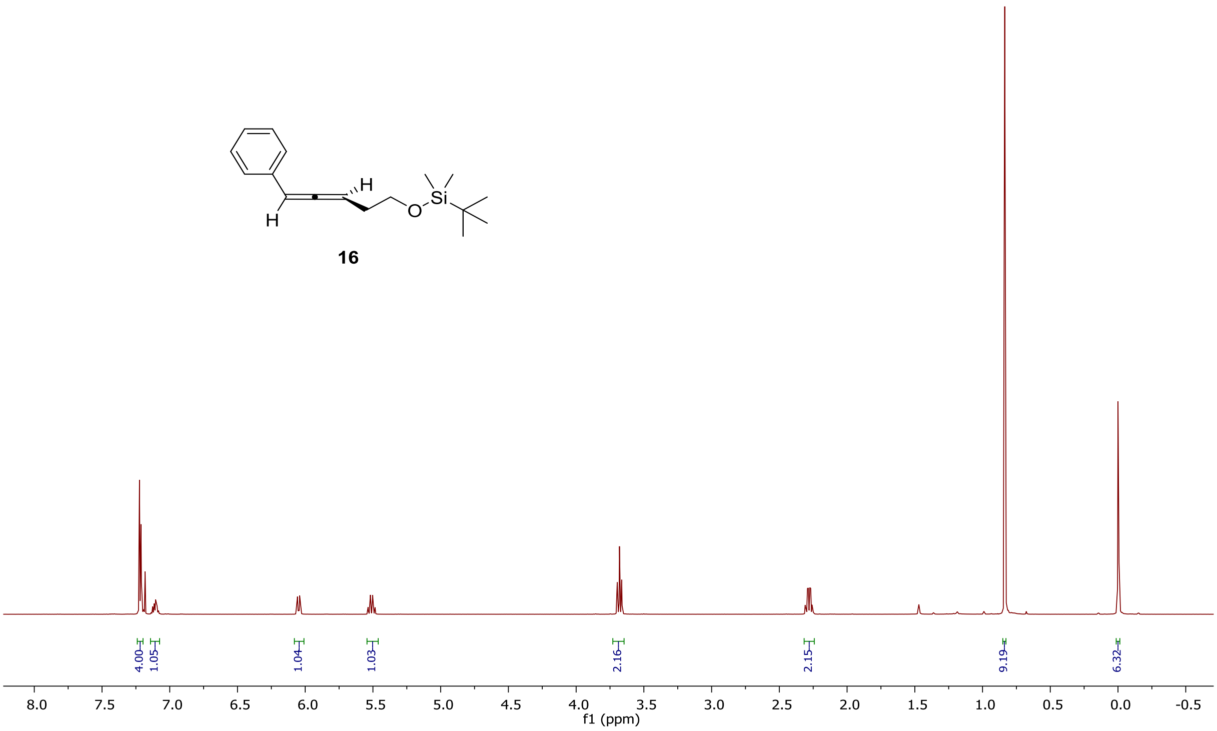
H
H
O
Si
16
4.00
1.05
1.04
1.03
2.16
2.15
9.19
6.32
8.0 7.5 7.0 6.5 6.0 5.5 5.0 4.5 4.0 3.5 3.0 2.5 2.0 1.5 1.0 0.5 0.0 -0.5
f1 (ppm)

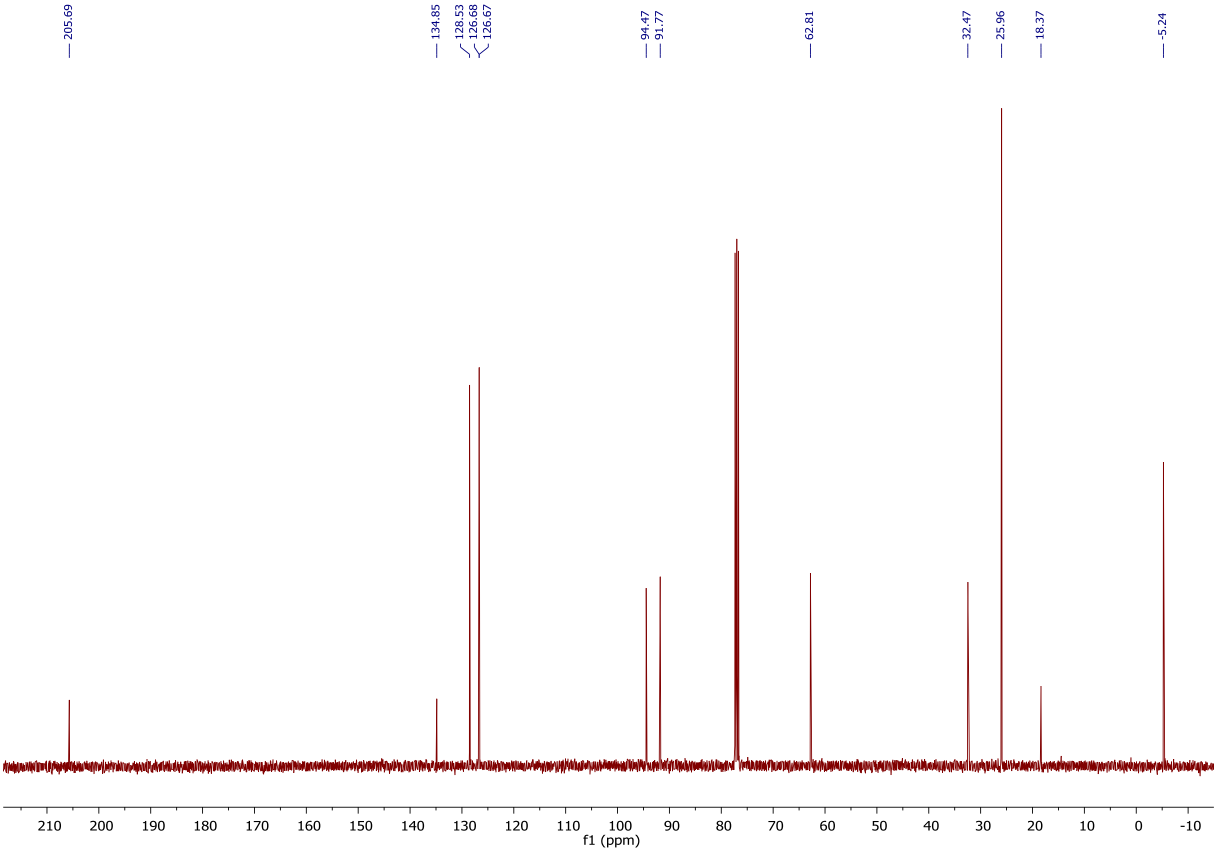
205.69
134.85
128.53
126.68
126.67
94.47
91.77
62.81
32.47
25.96
18.37
-5.24
210 200 190 180 170 160 150 140 130 120 110 100 90 80 70 60 50 40 30 20 10 0 -10
f1 (ppm)

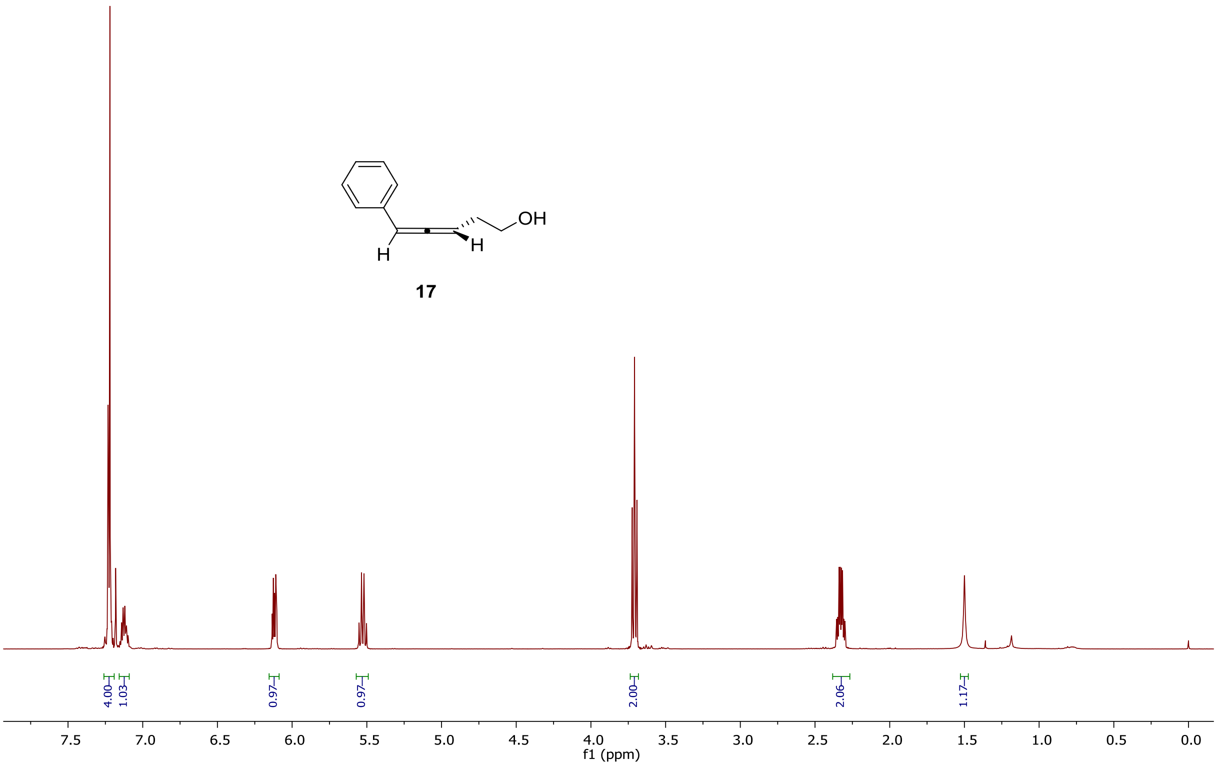
OH
H
H
4.00
1.03
0.97
0.97
2.00
2.06
1.17
7.5 7.0 6.5 6.0 5.5 5.0 4.5 4.0 3.5 3.0 2.5 2.0 1.5 1.0 0.5 0.0
f1 (ppm)

17

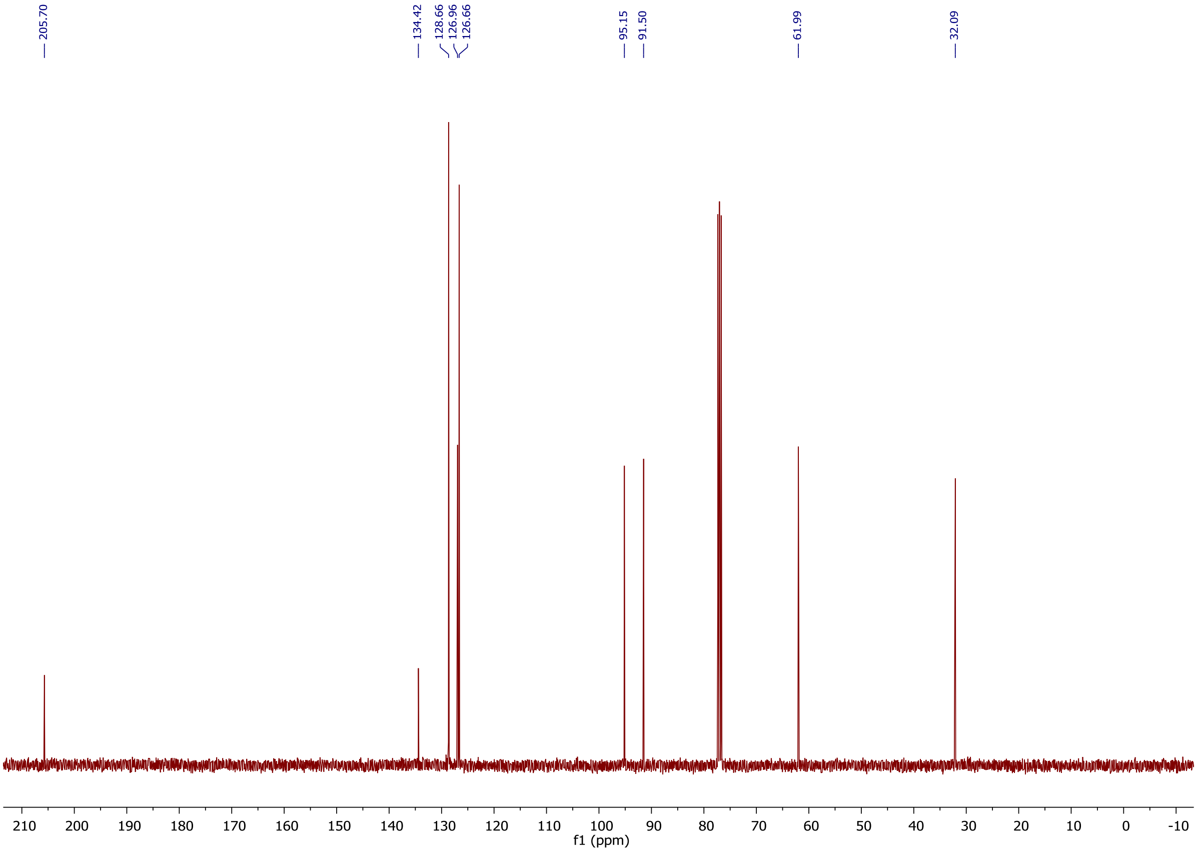
205.70
134.42
128.66
126.96
126.66
95.15
91.50
61.99
32.09
210 200 190 180 170 160 150 140 130 120 110 100 90 80 70 60 50 40 30 20 10 0 -10
f1 (ppm)

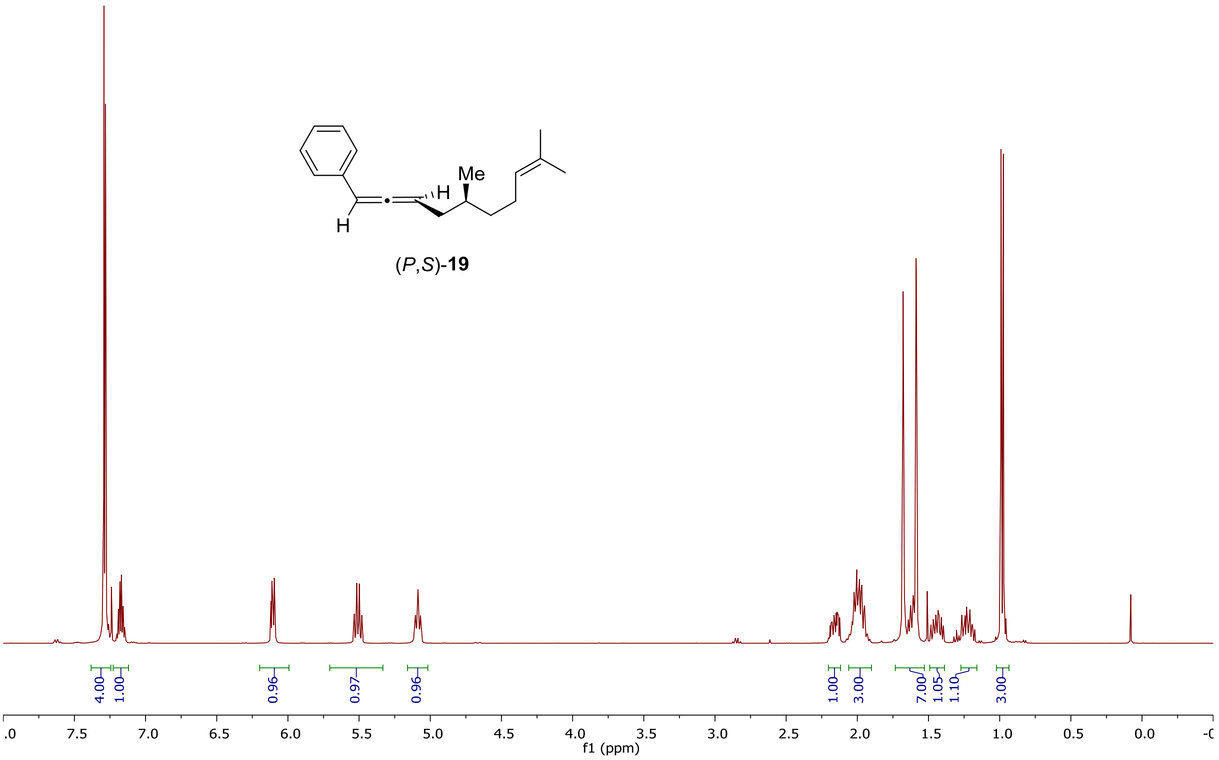
Me
H
H
4.00
1.00
0.96
0.97
0.96
1.00
3.00
7.00
1.05
1.10
3.00
f1 (ppm)

(*P*,*S*)-**19**

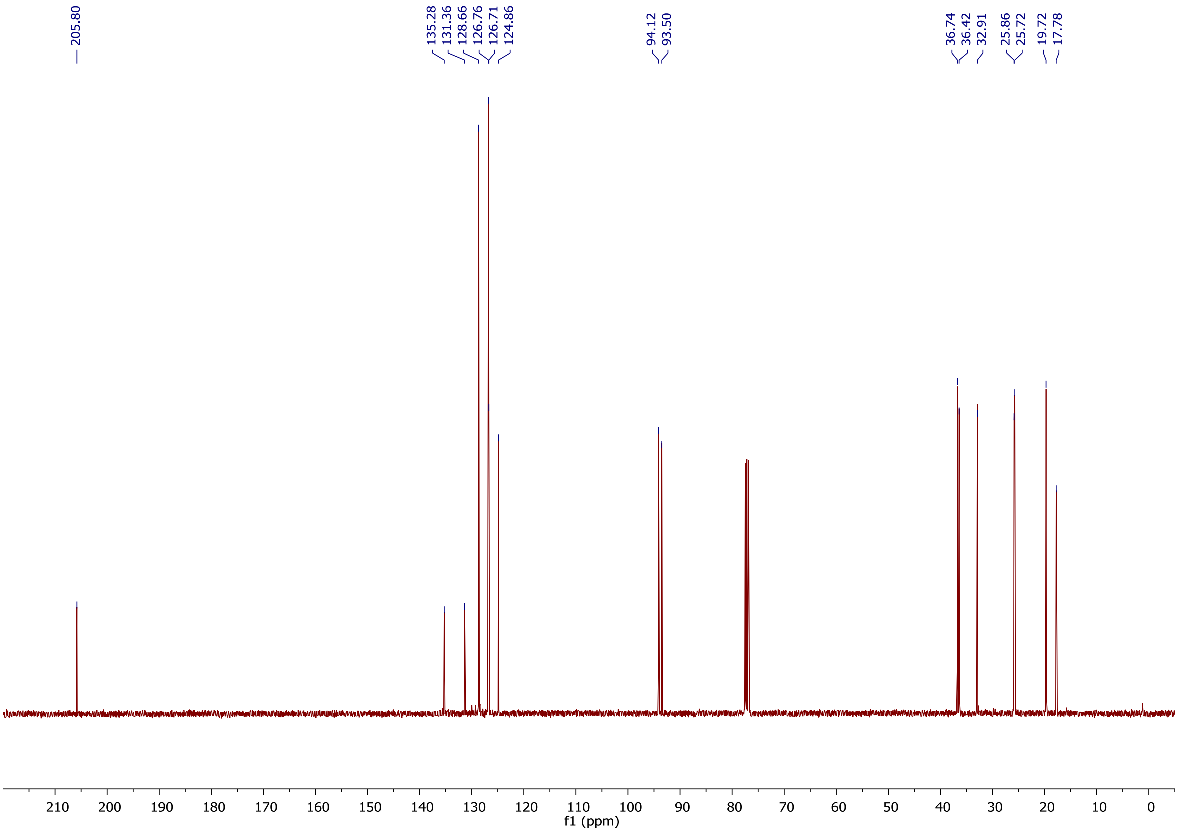
205.80
135.28
131.36
128.66
126.76
126.71
124.86
94.12
93.50
36.74
36.42
32.91
25.86
25.72
19.72
17.78
f1 (ppm)

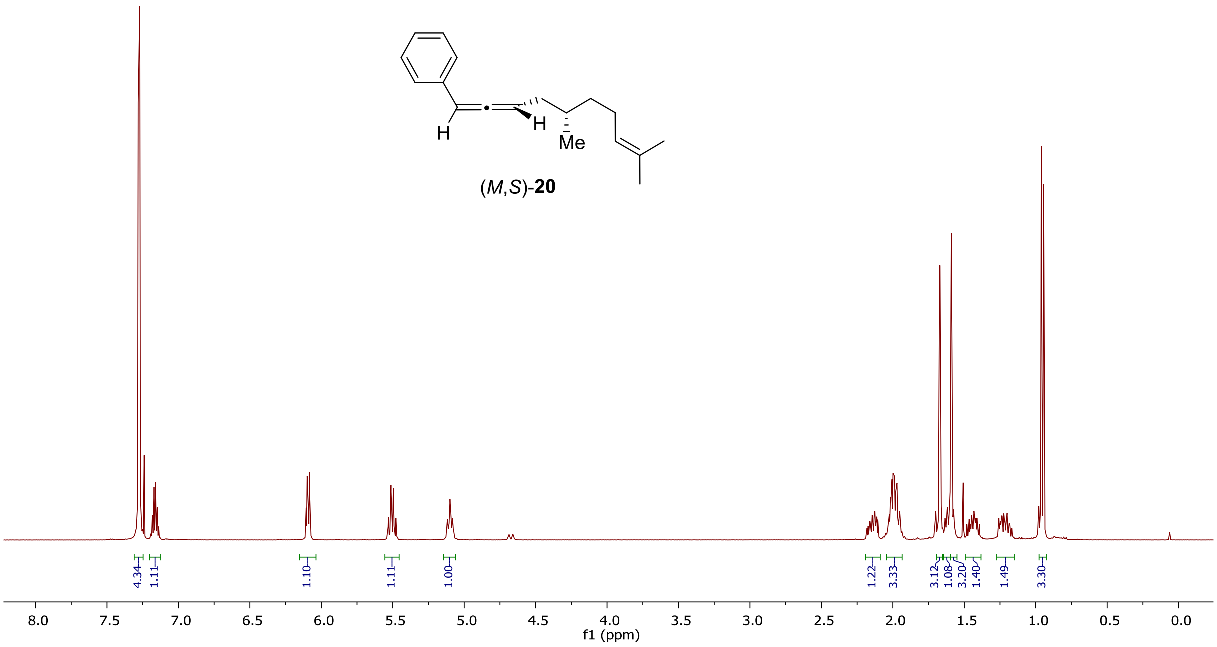
H
H
Me
4.34
1.11
1.10
1.11
1.00
1.22
3.33
3.12
1.08
3.20
1.40
1.49
3.30
8.0 7.5 7.0 6.5 6.0 5.5 5.0 4.5 4.0 3.5 3.0 2.5 2.0 1.5 1.0 0.5 0.0
f1 (ppm)

(*M*,*S*)-**20**

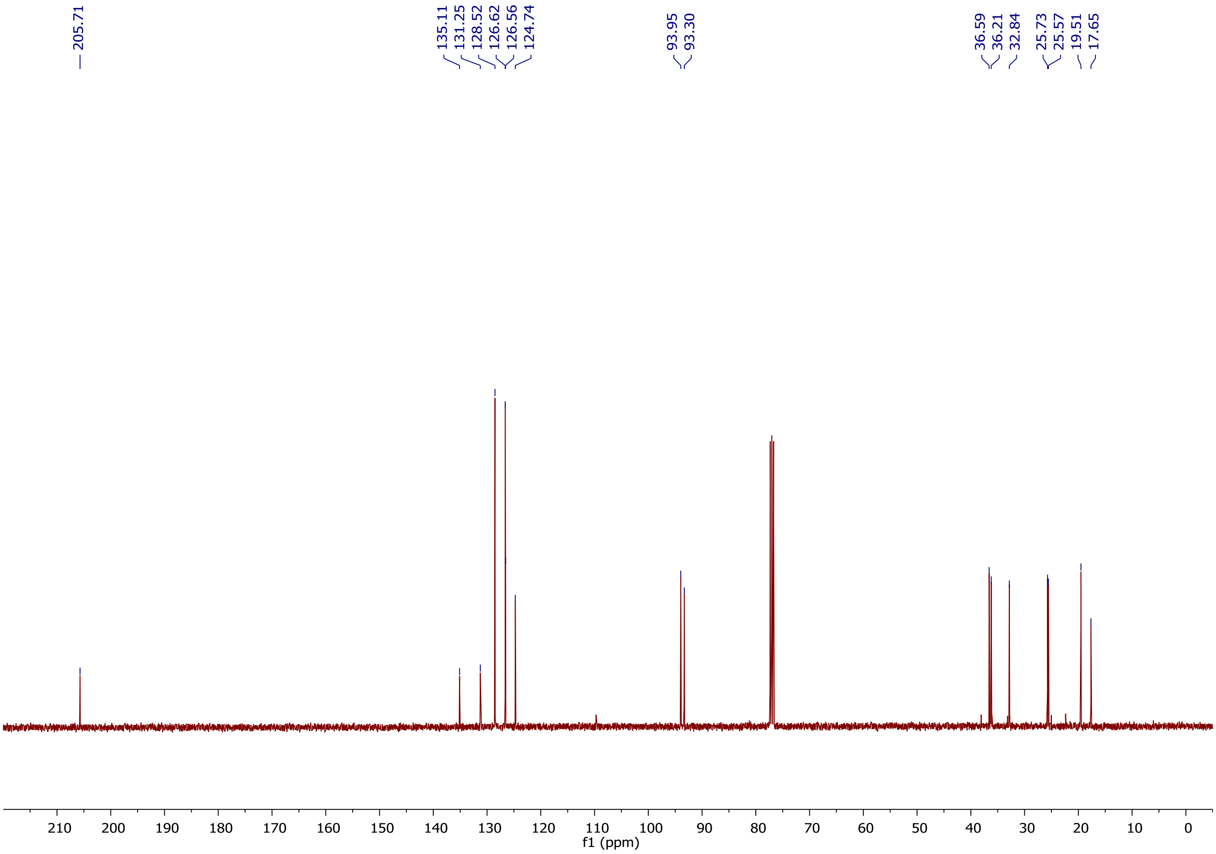
205.71
135.11
131.25
128.52
126.62
126.56
124.74
93.95
93.30
36.59
36.21
32.84
25.73
25.57
19.51
17.65
210 200 190 180 170 160 150 140 130 120 110 100 90 80 70 60 50 40 30 20 10 0
f1 (ppm)

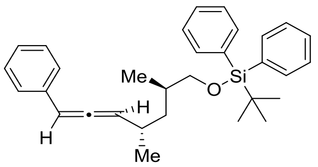

(*P*,*S*,*R*)-**22**

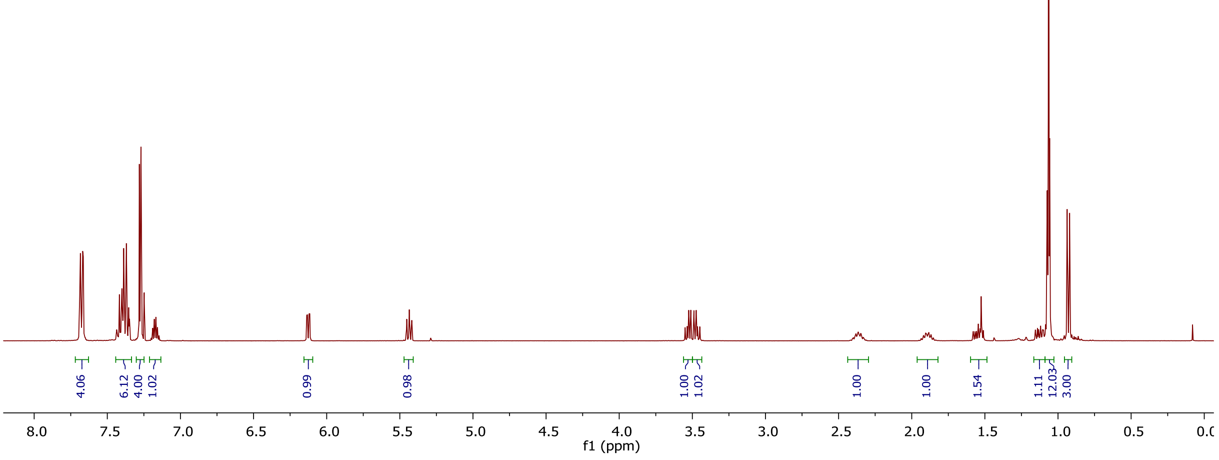

4.06
6.12
4.00
1.02
0.99
0.98
1.00
1.02
1.00
1.00
1.54
1.11
12.03
3.00
8.0 7.5 7.0 6.5 6.0 5.5 5.0 4.5 4.0 3.5 3.0 2.5 2.0 1.5 1.0 0.5 0.0
f1 (ppm)

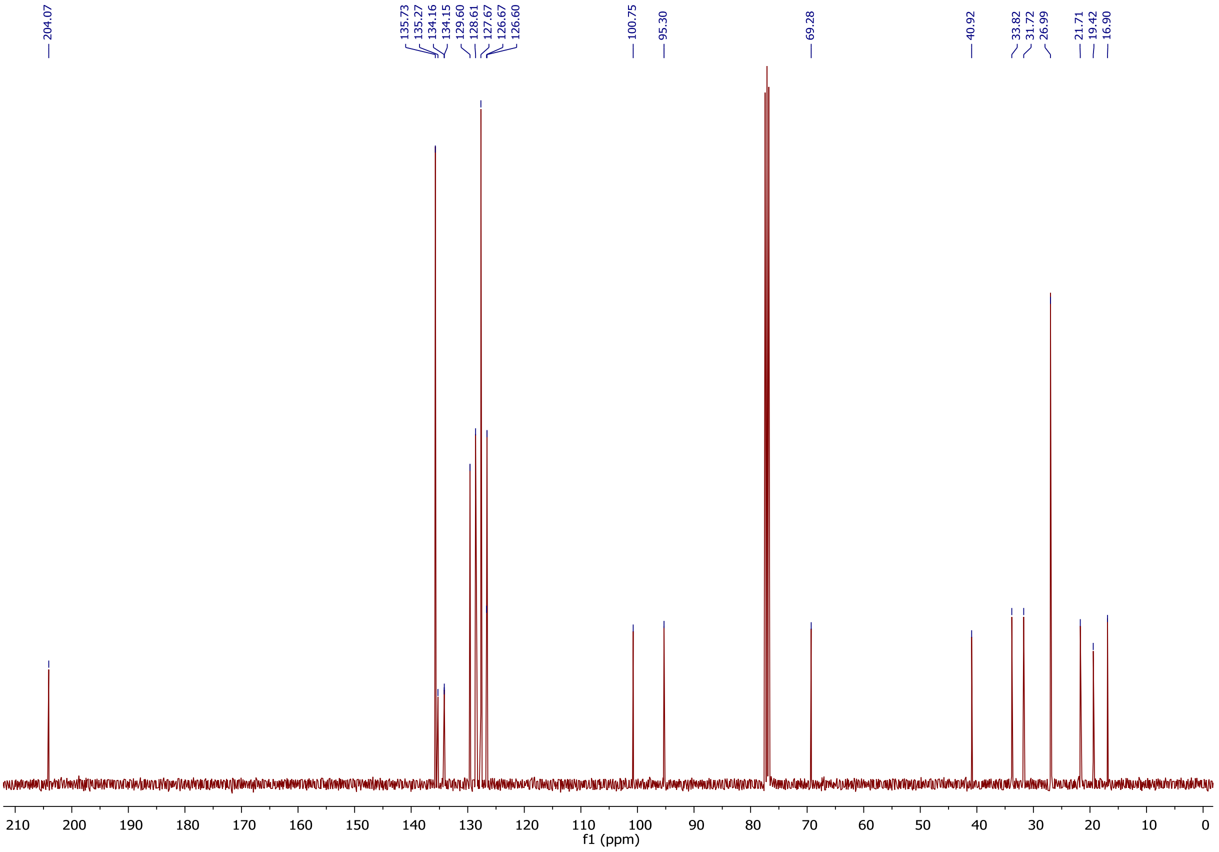

204.07
135.73
135.27
134.16
134.15
129.60
128.61
127.67
126.67
126.60
100.75
95.30
69.28
40.92
33.82
31.72
26.99
21.71
19.42
16.90
210 200 190 180 170 160 150 140 130 120 110 100 90 80 70 60 50 40 30 20 10 0
f1 (ppm)

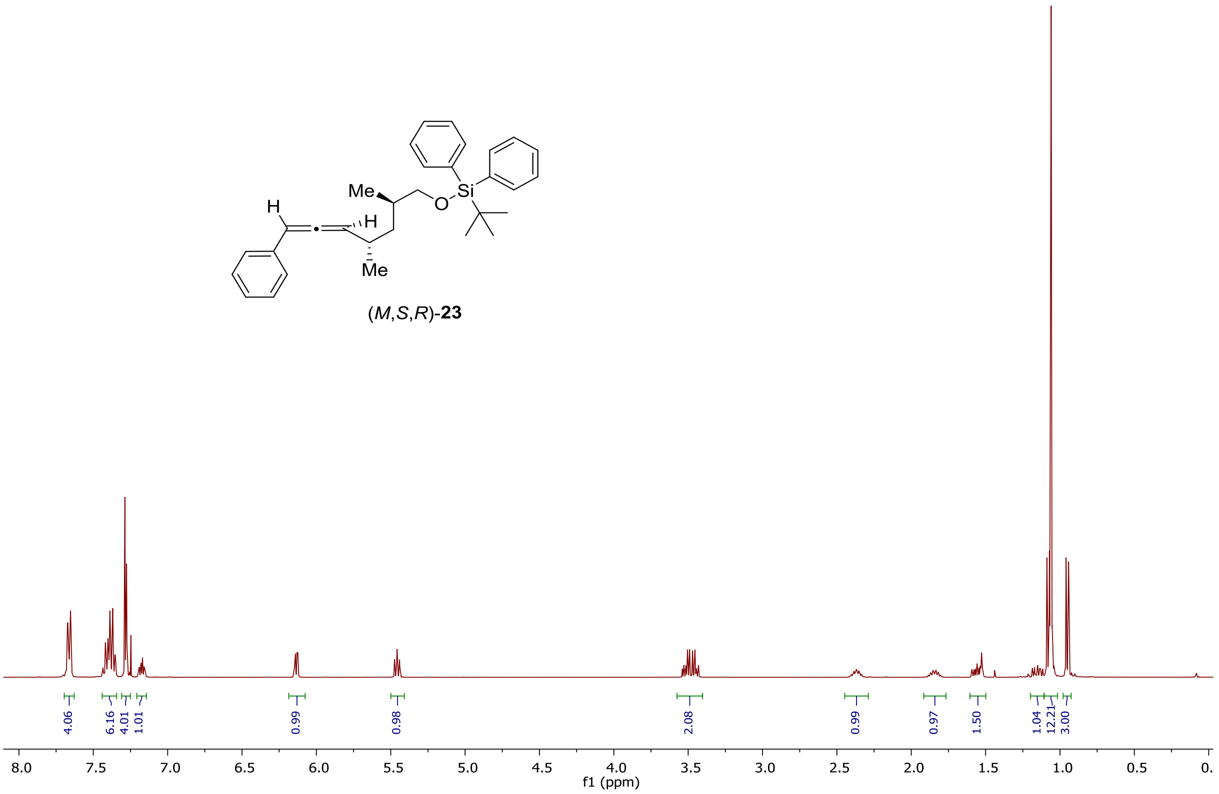
Me
H
H
O
Si
Me
(M,S,R)-23
4.06
6.16
4.01
1.01
0.99
0.98
2.08
0.99
0.97
1.50
1.04
12.21
3.00
8.0 7.5 7.0 6.5 6.0 5.5 5.0 4.5 4.0 3.5 3.0 2.5 2.0 1.5 1.0 0.5 0.
f1 (ppm)

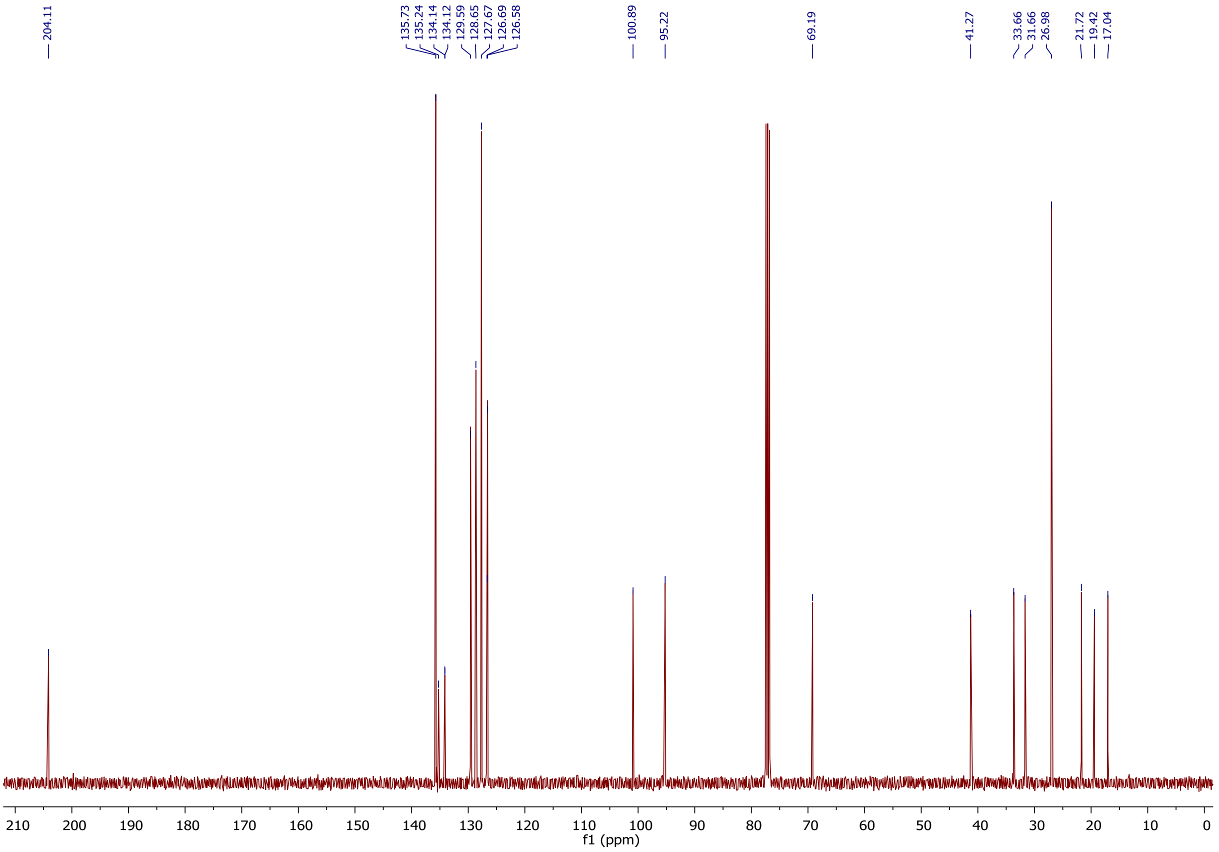
204.11
135.73
135.24
134.14
134.12
129.59
128.65
127.67
126.69
126.58
100.89
95.22
69.19
41.27
33.66
31.66
26.98
21.72
19.42
17.04
210 200 190 180 170 160 150 140 130 120 110 100 90 80 70 60 50 40 30 20 10 0
f1 (ppm)

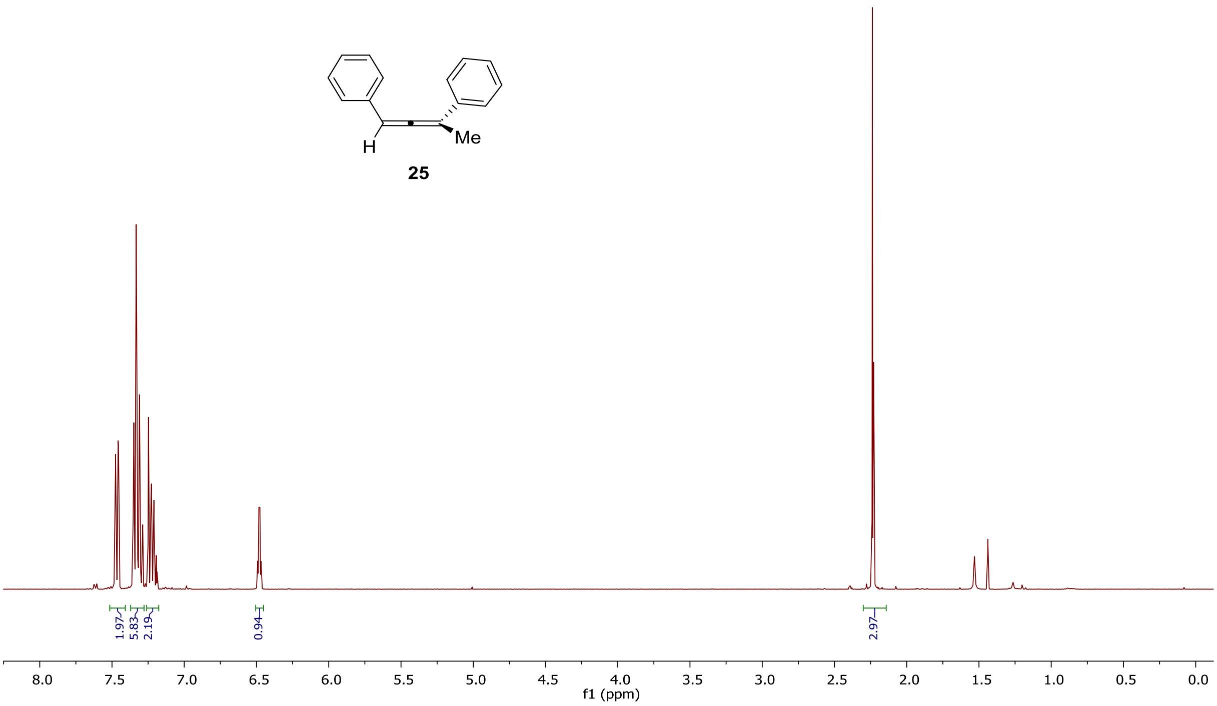
H
Me
25
1.97
5.83
2.19
0.94
2.97
8.0 7.5 7.0 6.5 6.0 5.5 5.0 4.5 4.0 3.5 3.0 2.5 2.0 1.5 1.0 0.5 0.0
f1 (ppm)

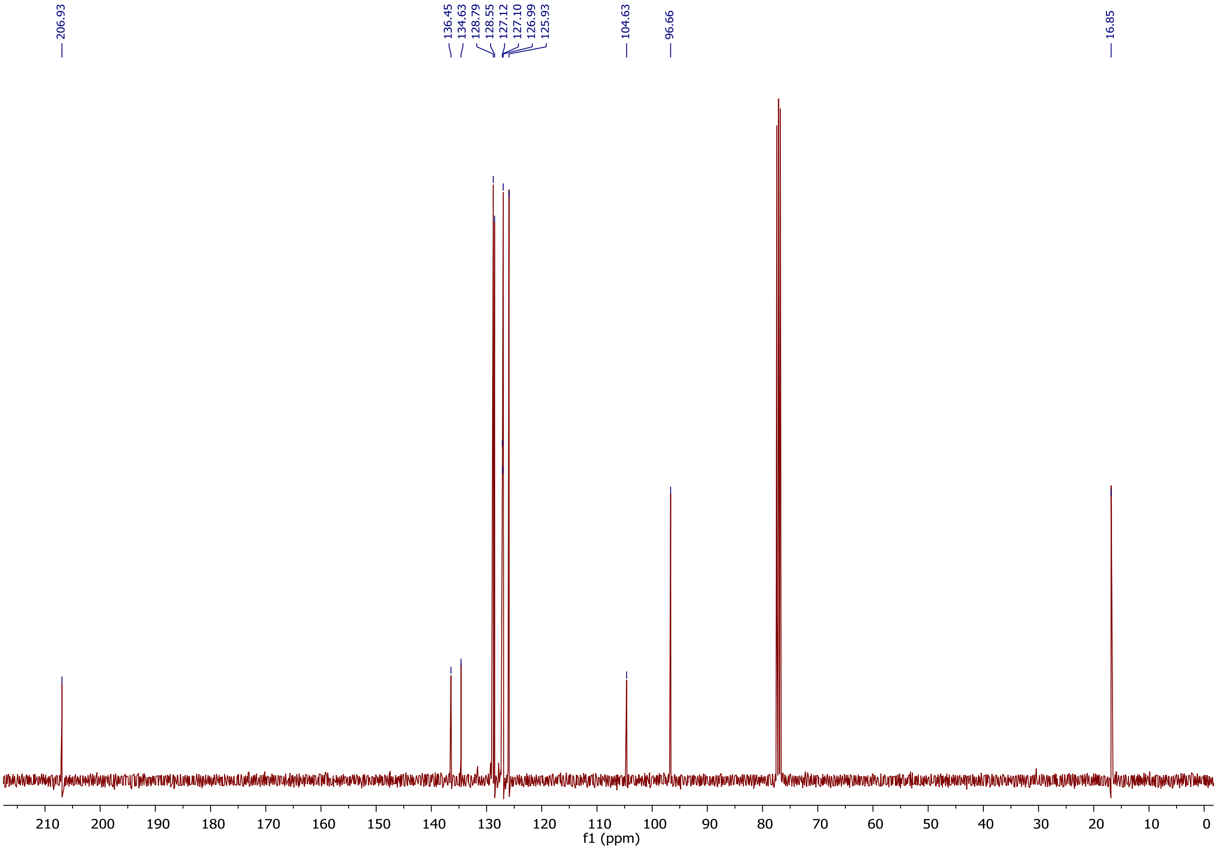
206.93
136.45
134.63
128.79
128.55
127.12
127.10
126.99
125.93
104.63
96.66
16.85
210 200 190 180 170 160 150 140 130 120 110 100 90 80 70 60 50 40 30 20 10 0
f1 (ppm)

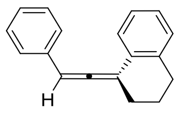

27

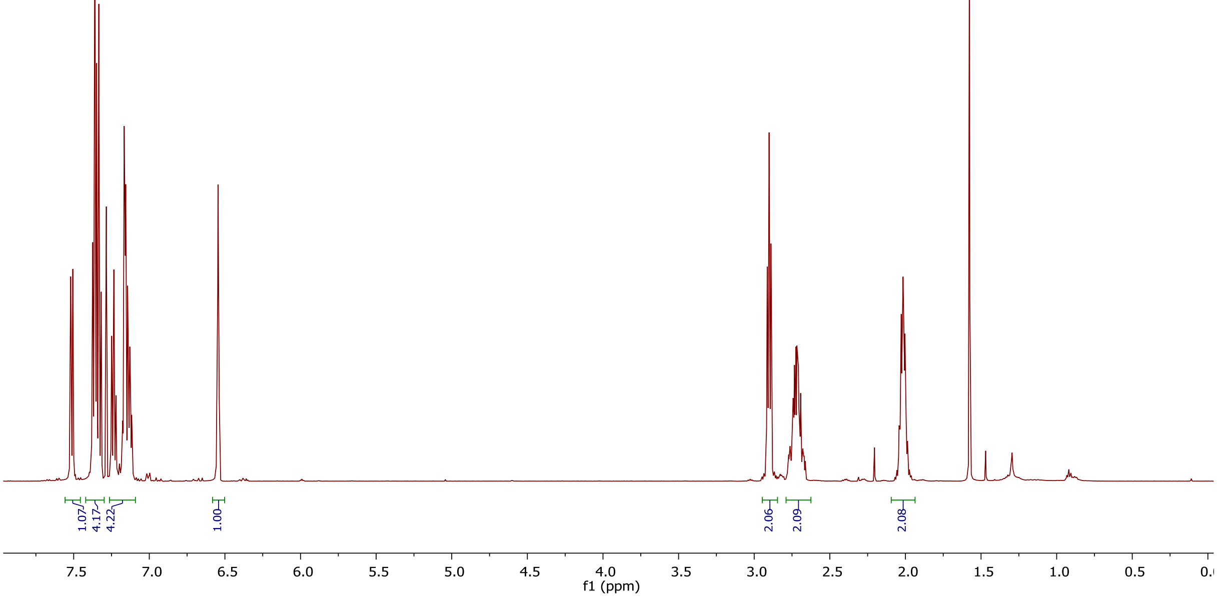
1.07
4.17
4.22
1.00
2.06
2.09
2.08
7.5 7.0 6.5 6.0 5.5 5.0 4.5 4.0 3.5 3.0 2.5 2.0 1.5 1.0 0.5
f1 (ppm)

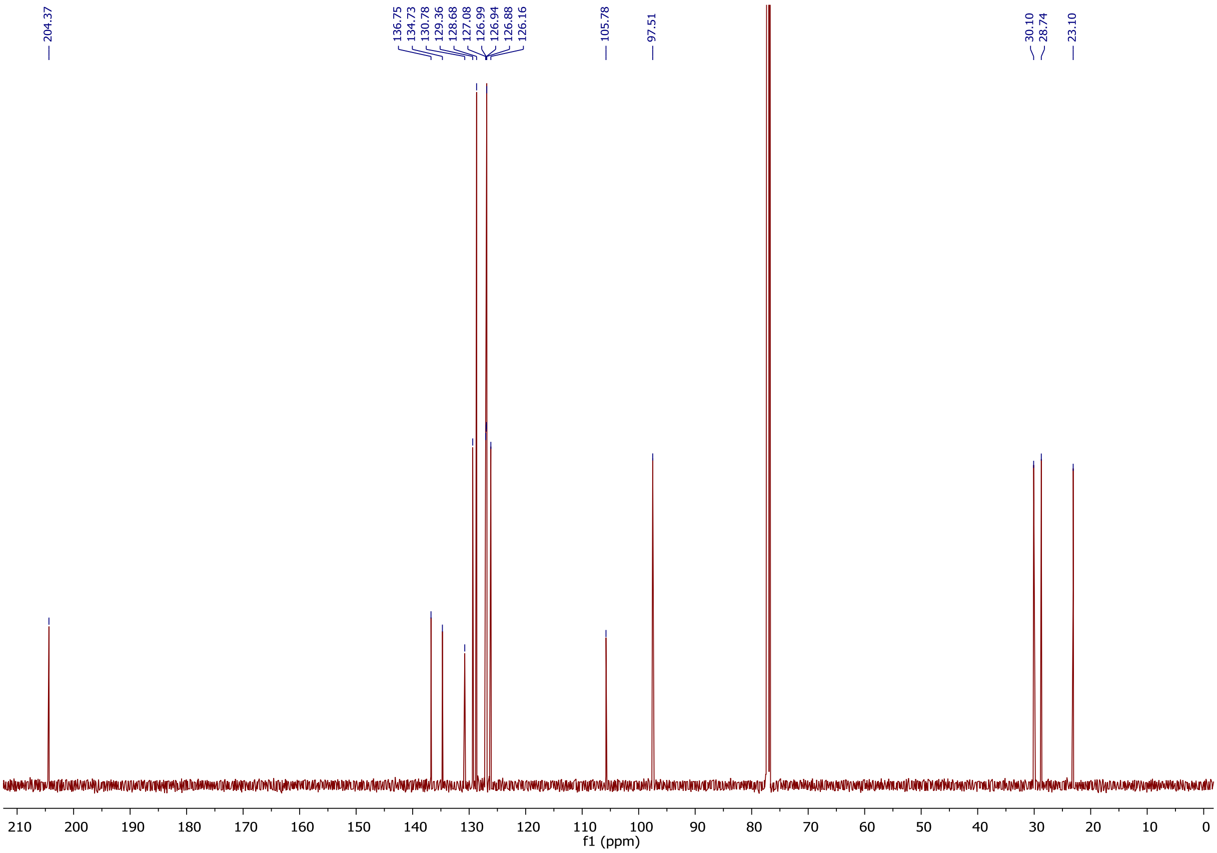
204.37
136.75
134.73
130.78
129.36
128.68
127.08
126.99
126.94
126.88
126.16
105.78
97.51
30.10
28.74
23.10
210 200 190 180 170 160 150 140 130 120 110 100 90 80 70 60 50 40 30 20 10 0
f1 (ppm)

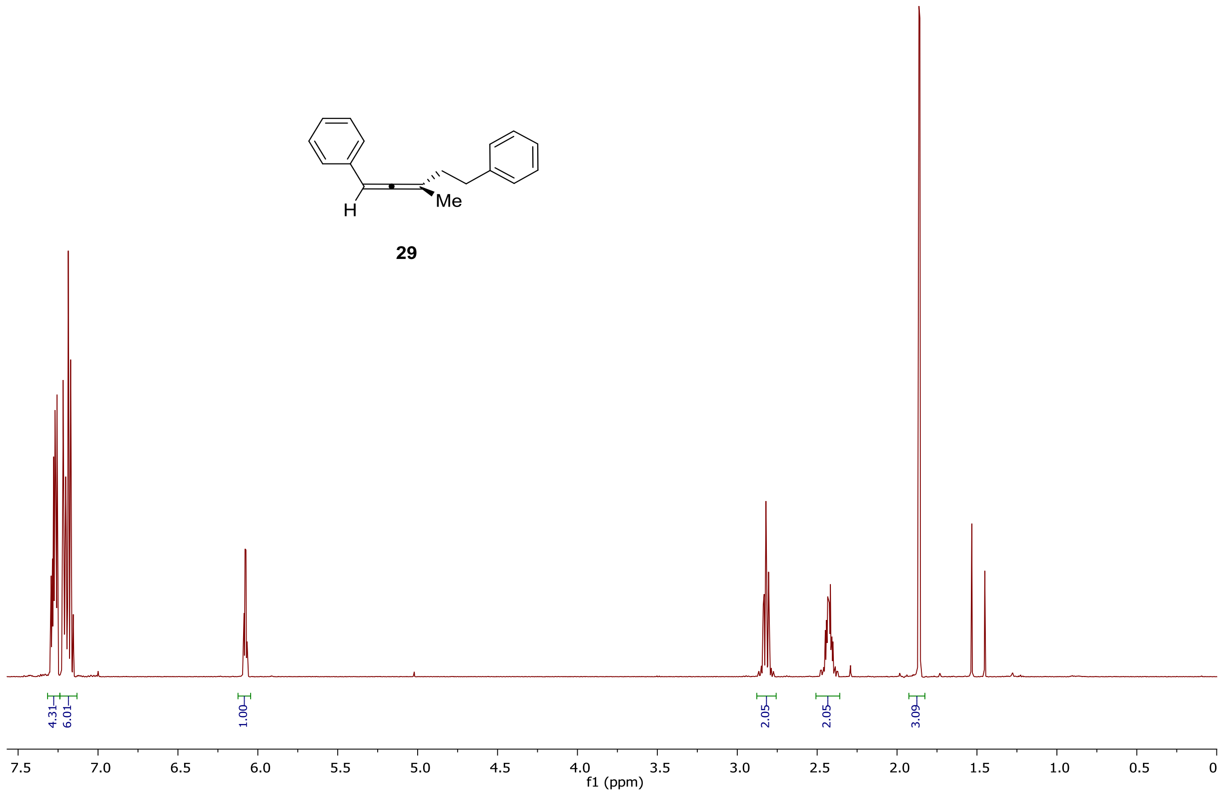
H
Me
29
4.31
6.01
1.00
2.05
2.05
3.09
7.5
7.0
6.5
6.0
5.5
5.0
4.5
4.0
3.5
3.0
2.5
2.0
1.5
1.0
0.5
0
f1 (ppm)

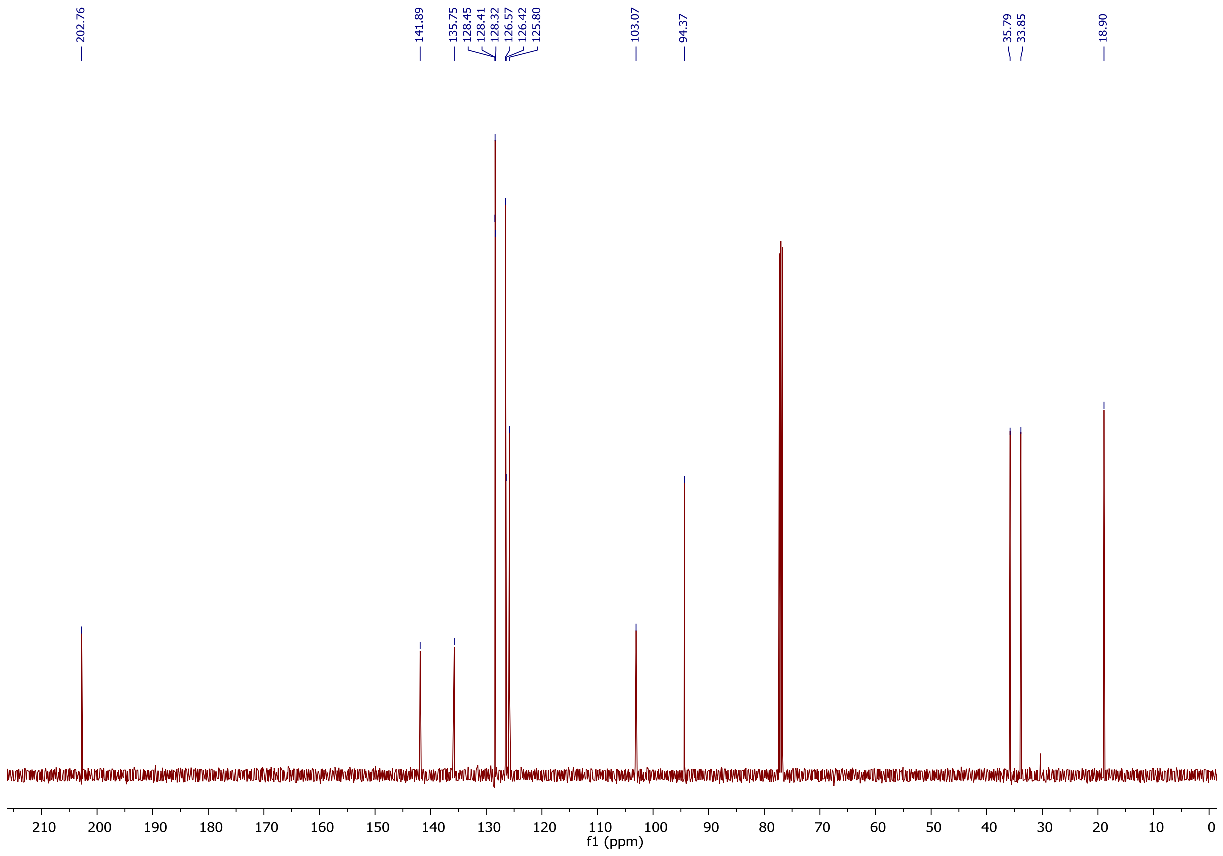
202.76
141.89
135.75
128.45
128.41
128.32
126.57
126.42
125.80
103.07
94.37
35.79
33.85
18.90
210
200
190
180
170
160
150
140
130
120
110
100
90
80
70
60
50
40
30
20
10
0
f1 (ppm)

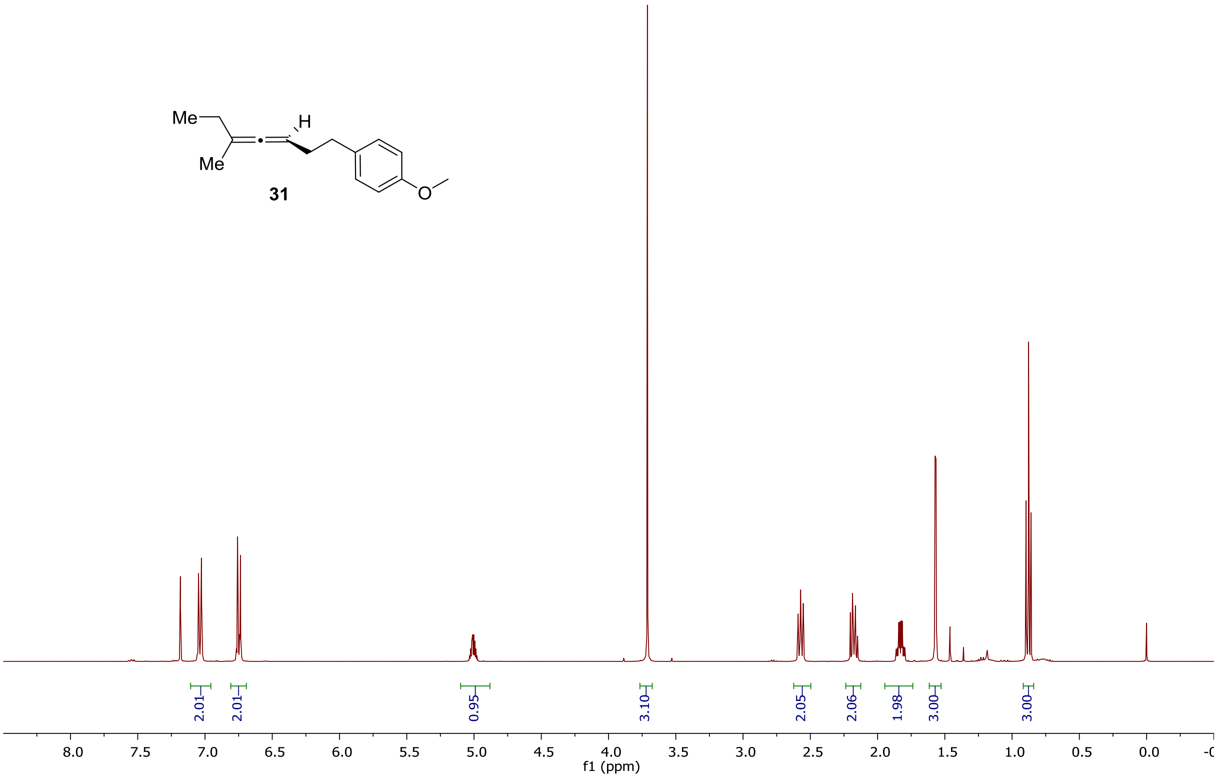
Me
Me
H
O
31
2.01
2.01
0.95
3.10
2.05
2.06
1.98
3.00
3.00
8.0 7.5 7.0 6.5 6.0 5.5 5.0 4.5 4.0 3.5 3.0 2.5 2.0 1.5 1.0 0.5 0.0
f1 (ppm)

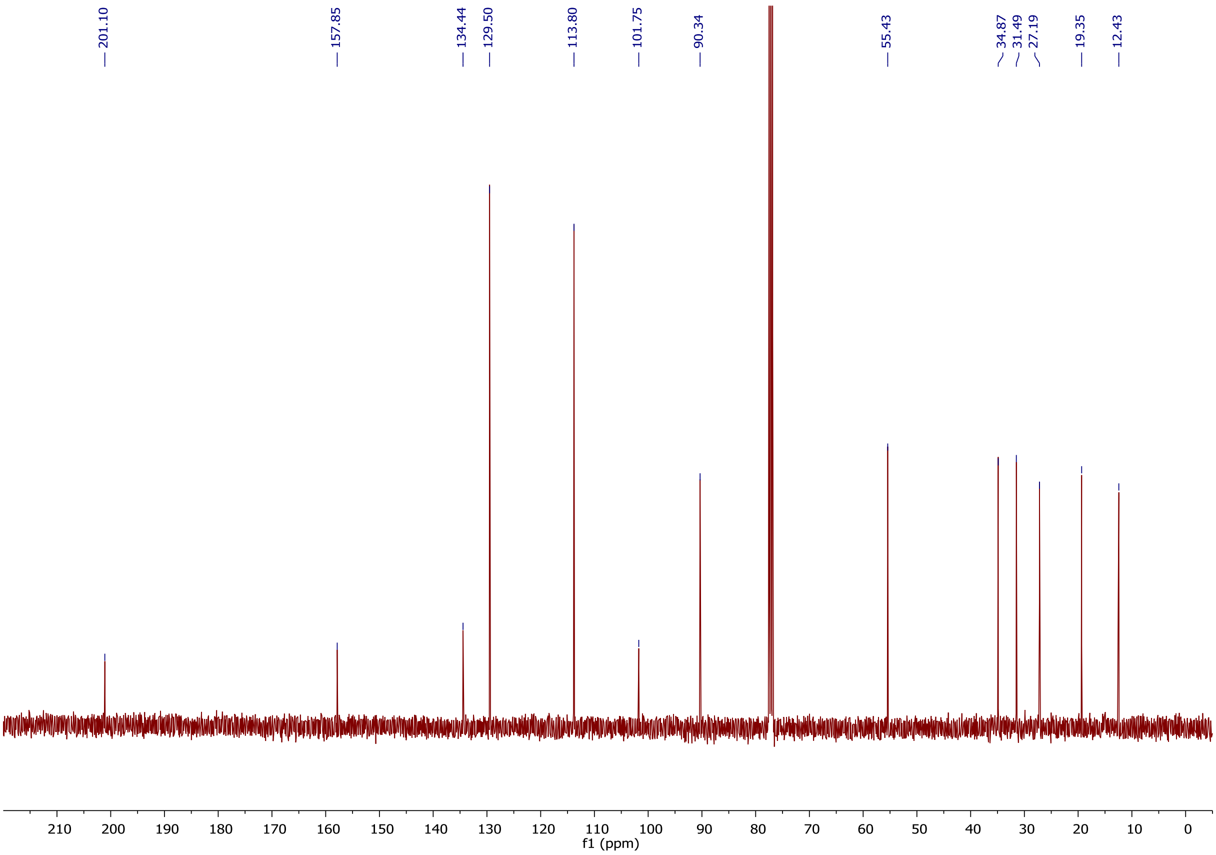
201.10
157.85
134.44
129.50
113.80
101.75
90.34
55.43
34.87
31.49
27.19
19.35
12.43
210 200 190 180 170 160 150 140 130 120 110 100 90 80 70 60 50 40 30 20 10 0
f1 (ppm)

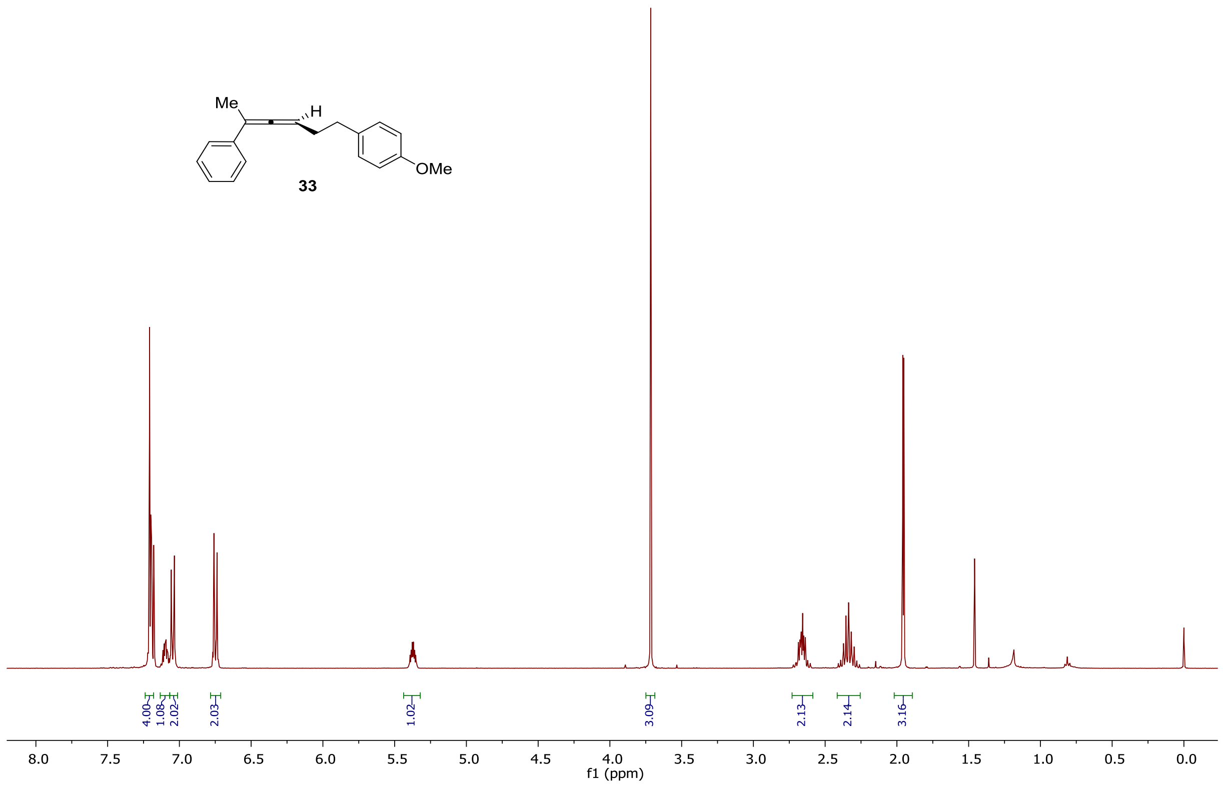
Me
H
OMe
33
4.00
1.08
2.02
2.03
1.02
3.09
2.13
2.14
3.16
8.0 7.5 7.0 6.5 6.0 5.5 5.0 4.5 4.0 3.5 3.0 2.5 2.0 1.5 1.0 0.5 0.0
f1 (ppm)

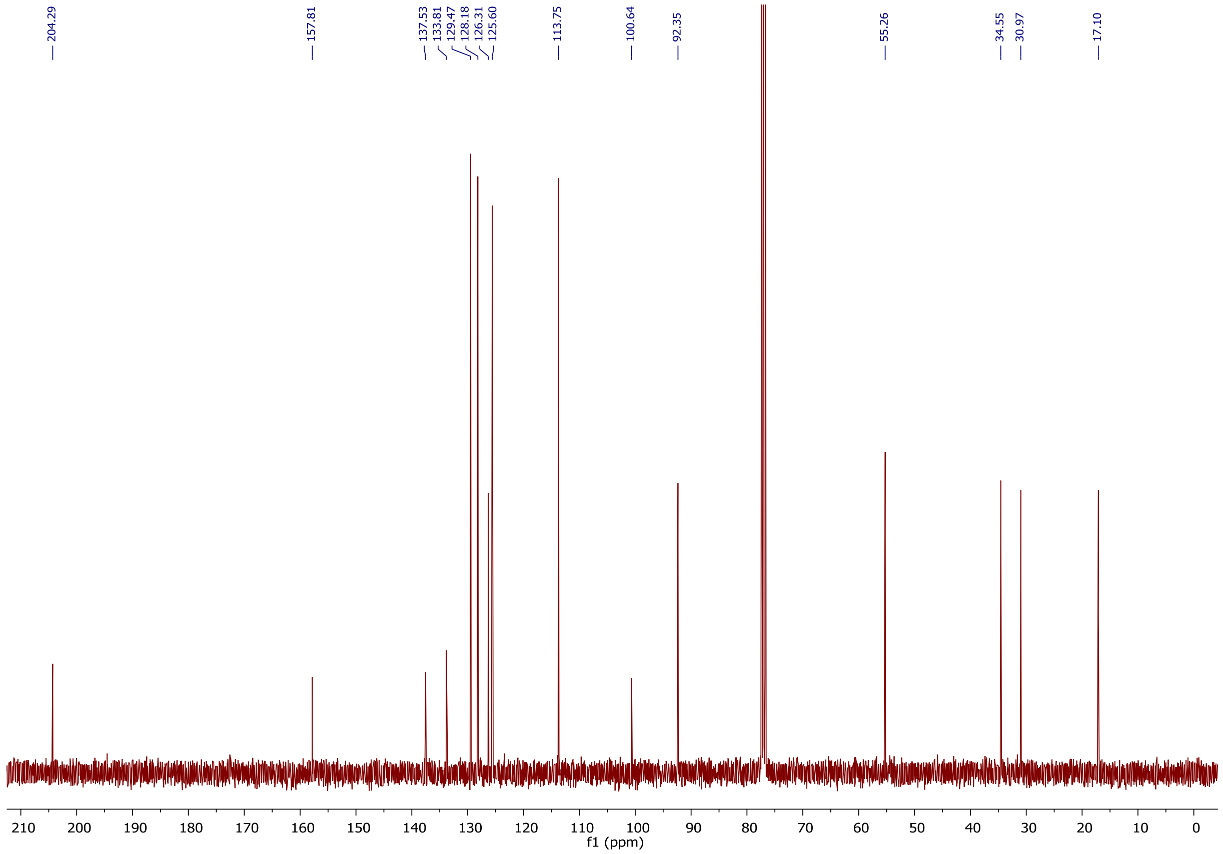
204.29
157.81
137.53
133.81
129.47
128.18
126.31
125.60
113.75
100.64
92.35
55.26
34.55
30.97
17.10
210 200 190 180 170 160 150 140 130 120 110 100 90 80 70 60 50 40 30 20 10 0
f1 (ppm)

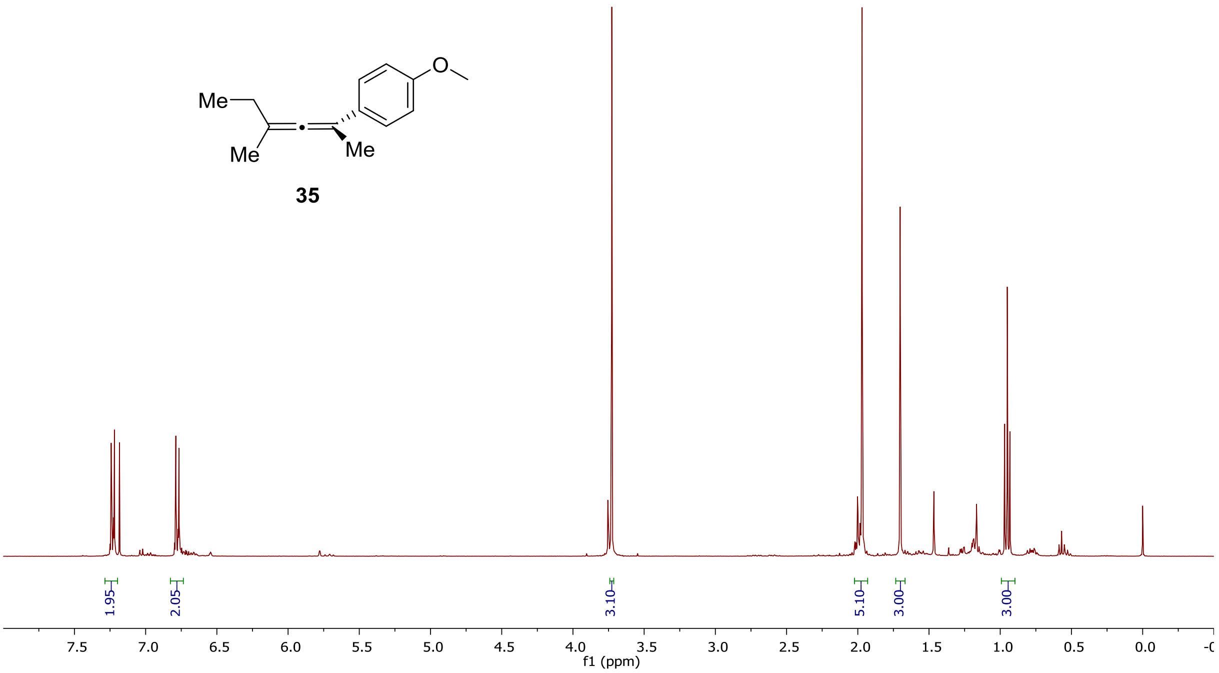
Me
Me
O
Me
1.95
2.05
3.10
5.10
3.00
3.00
7.5 7.0 6.5 6.0 5.5 5.0 4.5 4.0 3.5 3.0 2.5 2.0 1.5 1.0 0.5 0.0
f1 (ppm)

35

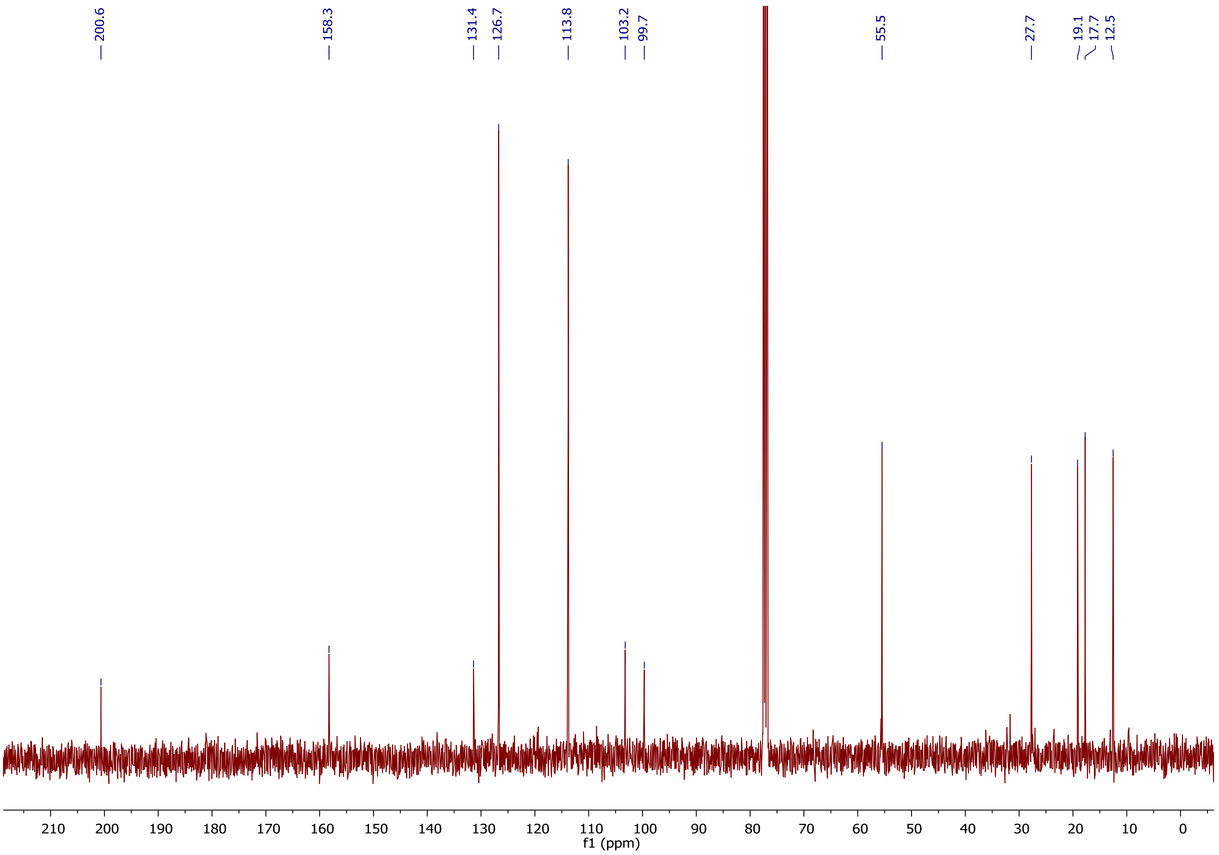
200.6
158.3
131.4
126.7
113.8
103.2
99.7
55.5
27.7
19.1
17.7
12.5
210 200 190 180 170 160 150 140 130 120 110 100 90 80 70 60 50 40 30 20 10 0
f1 (ppm)

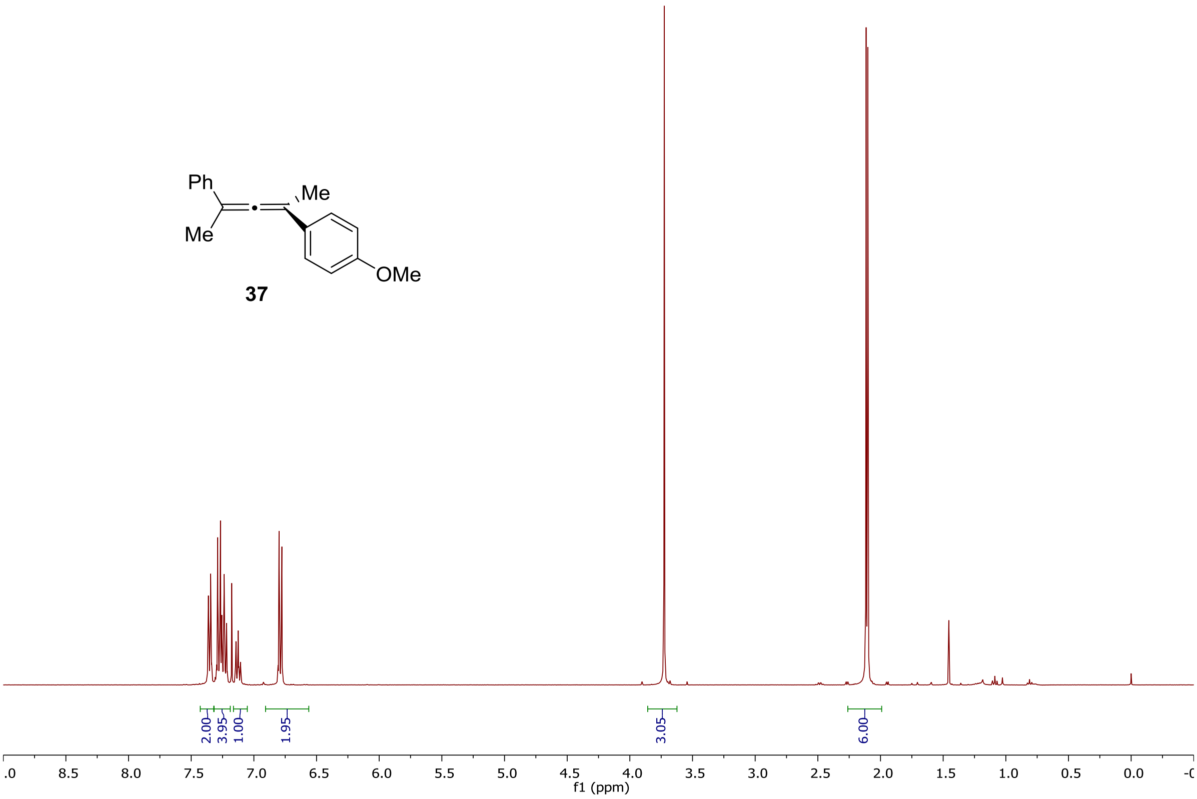
Ph
Me
Me
OMe
37
2.00
3.95
1.00
1.95
3.05
6.00
.0 8.5 8.0 7.5 7.0 6.5 6.0 5.5 5.0 4.5 4.0 3.5 3.0 2.5 2.0 1.5 1.0 0.5 0.0
f1 (ppm)

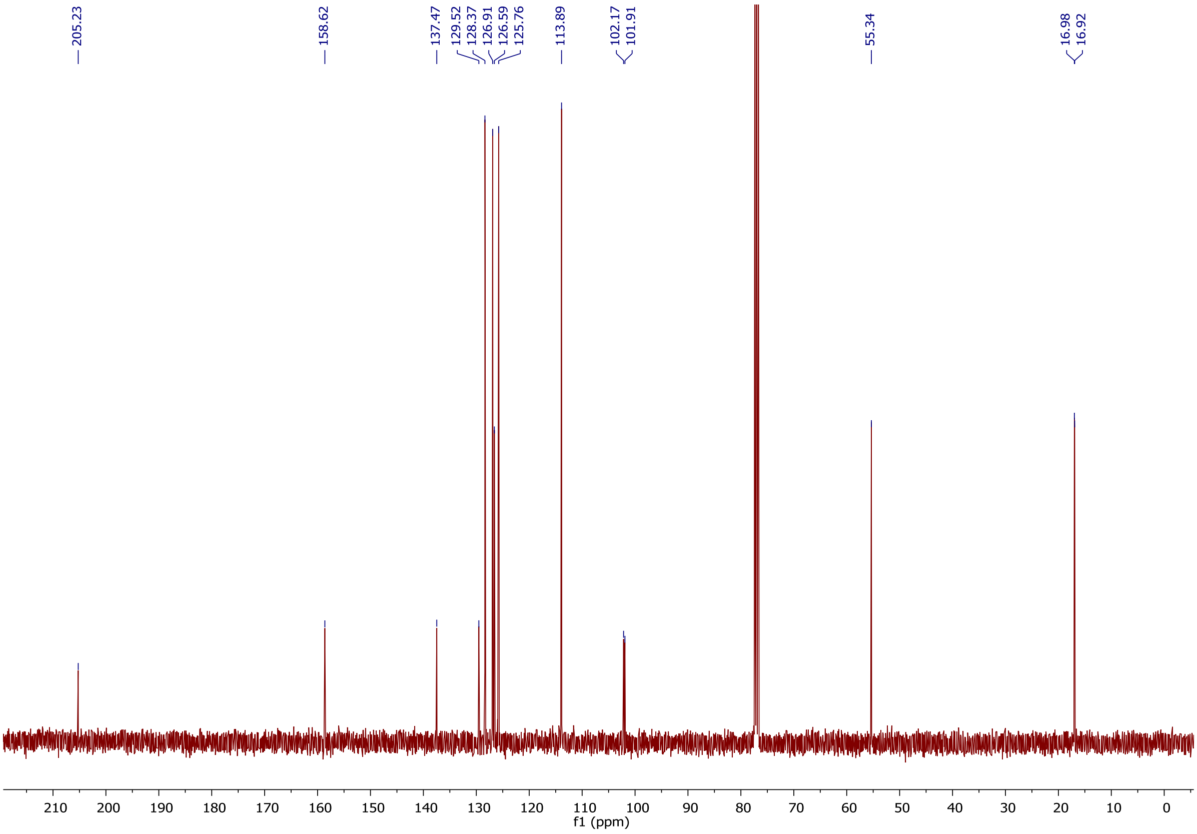
205.23
158.62
137.47
129.52
128.37
126.91
126.59
125.76
113.89
102.17
101.91
55.34
16.98
16.92
210 200 190 180 170 160 150 140 130 120 110 100 90 80 70 60 50 40 30 20 10 0
f1 (ppm)
